# Neoantigen-augmented iPSC cancer vaccine combined with radiotherapy promotes antitumor immunity in poorly immunogenic cancers

**DOI:** 10.1038/s41541-024-00881-5

**Published:** 2024-05-31

**Authors:** Kevin Chih-Yang Huang, William Tzu-Liang Chen, Jia-Yi Chen, Chien-Yueh Lee, Chia-Hsin Wu, Chia-Ying Lai, Pei-Chen Yang, Ji-An Liang, An‑Cheng Shiau, K. S. Clifford Chao, Tao-Wei Ke

**Affiliations:** 1 Department of Biomedical Imaging and Radiological Science, China Medical University, Taichung, 406040 Taiwan, ROC; 2 Translation Research Core, China Medical University Hospital, China Medical University, Taichung, 404327 Taiwan, ROC; 3 Cancer Biology and Precision Therapeutics Center, China Medical University, Taichung, 406040 Taiwan, ROC; 4 Department of Surgery, School of Medicine, China Medical University, Taichung, 406040 Taiwan, ROC; 5 Department of Colorectal Surgery, China Medical University HsinChu Hospital, China Medical University, HsinChu, 302 Taiwan, ROC; 6 Department of Colorectal Surgery, China Medical University Hospital, China Medical University, Taichung, 404327 Taiwan, ROC; 7 Proton Therapy and Science Center, China Medical University Hospital, China Medical University, Taichung, 404327 Taiwan, ROC; 8 Innovation Frontier Institute of Research for Science and Technology, National Taipei University of Technology, Taipei, 106344 Taiwan, ROC; 9 Department of Electrical Engineering, National Taipei University of Technology, Taipei, 106344 Taiwan, ROC; 10 Department of Biomedical Engineering, China Medical University, Taichung, 406040 Taiwan, ROC; 11 Bioinformatics and Biostatistics Core, Centers of Genomic and Precision Medicine, National Taiwan University, Taipei, 10055 Taiwan, ROC; 12 Department of Radiation Oncology, China Medical University Hospital, China Medical University, Taichung, 404327 Taiwan, ROC; 13 Department of Radiotherapy, School of Medicine, China Medical University, Taichung, 406040 Taiwan, ROC; 14 School of Chinese Medicine and Graduate Institute of Chinese Medicine, China Medical University, Taichung, 406040 Taiwan, ROC

**Keywords:** Colon cancer, Vaccines

## Abstract

Although irradiated induced-pluripotent stem cells (iPSCs) as a prophylactic cancer vaccine elicit an antitumor immune response, the therapeutic efficacy of iPSC-based cancer vaccines is not promising due to their insufficient antigenicity and the immunosuppressive tumor microenvironment. Here, we found that neoantigen-engineered iPSC cancer vaccines can trigger neoantigen-specific T cell responses to eradicate cancer cells and increase the therapeutic efficacy of RT in poorly immunogenic colorectal cancer (CRC) and triple-negative breast cancer (TNBC). We generated neoantigen-augmented iPSCs (NA-iPSCs) by engineering AAV2 vector carrying murine neoantigens and evaluated their therapeutic efficacy in combination with radiotherapy. After administration of NA-iPSC cancer vaccine and radiotherapy, we found that ~60% of tumor-bearing mice achieved a complete response in microsatellite-stable CRC model. Furthermore, splenocytes from mice treated with NA-iPSC plus RT produced high levels of IFNγ secretion in response to neoantigens and had a greater cytotoxicity to cancer cells, suggesting that the NA-iPSC vaccine combined with radiotherapy elicited a superior neoantigen-specific T-cell response to eradicate cancer cells. The superior therapeutic efficacy of NA-iPSCs engineered by mouse TNBC neoantigens was also observed in the syngeneic immunocompetent TNBC mouse model. We found that the risk of spontaneous lung and liver metastasis was dramatically decreased by NA-iPSCs plus RT in the TNBC animal model. Altogether, these results indicated that autologous iPSC cancer vaccines engineered by neoantigens can elicit a high neoantigen-specific T-cell response, promote tumor regression, and reduce the risk of distant metastasis in combination with local radiotherapy.

## Introduction

Harnessing the immune system to eradicate malignant cells is becoming a powerful therapeutic strategy for cancer treatment. The success of immune checkpoint inhibitors (ICIs) in treating several malignancies has greatly advanced basic research and clinical studies on cancer immunotherapy. However, more than 50% of cancer patients fail to respond to ICIs, suggesting that novel immunotherapeutic strategies are needed to treat patients with cancers^1^.

Induced pluripotent stem cells (iPSCs) have been reported to share tumor-associated antigen (TAA) profiles with cancer cells^2–4^, including melanoma, breast, and pancreatic cancer^2,5,6^. These genes, which are called iPSC-cancer signature genes, are highly expressed in pluripotent populations but marginally or not at all expressed in somatic tissues. iPSC-cancer signature genes-derived peptides exhibit immunogenic and provide therapeutic benefits for cancer treatment^2,5,7–11^. Kooreman et al. showed that autologous iPSCs elicit antitumor immune response with increased lymphocyte infiltration and cytokine release^9,12^, which implies that undifferentiated whole iPSCs are immunogenic and can be used as multiple TAAs cancer vaccines^12–14^. However, iPSC-based cancer vaccines cannot induce effective antitumor immunity against existing tumors, indicating that irradiated iPSCs lysates can only act as prophylactic cancer vaccines for cancer treatment^2,5,10,15,16^. Therefore, combining these vaccines with other strategies to increase the therapeutic efficacy of iPSC-based cancer vaccines is necessary.

Efficient vaccination against cancer relies on the induction of adaptive immune responses. Although prophylactic cancer vaccines have been successful in the prevention of cervical cancer and hepatocellular carcinoma by targeting viral antigens^17,18^, there is no therapeutic cancer vaccine that eradicates existing cancer cells by targeting shared TAAs. Due to immune tolerance and on-target off-tumor side effects, the results of clinical trials are still unsatisfactory^19^. The advent of next-generation sequencing (NGS) has enhanced the possibility of identifying new tumor-specific targets in oncoimmunology and accelerated the development of novel immunotherapeutic strategies with broader coverage for patients with cancer, such as tumor-specific antigen (neoantigen)-derived cancer vaccines^20^. Neoantigens are derived from mutated genes that are specific and recognized as non-self antigens. Neoantigen-based immunotherapies, such as adoptive T cell therapy and cancer vaccines, have been reported to induce remission in preclinical studies and several clinical trials^21–23^. Recent studies indicated that the quantity and quality of the neoantigen-reactive T-cell response contributed to the therapeutic efficacy of ICIs, cancer vaccines, and cryothermal therapy^24–27^, suggesting that the strong antitumor immune response elicited by these immunotherapeutic strategies was mainly mediated by neoantigen-specific T cells.

In this study, we showed that local radiotherapy enhanced the immunogenicity of iPSC-based cancer vaccines to provide more therapeutic benefit in colorectal cancer (CRC) animal model, which associated with an increase in tumor-infiltrating CD8^+^ effector/memory T and NK cell responses. But we found that the neoantigen-specific T cell response was not significantly increased by iPSC vaccination. To increase the antitumor effects, we generated a neoantigen-augmented iPSC (NA-iPSC) cancer vaccine to elicit neoantigen-specific T cell response. Combination treatment with these NA-iPSC cancer vaccines and local radiotherapy significantly increased the ratio of mice with complete responses and delayed tumor regrowth in a microsatellite-stable colorectal cancer (MSS-CRC) animal model. The isolated splenic CD8^+^ cells elicited IFNγ production in response to neoantigens and showed superior cytotoxic effects on tumor cells. Moreover, these similar therapeutic strategies also delayed tumor growth and reduced the risk of distant metastasis in a poorly immunogenic triple-negative breast cancer animal model. Taken together, these results indicated that neoantigen-augmented iPSC cancer vaccines provide better therapeutic effects against cancer cells and eradicate residual tumor cells in combination with radiotherapy.

## Results

### Local radiotherapy significantly increased the antitumor effect of iPSC-based cancer vaccines

**Fig. 1:**
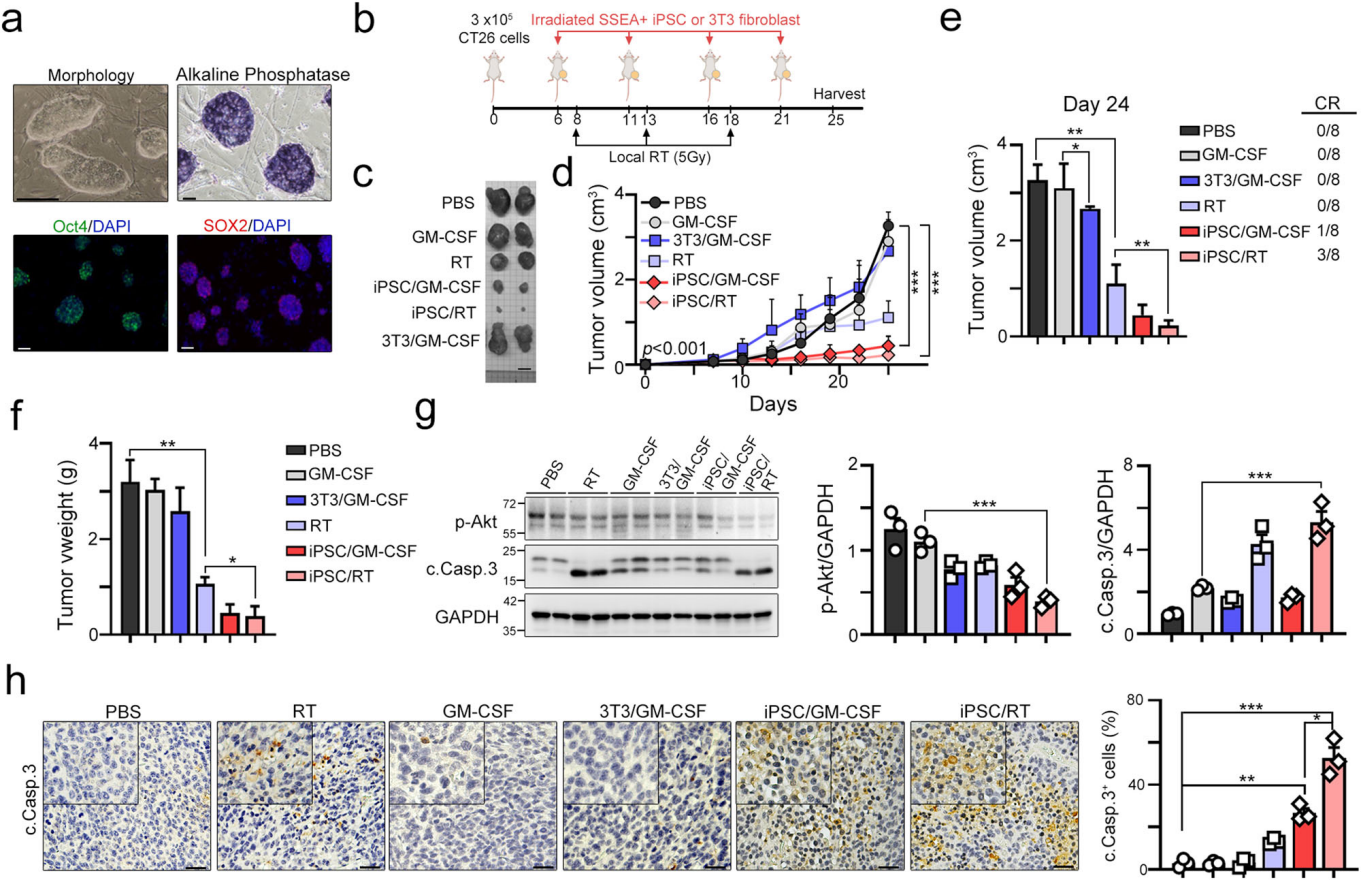
Vaccination with irradiated iPSC-whole-cell lysate combined with RT leads to tumor remission. **a** The identification and characterization of mouse iPSCs after 5 passages. Scale bar = 50 μm. **b** Schematic diagram of the irradiated iPSC vaccine and RT treatment. A total of 3 × 10^5^ CT26 cells were subcutaneously inoculated into BALB/c mice for 6 days, and irradiated iPSCs or fibroblasts (1 × 10^5^ cells, 50 Gy) were subcutaneously administered adjuvant GM-CSF (50 ng/shot) on Days 6, 11, 16 and 21. Local radiotherapy was given on Days 8, 13 and 18. The animal experiments were repeated twice, independently. **c** The representative image of the resected tumor tissues. Scale bar = 1 cm. **d** Tumor volume was calculated every 3 days (*n* = 8). **e** Tumor volume was calculated (Day 24, *n* = 8). **f** The resected tumor weight was measured (Day 24, *n* = 8). **g** The resected tumor tissues were analyzed by immunoblotting (*n* = 3). **h** The percentage of apoptotic cells (cleaved caspase-3^+^ cells) was evaluated by immunohistochemical analysis (*n* = 3). Scale bar = 30 μm. Significance was calculated with multiple *t* tests analysis and is presented as mean ± s.e.m. *p < 0.05; **p < 0.01 and ***p < 0.001.

Radiation can increase cancer immunogenicity by triggering STING-mediated type I IFN production, promoting MHC class I presentation, and proinflammatory cytokine release^28–32^. To demonstrate that the beneficial effect of iPSC-based cancer vaccines can be enhanced by radiotherapy, we generated mouse iPSCs from BALB/c fibroblasts and verified them by two well-known iPSC markers, Oct4 and Sox2 (Fig. 1a). We then examined the activity of alkaline phosphatase (AP), which has also been shown to be upregulated in pluripotent stem cells, including undifferentiated embryonic stem cells (ESCs), embryonic germ cells (EGCs) and iPSCs^11^. The results showed that iPSC clones highly expressed AP (Fig. 1a). Then, we used undifferentiated iPSCs irradiated with 50 Gy one hour before vaccination as autologous cancer vaccines^5,8^ and combined them with local radiotherapy to evaluate their therapeutic efficacy (Fig. 1b). As shown in Fig. 1b, 3 × 10^5^ CT26 cells were subcutaneously inoculated into BALB/c mice for 6 days, and irradiated undifferentiated iPSCs or fibroblasts (1 × 10^5^ cells, 50 Gy) were subcutaneously administered with the adjuvant GM-CSF (50 ng/shot) on Days 6, 11, 16 and 21. Local radiotherapy was given on Days 8, 13 and 18. We found that the irradiated iPSC adjuvant with GM-CSF significantly decreased tumor growth compared with the 3T3 fibroblast adjuvant with GM-CSF (Fig. 1c, d). Moreover, we found that 12.5% (1/8 = 12.5%) of mice achieved a complete response after 4 administrations (Fig. 1d). Local radiotherapy led to ~50% tumor regression, but there was no impact on the percentage of mice that achieved a complete response (CR). When irradiated iPSCs were combined with local radiotherapy, the therapeutic efficacy was superior to that of iPSC/GM-CSF. A total of 37.5% of vaccinated mice achieved a complete response, suggesting that radiotherapy combined with irradiated iPSC cancer vaccines might increase antitumor immunity and the therapeutic response. Consistently, the tumor volume and tumor weight also showed remarkable tumor regression in the iPSC/RT subgroup (Fig. 1e, f, Day 24). The survival marker p-Akt was significantly downregulated, and the apoptotic marker caspase-3 was cleaved in the iPSC plus RT subgroup (Fig. 1g). The number of cleaved caspase-3^+^ cells was also significantly increased in the iPSC plus RT subgroup (Fig. 1h). Altogether, these results showed that local radiotherapy enhanced the therapeutic effect of iPSC-based cancer vaccines.

**Fig. 2:**
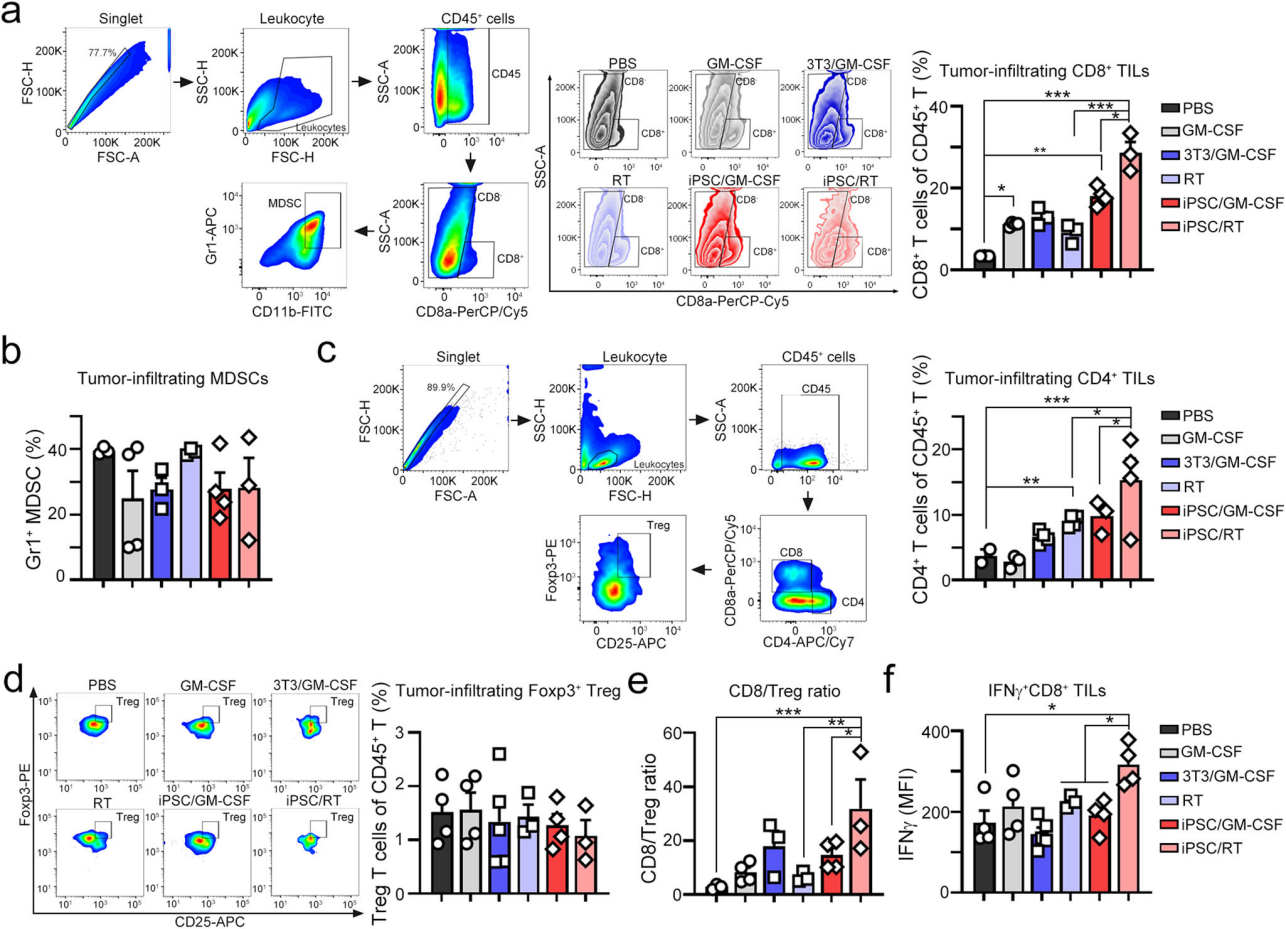
Vaccination with irradiated iPSCs combined with RT remarkably restored the immune cell profiles within the tumor microenvironment. **a** The gating strategy for tumor-infiltrating lymphocyte subsets. The subset of cytotoxic CD8^+^ cells within tumors was extracted for flow cytometry (*n* = 3–4). **b** The subset of Gr1^+^CD11b^+^ MDSCs within tumors was extracted for flow cytometry (*n* = 3–4). **c** The subset of CD4^+^ cells within tumors was extracted for flow cytometry (*n* = 3–4). **d** The subset of regulatory T lymphocytes CD4^+^CD25^+^Foxp3^+^ cells (T_reg_) within tumors was extracted for flow cytometry (*n* = 3–4). **e** The ratio of tumor-infiltrating CD8/Foxp3^+^ Treg cells was analyzed (*n* = 3–4). **f** The subset of cytotoxic IFNγ^+^CD8^+^ cells within tumors was extracted for flow cytometry (*n* = 3–4). Significance was calculated with multiple *t* tests analysis and is presented as mean ± s.e.m. *p < 0.05; **p < 0.01 and ***p < 0.001.

The antitumor effect of irradiated iPSCs was associated with their immunogenicity^2,5^, which influenced the infiltration of immune cells within tumors. To evaluate the immune cell profile within the tumor microenvironment after combination treatment, tumor-infiltrating immune cells were isolated from resected tumors (Fig. 2a). Local radiotherapy or iPSC/GM-CSF alone significantly increased the percentage of tumor-infiltrating CD8^+^ immune cells (Fig. 2a). 3T3/GM-CSF slightly increased the percentage of tumor-infiltrating CD8^+^ immune cells compared to iPSC/GM-CSF alone (Fig. 2a), suggesting that undifferentiated iPSCs were more immunogenic than 3T3 fibroblast cells. Furthermore, combination with iPSC and local RT remarkably increased the density of tumor-infiltrating CD8^+^ immune cells (Fig. 2a). But there was no difference in the frequency of tumor-infiltrating and Gr1^+^ CD11b^+^ MDSCs among these subgroups (Fig. 2b). Additionally, combined treatment remarkably upregulated tumor-infiltrating CD4^+^ immune cells compared to local radiotherapy alone (*p* < 0.05, Fig. 2c). The combined treatment elicited less infiltration of Foxp3^+^CD25^+^ Tregs in the iPSC/RT subgroup (Fig. 2d). The ratio of tumor-infiltrating CD8^+^/Foxp3^+^CD25^+^ Tregs and the frequency of cytotoxic IFNγ^+^ CD8^+^ cells were significantly higher in the iPSC/RT subgroup, suggesting a predominance of immune system activation over suppression within the tumor microenvironment (Fig. 2e, f). Altogether, these results showed that local radiotherapy together with an undifferentiated iPSC cancer vaccine elicited greater antitumor therapeutic efficacy.

**Fig. 3:**
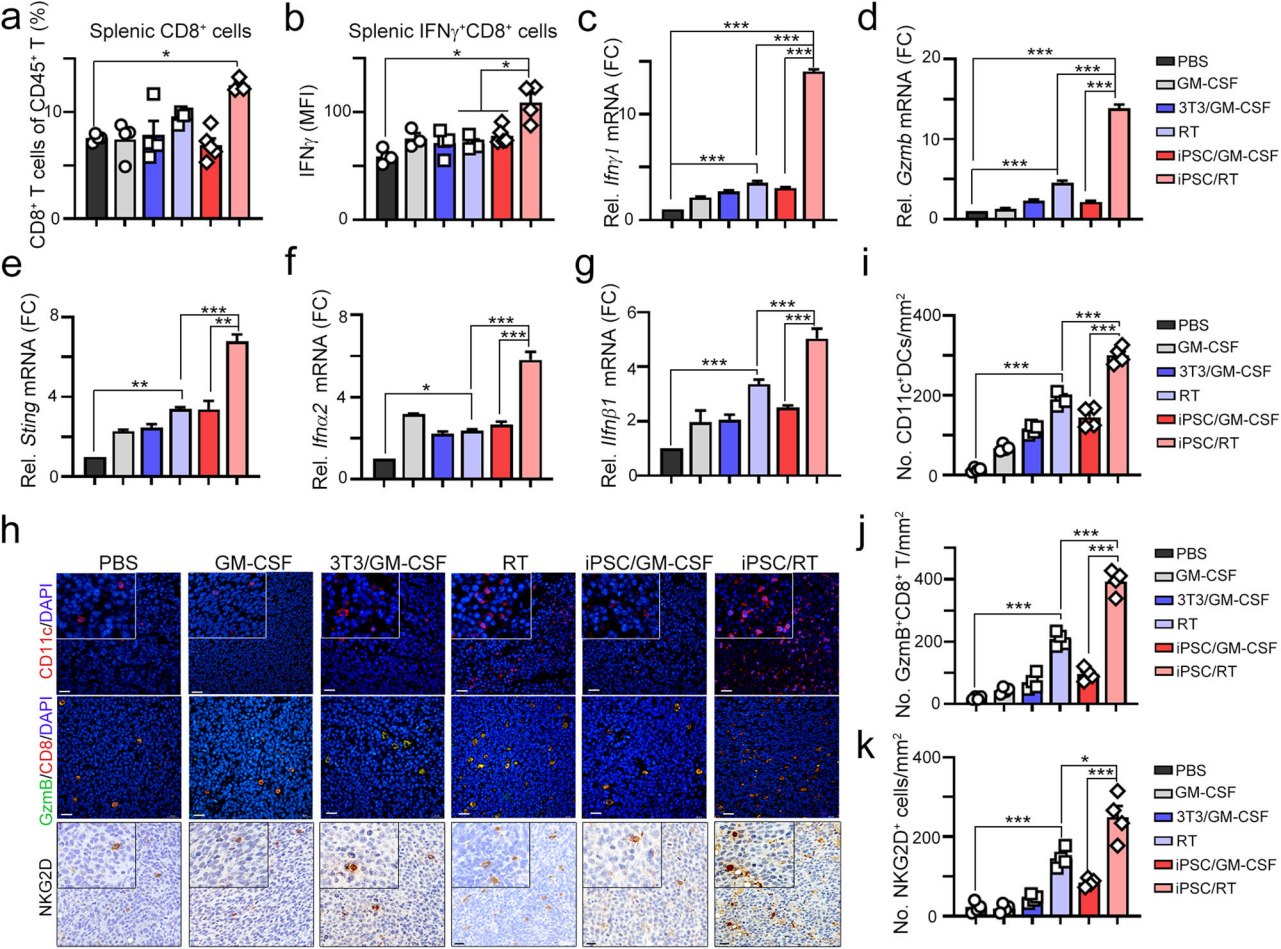
Vaccination with irradiated iPSCs combined with RT markedly increased antitumor immunity. **a** The subset of cytotoxic CD8^+^ cells within the spleen was extracted for flow cytometry (*n* = 3–4). **b** The subset of cytotoxic IFNγ^+^CD8^+^ cells within the spleen was extracted for flow cytometry (*n* = 3–4). **c** The level of *Ifnγ* mRNA within resected tumors was analyzed by qRT‒PCR (*n* = 3–4). **d** The level of *Gzmb* mRNA within resected tumors was analyzed by qRT‒PCR (*n* = 3–4). **e** The level of *Tmem173* (*Sting)* mRNA within resected tumors was analyzed by qRT‒PCR (*n* = 3–4). **f** The level of *Ifnα2* mRNA within resected tumors was analyzed by qRT‒PCR (*n* = 3–4). **g** The level of *Ifnβ1* mRNA within resected tumors was analyzed by qRT‒PCR (*n* = 3–4). **h** Tumor-infiltrating CD11c^+^ DCs, GzmB^+^CD8^+^ T cells, and NKG2D^+^NK cells were evaluated by immunofluorescence staining. Scale bar = 50 μm. **i** The quantification of tumor-infiltrating CD11^+^ DCs is shown (*n* = 4). **j** The quantification of tumor-infiltrating GzmB^+^CD8^+^ T cells is shown (*n* = 4). **k** The quantification of tumor-infiltrating NKG2D^+^ NK cells is shown (*n* = 4). Significance was calculated with multiple *t* tests analysis and is presented as mean ± s.e.m. *p < 0.05; **p < 0.01 and ***p < 0.001.

Moreover, the frequency of splenic CD8^+^ and cytotoxic IFNγ^+^ CD8^+^ T cells was also markedly increased in the iPSC/RT subgroup (Fig. 3a, b). These results showed that iPSCs combined with RT synergistically elicited a systematic antitumor immune response. Moreover, the levels of *Ifnγ* and *Gzmb* in resected tumors were also significantly increased in the iPSC/RT subgroup (Fig. 3c, d). Previous studies showed that activation of STING by RT can increase the antitumor immunity of cancer vaccines in murine pancreatic cancer^33^. Therefore, we analyzed whether radiotherapy activated cGAS/STING signaling to increase the clinical efficacy of iPSC-based cancer vaccines. We first examined the expression levels of *Sting (Tmem173), Ifnα2* and *Ifnβ1* in these subgroups. We found that local radiotherapy significantly upregulated *Sting (Tmem173), Ifnα2* and *Ifnβ1* (Fig. 3e–g). However, iPSCs alone had little influence on the levels of *Sting (Tmem173), Ifnα2* and *Ifnβ1* (Fig. 3e–g), suggesting that radiotherapy promoted type I IFN production via STING to increase the antitumor efficacy of iPSC cancer vaccines. Furthermore, the infiltration of CD11c^+^ dendritic cells (DCs), GzmB^+^ T cells, and NKG2D^+^ NK cells was significantly increased in the iPSC/RT subgroup compared to the other subgroups (Fig. 3h–k). Taken together, these results showed that combination treatment of RT and irradiated undifferentiated iPSC cancer vaccines increased systematic antitumor immunity to eradicate tumor cells.

### iPSC cancer vaccines elicited an insufficient neoantigen-specific T cell response

**Fig. 4:**
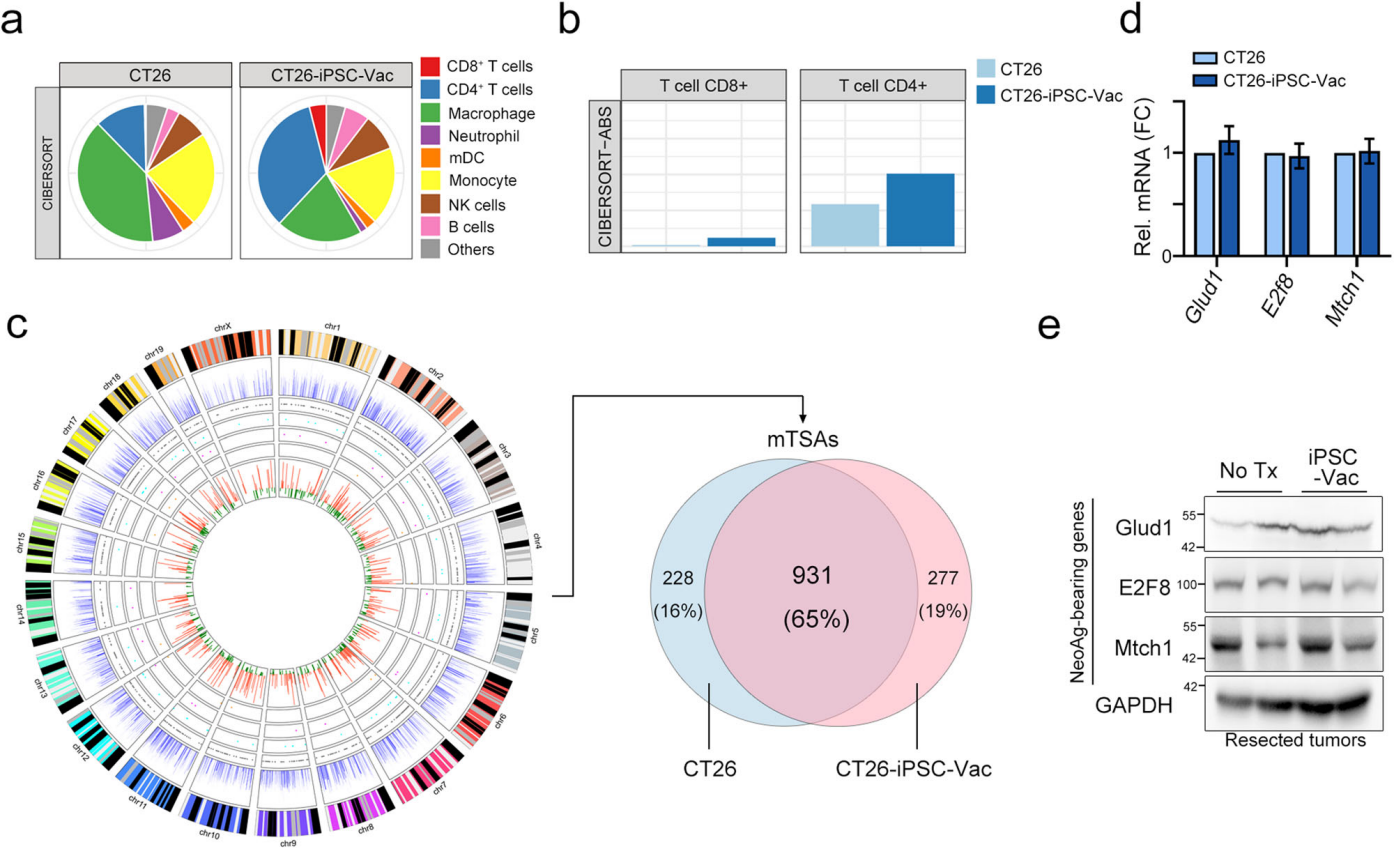
Heterologous neoantigens existed within residual tumors after iPSC-based cancer vaccine administration. **a** The immune cell profile in resected tumor tissues from CT26 and CT26-iPSC-Vac. **b** The tumor-infiltrating CD4 and CD8^+^ T cells were evaluated by TIMER2.0. **c** Circos diagram showing (outer to inner): Track 1: chromosome & cytoband; Track 2: all gene expression values normalized by log_10_(CPM) from RNA-seq (blue barplot); Track 3: mTSAs from missense variants (gray dots); Tracker 4: mTSAs from frameshift variants (cyan dots); Track 5: mTSAs from splice region variants (magenta dots); Track 6: mTSAs from inframe insertion/deletion (orange dots); Tracker 7: IC50 values normalized by -log_10_(IC50/500) (red bars indicate IC50 < 500 nM; green bars indicate IC50 > 500 nM). Venn diagram of identified missense mutations between CT26 and CT26-iPSC-Vac tumors. Reactive peptide sequences and their IC50 values as calculated by NetMHCpan ver3.0. **d** The neoantigen-bearing gene expression in resected tumor tissues was evaluated by qRT‒PCR (*n* = 3). **e** The neoantigen-bearing protein expression in resected tumor tissues was evaluated by western blotting (*n* = 3). Significance was calculated with multiple *t* tests analysis and is presented as mean ± s.e.m.

Neoantigens derived from nonsynonymous mutations of malignant tumor cells could elicit strong antitumor immune responses compared with tumor-associated antigens (TAAs). Therefore, neoantigen-based cancer therapeutic vaccines have been proven to be a promising antitumor immunotherapy strategy with maximized therapeutic efficacy and minimized risk of autoimmunity^22^. Since the TAA profiles were shared between iPSCs and cancer cells, we assumed that the extent of the neoantigen-specific T cell response might be important to increase the therapeutic efficacy of iPSC cancer vaccines. To comprehensively understand the genomic profile and gene expression within iPSC-vaccinated tumors, we analyzed iPSC-vaccinated residual tumors and untreated tumors by whole-exome sequencing (WES) and transcriptome sequencing (RNA-seq). We first evaluated immune cell profiling by TIMER2.0 immune estimation according to the transcriptomic results^34^. We found a significant immune cell profile change between CT26-tumor and CT26-iPSC-Vac-tumor tissues (Fig. 4a, b). The infiltration of CD4^+^ and CD8^+^ T cells was markedly increased in CT26-iPSC-Vac tumor tissue (Fig. 4b). By integrating WES and RNA-seq results, we identified high-confidence single nucleotide variations (SNVs) in exons (missense mutations) that derived tumor-specific antigens (mTSAs, neoantigens) that can be presented by the mouse HLA complex, H2-K^d^, H2D^d^ and H2-L^d^ in CT26 resected tumor (Fig. 4c). There were 1158 and 1208 missense mutation-derived neoantigens in CT26 resected tumor and CT26-iPSC-Vac residual tumor (Fig. 4c, Supplementary Tables 3 and 4). We then compared the difference in mTSAs between CT26-tumor and CT26-iPSC-Vac-tumor tissues. We found that 65% of mTSAs (931 mTSAs, IC_50_ < 500 nM) were still shared between CT26-tumor and CT26-iPSC-Vac-tumor tissues (Fig. 4c, Supplementary Table 5). We then evaluated the levels of these mTSA-bearing genes (Glud1, Mtch3, and E2f8) by qRT‒PCR and western blotting (Fig. 4d, e). We found no significant differential expression in these three genes (Fig. 4d, e), suggesting that targeting neoantigens to trigger neoantigen-reactive T cell responses may be critical to increase the therapeutic efficacy of iPSC cancer vaccines.

### Modified iPSCs with neoantigens by AAV enhanced neoantigen-specific T cell response in colorectal and breast cancer models with poor immunogenicity

**Fig. 5:**
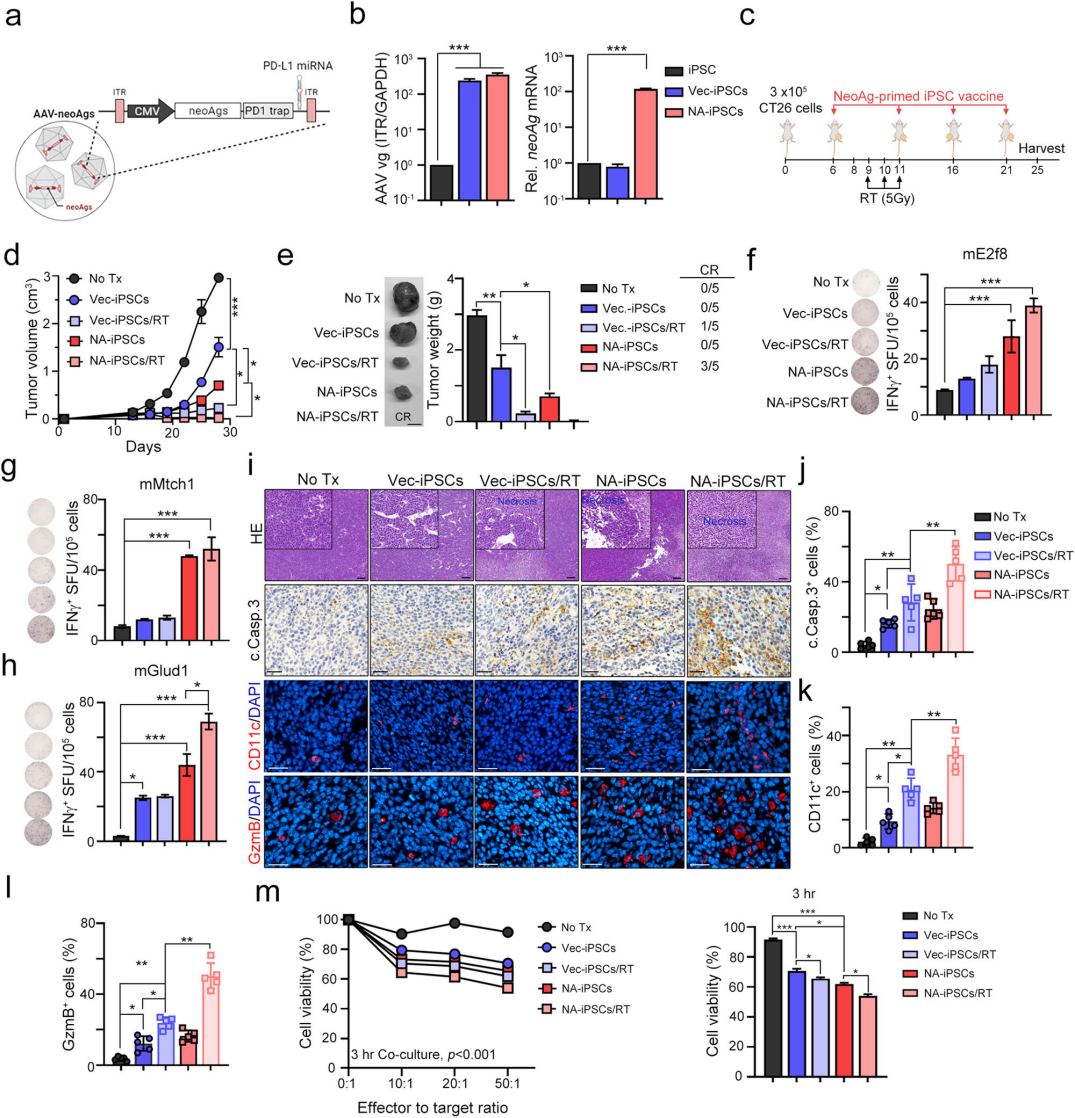
Neoantigen-augmented iPSC (NA-iPSC) vaccination in combination with radiotherapy eradicated tumors in a CRC animal model. **a** Diagram of AAV-neoAgs-CT26 structure. **b** Mouse iPSCs were infected with AAV-Vec and AAV-neoAg (CT26, 1 × 10^8^ vg/mL) for 2 days, and iPSCs were harvested for qPCR and qRT‒PCR (*n* = 3). **c** The treatment scheme of NA-iPSC vaccination and radiotherapy. Mouse CT26 colon cancer cells were subcutaneously inoculated into BALB/c mice (*n* = 5) for 6 days and subcutaneously vaccinated with irradiated (50 Gy) NA-iPSCs (1 × 10^9^ cells) on Days 6, 10, 16 and 21. Local radiotherapy (5 Gy) was given on Days 9, 10 and 11. Tumor volume was measured every 3 days. The animal experiments were repeated twice, independently. **d** Tumor volume was measured every 3 days (*n* = 5). **e** The resected tumors were weighted on Day 25 (*n* = 5). Scale bar = 0.5 cm. Sixty percent of mice showed a complete response to NA-iPSC/RT treatment. **f** The isolated splenocytes (2.5 × 10^5^ cells) were stimulated ex vivo with mE2F8 peptide (1 μg/mL) for 24 h and analyzed by ELISPOT analysis (*n* = 3). **g** The isolated splenocytes (2.5 × 10^5^ cells) were stimulated ex vivo with mMtch1 peptide (1 μg/mL) for 24 h and analyzed by ELISPOT analysis (*n* = 3). **h** The isolated splenocytes (2.5 × 10^5^ cells) were stimulated ex vivo with mGlud1 peptide (1 μg/mL) for 24 h and analyzed by ELISPOT analysis (*n* = 3). **i** The cleaved caspase-3^+^ tumor cells, tumor-infiltrating CD11^+^ DCs and GzmB^+^ T cells were evaluated by immunofluorescence staining. Scale bar = 100 μm (for HE staining) and 30 μm. N: necrosis. **j** The quantification of apoptotic cells (cleaved caspase-3^+^ cells) is shown (*n* = 5). **k** The quantification of tumor-infiltrating CD11^+^ DCs is shown (*n* = 5). **l** The quantification of tumor-infiltrating GzmB^+^ T cells is shown (*n* = 5). **m** The cytotoxic effects of splenic T cells were evaluated by CCK8 (*n* = 3). Significance was calculated with multiple *t* tests analysis and is presented as mean ± s.e.m. *p < 0.05; **p < 0.01 and ***p < 0.001.

Our previous studies have demonstrated the therapeutic efficacy of neoantigen-based cancer vaccines in combination with radiotherapy in poorly immunogenic CT26 (microsatellite-stable CRC, MSS-CRC) and 4T1 (triple-negative breast cancer, TNBC) animal models^22^. Therefore, we first developed a protocol to generate genetically modified neoantigen-augmented iPSC (NA-iPSC) infection by engineered AAV-neoAg, which include 8 neoantigens (CT26), PD1 traps, and PD-L1-targeting microRNAs (Fig. 5a). The immunogenicity of these eight neoantigens derived from mutations in the CT26 cell line has been validated^22^. As shown in Fig. 5b, mouse iPSCs were infected with AAV-Vec (AAV-Vector, hereafter named as Vec-iPSCs) and AAV-neoAg (AAV-neoAgs-CT26, hereafter named as NA-iPSCs) for 2 days, and the levels of ITR (inverted tandem repeat) and *neoAg* mRNA were evaluated by qPCR. We found that the infection efficacy of AAV-Vec and AAV-neoAg was similar, which was evaluated according to the level of ITR (Fig. 5b). However, the level of *neoAg* mRNA was significantly higher in iPSCs after AAV-neoAg infection (>100-fold, *p* < 0.001, Fig. 5b), suggesting that NA-iPSCs (CT26) were successfully established.

Then, we subcutaneously inoculated mouse CT26 colon cancer cells into BALB/c mice for 6 days and subcutaneously vaccinated the mice with irradiated (50 Gy) NA-iPSCs (1 × 10^5^ cells) on Days 6, 10, 16, and 21. Local radiotherapy (5 Gy) was given on Days 9, 10, and 11 (Fig. 5c). As shown in Fig. 5d, e, the tumor volume and tumor weight were dramatically decreased in the NA-iPSC/RT group (*p* < 0.001). Moreover, the complete response rate was superior in NA-iPSCs/RT (3/5 = 60%, the tumor-free period was retained at least 180 days), suggesting that neoantigen-modified iPSCs in combination with radiotherapy markedly increased the therapeutic efficacy. The tumor-free period was retained at least 180 days. After treatment, splenocytes were isolated for neoantigen-specific ELISPOT assay. Splenocytes were seeded into 96-well plates and ex vivo stimulated with individual neopeptides (mE2f8, mMtch1, and mGlud1) for 24 h. Then, the frequency of IFNγ^+^ ELISPOT was evaluated (Fig. 5f–h). We found that after vaccination with iPSCs alone, the neoAg-specific T cell response was extremely low. However, vaccination with NA-iPSCs elicited a high response to neoantigens, especially when combined with local radiotherapy, suggesting that NA-iPSC vaccination successfully triggered a neoAg-specific T cell immune response. the proportion of apoptotic cells were significantly increased (Fig. 5i, j), and the frequency of tumor-infiltrating CD11c^+^ DCs and GzmB^+^ T cells was markedly increased (Fig. 5i, k, l) in the resected tumors. Furthermore, the cytotoxic ability of splenic T cells was superior in the NA-iPSC/RT subgroup compared to the other subgroups (Fig. 5m), indicating that the NA-iPSC cancer vaccine remarkably resulted in systematic antitumor immunity by prompting neoantigen-specific T-cell responses to recognize and eradicate tumor cells in combination with radiotherapy.

**Fig. 6:**
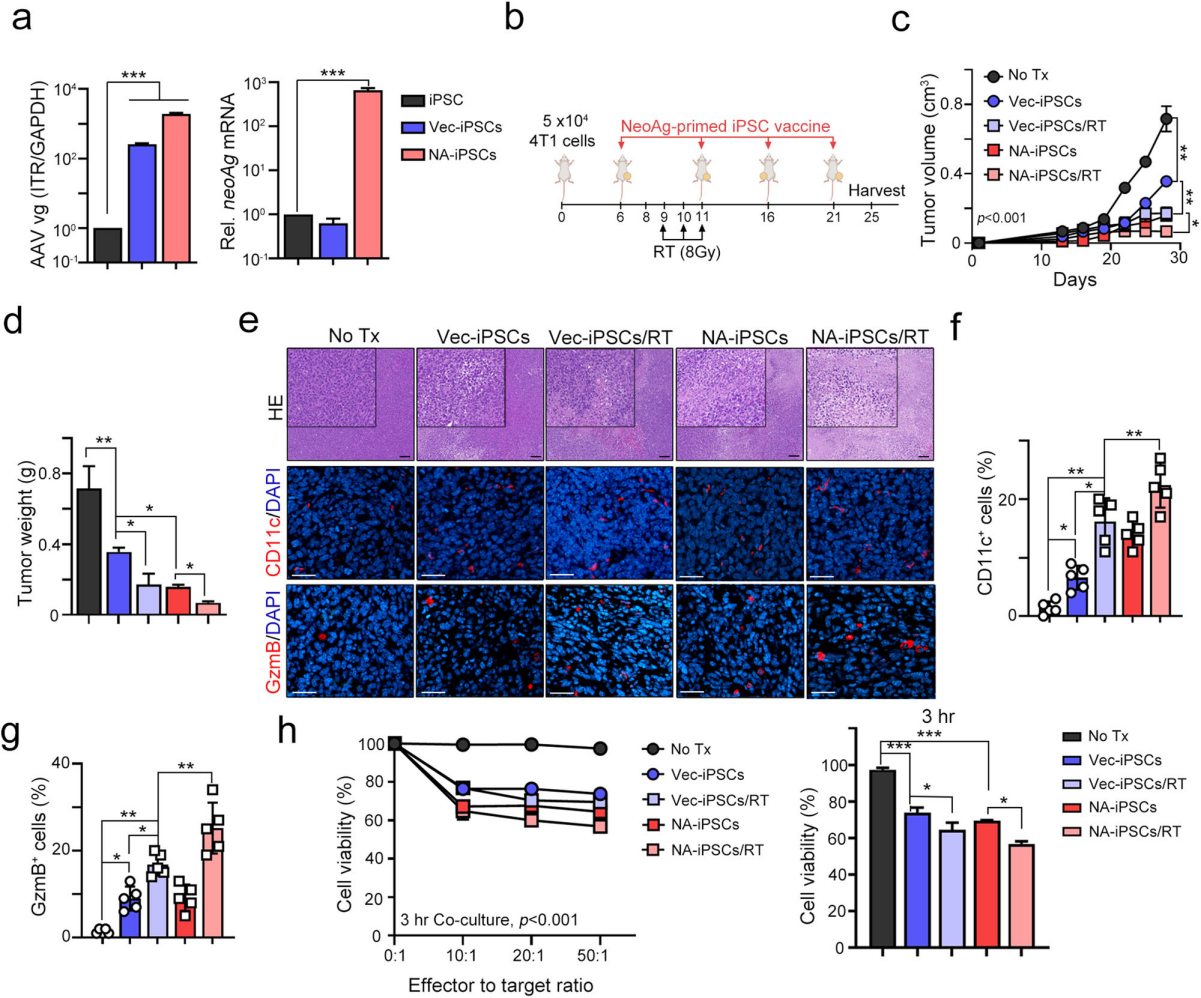
Neoantigen-augmented iPSC (NA-iPSC) vaccination in combination with radiotherapy eradicated tumors in a murine mammary animal model. **a** Mouse iPSCs were infected with AAV-Vec and AAV-neoAgs (AAV-neoAgs-4T1, 1 × 10^8^ vg/mL) for 2 days, and iPSCs were harvested for qPCR and qRT‒PCR (*n* = 3). **b** The treatment scheme of NA-iPSC vaccination and radiotherapy. Mouse CT26 colon cancer cells were subcutaneously inoculated into BALB/c mice (*n* = 5) for 6 days and subcutaneously vaccinated with irradiated (50 Gy) NA-iPSCs (1 × 10^9^ cells) on Days 6, 10, 16, and 21. Local radiotherapy (8 Gy) was given on Days 9, 10, and 11. Tumor volume was calculated every 3 days. The animal experiments were repeated twice, independently. **c** The tumor volume was recorded every 3 days (*n* = 5). **d** The resected tumors were weighted on Day 25 (*n* = 5). **e** The density of tumor-infiltrating CD11^+^ DCs and GzmB^+^ T cells was evaluated by immunofluorescent microscopy. Scale bar = 100 μm (for HE staining) and 30 μm. **f** The quantification of tumor-infiltrating CD11^+^ DCs is shown (*n* = 5). **g** The quantification of tumor-infiltrating GzmB^+^ T cells is shown (*n* = 5). **h** The cytotoxic effects of splenic T cells were evaluated by CCK8 (*n* = 3). Significance was calculated with multiple *t* tests analysis and is presented as mean ± s.e.m. *p < 0.05; **p < 0.01 and ***p < 0.001.

**Fig. 7:**
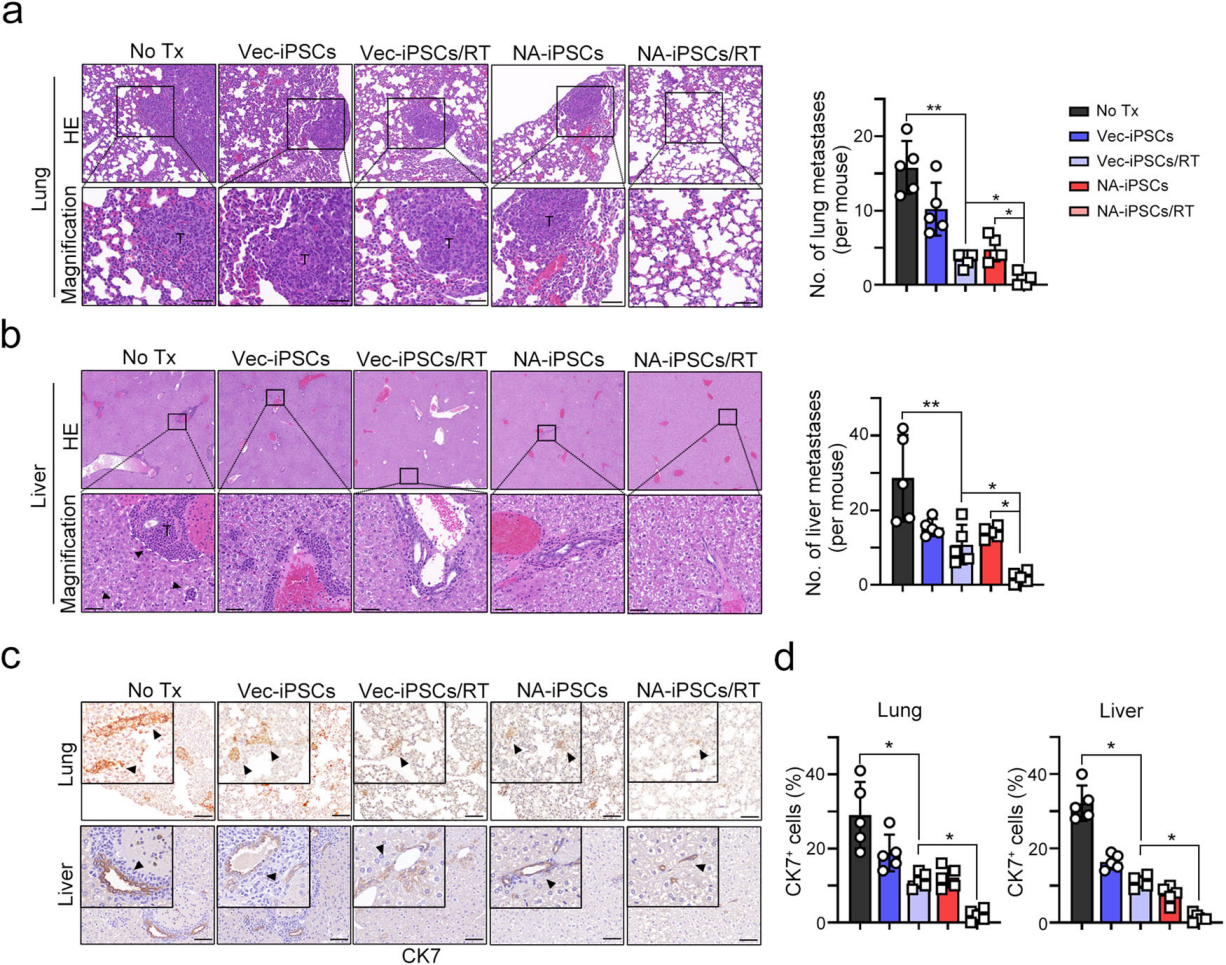
The occurrence of lung and liver metastases was significantly decreased when NA-iPSC vaccination and radiotherapy were administered in a murine mammary animal model. **a** The resected lungs were analyzed by HE staining. The quantification of lung metastatic nodes is shown (*n* = 4–5). Scare bar = 50 μm. **b** The resected livers were analyzed by HE. The quantification of liver metastatic nodes is shown (*n* = 5). Scare bar = 50 μm. **c** Representative images of CK7^+^ breast cancer cells within the resected lung and liver are shown (*n* = 5). Scare bar = 50 μm. **d** The quantification of CK7^+^ breast cancer cells within the lungs and livers is shown (*n* = 5). Significance was calculated with multiple *t* tests analysis and is presented as mean ± s.e.m. *p < 0.05; **p < 0.01 and ***p < 0.001.

To further demonstrate that neoantigen-reactive T-cell responses increased the therapeutic efficacy of NA-iPSCs against poorly immunogenic cancer, we generated NA-iPSCs genetically modified by AAV-neoAgs-4T1 that contained eight mutated TSAs in 4T1 mammary cancer cells^22^. As shown in Fig. 6a, the infection efficacy of AAV-Vec (AAV-Vector, hereafter named as Vec-iPSCs) and AAV-neoAg (AAV-neoAgs-4T1, hereafter named as NA-iPSCs) was similar (Fig. 6b). However, the level of *neoAg* mRNA was significantly higher in iPSCs after AAV-neoAg infection (>100-fold, *p* < 0.001, Fig. 6b). We then vaccinated BALB/c mice bearing 4T1 tumors, which are poorly immunogenic mammary cancer cells, by using the protocol described above. Local radiotherapy (8 Gy) was given on Days 9, 10, and 11. As shown in Fig. 6c, d, the tumor volume and tumor weight were dramatically decreased in the NA-iPSC/RT group (*p* < 0.001), suggesting that NA-iPSCs in combination with radiotherapy also markedly increased the therapeutic efficacy in poorly immunogenic mammary cancer cells. The frequency of tumor-infiltrating CD11c^+^ DCs and GzmB^+^ T cells was markedly increased (Fig. 6e, f, g) in the resected tumors. Furthermore, the cytotoxic ability of splenic T cells was also superior in the NA-iPSC/RT subgroup cocultured with 4T1 cancer cells (Fig. 6h), indicating that neoantigen-specific T-cell responses are critical to enhance the therapeutic efficacy of iPSC cancer vaccines. Furthermore, the occurrence of lung metastases (Fig. 7a) and liver metastases (Fig. 7b) was markedly reduced in the NA-iPSC/RT subgroup. The proportion of CK7^+^ breast cancer cells were significantly decreased in resected lungs and livers after NA-iPSC/RT treatment (Fig. 7c, d). Altogether, these results show that the engineered NA-iPSCs can prompt a neoantigen-specific T cell response, leading to a superior T-cell-mediated immune response and thus enhancing the therapeutic efficacy of RT to inhibit tumor growth and distant metastasis.

## Discussion

In this study, we found that local radiotherapy significantly increased the immunogenicity of iPSC-based cancer vaccines by enhancing STING-mediated type I IFN signaling to increase the infiltration of dendritic cells, cytotoxic CD8^+^ T cells, and NK cells within tumors. The iPSC-based cancer vaccine exerted antitumor effects on poorly immunogenic MSS-CRC when combined with local radiotherapy. Moreover, the engineered NA-iPSC-based cancer vaccine exerted superior therapeutic efficacy by targeting TAAs as well as TSAs (neoantigens) in murine MSS-CRC and TNBC models. The NA-iPSC-based cancer vaccine significantly delayed tumor growth, stimulated antitumor T responses, and inhibited distant metastasis in combination with radiotherapy. These neoantigen-augmented iPSC cancer vaccines remarkably enhanced neoantigen-reactive CD8^+^ T cell responses and promoted the production of antitumor cytokines such as IFNγ and GzmB to eradicate tumor cells and decrease the risk of distant metastasis. Our results demonstrated the effectiveness and antitumor effects of NA-iPSC-based cancer vaccine in combination with RT in poorly immunogenic cancers.

Cancer vaccines based on TAAs were shown to be immunogenic and induced clinical responses in different malignancies such as NY-ESO-1, MAGE-A3, and glypican 3^35,36^. Due to the low expression of TAAs in some normal tissues, immune tolerance toward these antigens occurs by upregulating immunosuppressive mechanisms, such as Foxp3^+^ regulatory T cells (Treg). Treg-mediated immunosuppression may exhaust TAA-reactive T cell response, resulting in the failure of TAA-based cancer vaccines^37^. Administration of irradiated murine and human iPSCs as prophylactic cancer vaccine significantly elicited antitumor effects and inhibited tumor recurrence in multiple malignancies, including lung cancer^7^, melanoma^8^, breast cancer^6^ and pancreatic ductal adenocarcinoma^5^. But iPSCs-based cancer vaccines are insufficient to eradicate existing tumor cells as therapeutic cancer vaccines^2,38^. Here we found that the combination of RT and iPSC-based cancer vaccine significantly increased the frequency of tumor-infiltrating dendritic cells, cytotoxic T, and NK cells. Moreover, the population of CD4^+^CD25^+^Foxp3^+^ Tregs and Gr1^+^CD11b^+^ MDSCs was not increased by RT plus iPSC-based cancer vaccines, indicating that this therapeutic strategy might not induce Treg-mediated suppression of TAA-reactive T cells. Consistent with our results, Ouyang et al. found that an iPSC-based cancer vaccine did not induce CD4^+^CD25^+^Foxp3^+^ Treg infiltration into the tumor-draining lymph node (TDLN) and spleen, reversing the immunosuppressive microenvironment^5^. Additionally, radiation triggered MHC upregulation for driving the broader antigen presentation and activated dsDNA-mediated cGAS/STING-dependent type I IFN production to enhance cancer immunogenicity^39,40^. Owing to the antitumor immunomodulating role of radiotherapy, recent clinical study showed that neoadjuvant radiotherapy and pancreatic adenocarcinoma (PDAC) vaccine (GVAX) significantly sensitized to ICIs in patients with locally advanced PDACs (LAPC), which is one of the poorly immunogenic cancers^41^. These results indicate that the RT can reinvigorate antitumor immunity and exert synergistic effects with autologous whole iPSC cancer vaccine to break immune tolerance, which is a potentially effective antitumor strategy suitable for MSS-CRC patients that were ICIs-unresponsive patients bearing a low mutational burden.

The immunogenicity and antigenicity of tumors were partially contributed from tumor mutational load, which has been considered a response predictor for ICIs^42,43^. Previous studies indicated that high tumor mutational burdens increase the amount of immunogenic neoantigens to promote neoantigen-specific T cells response for immunosurveillance and tumor eradication, enhancing the therapeutic efficacy of ICI^24,25,27,44–46^. However, most malignancies have a relatively lower somatic mutational burden^43^. We here generated autologous neoantigen-augmented iPSC-based cancer vaccines targeting TAAs as well as tumor-specific antigens (TSAs, neoantigens). We found that NA-iPSCs combined with RT achieved superior therapeutic efficacy in these two poorly immunogenic murine models. NA-iPSCs plus RT not only achieved an ~60% complete response but also reduced the risk of lung and liver metastases. Furthermore, neoantigen-reactive T-cell responses were more efficient when NA-iPSCs and RT were administered. Recent elegant study by Lhuillier et al. demonstrated that radiotherapy enhanced neoantigen-bearing gene upregulation for neoantigen-specific CD8^+^ T cell response, suggesting that radiotherapy works in concert with neoantigen vaccination to improve tumor control in preclinical study^47^. Therefore, targeting neoantigens by NA-iPSC cancer vaccines in combination with RT remarkably increases the frequencies of neoantigen-specific CD8^+^ T cells to recognize tumor cells for cell killing, demonstrating impressive therapeutic benefits of NA-iPSC cancer vaccines for RT treatment.

The safety and effectiveness of personalized neoantigen cancer vaccines has been demonstrated in advanced lung cancer, melanoma, and pancreatic cancer^48,49^. But the quantity and quality of neoantigens may greatly determine their clinical benefits^50^. Insufficient tumor tissues from unresectable cancer patients may constrain the accuracy on neoantigen identification, and reduce the therapeutic efficacy of neoantigen-based cancer vaccine. Additionally, loss of tumor antigen by immune escape mechanisms led to poor response to cancer vaccine^51^. Therefore, we believed that combined multiple neoantigens and iPSC (multiple TAAs) cancer vaccines can broadly elicit tumor-specific immune responses with lots of antigen repertoire, mitigating the risk of immune escape by single antigen depletion^50,52^. Furthermore, recent studies showed that iPSC-derived DC and iPSC-derived neoantigen-specific T cell therapy generated systemic antitumor immunity to augment the response to ICIs in combination with radiotherapy^53,54^, suggesting that the feasibility of iPSC-based immunotherapy in cancer treatment. However, our study still has some limitations. First, we only evaluated the autologous iPSCs in two poorly immunogenic murine models with the same HLA haplotype (BALB/c, H-2K^d^). It is necessary to evaluate the therapeutic potential of NA-iPSCs in humanized HLA transgenic mice with different HLA paplotypes. Second, the feasibility of allogenic iPSCs. Although autologous iPSCs may provide a more accurate and representative of personalized neoantigens, allogenic iPSCs are a more attractive tool in “off-the-shelf” iPSC vaccination. Gąbka-Busze et al. indicated that administration of a vaccine composed of allogenic iPSCs admixed with IL16-modified melanoma cells showed superior extension disease-free survival and long-term overall survival in melanoma animal model^8^. Since public shared neoantigens derived from recurrent mutations in cancer driver genes have been identified^55^, the generation of “off-the-shelf” allogenic iPSC cancer vaccine may shorten the waiting time in clinical.

Taken together, our results demonstrate the feasibility and effeteness of iPSC-based cancer vaccine combined with RT triggered systemic antitumor immunity against existing cancers. Furthermore, armoring with neoantigens in iPSCs-based cancer vaccines enhanced the neoantigen-reactive T cell response to eradicate cancer cells in combination with RT, suggesting NA-iPSC-based therapeutic cancer vaccines could be an innovative immunotherapeutic strategy in poorly immunogenic cancers.

## Methods

### Generation and maintenance of murine iPSCs

Murine iPSCs from BALB/c mice were generated as previously described^2^. Briefly, fibroblasts from female BALB/c mice were obtained from a Sendai reprogramming kit (A34546, Thermo Fisher Scientific, CA, USA). After reprogramming, BALB/c mouse iPSCs were grown in 6-well plates with Attachment Factor Protein (1×) AF-based coating solution and incubated in complete 2i medium (50% Neurobasal and 50% DMEM/F12 medium with N2 supplement, B27 supplement, penicillin/streptomycin, 1 μM PD03259010, 3 μM CHIR99021, 2 mM L-glutamine, and 10 ng/ml murine leukemia inhibiting factor). The cells were then expanded, and the medium was changed every day. Murine iPSC was routinely tested for mycoplasma contamination by PCR. Murine iPSC colonies were manually passaged and allowed to grow for 5 passages, followed by sorting for SSEA-1 using magnetic bead sorting (Miltenyi Biotech., CA, USA) to obtain a pure pluripotent population.

### CRC mouse animal model for iPSC vaccination

Adult female BALB/c mice (6 weeks old) were used. Animals were randomly assigned to different treatment subgroups (*n* = 8). All experiments were approved by the China Medical University Institutional Animal Care and Use Committee [Protocol No. CMUIACUC-2018-167]. The murine CRC cell line CT26 were derived from BALB/c mice. The cancer cells were grown in RPMI 1640 and 10% heat-inactivated fetal bovine serum under standard culture conditions^56^. CT26 cell line was routinely tested for mycoplasma contamination. For cancer cell inoculation, 3 × 10^5^ CT26 cells were resuspended in 50% Matrigel in RPMI without serum and subcutaneously injected into the right leg of the mice^57^. Tumor growth was measured every three days. The endpoint was predefined by mouse death or a tumor reaching 2 cm in any direction and endpoint was defined when mice death or tumor volume reached. Mice were sacrificed after exposure to pentobarbital overdose (50 mg/kg).

### iPSC vaccine preparation, immunization and local radiotherapy treatment

For each mouse, 2 × 10^6^ autologous murine iPSCs were sorted by pluripotent marker SSEA-1 and were irradiated (50 Gy). Irradiated iPSCs were suspended in PBS containing 5 mM CpG ODN1826 (Invivogen, CA, USA). Mice were anesthetized with 2% isoflurane (Isothesia, Butler Schein), and subcutaneous immunized with irradiated iPSCs vaccine in the flanks of the mice on indicated days.

For radiotherapy, mice were anesthetized with 300 μL PBS with ketamine (140 mg/kg) and xylazine (3 mg/kg) by intraperitoneal injection before irradiation^58^. The dosimetry data on the irradiation square (8.5 cm × 8.5 cm, depth 5 cm) has been tested and collected to validate the dose of irradiation (293.2 ± 4.4 cGy/300 MU) as previously described^59^. Tumors were received fractionated radiotherapy (6 MV X-ray with 400 MU/min, TrueBeam, Varian) on the indicated days. Following complete anesthesia, the right leg was placed in the square irradiation field (6 cm × 6 cm), and the mouse’s body was kept away from the leg. Radiation was delivered to the irradiation field with the center height of the tumor according to the X-ray beam collimator. The half-beam block was used to protect vital organs, and a 1.5 cm transparent tissue-equivalent bolus was used to cover the irradiated site for an even distribution of irradiation throughout the tumor. The dose was calibrated using a Radcal ion chamber (Monrovia, CA, USA). The length and width (L and W, respectively) of the tumors were measured every 3 days, and the tumor volume (V) was calculated by the formula: V = (L × W^2^)/2. The mice were sacrificed at the termination of the experiments, and the tumor tissues were collected for lysis, immunoblotting analysis, flow cytometry, and immunohistochemistry. The spleens were harvested for flow cytometry. The animal experiments were repeated twice, independently.

### Evaluation of immune cell infiltration in vivo

Tumors and spleens from the mice were isolated and weighed and then placed in petri dishes containing basal RPMI media at room temperature to prevent dehydration, as previously described^60,61^. The tumor and spleen were minced into small pieces (1–2 mm) using a beaver blade, filtered through a 70 μm strainer, spun down, and resuspended in basal RPMI media. Thereafter, the cell suspensions were layered over Ficoll-Paque medium and centrifuged at 1025 × *g* for 20 min. The mononuclear cell layer was transferred into a conical tube, and 20 ml of complete RPMI medium was added and then gently mixed and centrifuged at 650 × *g* for 10 min. Finally, the supernatant was removed, and splenic and tumor-infiltrating lymphocytes (TILs) were resuspended in complete RPMI medium.

TILs were then resuspended in 500 μL of staining buffer (2% BSA and 0.1% NaN_3_ in PBS). The cells were stained with a surface marker panel and intracellular cytokines by different panels: (1) CD8/MDSC panel: PerCP/Cy5 anti-mouse CD8a (1:100, clone 53-6.7, Cat#100732, BioLegend, CA, USA), FITC anti-human/mouse CD11b (1:100, clone M1/70, Cat#101206, BioLegend), PE/Cy7 anti-mouse CD45 (1:100, clone 30-F11, Cat#103114, BioLegend), APC anti-mouse Gr-1 (1:100, clone RB6-8C5, Cat#108412, BioLegend) and PE anti-mouse CD44 (1:100, clone QA19A43, Cat#163610, BioLegend); (2) Foxp^+^ Treg panel: APC anti-mouse CD25 (1:100, clone PC61, Cat#102012, BioLegend), APC/Cy7 anti-mouse CD4 (1:100, clone RM4-5, Cat#100526, BioLegend), PerCP/Cy5 anti-mouse CD8a (1:100, clone 53-6.7, Cat#100732, BioLegend) and PE anti-mouse FOXP3 (1:100, clone QA20A67, Cat#118904, BioLegend); (3) IFNγ^+^CD8^+^ T cell panel: FITC anti-mouse IFNγ (1:100, clone XMG1.2, Cat#505806, BioLegend), PerCP/Cy5 anti-mouse CD8a (1:100, clone 53-6.7, Cat#100732, BioLegend) and PE/Cy7 anti-mouse CD45 (1:100, clone 30-F11, Cat#103114, BioLegend), and their isotypes (BioLegend). The intracellular marker Foxp3 was fixed and permeabilized by a FOXP3 Fix/Perm buffer set before staining (Cat#421403, Biolegend). The intracellular marker INFγ was activated by the protein transport inhibitor brefeldin A for 3 h before staining. These samples were analyzed using a Guava® easyCyte™ Flow Cytometer (Luminex, CA, USA) and FlowJo v10.0.7 software (Ashland, USA).

### Histopathology and immunohistochemistry of tumors

The tumors were explanted from mice and processed for histopathology at the time of sacrifice. Briefly, the organs were fixed in 4% paraformaldehyde for 72 h and transferred to 70% ethanol. Fixed samples were embedded in paraffin, and sections were cut and stained with hematoxylin and eosin (H&E) for histological analysis by a pathologist.

The antibodies used in this study were as follows: anti-mouse CD11c (1:300, ab219799, Abcam, Cambridge, UK), anti-mouse CD8a (1:300, ab217344, Abcam), anti-mouse GzmB (1:300, ab255598, Abcam), anti-NKG2D antibody (1:300, ab203353) and Ki67 (1:300, ab15580, Abcam). Tissue sections (3 µm thickness) were stained with the HRP-conjugated avidin–biotin complex (ABC) from the Vectastain Elite ABC Kit (Vector Laboratories, Burlingame, CA, USA) and DAB chromogen (Vector Laboratories) and counterstained with hematoxylin. Staining for CD8, GzmB, and NKG2D was positive in tumor-infiltrating lymphocytes (TILs) and was evaluated using a microscope (OLYMPUS BX53, Tokyo, Japan). To evaluate the infiltrating frequency of TILs, the central region of the tumor tissue was reviewed at 40× magnification, and the number of TILs within the tumor bed was counted. The average number of TILs in five high-power fields was included to examine the number of immune cells per square millimeter (No. of TILs/mm^2^)^61,62^.

### Western blot analysis

Total lysates (30 μg) were resolved in an SDS‒PAGE gel and transferred onto PVDF membranes (Millipore, MA, USA)^63,64^ for immunoblotting analysis with the indicated antibodies overnight at 4 °C and probed with HRP-conjugated secondary antibodies for 2 h at room temperature. All antibodies were prepared with T-Pro Protein Free Blocking Buffer (BioLion Tech., Taipei, Taiwan). The blot membrane was then incubated with Immobilon Western Chemiluminescent HRP Substrate (Millipore, CA, USA), visualized by an ImageQuant™ LAS 4000 biomolecular imager (GE Healthcare, Amersham, UK), processed using Adobe Photoshop and quantified using ImageJ software (NIH, MD, USA). Each blot was stripped by immunoblotting stripping buffer (BioLion Tech.) before incubation with the other antibodies^64^. Raw uncropped blots were shown in Supplementary Figs. 1 and 2.

The following antibodies were used: Glud1 (1:1000, A7631, ABclonal, MA, USA), Mtch1 (1:1000, A8063, ABclonal), E2F8 (1:1000, A1135, ABclonal), and cleaved caspase-3 (1:1000, #9661, Cell Signaling and IR96-401, iReal Biotech., Taipei, Taiwan), p-Akt^S473^ (1:1000, #9271, Cell Signaling, CA, USA) and GAPDH (1:3000, IR3-8, iReal Biotech., Taipei, Taiwan).

### qRT‒PCR

Total RNA was extracted from the cell lines and resected tumor tissues with TRIzol (Thermo Fisher Scientific, CA, USA), quantified by measuring the absorbance at 260 nm, and then reverse-transcribed into cDNA by iScript™ Reverse Transcription Supermix (Bio-Rad, CA, USA) as previously described^58^. Primers were designed by the Primer design tool (NCBI, USA) according to sequence information from the NCBI database (Supplementary Table 1). qRT‒PCR was performed in a final reaction volume of 20 μL with iQ™ SYBR® Green Supermix (Bio-Rad, CA, USA) by StepOnePlus Real Time PCR (Thermo Fisher Scientific, CA, USA). All reactions were performed in triplicate for each sample, and GAPDH was employed as a reference gene for normalization. The relative values of gene expression were calculated using the 2^−ΔΔCt^ method. The comparisons of gene expression levels were performed using the *t* test.

### RNA sequencing data analysis

For RNA-seq data, quality was examined by analyzing per base sequence quality plots using FastQC. The trimming of sequence reads was performed by Trimmomatic (v0.39). RNA-seq reads were aligned to the mouse genome (mm10) using HISAT2 (v2.1.0) software. Reads that overlapped with exon coordinates were counted using featureCounts (v2.0.3). The read count matrix was normalized by converting the counts to counts per million (CPM). Subsequently, a statistical analysis was performed using edgeR (v4.2.2) to identify genes that were significantly expressed.

### AAV vectors generation and recombinant AAV virus purification

All AAV viral particles were produced as previously described^22^. Briefly, codon-optimized sequences cloned into a CMV-driven AAV2-CMV expression vector and cotransfected with the helper plasmids pRC2-miR342 and pHelper (AAVpro Helper Free System, #6230, Takara Bio., CA, USA) using 293 T cells by the triple transfection method^22^. The virus particles were produced: AAV-Vec (AAV-Vector), AAV-neoAgs-CT26 (the same sequence of meAAV-TSA-CT26 in our previous work, Supplementary Table 2) and AAV-neoAgs-4T1 (the same sequence of meAAV-TSA-4T1 in our previous work, Supplementary Table 2)^22^. Seventy-two hours after transfection, the cells were collected by centrifugation, and recombinant AAV2 vectors were produced and purified using an AAVpro purification kit (#6232, Takara Bio., CA, USA). AAV2 titration was performed by qPCR on the vector genomes by AAV real-time PCR titration kit (#6233, Takara Bio., CA, USA) according to manufacture’ manual.

### Vaccination with neoantigen-augmented iPSC (NA-iPSC) in CRC and TNBC animal model

3 × 10^5^ CT26 cells or 5 × 10^4^ 4T1 cells were resuspended in 50% Matrigel in RPMI without serum and subcutaneously injected into the right leg of BALB/c mice (*n* = 5)^57^. Local radiotherapy and neoantigen-augmented iPSCs (NA-iPSCs) were vaccinated on indicated days. Briefly, 1 × 10^5^ murine iPSCs (derived from BALB/c mice) were infected with AAV-Vec and AAV-neoAg (1 × 10^8^ vg/mL) that for 2 days^22^. Then, AAV-transduced iPSCs were irradiated (50 Gy) and suspended in 100 μL PBS for subcutaneous vaccination. The tumor volume (V) was calculated by the formula: V = (L × W^2^)/2. The mice were sacrificed at the termination of the experiments, and the tumor tissues were collected for immunohistochemistry. The animal experiments were repeated twice, independently.

### Ex vivo immune analysis

IFNγ ELISPOT assays were performed on spleen single-cell suspensions according to the manufacturer’s instructions^22^. Mouse splenocytes were plated in duplicate and stimulated with individual 25-mer peptides (mE2f8, mMtch1, and mGlud1) at a final concentration of 1 μg/ml overnight. The peptide diluents dimethyl sulfoxide (Sigma‒Aldrich, MO, USA) and concanavalin A (Sigma‒Aldrich, MO, USA) were used as negative and positive controls, respectively. Plates were developed by subsequent incubation with biotinylated anti-mouse IFNγ antibody, conjugated streptavidin–alkaline phosphatase, and finally with 5-bromo-4-chloro-3-indoyl-phosphate/nitro blue tetrazolium 1-Step solution (Thermo Fisher Scientific, CA, USA). An automated enzyme-linked immunosorbent assay video analysis system automated plate reader was used to analyze the plates (ImmunoSPOT, OH, USA). ELISpot data were expressed as IFN-γ SFCs per million splenocytes. ELISpot responses were considered positive if all the following conditions occurred: (i) IFNγ production in ConA-stimulated wells, (ii) the number of spots seen in positive wells was three times the number detected in the mock control wells (dimethyl sulfoxide), and (iii) at least 30 specific spots/million splenocytes.

### Cell viability of splenic T cells and tumor cells

For cell viability analysis, CT26 (5 × 10^3^ cells) or 4T1 cells (5 × 10^3^ cells) were cocultured with isolated splenic T cells (1 × 10^4^ cells) from vaccinated mice for 3 h on indicated effector to target ratio. Tumor cell specific killing ability was evaluated by CCK analysis.

### Quantification and statistical analyses

All of the data are expressed as the mean ± SEM. Intergroup differences were appropriately assessed by either unpaired two-tailed Student’s *t* test or one-way analysis of variance (ANOVA) with multiple comparison tests using PRISM 9 GraphPad software. ∗*p* < 0.05, ∗∗*p* < 0.01, ∗∗∗*p* < 0.001.

### Reporting summary

Further information on research design is available in the Nature Research Reporting Summary linked to this article.

### Supplementary information

Supplementary information

REPORTING SUMMARY

## Data Availability

All RNA-seq are deposited in a publicly accessible NCBI database GSE262852 (https://www.ncbi.nlm.nih.gov/geo/query/acc.cgi?acc=GSE262852). The other datasets used and/or analyzed during the current study are available from the corresponding author on reasonable request.
