# Supplementary material for: Neoantigen-augmented iPSC cancer vaccine combined with radiotherapy promotes antitumor immunity in poorly immunogenic cancers
Source: NPJ Vaccines. 2024 May 31;9:95. doi: 10.1038/s41541-024-00881-5 (PMC11143272; doi:10.1038/s41541-024-00881-5)
Supplement: Supplementary file 1 — Supplementary information [file 41541_2024_881_MOESM1_ESM.pdf]

Supplementary information

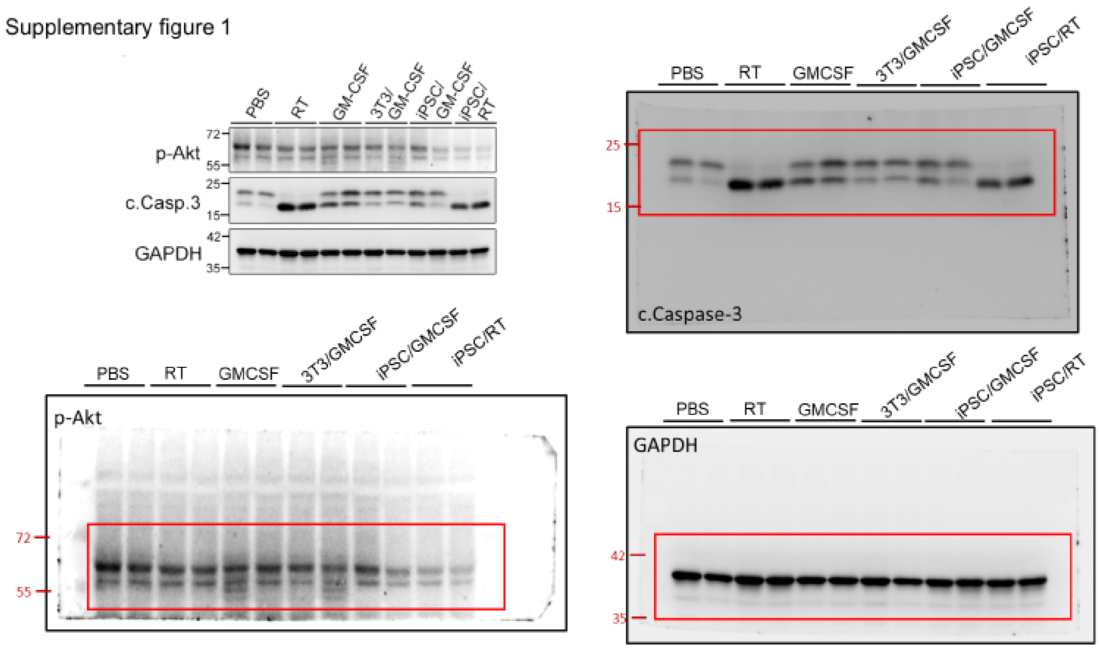

Supplementary Figure 1. The raw uncropped blot images in Fig. 1g.

Supplementary figure 2

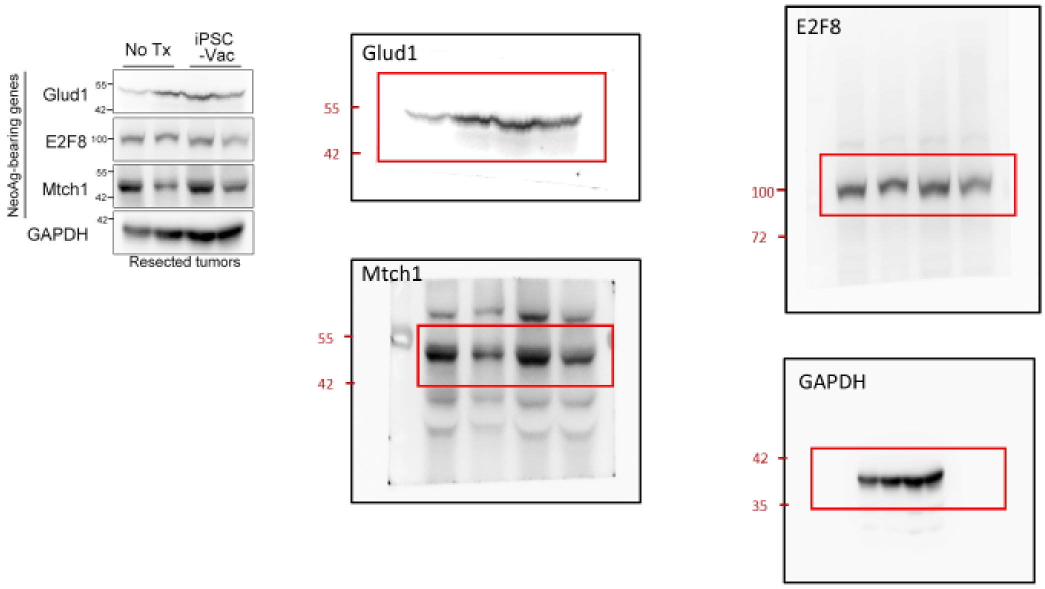

Supplementary Figure 2. The raw uncropped blot images in Fig. 4e.

Supplementary Table 1. Primer sequences were used.

Gene	Primer sequence (5' to 3')
Ifng1	TGGGCGATCAAGGAAAACGA<br>TTGATGCTTTAGCCTGCCGT
Gzmb	GAAGCCAGGAGATGTGTGCT<br>GCACGTTTGGTCTTTGGGTC
Tmem173 (sting1)	TGGCTGCTGATGCCATACTC<br>TAGTCCAAGTTCGTGCGAGG
Ifna2	CCTCCTCTGACCCAGGAAGA<br>AAGACAGGGCTCTCCAGACT
Ifnb1	CCTGGAGCAGCTGAATGGAA<br>CCACCCAGTGCTGGAGAAAT
Glud1	ATCATCAAGCCCTGCAACCA<br>AACGGATACCTCCCTTGCAG
E2f8	CCTGGAGTAGAATTCCGGGC<br>CAGGCTCACTATCTGAGGCG
Mtch1	AGGCCAAGTACAGTGGTGTG<br>ACAGCCCCACAAGAAAACCA
Gapdh	GGAGAGTGTTTCCTCGTCCC<br>ATGAAGGGGTCGTTGATGGC
CT26-neoAg	AGTGGGCTTTGGAAGGATCG<br>TCGATAGCGTTCACGTAGGC
4T1-neoAg	GATCGGCGCCATTAGGTTCT<br>TCTGCATGCTTGTCAGGTCC
ITR	GGAACCCCTAGTGATGGAGTT<br>CGGCCTCAGTGAGCGA

Supplementary Table 2. Neoantigens in mouse colon carcinoma CT26 and breast cancer 4T1 cell line.

ID	Neopeptide sequence	Gene origin	Mutation	DNA sequence
**CT26**				
CT1	ILPQAPSGPSYA[I/T]YLQPAQAQMLTP	E2f8	Single base mutation	ATTCTGCCCCAAGCTCCTAGCGGACCTAGCTACGCCACCTATCTCCAGCCCGCCCAAGCCCAGATGCTGACTCCT
CT2	KPLRRNNSYTSY[T/I]MAICGMPLDSF	Slc20a1	Single base mutation	AAGCCTCTGAGGAGGAACAACTCCTACACCAGCTACATCATGGCCATTTGCGGAATGCCTCTGGATTCCTTCAGA
CT3	PGPQNFPPQNMF[G/E]FPPHLSPPLLPP	Phf3	Single base mutation	CCCGGACCCCAGAACTTCCCTCCCCAGAACATGTTCGAGTTCCCTCCTCATCTGTCCCCCCCTCTGCTGCCTCCT
CT4	VIQTSKYYMRDV[T/I]AIESAWLLELAP	Dhx35	Single base mutation	GTGATCCAGACCAGCAAGTACTACATGAGGGACGTGATCGCCATTGAGAGCGCTTGGCTGCTGGAGCTGGCTCCT
CT5	SWIHCWKYLSVQ[G/S]SQLFRGSSLLFR	Mtch1	Single base mutation	AGCTGGATCCATTGCTGGAAGTACCTCAGCGTCCAGTCCAGCCAGCTGTTTAGAGGCAGCAGCCTCCTCTTTAGG
CT6	LLPFYPPDEALE[T/I]GLELNSSALPPT	Slc4a3	Single base mutation	CTGCTGCCCTTCTACCCTCCCGACGAGGCTCTGGAGATCGGACTGGAGCTGAATAGCAGCGCTCTCCCCCCCACT
CT7	HIHRAGGLFVAD[E/A]IQVGFGRIGKHF	Agxt2l2	Single base mutation	CACATTCATAGGGCCGGAGGACTGTTTGTGGCCGATGCCATCCAAGTGGGCTTTGGAAGGATCGGCAAGCACTTC
CT8	LRTAAYVNAIEK[V/I]FKVYNEAGVTFT	Glud1	Single base mutation	CTGAGGACCGCCGCCTACGTGAACGCTATCGAGAAGATCTTCAAGGTCTACAACGAGGCCGGCGTGACTTTCACT
**4T1**				
4T-1	TPRGKIQAKKWS[R/L]VPFSIPVFDILQ	Dhx58	Single base mutation	ACCCCTAGGGGCAAGATCCAAGCCAAGAAGTGGAGCCTGGTGCCTTTCAGCATCCCTGTGTTCGACATCCTGCAA
4T-2	KADVFHAYLSLL[K/T]QTRPVQSWLCDP	Cand1	Single base mutation	AAGGCAGACGTGTTCCACGCCTACCTGAGCCTGCTGACACAGACAAGGCCTGTGCAGAGCTGGCTGTGCGACCCT
4T-3	NDEPDLDPVQEL[T/L]YDLRSQCDAIRV	Wdr11	Single base mutation	AATGACGAGCCTGACCTGGACCCTGTGCAAGAGCTGATCTACGATCTGAGGTCTCAGTGCGACGCCATTAGGGTG
4T-4	VKEEDSLHWQRP[G/E]DVQKVKALSFYQ	Pzp	Single base mutation	GTTAAAGAGGAGGACAGCCTGCACTGGCAGAGGCCCGAGGACGTGCAGAAGGTGAAGGCCCTGAGCTTCTATCAG
4T-5	LREEASEILEEM[S/R]HKLRIGAIRFFA	Gnpat	Single base mutation	TTGCGCGAGGAGGCAAGCGAGATCCTGGAGGAGATGAGGCACAAGCTGAGGATCGGCGCCATTAGGTTCTTCGCT
4T-6	AAALQMIIAYAY[T/R]GNLAVNDSTVEQ	Kbtbd2	Single base mutation	GCTGCCGCCCTGCAGATGATCATCGCCTACGCCTATAGGGGCAACCTGGCCGTGAACGACAGCACCGTGGAGCAA
4T-7	DYTAAGFSSFQK[I/L]RLDLTSMQIITT	Adamts9	Single base mutation	GACTATACCGCCGCCGGCTTCAGCAGCTTTCAGAAGCTGAGGCTGGACCTGACAAGCATGCAGATCATCACCACA
4T-8	ILDLLLLYKKHK[G/A]KKMTVPVRRHAY	Chsy1	Single base mutation	ATCCTGGACCTGCTGCTGCTGTACAAGAAGCACAAGGCCAAGAAGATGACCGTGCCTGTGAGGAGGCACGCCTAC

Supplementary Table 3. The potential neoantigens in CT26 resected tumors

ID	H-2-Dd	H-2-Kd	H-2-Ld	Gene	AA Change	Num Passing Transcripts	Best Peptide	Pos	Num Passing Peptides	IC50 MT	IC50 WT	%ile MT	%ile WT	RNA Expr	RNA VAF	Allele Expr	RNA Depth	DNA VAF	Tier	Evaluation	CHROM	START	END	POS	REF_y	ALT_y	biotype
9-19477584-19477585-C-T		5	1	Olfr850	V222I	1	SYIHIISSI	3	6	5.085	13.034	0.1	0.1	0	0	0	0	0.2	NoExpr	Pending	9	19477584	19477585	19477585	C	T	missense_variant
4-140827908-140827910-CA-TG		4		Padi1	YV306-307YM	1	LYMCSVTDI	3	4	16.01	36.37	0.1	0.2	0.523	0	0	0	0.204	NoExpr	Pending	4	140827908	140827910	140827909	CA	TG	missense_variant
11-58758048-58758049-G-A		2	1	Olfr316	R128Q	1	RYIAICQPL	7	3	17.1	22.84	0.1	0.2	0	0	0	0	0.019	NoExpr	Pending	11	58758048	58758049	58758049	G	A	missense_variant
11-71218063-71218064-G-A		1	1	Nlrp1b	H204Y	1	YPKELLFIL	1	2	18.88	20.464	0.2	0.2	2.359	0	0	0	0.25	NoExpr	Pending	11	71218063	71218064	71218064	G	A	missense_variant
3-109934595-109934596-G-A		3	1	Ntng1	A287V	1	RYFYVISDI	5	4	19.102	30.067	0.1	0.1	0	0	0	0	0.026	NoExpr	Pending	3	109934595	109934596	109934596	G	A	missense_variant
11-87889593-87889594-C-T		1	3	Olfr462	V101I	1	GCMAQIFFF	6	4	20.027	24.333	0.2	0.2	0	0	0	0	0.026	NoExpr	Pending	11	87889593	87889594	87889594	C	T	missense_variant
6-141943448-141943449-G-C		4		Slco1a1	Q46E	1	VYMNSMLTEI	9	4	20.57	20.78	0.2	0.2	0	0	0	0	0.234	NoExpr	Pending	6	141943448	141943449	141943449	G	C	missense_variant
2-79333634-79333635-G-A		1	1	Cerkl	T447I	1	FPFVEIYII	6	2	21.429	32.584	0.2	0.2	0.225	0	0	0	0.374	NoExpr	Pending	2	79333634	79333635	79333635	G	A	missense_variant
15-57259135-57259136-T-A		5		Slc22a22	Q148H	1	KYYAHATSL	5	5	21.941	8.89	0.2	0.1	0	0	0	0	0.182	NoExpr	Pending	15	57259135	57259136	57259136	T	A	missense_variant
3-14770865-14770866-C-T		1		Car1	S131N	1	KYSNASEAI	4	1	22.712	16.915	0.2	0.1	0	0	0	0	0.556	NoExpr	Pending	3	14770865	14770866	14770866	C	T	missense_variant
11-40710196-40710197-C-G		1		Hmmr	A449P	1	KYNDTPQSL	6	1	22.922	17.992	0.2	0.2	42.361	0	0	0	0.023	NoExpr	Pending	11	40710196	40710197	40710197	C	G	missense_variant
14-50736547-50736548-C-A		1	3	Olfr749	V205F	1	YAQSSFFLF	7	3	23.442	870.623	0.2	2.6	0	0	0	0	0.057	NoExpr	Pending	14	50736547	50736548	50736548	C	A	missense_variant
19-13410933-13410934-C-T		1		Olfr1469	R122C	1	CYAAVCKPL	1	1	24.071	38.53	0.2	0.2	0	0	0	0	0.325	NoExpr	Pending	19	13410933	13410934	13410934	C	T	missense_variant
7-7241578-7241579-C-T		2		Vmn2r29	C432Y	1	HYLKVHSFL	2	2	24.271	519.111	0.1	1.6	0.062	0	0	0	0.051	NoExpr	Pending	7	7241578	7241579	7241579	C	T	missense_variant
3-135524582-135524583-C-T		1	2	Manba	L306F	1	YNMTIFFAL	6	3	26.533	40.027	0.2	0.2	7.112	0	0	0	0.035	NoExpr	Pending	3	135524582	135524583	135524583	C	T	missense_variant
17-37299756-37299757-T-A		6	1	Olfr101	N222Y	1	SYFYIITYL	8	7	29.18	38.823	0.2	0.2	0	0	0	0	0.028	NoExpr	Pending	17	37299756	37299757	37299757	T	A	missense_variant
4-156331348-156331350-AG-TA		3	1	Vmn2r-ps159	PV56-57PI	1	FYLGAADTPI	10	4	30.19	81.403	0.1	0.1	0	0	0	0	0.069	NoExpr	Pending	4	156331348	156331350	156331349	AG	TA	missense_variant
17-36032407-36032408-G-A	1	3	3	H2-T23	R26W	1	SPHSLWYFT	6	7	30.761	30.549	0.2	0.2	76.781	0	0	0	0.138	NoExpr	Pending	17	36032407	36032408	36032408	G	A,T	missense_variant
17-38209033-38209034-C-T		4		Olfr135	P263L	1	MYLQPLSSI	6	4	31.48	36.96	0.1	0.1	0	0	0	0	0.027	NoExpr	Pending	17	38209033	38209034	38209034	C	T	missense_variant
19-33749463-33749464-G-A		1		Lipo2	P58S	1	GYILSINRI	5	1	31.496	99.83	0.1	0.23	0.029	0	0	0	0.47	NoExpr	Pending	19	33749463	33749464	33749464	G	A	missense_variant
16-58823835-58823836-C-T		1	2	Olfr175	S291N	1	IPLLNPFIYNL	10	3	31.74	95.29	0.03	0.05	0	0	0	0	0.268	NoExpr	Pending	16	58823835	58823836	58823836	C	T	missense_variant
9-19360965-19360966-G-C		1	1	Olfr846	L130V	1	RYAAICHPV	9	2	32.589	18.96	0.2	0.2	0	0	0	0	0.253	NoExpr	Pending	9	19360965	19360966	19360966	G	C	missense_variant
2-87357847-87357848-C-A		1	1	Olfr1120	P135T	1	RYVAICNTL	8	2	34.03	38.331	0.2	0.3	0	0	0	0	0.055	NoExpr	Pending	2	87357847	87357848	87357848	C	A	missense_variant
11-120956420-120956421-G-A		1		Slc16a3	R145H	1	RYFNKRHPI	7	1	35.11	82.08	0.2	0.2	15.504	0	0	0	0.078	NoExpr	Pending	11	120956420	120956421	120956421	G	A	missense_variant
11-71114434-71114435-G-T		5		Nlrp1a	H651Q	1	YYAVQSLCTTL	5	5	35.76	92.081	0.2	0.2	0.059	0	0	0	0.054	NoExpr	Pending	11	71114434	71114435	71114435	G	T	missense_variant
2-87071285-87071286-G-A		2		Olfr1107	P283S	1	FYTLVISML	7	2	35.817	280.711	0.2	0.66	0	0	0	0	0.403	NoExpr	Pending	2	87071285	87071286	87071286	G	A	missense_variant
17-37299642-37299644-GG-TA		2	1	Olfr101	IR259-260IS	1	TYISPASGSSL	4	3	36.67	39.46	0.1	0.1	0	0	0	0	0.029	NoExpr	Pending	17	37299642	37299644	37299643	GG	TA	missense_variant
14-52349728-52349729-G-A		5	2	Olfr1513	H106Y	1	FYYFLGSTV	3	7	36.83	22.71	0.2	0.2	0	0	0	0	0.084	NoExpr	Pending	14	52349728	52349729	52349729	G	A	missense_variant
9-39461642-39461643-T-G		6		Olfr954	F71V	1	YFLSSLSVI	8	6	37.09	40.559	0.2	0.2	0	0	0	0	0.275	NoExpr	Pending	9	39461642	39461643	39461643	T	G	missense_variant
7-43497859-43497860-A-G		2		4931406B18Rik	V316A	1	AYGAIASAL	1	2	37.71	57.84	0.2	0.2	0	0	0	0	0.362	NoExpr	Pending	7	43497859	43497860	43497860	A	G	missense_variant
10-129767874-129767875-A-C		1		Olfr808	K126N	1	RYVAICNPL	7	1	38.331	96.154	0.3	0.5	0	0	0	0	0.261	NoExpr	Pending	10	129767874	129767875	129767875	A	C	missense_variant
17-37299636-37299638-CA-TG		2		Olfr101	PA261-262PT	1	TYIRPTSGSSL	6	2	38.55	39.46	0.1	0.1	0	0	0	0	0.029	NoExpr	Pending	17	37299636	37299638	37299637	CA	TG	missense_variant
7-86811577-86811579-TG-CA		3	1	Vmn2r77	M704T	1	NYIIPICTTI	9	4	38.57	142.57	0.2	0.2	0	0	0	0	0.054	NoExpr	Pending	7	86811577	86811579	86811578	TG	CA	missense_variant
13-13393294-13393295-G-C		2	2	Gpr137b	L67V	1	SYQSVFVFL	7	4	38.91	37.919	0.2	0.2	19.537	0	0	0	0.051	NoExpr	Pending	13	13393294	13393295	13393295	G	C	missense_variant
7-5481284-5481285-T-A		1	2	Vmn2r28	T639S	1	FCFLCSFFF	6	2	39.264	40.087	0.2	0.2	0	0	0	0	0.032	NoExpr	Pending	7	5481284	5481285	5481285	T	A	missense_variant
10-36993649-36993650-C-T		4	1	Hdac2	P228S	1	KYYAVNFSM	8	4	39.48	44.12	0.2	0.2	75.457	0	0	0	0.569	NoExpr	Pending	10	36993649	36993650	36993650	C	T	missense_variant
9-38402967-38402968-T-G		1	5	Olfr147	F31L	1	LPLLYLFLI	4	6	40.163	27.595	0.2	0.2	0	0	0	0	0.284	NoExpr	Pending	9	38402967	38402968	38402968	T	G	missense_variant
4-49380300-49380301-G-A		2		Acnat2	P359L	1	AYLGAGHLI	3	2	41.313	298.6	0.2	0.7	0	0	0	0	0.464	NoExpr	Pending	4	49380300	49380301	49380301	G	A	missense_variant
9-39217775-39217776-A-G		5		Olfr944	I140V	1	SYQVYNSLI	4	5	41.347	53.03	0.2	0.2	0	0	0	0	0.288	NoExpr	Pending	9	39217775	39217776	39217776	A	G	missense_variant
16-96593076-96593077-T-G		2	2	Dscam	K2008N	1	NPYANSYTL	5	4	41.384	51.642	0.2	0.2	0.081	0	0	0	0.146	NoExpr	Pending	16	96593076	96593077	96593077	T	G	missense_variant
6-58955280-58955281-T-A		1		Fam13a	K369M	1	KYRPSHSDM	9	1	41.696	143.579	0.2	0.6	0.276	0	0	0	0.239	NoExpr	Pending	6	58955280	58955281	58955281	T	A	missense_variant
2-63980415-63980416-C-A		1		Fign	S170I	1	SYSSSTCGI	9	1	42.252	688.934	0.2	1.8	0	0	0	0	0.18	NoExpr	Pending	2	63980415	63980416	63980416	C	A	missense_variant
8-43569341-43569342-T-G		2		Adam26a	E370D	1	SYEDMYSVV	4	2	43.46	38.394	0.2	0.2	0	0	0	0	0.202	NoExpr	Pending	8	43569341	43569342	43569342	T	G	missense_variant
17-33999978-33999980-AC-GT		3	1	H2-K1	V30T	1	GPHSLRYFT	9	4	44.361	56.364	0.2	0.3	899.72	0	0	0	0.132	NoExpr	Pending	17	33999978	33999980	33999979	AC	GT	missense_variant
4-156334451-156334452-C-T		4		Vmn2r-ps159	P343L	1	KYLVDISHTI	3	4	44.64	182.056	0.2	0.4	0	0	0	0	0.065	NoExpr	Pending	4	156334451	156334452	156334452	C	T	missense_variant
4-148472044-148472045-G-A		3	2	Mtor	V971M	1	MVQAITFIF	1	5	45.576	85.364	0.2	0.5	20.742	0	0	0	0.243	NoExpr	Pending	4	148472044	148472045	148472045	G	A	missense_variant
9-38047584-38047585-G-A		2		Olfr884	R121K	1	KYAAICKPL	1	2	45.576	47.592	0.3	0.3	0	0	0	0	0.434	NoExpr	Pending	9	38047584	38047585	38047585	G	A	missense_variant
17-37589975-37589976-C-T		1	1	Olfr114	V126I	1	RYIAICLPL	5	2	45.61	37.005	0.2	0.2	0	0	0	0	0.42	NoExpr	Pending	17	37589975	37589976	37589976	C	T	missense_variant
5-18267182-18267183-C-T		4		Gnai1	A301T	1	TYEEAATYI	7	4	46.73	74.97	0.2	0.4	0.057	0	0	0	0.344	NoExpr	Pending	5	18267182	18267183	18267183	C	T	missense_variant
2-88794482-88794483-T-A		1	1	Olfr1201	L34M	1	FIIFLVFYM	9	2	48.865	68.207	0.2	0.3	0	0	0	0	0.042	NoExpr	Pending	2	88794482	88794483	88794483	T	A	missense_variant
7-107097554-107097555-T-A		1	2	Olfr17	L30H	1	EVQALLFHL	8	3	49.162	58.614	0.3	0.3	0	0	0	0	0.223	NoExpr	Pending	7	107097554	107097555	107097555	T	A	missense_variant
9-40100652-40100653-G-A		1	2	Olfr984	T279I	1	IPMLNPAIYTL	1	3	49.49	235.45	0.03	0.13	0	0	0	0	0.488	NoExpr	Pending	9	40100652	40100653	40100653	G	A	missense_variant
2-158851763-158851764-C-T		4		Dhx35	T646I	1	YYMRDVIAI	7	4	50.033	9.289	0.2	0.1	11.253	0	0	0	0.347	NoExpr	Pending	2	158851763	158851764	158851764	C	T	missense_variant
4-62499759-62499760-A-T		1	1	Hdhd3	F60I	1	AYRAQSHNI	9	2	51.297	1130.03	0.2	2.6	3.841	0	0	0	0.183	NoExpr	Pending	4	62499759	62499760	62499760	A	T	missense_variant
11-58566975-58566976-G-A		1		Olfr224	A123V	1	RYVVICHPL	4	1	51.54	37.919	0.3	0.2	0	0	0	0	0.059	NoExpr	Pending	11	58566975	58566976	58566976	G	A	missense_variant
3-103812188-103812189-G-C		3		Ap4b1	G73A	1	LYMATYAPL	4	3	51.81	115.45	0.2	0.2	20.206	0	0	0	0.097	NoExpr	Pending	3	103812188	103812189	103812189	G	C	missense_variant
4-115532645-115532646-C-T		1	4	Cyp4a10	L493F	1	VPMPLARFVL	8	5	51.895	471.752	0.17	0.6	0	0	0	0	0.049	NoExpr	Pending	4	115532645	115532646	115532646	C	T	missense_variant
16-58824247-58824248-G-T		4		Olfr175	L154M	1	AYIAGNMHSM	7	4	52.16	61.3	0.1	0.1	0	0	0	0	0.066	NoExpr	Pending	16	58824247	58824248	58824248	G	T	missense_variant
10-69973804-69973805-T-G		2		Ank3	S1139A	1	QYFAVVARI	7	2	52.25	8.956	0.2	0.1	0.163	0	0	0	0.04	NoExpr	Pending	10	69973804	69973805	69973805	T	G	missense_variant
12-113597884-113597885-C-G		1	1	Ighv5-4	L7F	1	MNFGLSFIF	7	2	52.34	1314.71	0.3	4.1	0	0	0	0	0.056	NoExpr	Pending	12	113597884	113597885	113597885	C	G	missense_variant

ID	H-2-Dd	H-2-Kd	H-2-Ld	Gene	AA Change	Num Passing Transcripts	Best Peptide	Pos	Num Passing Peptides	IC50 MT	IC50 WT	%ile MT	%ile WT	RNA Expr	RNA VAF	Allele Expr	RNA Depth	DNA VAF	Tier	Evaluation	CHROM	START	END	POS	REF_y	ALT_y	biotype
4-147755804-147755805-T-A		3		Zfp984	K196N	1	KYLTQRENL	8	3	52.459	80.869	0.2	0.3	20.836	0	0	0	0.34	NoExpr	Pending	4	147755804	147755805	147755805	T	A	missense_variant
14-119041595-119041596-T-A		1		Uggt2	K833I	1	IYNTVGVNI	1	1	53.62	23.05	0.2	0.2	0.464	0	0	0	0.249	NoExpr	Pending	14	119041595	119041596	119041596	T	A	missense_variant
12-114937284-114937285-G-A		5		Ighv1-42	P60L	1	YYMNWVKQSL	10	5	54.25	2326.4	0.2	1.8	0	0	0	0	0.241	NoExpr	Pending	12	114937284	114937285	114937285	G	A	missense_variant
3-40910580-40910581-C-G		1		Abhd18	P86A	1	HYVPGIMAI	8	1	57.1	49.31	0.2	0.2	1.231	0	0	0	0.23	NoExpr	Pending	3	40910580	40910581	40910581	C	G	missense_variant
11-58757723-58757724-G-T		2	1	Olfr316	D20Y	1	YGHMDTFLF	1	3	57.412	155.955	0.3	0.8	0	0	0	0	0.051	NoExpr	Pending	11	58757723	58757724	58757724	G	T	missense_variant
19-39643354-39643355-T-A		3		Cyp2c67	N133Y	1	RYLGMGKRTI	2	3	57.94	2880.45	0.2	4.3	0	0	0	0	0.203	NoExpr	Pending	19	39643354	39643355	39643355	T	A	missense_variant
17-33999988-33999989-T-A	1	3	4	H2-K1	R27W	1	GPHSLWYFV	6	8	58.456	56.364	0.2	0.3	899.72	0	0	0	0.116	NoExpr	Pending	17	33999988	33999989	33999989	T	A,G	missense_variant
7-26611648-26611649-G-A		2	3	Vmn1r185	H144Y	1	LYMLMHFII	2	4	59.032	933.83	0.2	1.4	0	0	0	0	0.036	NoExpr	Pending	7	26611648	26611649	26611649	G	A	missense_variant
2-86978564-86978565-A-C		2		Olfr1100	I77S	1	SYSTVSTPKTL	6	2	59.09	96.18	0.2	0.2	0	0	0	0	0.155	NoExpr	Pending	2	86978564	86978565	86978565	A	C	missense_variant
5-109085949-109085950-C-T		3		Vmn2r12	A799T	1	VYHSTRGTV	8	3	59.09	126.84	0.4	0.4	0	0	0	0	0.019	NoExpr	Pending	5	109085949	109085950	109085950	C	T	missense_variant
17-37299650-37299651-G-A		5		Olfr101	T257I	1	IYIRPASGSSL	1	5	60.33	39.46	0.1	0.1	0	0	0	0	0.029	NoExpr	Pending	17	37299650	37299651	37299651	G	A	missense_variant
7-104564378-104564379-C-T		1	2	Olfr652	L53F	1	YFIFMERSL	2	3	60.407	211.393	0.3	1	0	0	0	0	0.208	NoExpr	Pending	7	104564378	104564379	104564379	C	T	missense_variant
13-22179086-22179087-A-T		4	1	Vmn1r191	C166S	1	SDNYSYFIL	5	5	60.814	56.364	0.3	0.3	0	0	0	0	0.157	NoExpr	Pending	13	22179086	22179087	22179087	A	T	missense_variant
17-37923941-37923943-CA-TG		1		Olfr128	AI125-126AV	1	RYVAVCLPL	5	1	61.12	102.603	0.3	0.5	0	0	0	0	0.004	NoExpr	Pending	17	37923941	37923943	37923942	CA	TG	missense_variant
10-79247983-79247984-C-T		2		Vmn2r81	P64L	1	KYFLMDSDN	4	2	61.981	32.156	0.3	0.2	0	0	0	0	0.208	NoExpr	Pending	10	79247983	79247984	79247984	C	T	missense_variant
9-96685966-96685967-C-A		1		Zbtb38	Q1021H	1	KHFQSSSTL	2	1	63.053	141.11	0.3	0.3	18.642	0	0	0	0.037	NoExpr	Pending	9	96685966	96685967	96685967	C	A	missense_variant
7-103584637-103584639-TG-CA		2		Olfr617	GV205-206GM	1	IYYGMIVAL	5	2	64.159	103.576	0.2	0.3	0	0	0	0	0.017	NoExpr	Pending	7	103584637	103584639	103584638	TG	CA	missense_variant
9-19249033-19249034-G-A		1	1	Olfr843	R122C	1	CYLAIVHPL	1	2	64.54	36.58	0.3	0.2	0	0	0	0	0.258	NoExpr	Pending	9	19249033	19249034	19249034	G	A	missense_variant
11-58529802-58529803-G-A		1	4	Olfr330	T61I	1	HLHIPMYFF	4	4	65.137	72.083	0.3	0.4	0	0	0	0	0.014	NoExpr	Pending	11	58529802	58529803	58529803	G	A	missense_variant
7-102659059-102659060-C-T		3	1	Olfr555	P80S	1	SSMLSIFWF	1	4	65.438	111.88	0.3	0.6	0	0	0	0	0.479	NoExpr	Pending	7	102659059	102659060	102659060	C	T	missense_variant
14-50736550-50736551-A-G		4	1	Olfr749	F204L	1	FYAQSSLVL	7	5	65.654	59.602	0.3	0.3	0	0	0	0	0.069	NoExpr	Pending	14	50736550	50736551	50736551	A	G	missense_variant
7-143536025-143536026-C-T		2		Nap1l4	V63I	1	SYIETLPKAI	10	2	65.68	164.32	0.2	0.3	86.366	0	0	0	0.3	NoExpr	Pending	7	143536025	143536026	143536026	C	T	missense_variant
6-29204633-29204634-T-C		1		Impdh1	Y282C	1	CQIAMVHYI	1	1	65.779	34.442	0.28	0.2	42.968	0	0	0	0.244	NoExpr	Pending	6	29204633	29204634	29204634	T	C	missense_variant
6-148842863-148842864-G-A		3		Caprin2	S1021F	1	KYFTFSGYL	3	3	65.842	115.15	0.3	0.4	3.63	0	0	0	0.605	NoExpr	Pending	6	148842863	148842864	148842864	G	A	missense_variant
19-4762288-4762289-A-G		4		Rbm4b	N242S	1	SYAEQTMSHL	1	4	66.07	212.94	0.2	0.32	7.929	0	0	0	0.37	NoExpr	Pending	19	4762288	4762289	4762289	A	G	missense_variant
2-180713220-180713221-C-T		1		Gid8	P7S	1	SYAEKSDEI	6	1	67.01	134.86	0.4	0.4	51.7	0	0	0	0.454	NoExpr	Pending	2	180713220	180713221	180713221	C	T	missense_variant
14-32659999-32660000-T-G	1	1	4	3425401B19Rik	Q1336P	1	SPLAAPTFL	2	5	68.522	1179.86	0.3	2.8	0.053	0	0	0	0.169	NoExpr	Pending	14	32659999	32660000	32660000	T	G	missense_variant
17-57224868-57224869-C-T		1	1	C3	V254I	1	YYIDDPNGLEI	11	2	69.16	254.608	0.2	0.6	68.522	0	0	0	0.317	NoExpr	Pending	17	57224868	57224869	57224869	C	T	missense_variant
16-32751992-32751993-C-A		2		Muc4	P624T	1	SYATIMSSS	4	2	69.677	62.374	0.22	0.24	0.047	0	0	0	0.232	NoExpr	Pending	16	32751992	32751993	32751993	C	A,T	missense_variant
10-100064109-100064110-G-C		1	2	Kitl	M52I	1	LPNDYIITL	6	3	69.796	96.79	0.3	0.4	40.462	0	0	0	0.135	NoExpr	Pending	10	100064109	100064110	100064110	G	C	missense_variant
19-11472471-11472472-A-C		4	1	Ms4a6c	N76T	1	YFTSVFSVL	3	5	70.349	95.556	0.3	0.3	78.803	0	0	0	0.032	NoExpr	Pending	19	11472471	11472472	11472472	A	C	missense_variant
15-44526819-44526820-C-T		2		Pkhd1l1	P1444L	1	YRIFSVSSL	9	2	71.3	644.303	0.4	2.6	0	0	0	0	0.067	NoExpr	Pending	15	44526819	44526820	44526820	C	T	missense_variant
17-33269092-33269093-G-A		1		Olfr63	R123H	1	HYVAICRPL	1	1	71.795	59.09	0.4	0.4	0	0	0	0	0.354	NoExpr	Pending	17	33269092	33269093	33269093	G	A	missense_variant
4-156338199-156338200-A-T		2		Vmn2r-ps159	I565L	1	KYANLEQTH	5	2	72.126	121.644	0.3	0.4	0	0	0	0	0.019	NoExpr	Pending	4	156338199	156338200	156338200	A	T	missense_variant
9-20314065-20314066-C-T		3		Olfr18	A285T	1	VYLSSTVSL	6	3	72.576	65.133	0.4	0.3	0	0	0	0	0.329	NoExpr	Pending	9	20314065	20314066	20314066	C	T	missense_variant
19-13103021-13103022-G-A		1	1	Olfr1458	A94V	1	SYNVCASQM	4	2	72.626	39.72	0.2	0.2	0	0	0	0	0.439	NoExpr	Pending	19	13103021	13103022	13103022	G	A	missense_variant
14-14931283-14931284-G-C		1		Nek10	G752A	1	VYEPVPEAI	8	1	72.654	173.884	0.2	0.4	0	0	0	0	0.294	NoExpr	Pending	14	14931283	14931284	14931284	G	C	missense_variant
2-87213695-87213696-G-A		3		Olfr1113	G268E	1	FYESASITYL	3	3	72.89	86.52	0.2	0.2	0	0	0	0	0.351	NoExpr	Pending	2	87213695	87213696	87213696	G	A	missense_variant
9-38606772-38606773-T-G		2	3	Olfr914	F103V	1	MTQFYVFGF	6	5	73.365	51.895	0.4	0.22	0	0	0	0	0.174	NoExpr	Pending	9	38606772	38606773	38606773	T	G	missense_variant
10-116353855-116353856-G-A		2		Ptprb	A1520T	1	SYLEYRHNTSI	9	2	73.572	207.434	0.2	0.4	2.163	0	0	0	0.111	NoExpr	Pending	10	116353855	116353856	116353856	G	A	missense_variant
X-23958723-23958724-T-C		1		Gm4985	K71E	1	KYINLIAKLEL	10	1	74.111	113.097	0.2	0.29	0	0	0	0	0.275	NoExpr	Pending	X	23958723	23958724	23958724	T	C	missense_variant
8-46844829-46844830-C-G		2		Irf2	T235S	1	SYAESESTD	7	2	74.66	92.43	0.3	0.3	36.152	0	0	0	0.048	NoExpr	Pending	8	46844829	46844830	46844830	C	G	missense_variant
11-115205094-115205095-C-T		3	1	Tmem104	T154I	1	IYLYGDLAI	1	3	75.381	150.48	0.2	0.3	6.025	0	0	0	0.237	NoExpr	Pending	11	115205094	115205095	115205095	C	T	missense_variant
9-39819802-39819803-C-T		1	3	Olfr970	L55F	1	HFHTPMYYF	2	4	75.48	94.805	0.4	0.5	0	0	0	0	0.245	NoExpr	Pending	9	39819802	39819803	39819803	C	T	missense_variant
X-100594004-100594005-G-A		2		P2ry4	H96Y	1	YYYAARNYW	8	2	75.525	77.999	0.2	0.3	0	0	0	0	0.065	NoExpr	Pending	X	100594004	100594005	100594005	G	A	missense_variant
1-173926891-173926892-C-T		1		Ifi203	E758K	1	SNTLSKSAI	6	1	76.048	93.56	0.4	0.4	166.52	0	0	0	0.241	NoExpr	Pending	1	173926891	173926892	173926892	C	T	missense_variant
12-115145523-115145524-A-G		1		Ighv1-52	L105P	1	AYMQLSSPT	8	1	76.606	123.905	0.2	0.4	0	0	0	0	0.063	NoExpr	Pending	12	115145523	115145524	115145524	A	G	missense_variant
2-107295584-107295585-T-G		1	1	Kcna4	F221L	1	DPLRNEYLF	8	2	77.594	27.786	0.4	0.2	0.007	0	0	0	0.029	NoExpr	Pending	2	107295584	107295585	107295585	T	G	missense_variant
4-86583171-86583172-C-T		2		Haus6	A821T	1	SYETLKKSL	4	2	78.21	44.082	0.3	0.2	14.961	0	0	0	0.501	NoExpr	Pending	4	86583171	86583172	86583172	C	T	missense_variant
2-87358133-87358134-C-T		2		Olfr1120	T230I	1	SYSKIIAII	8	2	78.933	37.919	0.3	0.2	0	0	0	0	0.395	NoExpr	Pending	2	87358133	87358134	87358134	C	T	missense_variant
2-87846960-87846961-C-T		2		Olfr1150-ps1	T230I	1	SYSKIIAII	8	2	78.933	37.919	0.3	0.2	0	0	0	0	0.35	NoExpr	Pending	2	87846960	87846961	87846961	C	T	missense_variant
3-145077971-145077972-A-T		1		Clca2	N627K	1	IYAKVRKGL	4	1	79.115	142.646	0.3	0.4	1.457	0	0	0	0.034	NoExpr	Pending	3	145077971	145077972	145077972	A	T	missense_variant
1-32414963-32414964-G-C		1		Khdrbs2	A144P	1	PYSRMSHAL	1	1	79.298	18.124	0.3	0.2	0	0	0	0	0.138	NoExpr	Pending	1	32414963	32414964	32414964	G	C	missense_variant
7-3717393-3717394-C-A		4		Pirb	V327L	1	YYEPRLSLL	8	4	79.816	51.179	0.3	0.2	15.122	0	0	0	0.034	NoExpr	Pending	7	3717393	3717394	3717394	C	A	missense_variant
10-84591235-84591236-A-C		3		Tcp11l2	K188T	1	NYVITTMGTI	9	3	79.95	445.8	0.2	0.61	16.121	0	0	0	0.249	NoExpr	Pending	10	84591235	84591236	84591236	A	C	missense_variant
11-103631333-103631334-G-C		1		Lyzl6	C145W	1	SYWMTGWHL	7	1	79.999	115.571	0.3	0.4	0	0	0	0	0.157	NoExpr	Pending	11	103631333	103631334	103631334	G	C	missense_variant
12-86058952-86058953-A-C		2		Tgfb3	F343V	1	YYANVCSGP	5	2	80.555	556.019	0.4	1.8	21.21	0	0	0	0.233	NoExpr	Pending	12	86058952	86058953	86058953	A	C	missense_variant
4-88683332-88683333-C-G		5		Ifna2	R149S	1	KYFHSITVYL	5	5	80.72	266.59	0.2	0.41	0	0	0	0	0.047	NoExpr	Pending	4	88683332	88683333	88683333	C	G	missense_variant
4-88835970-88835971-G-C		5		Ifna5	R149S	1	KYFHSITVYL	5	5	80.72	266.59	0.2	0.41	0	0	0	0	0.02	NoExpr	Pending	4	88835970	88835971	88835971	G	C	missense_variant
12-113702557-113702558-C-G		1	1	Ighv5-12	L7F	1	MNLGLSFIF	7	2	80.878	2031.56	0.4	5.8	0	0	0	0	0.055	NoExpr	Pending	12	113702557	113702558	113702558	C	G	missense_variant
17-35266221-35266222-G-A		4		H2-D1	V311I	1	SYMVIIAVL	6	4	81.487	82.431	0.3	0.3	1425.5	0	0	0	0.048	NoExpr	Pending	17	35266221	35266222	35266222	G	A	missense_variant
6-40464143-40464144-T-G		1		Wee2	M461R	1	FYGLLKNRI	8	1	82.241	79.632	0.2	0.2	0	0	0	0	0.171	NoExpr	Pending	6	40464143	40464144	40464144	T	G	missense_variant

ID	H-2-Dd	H-2-Kd	H-2-Ld	Gene	AA Change	Num Passing Transcripts	Best Peptide	Pos	Num Passing Peptides	IC50 MT	IC50 WT	%ile MT	%ile WT	RNA Expr	RNA VAF	Allele Expr	RNA Depth	DNA VAF	Tier	Evaluation	CHROM	START	END	POS	REF_y	ALT_y	biotype
4-100441382-100441383-A-T		5		Ror1	K651M	1	YYRVQSMSSL	7	5	83.537	89.14	0.2	0.2	0.016	0	0	0	0.362	NoExpr	Pending	4	100441382	100441383	100441383	A	T	missense_variant
2-89283062-89283063-T-G		1	3	Olfr1229	K23N	1	NPNVQNIIF	6	4	84.446	74.97	0.3	0.34	0	0	0	0	0.146	NoExpr	Pending	2	89283062	89283063	89283063	T	G	missense_variant
9-89090620-89090621-T-A		1		Trim43b	K160I	1	NYVEIERRTTL	5	1	85.483	241.32	0.2	0.31	0.269	0	0	0	0.091	NoExpr	Pending	9	89090620	89090621	89090621	T	A	missense_variant
11-71122723-71122724-G-A		2		Nlrp1a	H567Y	1	KYLVIQTDM	2	2	85.69	1222.05	0.3	3.4	0.059	0	0	0	0.059	NoExpr	Pending	11	71122723	71122724	71122724	G	A	missense_variant
11-58684276-58684277-C-T		1	2	Olfr320	L135F	1	RPLHYPVFM	8	3	86.099	190.108	0.4	0.8	0	0	0	0	0.046	NoExpr	Pending	11	58684276	58684277	58684277	C	T	missense_variant
2-86958780-86958781-A-T		3		Olfr1099	L226M	1	SYLSILSTIM	10	3	86.293	65.133	0.2	0.2	0	0	0	0	0.044	NoExpr	Pending	2	86958780	86958781	86958781	A	T	missense_variant
6-42838383-42838384-T-G		2		Olfr449	F168V	1	IYFISRLSV	9	2	86.43	839.963	0.4	1.3	0	0	0	0	0.213	NoExpr	Pending	6	42838383	42838384	42838384	T	G	missense_variant
10-127658676-127658677-G-A		1		Stat6	M703I	1	SFQSLEESI	9	1	86.714	442.1	0.3	0.8	79.234	0	0	0	0.238	NoExpr	Pending	10	127658676	127658677	127658677	G	A	missense_variant
10-121615051-121615052-C-G		1		Xpot	A148P	1	LYLRILMPI	8	1	88	58.088	0.2	0.2	70.379	0	0	0	0.311	NoExpr	Pending	10	121615051	121615052	121615052	C	G	missense_variant
6-146396964-146396965-G-A		1	2	Itpr2	L366F	1	NDIASFFEL	6	3	89.501	138.302	0.4	0.6	12.124	0	0	0	0.213	NoExpr	Pending	6	146396964	146396965	146396965	G	A	missense_variant
1-58752442-58752443-G-C		1	3	Cflar	A302P	1	MPQHQDYDS	2	4	89.743	1104.08	0.4	3.5	99.151	0	0	0	0.153	NoExpr	Pending	1	58752442	58752443	58752443	G	C	missense_variant
9-109722218-109722219-A-C		3		Fbxw26	F337V	1	GYLTASVSL	7	3	90.11	113.097	0.4	0.6	0	0	0	0	0.276	NoExpr	Pending	9	109722218	109722219	109722219	A	C	missense_variant
11-71114444-71114445-T-G		2		Nlrp1a	Y648S	1	SYSAVHSLC	3	2	91.22	64.282	0.3	0.22	0.059	0	0	0	0.078	NoExpr	Pending	11	71114444	71114445	71114445	T	G	missense_variant
7-7476935-7476937-CA-GG		1	1	Vmn2r32	V79A	1	YALALAFAM	6	2	92.009	77.09	0.4	0.4	0	0	0	0	0.025	NoExpr	Pending	7	7476935	7476937	7476936	CA	GG	missense_variant
7-3842293-3842294-C-T		4		Pira2	E322K	1	YYKPRLSVL	3	4	92.489	51.179	0.3	0.2	8.62	0	0	0	0.067	NoExpr	Pending	7	3842293	3842294	3842294	C	T	missense_variant
7-49464889-49464890-T-C		1		Nav2	V874A	1	GYMSDGDAL	8	1	93.083	214.93	0.5	0.6	18.378	0	0	0	0.194	NoExpr	Pending	7	49464889	49464890	49464890	T	C	missense_variant
17-23900506-23900509-TAA-CTC	1	3		Dcpp2	YN98-99YS	1	TYSPSALTQI	3	4	93.38	65.44	0.2	0.2	0	0	0	0	0.029	NoExpr	Pending	17	23900506	23900509	23900507	TAA	CTC	missense_variant
10-52101838-52101839-A-T		4		Ros1	L1458H	1	LYATNTSHTL	8	4	93.6	194.09	0.2	0.3	0	0	0	0	0.295	NoExpr	Pending	10	52101838	52101839	52101839	A	T	missense_variant
2-111983699-111983700-C-G		1	2	Olfr1309	A133P	1	RYVPICKPL	4	3	94.095	96.154	0.5	0.5	0	0	0	0	0.274	NoExpr	Pending	2	111983699	111983700	111983700	C	G	missense_variant
9-50913501-50913502-G-C		1	3	Sik2	A328G	1	YNHFAGIYF	6	4	94.095	108.306	0.4	0.6	11.496	0	0	0	0.067	NoExpr	Pending	9	50913501	50913502	50913502	G	C	missense_variant
3-127562336-127562337-A-G		1		Zgrf1	N404S	1	SFCKSSSVL	5	1	94.095	242.01	0.3	0.6	9.63	0	0	0	0.062	NoExpr	Pending	3	127562336	127562337	127562337	A	G	missense_variant
6-57002713-57002714-A-C		2	1	Vmn1r6	K120N	1	LNNYMIYAF	2	3	94.189	202.768	0.5	0.9	0	0	0	0	0.3	NoExpr	Pending	6	57002713	57002714	57002714	A	C	missense_variant
9-3450127-3450128-C-T		1	2	Cwf19l2	L610F	1	DINQNRFFM	7	3	95.94	2558.59	0.5	4.4	12.51	0	0	0	0.463	NoExpr	Pending	9	3450127	3450128	3450128	C	T	missense_variant
2-86508739-86508740-A-T		2	1	Olfr1076	I94F	1	EQNTISFYF	7	3	97.2	688.934	0.5	2.2	0	0	0	0	0.045	NoExpr	Pending	2	86508739	86508740	86508740	A	T	missense_variant
17-20114053-20114054-G-A		2		Fpr-rs7	T58I	1	IYVAGFRMTHI	11	2	97.6	1179.53	0.2	2.3	0	0	0	0	0.028	NoExpr	Pending	17	20114053	20114054	20114054	G	A	missense_variant
9-3002229-3002230-A-T		1	3	Gm10722	Y184F	1	RSFSVHFSF	3	4	98.257	192.234	0.5	0.8	0.048	0	0	0	0.1	NoExpr	Pending	9	3002229	3002230	3002230	A	T	missense_variant
2-86988394-86988395-A-C		2		Olfr1101	S260R	1	MYMRPRSSYTI	6	2	98.43	93.15	0.2	0.2	0	0	0	0	0.153	NoExpr	Pending	2	86988394	86988395	86988395	A	C	missense_variant
18-49723694-49723695-A-C		1	1	Dtwd2	I175S	1	VYPSTISLI	7	2	98.46	676.488	0.3	2.2	6.309	0	0	0	0.08	NoExpr	Pending	18	49723694	49723695	49723695	A	C	missense_variant
9-66462954-66462955-G-A		2		Herc1	G3008R	1	RYRSNGSYV	1	2	98.59	115.571	0.4	0.6	14.776	0	0	0	0.551	NoExpr	Pending	9	66462954	66462955	66462955	G	A	missense_variant
12-115861900-115861901-G-T		1		Ighv1-77	T106K	1	AYMQLSSLK	9	1	98.648	123.905	0.4	0.4	0	0	0	0	0.062	NoExpr	Pending	12	115861900	115861901	115861901	G	T	missense_variant
5-14676235-14676236-C-T		1		Pclo	P1703S	1	SHGPLLSTI	7	1	98.876	659.754	0.3	2.1	0	0	0	0	0.37	NoExpr	Pending	5	14676235	14676236	14676236	C	T	missense_variant
X-102520774-102520775-T-G		3		Phka1	K1092N	1	FYQNVWKIL	4	3	99.86	61.389	0.3	0.3	6.385	0	0	0	0.266	NoExpr	Pending	X	102520774	102520775	102520775	T	G	missense_variant
9-44818767-44818768-C-T		2		Kmt2a	M3414I	1	AAITAASSI	3	2	100.48	127.391	0.4	0.3	10.11	0	0	0	0.143	NoExpr	Pending	9	44818767	44818768	44818768	C	T	missense_variant
10-94579064-94579065-G-A		2		Tmcc3	G241R	1	AYGRSATIV	4	2	101.63	197.738	0.4	0.4	4.941	0	0	0	0.175	NoExpr	Pending	10	94579064	94579065	94579065	G	A	missense_variant
8-20944652-20944653-C-T		2		AY761185	D20N	1	FYVQANSTQ	6	2	101.88	107.421	0.3	0.4	0.067	0	0	0	0.584	NoExpr	Pending	8	20944652	20944653	20944653	C	T	missense_variant
1-85610757-85610759-AG-GC		3		Sp140	K72S	1	EYQETCSNL	7	3	101.92	748.618	0.3	1.2	95.257	0	0	0	0.023	NoExpr	Pending	1	85610757	85610759	85610758	AG	GC,GG	missense_variant
12-115193725-115193726-C-A		2		Ighv1-54	M100I	1	AYIQLSSLT	3	2	102.35	123.905	0.3	0.4	0	0	0	0	0.003	NoExpr	Pending	12	115193725	115193726	115193726	C	A	missense_variant
12-115242852-115242853-C-A		2		Ighv1-56	M100I	1	AYIQLSSLT	3	2	102.35	123.905	0.3	0.4	0	0	0	0	0.036	NoExpr	Pending	12	115242852	115242853	115242853	C	A	missense_variant
11-71109048-71109049-G-A		1	2	Nlrp1a	P762S	1	KPHVMVSTM	7	2	102.6	27.102	0.4	0.2	0.059	0	0	0	0.051	NoExpr	Pending	11	71109048	71109049	71109049	G	A	missense_variant
9-3023630-3023632-TG-AT		1	7	Gm10718	SV27-28SF	1	FPPFSFFL	6	8	102.96	554.879	0.18	0.39	0	0	0	0	0.015	NoExpr	Pending	9	3023630	3023632	3023631	TG	AG,AT	missense_variant
7-85624374-85624375-G-A		1		Vmn2r71	S799N	1	VYHNTKGKI	4	1	103.58	77.137	0.3	0.3	0	0	0	0	0.054	NoExpr	Pending	7	85624374	85624375	85624375	G	A	missense_variant
2-87489670-87489671-A-C		1		Pramel7	I426R	1	NYVRVEIFI	4	1	103.77	242.153	0.3	0.5	0	0	0	0	0.169	NoExpr	Pending	2	87489670	87489671	87489671	A	C	missense_variant
17-22572068-22572070-TT-AC		1	2	Vmn2r111	IN82-83MY	1	YALALAFSM	9	3	103.95	348.201	0.4	0.8	0	0	0	0	0.039	NoExpr	Pending	17	22572068	22572070	22572069	TT	AC	missense_variant
7-9027831-9027832-A-G		2	1	Vmn2r39	V79A	1	YALALAFSM	6	3	103.95	84.69	0.4	0.3	0	0	0	0	0.15	NoExpr	Pending	7	9027831	9027832	9027832	A	G	missense_variant
12-113716789-113716791-GT-TA		3		Ighv2-6	T77Y	1	TYYNSALKSRL	2	3	104.35	976.967	0.2	2.2	0.108	0	0	0	0.045	NoExpr	Pending	12	113716789	113716791	113716790	GT	TA,TC	missense_variant
15-41096161-41096162-G-A		2		Zfpm2	M212I	1	SRLQAASHI	9	2	104.49	396.36	0.3	1.2	30.842	0	0	0	0.131	NoExpr	Pending	15	41096161	41096162	41096162	G	A	missense_variant
7-140336052-140336053-T-G		2		Olfr527	L64V	1	YFFLVNVAI	7	2	105.02	105.017	0.4	0.5	0	0	0	0	0.147	NoExpr	Pending	7	140336052	140336053	140336053	T	G	missense_variant
11-36047471-36047472-A-T		2		Tenm2	L1458H	1	DYSLSKHAI	7	2	105.5	246.752	0.3	0.4	0	0	0	0	0.242	NoExpr	Pending	11	36047471	36047472	36047472	A	T	missense_variant
16-59216159-59216160-A-C		2		Olfr199	L151R	1	AYLRGALHL	4	2	105.7	170.612	0.3	0.6	0	0	0	0	0.264	NoExpr	Pending	16	59216159	59216160	59216160	A	C	missense_variant
2-76789486-76789487-C-T		1		Ttn	V14200I	1	FRIAAENAI	3	1	106.23	515.537	0.3	1	0.231	0	0	0	0.334	NoExpr	Pending	2	76789486	76789487	76789487	C	T	missense_variant
12-114914640-114914641-G-C		3		Ighv1-39	N103K	1	AYMQLKSLT	6	3	106.93	122.487	0.3	0.4	0	0	0	0	0.004	NoExpr	Pending	12	114914640	114914641	114914641	G	C	missense_variant
11-60762537-60762538-C-T		3		Top3a	V82I	1	LYGQNITMI	6	3	107.71	113.308	0.3	0.3	6.246	0	0	0	0.579	NoExpr	Pending	11	60762537	60762538	60762538	C	T	missense_variant
11-74206597-74206598-A-G		1		Olfr43	F206S	1	MYLGVGVSSV	8	1	108.01	251.868	0.1	0.27	0	0	0	0	0.264	NoExpr	Pending	11	74206597	74206598	74206598	A	G	missense_variant
13-68689306-68689307-A-T		2	1	Adcy2	L641H	1	AFHSLIFIL	3	3	108.89	270.29	0.5	1.1	0.042	0	0	0	0.156	NoExpr	Pending	13	68689306	68689307	68689307	A	T	missense_variant
10-75638159-75638160-G-A	2	1		Susd2	H654Y	1	KYNPNFKPL	2	3	109.39	1815.73	0.5	6.8	0.727	0	0	0	0.252	NoExpr	Pending	10	75638159	75638160	75638160	G	A	missense_variant
2-87833826-87833827-G-C		3		Olfr1148	V263L	1	FYGSATLTYL	7	3	109.47	113.49	0.2	0.2	0	0	0	0	0.152	NoExpr	Pending	2	87833826	87833827	87833827	G	C	missense_variant
5-61809303-61809304-G-T		2		G6pd2	A141S	1	FYLSLPPTV	4	2	109.49	101.499	0.5	0.4	0	0	0	0	0.144	NoExpr	Pending	5	61809303	61809304	61809304	G	T	missense_variant
11-58831539-58831540-A-C		2	1	Olfr312	I129L	1	RPLLYGTIM	4	3	109.61	78.855	0.5	0.4	0	0	0	0	0.01	NoExpr	Pending	11	58831539	58831540	58831540	A	C	missense_variant
2-129208196-129208197-C-T		3		Slc20a1	T425I	1	SYTSYIMAI	6	3	110.68	97.1	0.3	0.3	170.79	0	0	0	0.471	NoExpr	Pending	2	129208196	129208197	129208197	C	T	missense_variant
17-35380616-35380617-G-T		2		H2-Q4	K225N	1	RYLELGNETLL	7	2	111.1	143.492	0.2	0.2	256.73	0	0	0	0.073	NoExpr	Pending	17	35380616	35380617	35380617	G	T	missense_variant
18-84013927-84013928-T-C		2		Tshz1	N785S	1	LYKISSSML	6	2	111.5	125.05	0.3	0.3	23.599	0	0	0	0.556	NoExpr	Pending	18	84013927	84013928	84013928	T	C	missense_variant
16-30308613-30308614-G-A		1	2	Gp5	T414I	1	LPQLIQVLL	5	3	111.64	149.624	0.5	0.7	0	0	0	0	0.369	NoExpr	Pending	16	30308613	30308614	30308614	G	A	missense_variant

ID	H-2-Dd	H-2-Kd	H-2-Ld	Gene	AA Change	Num Passing Transcripts	Best Peptide	Pos	Num Passing Peptides	IC50 MT	IC50 WT	%ile MT	%ile WT	RNA Expr	RNA VAF	Allele Expr	RNA Depth	DNA VAF	Tier	Evaluation	CHROM	START	END	POS	REF_y	ALT_y	biotype
1-59199148-59199149-G-T		4		Als2	L675I	1	SYIALVDKNI	3	4	111.89	137.49	0.2	0.2	12.417	0	0	0	0.131	NoExpr	Pending	1	59199148	59199149	59199149	G	T	missense_variant
12-59160181-59160182-G-A		3		Mia2	A317T	1	IYAAKLNTSL	8	3	111.91	869.49	0.2	1.2	51.472	0	0	0	0.454	NoExpr	Pending	12	59160181	59160182	59160182	G	A	missense_variant
2-87252095-87252096-T-C		3		Olfr1115	I53T	1	FFIYMSTLL	7	3	112.76	597.159	0.2	1.5	0	0	0	0	0.039	NoExpr	Pending	2	87252095	87252096	87252096	T	C	missense_variant
11-71181702-71181703-C-T		4		Nlrp1b	C438Y	1	EFFASMSYI	8	4	113.78	222.377	0.2	0.6	2.359	0	0	0	0.05	NoExpr	Pending	11	71181702	71181703	71181703	C	T	missense_variant
17-37850800-37850801-G-T		4		Olfr126	D70Y	1	LYLSSLSVTV	2	4	115.57	4335.14	0.3	5.3	0	0	0	0	0.02	NoExpr	Pending	17	37850800	37850801	37850801	G	T	missense_variant
6-40571579-40571580-C-T		2	2	Olfr460	L65F	1	YFFLVHFSI	7	3	115.57	114.62	0.59	0.5	0	0	0	0	0.826	NoExpr	Pending	6	40571579	40571580	40571580	C	T	missense_variant
7-10274335-10274336-A-T		2		Vmn1r66	L257I	1	AFYTLSSII	9	2	115.9	291.66	0.3	0.6	0	0	0	0	0.214	NoExpr	Pending	7	10274335	10274336	10274336	A	T	missense_variant
12-114479302-114479303-C-T		2		Ighv10-1	V21M	1	FYQGVHCEM	9	2	116.17	133.77	0.2	0.3	0	0	0	0	0.157	NoExpr	Pending	12	114479302	114479303	114479303	C	T	missense_variant
7-26611642-26611643-G-C		1	1	Vmn1r185	L146V	1	LHMVMHFII	4	1	117.22	138.357	0.6	0.6	0	0	0	0	0.058	NoExpr	Pending	7	26611642	26611643	26611643	G	C	missense_variant
5-94535049-94535050-T-C		2	2	Gm3139	S68P	1	GPLINMHNL	2	4	117.44	2207.14	0.46	5.6	0	0	0	0	0.027	NoExpr	Pending	5	94535049	94535050	94535050	T	C	missense_variant
10-116314083-116314084-G-C		3		Ptprb	G200A	1	VYINASTV	5	3	117.49	149.93	0.14	0.21	2.163	0	0	0	0.201	NoExpr	Pending	10	116314083	116314084	116314084	G	C	missense_variant
X-143101994-143101995-G-T		2		Rtl9	R801I	1	SITPVYGTI	2	2	118.1	92.276	0.6	0.4	0.054	0	0	0	0.663	NoExpr	Pending	X	143101994	143101995	143101995	G	T	missense_variant
6-132803745-132803747-GA-TT		2	2	Tas2r117	PT282-283PS	1	FPSFHSYIL	3	4	118.58	169.044	0.2	0.3	0	0	0	0	0.13	NoExpr	Pending	6	132803745	132803747	132803746	GA	TT	missense_variant
12-115145530-115145531-T-A		2		Ighv1-52	S103C	1	AYMQLCSLT	6	2	119.15	123.905	0.3	0.4	0	0	0	0	0.049	NoExpr	Pending	12	115145530	115145531	115145531	T	A	missense_variant
12-115359183-115359184-T-A		2		Ighv1-61	S103C	1	AYMQLCSLT	6	2	119.15	123.905	0.3	0.4	0	0	0	0	0.105	NoExpr	Pending	12	115359183	115359184	115359184	T	A	missense_variant
6-70961016-70961017-G-A		4	2	Foxi3	S385N	1	SPFYNFNM	7	6	120	574.93	0.1	0.1	0	0	0	0	0.047	NoExpr	Pending	6	70961016	70961017	70961017	G	A	missense_variant
15-101528756-101528757-T-C		1		Krt84	T324A	1	SHISEASVI	6	1	120.02	186.78	0.4	0.48	0	0	0	0	0.057	NoExpr	Pending	15	101528756	101528757	101528757	T	C	missense_variant
5-71869471-71869472-A-C		3		Gabrb1	N105T	1	SYSGIPLTL	8	3	120.25	230.192	0.4	0.84	0.016	0	0	0	0.189	NoExpr	Pending	5	71869471	71869472	71869472	A	C	missense_variant
X-138220466-138220467-C-G		2		Il1rapl2	R147G	1	CYNSRIGYL	7	2	120.45	125.49	0.3	0.3	0	0	0	0	0.1	NoExpr	Pending	X	138220466	138220467	138220467	C	G	missense_variant
2-98662330-98662331-G-C		1	1	Gm10801	S32T	1	FSFSMIFTF	8	2	120.5	100.462	0.5	0.4	0.204	0	0	0	0.042	NoExpr	Pending	2	98662330	98662331	98662331	G	C	missense_variant
8-110415777-110415778-A-C		3	1	Hydin	E763A	1	YPYAKTIQL	4	4	120.73	216.686	0.6	0.7	0	0	0	0	0.167	NoExpr	Pending	8	110415777	110415778	110415778	A	C	missense_variant
7-43815408-43815409-A-C		1		Klk7	K236T	1	VYTQVCTYK	7	1	121.98	765.755	0.4	1.4	0	0	0	0	0.18	NoExpr	Pending	7	43815408	43815409	43815409	A	C	missense_variant
2-178403761-178403762-C-A		1	1	Sycp2	D22Y	1	NYFKPLLAL	2	2	122	5167.26	0.39	14	0.019	0	0	0	0.238	NoExpr	Pending	2	178403761	178403762	178403762	C	A	missense_variant
9-3001430-3001431-T-A		1		Gm10722	F169Y	1	NYPPFSSSI	2	1	123.32	1133.26	0.44	4.1	0.048	0	0	0	0.03	NoExpr	Pending	9	3001430	3001431	3001431	T	A	missense_variant
10-130210776-130210777-G-A		1	2	Olfr827	L118F	1	QLFLYAFFM	7	3	123.32	3000.81	0.59	4.8	0	0	0	0	0.62	NoExpr	Pending	10	130210776	130210777	130210777	G	A	missense_variant
12-115193709-115193710-T-A		1		Ighv1-54	T106S	1	AYMQLSSLS	9	1	123.34	123.905	0.4	0.4	0	0	0	0	0.005	NoExpr	Pending	12	115193709	115193710	115193710	T	A	missense_variant
12-115242836-115242837-T-A		1		Ighv1-56	T106S	1	AYMQLSSLS	9	1	123.34	123.905	0.4	0.4	0	0	0	0	0.036	NoExpr	Pending	12	115242836	115242837	115242837	T	A	missense_variant
9-18856953-18856954-G-A		1		Olfr829	V110I	1	TYGGCLTQI	9	1	123.63	423.79	0.4	1.4	0	0	0	0	0.463	NoExpr	Pending	9	18856953	18856954	18856954	G	A	missense_variant
7-101504601-101504602-G-C		1		Gm45837	G485A	1	IRIPADQAI	8	1	123.67	295.978	0.4	0.8	6.522	0	0	0	0.228	NoExpr	Pending	7	101504601	101504602	101504602	G	C	missense_variant
1-85610789-85610790-G-T		1		Sp140	D83Y	1	YYILSNVQK	2	1	124.24	5435.76	0.5	18	95.257	0	0	0	0.02	NoExpr	Pending	1	85610789	85610790	85610790	G	T	missense_variant
5-86906598-86906599-A-C		2	3	Ugt2b34	F108V	1	LPKQSVWGYFI	6	5	124.63	77.16	0.17	0.14	0	0	0	0	0.076	NoExpr	Pending	5	86906598	86906599	86906599	A	C	missense_variant
12-91825362-91825363-C-T		2		Sel1l	A299T	1	RYWTGIGVL	4	2	124.87	187.107	0.4	0.4	42.491	0	0	0	0.356	NoExpr	Pending	12	91825362	91825363	91825363	C	T	missense_variant
9-39184494-39184495-T-C		1	5	Olfr943	F106L	1	GCMAQLYLF	8	5	125.27	44.857	0.6	0.2	0	0	0	0	0.335	NoExpr	Pending	9	39184494	39184495	39184495	T	C	missense_variant
7-18526226-18526227-G-A		2		Psg25	R249W	1	RYWKMKLAHI	3	2	125.67	467.66	0.2	0.49	0	0	0	0	0.028	NoExpr	Pending	7	18526226	18526227	18526227	G	A	missense_variant
14-60027953-60027954-T-G		1		Atp8a2	K311T	1	WYIKTMDTN	5	1	127.43	1120.11	0.4	2.1	0.029	0	0	0	0.287	NoExpr	Pending	14	60027953	60027954	60027954	T	G	missense_variant
9-15007975-15007976-C-G		1	1	Panx1	E196Q	1	KYPIVQQYL	6	2	129.15	126.5	0.4	0.4	2.053	0	0	0	0.1	NoExpr	Pending	9	15007975	15007976	15007976	C	G	missense_variant
7-23984770-23984772-AT-CC		1	1	Vmn1r181	IF220-221IL	1	HRQRMQYIL	9	2	130.02	92.683	0.6	0.5	0	0	0	0	0.056	NoExpr	Pending	7	23984770	23984772	23984771	AT	CC	missense_variant
7-9986994-9986995-G-T		2		Vmn2r49	P190T	1	LYQMATKDTSL	6	2	130.18	150.28	0.2	0.26	0	0	0	0	0.072	NoExpr	Pending	7	9986994	9986995	9986995	G	T	missense_variant
7-9942458-9942459-C-T		3		Vmn2r48	S365N	1	NYVNSASNCK	4	3	130.23	83.537	0.2	0.2	0	0	0	0	0.293	NoExpr	Pending	7	9942458	9942459	9942459	C	T	missense_variant
6-57956170-57956171-T-G		2	1	Vmn1r24	K121Q	1	LKQYIIYAF	3	3	131.59	613.762	0.8	2.5	0	0	0	0	0.475	NoExpr	Pending	6	57956170	57956171	57956171	T	G	missense_variant
12-98815984-98815985-T-G		2		Eml5	D1396A	1	HYLNDGDAI	8	2	131.72	437.17	0.3	0.61	6.1	0	0	0	0.25	NoExpr	Pending	12	98815984	98815985	98815985	T	G	missense_variant
2-87693491-87693492-A-T		1	1	Olfr1136	L130H	1	NPHMYAVDM	3	2	131.83	265.461	0.3	1.1	0	0	0	0	0.217	NoExpr	Pending	2	87693491	87693492	87693492	A	T	missense_variant
7-42011872-42011873-C-A		2		Vmn2r59	R839S	1	CYIILLSPE	7	2	131.85	437.613	0.4	1.3	0	0	0	0	0.162	NoExpr	Pending	7	42011872	42011873	42011873	C	A	missense_variant
4-156334442-156334443-C-T		1		Vmn2r-ps159	A340V	1	KFMQTMNTV	9	1	133.03	762.237	0.4	2.3	0	0	0	0	0.076	NoExpr	Pending	4	156334442	156334443	156334443	C	T	missense_variant
14-53363953-53363954-G-A		2		Trav13n-4	G60E	1	FYQRPEGRL	6	2	133.85	123.42	0.4	0.4	0.122	0	0	0	0.066	NoExpr	Pending	14	53363953	53363954	53363954	G	A	missense_variant
16-32753805-32753806-A-T		1	1	Muc4	Q1227H	1	APITSIHIL	7	1	134.84	328.852	0.6	1.2	0.047	0	0	0	0.029	NoExpr	Pending	16	32753805	32753806	32753806	A	T	missense_variant
11-58551585-58551586-A-T		4		Olfr328	S218T	1	SYTSILLTV	3	4	135.94	166.96	0.5	0.7	0	0	0	0	0.031	NoExpr	Pending	11	58551585	58551586	58551586	A	T	missense_variant
17-35380576-35380578-CG-AG		1		H2-Q4	A212E	1	AYLEGECVQSL	6	1	137.41	128.777	0.2	0.2	256.73	0	0	0	0.024	NoExpr	Pending	17	35380576	35380578	35380577	CG	AG,AA	missense_variant
7-7384459-7384460-A-T		3	1	Vmn2r31	I704K	1	NYIIPKCSL	6	4	139.4	185.038	0.33	0.4	0	0	0	0	0.069	NoExpr	Pending	7	7384459	7384460	7384460	A	T	missense_variant
7-8367934-8367935-A-T		1	2	Vmn2r44	I704K	1	NYIIPKCSL	6	3	139.4	185.038	0.33	0.4	0	0	0	0	0.065	NoExpr	Pending	7	8367934	8367935	8367935	A	T	missense_variant
14-37092018-37092019-G-A		1		Cdhr1	A150V	1	ENIPVGSSI	5	1	139.99	274.322	0.4	0.8	0	0	0	0	0.796	NoExpr	Pending	14	37092018	37092019	37092019	G	A	missense_variant
14-50298648-50298649-A-C		1		Olfr733	V220G	1	SYAGILCHV	4	1	140.42	158.168	0.6	0.6	0	0	0	0	0.224	NoExpr	Pending	14	50298648	50298649	50298649	A	C	missense_variant
1-188415854-188415855-C-T		1		Ush2a	T993I	1	SYFRFDPLI	9	1	140.42	2178.16	0.6	9.2	0	0	0	0	0.586	NoExpr	Pending	1	188415854	188415855	188415855	C	T	missense_variant
14-34343681-34343682-G-A		1		Glud1	V546I	1	AYVNAIEKI	9	1	141.15	398.24	0.4	1.4	251.58	0	0	0	0.777	NoExpr	Pending	14	34343681	34343682	34343682	G	A	missense_variant
10-81362244-81362245-G-C		1	3	Mfsd12	G320A	1	SAFFSSFLM	2	4	143.88	163.305	0.5	0.5	14.069	0	0	0	0.098	NoExpr	Pending	10	81362244	81362245	81362245	G	C	missense_variant
17-35471049-35471050-G-C		1		H2-Q10	E190Q	1	AYLEAECVQWL	9	1	145.05	271.686	0.3	0.6	11.753	0	0	0	0.063	NoExpr	Pending	17	35471049	35471050	35471050	G	C	missense_variant
2-85976087-85976088-A-C		2	1	Olfr1029	S282R	1	RPLLNPLIYSL	1	3	145.52	158.68	0.1	0.2	0	0	0	0	0.252	NoExpr	Pending	2	85976087	85976088	85976088	A	C	missense_variant
2-88682892-88682893-G-T		1	2	Olfr1195	L280I	1	FINPLIYTL	2	3	145.88	220.206	0.7	0.8	0	0	0	0	0.197	NoExpr	Pending	2	88682892	88682893	88682893	G	T	missense_variant
17-37624365-37624366-C-T		3		Olfr116	G90S	1	SSLAGSSYI	7	3	145.91	388.269	0.39	1.2	0	0	0	0	0.016	NoExpr	Pending	17	37624365	37624366	37624366	C	T	missense_variant
7-21350126-21350127-T-C		2	1	Vmn1r128	F252S	1	SYLLNCICI	1	3	146.63	363.153	0.6	0.7	0	0	0	0	0.14	NoExpr	Pending	7	21350126	21350127	21350127	T	C	missense_variant
2-88412285-88412286-G-A		2		Olfr1180	P124L	1	RYVAICRLL	8	2	148	59.09	0.5	0.4	0.103	0	0	0	0.343	NoExpr	Pending	2	88412285	88412286	88412286	G	A	missense_variant
7-103565110-103565111-C-A		1	1	Olfr616	S56I	1	ILHEPQYIF	1	2	148.23	138.909	0.8	0.8	0	0	0	0	0.29	NoExpr	Pending	7	103565110	103565111	103565111	C	A	missense_variant

ID	H-2-Dd	H-2-Kd	H-2-Ld	Gene	AA Change	Num Passing Transcripts	Best Peptide	Pos	Num Passing Peptides	IC50 MT	IC50 WT	%ile MT	%ile WT	RNA Expr	RNA VAF	Allele Expr	RNA Depth	DNA VAF	Tier	Evaluation	CHROM	START	END	POS	REF_y	ALT_y	biotype
X-74303872-74303874-GG-TT	1	1	2	Atp6ap1	W420F	1	IFMGLLTTL	2	4	148.62	239.04	0.4	0.5	194.82	0	0	0	0.037	NoExpr	Pending	X	74303872	74303874	74303873	GG	TT	missense_variant
19-11472482-11472483-T-A		4	2	Ms4a6c	S80T	1	YFNSVFTVL	7	6	149.03	95.556	0.4	0.3	78.803	0	0	0	0.034	NoExpr	Pending	19	11472482	11472483	11472483	T	A	missense_variant
5-103529659-103529660-T-C		1	2	Ptpn13	S715P	1	PYFRLEHYL	1	3	151.04	18.96	0.4	0.2	29.29	0	0	0	0.255	NoExpr	Pending	5	103529659	103529660	103529660	T	C	missense_variant
12-115847938-115847939-G-A		2		Ighv1-76	A98V	1	VYMQLSSL	1	2	152.31	100.95	0.22	0.14	0	0	0	0	0.058	NoExpr	Pending	12	115847938	115847939	115847939	G	A	missense_variant
7-14491915-14491917-CC-GT		3	1	Sult2a7	W33Y	1	SYPKSGMTYL	9	4	153.12	276.91	0.3	0.5	0	0	0	0	0.075	NoExpr	Pending	7	14491915	14491917	14491916	CC	GT	missense_variant
4-147613774-147613775-G-A		1		Zfp979	T159I	1	NRLKVSSII	8	1	153.14	62.964	0.6	0.3	16.978	0	0	0	0.284	NoExpr	Pending	4	147613774	147613775	147613775	G	A	missense_variant
5-146526216-146526217-G-A		3	1	Gm3404	V70M	1	EYMVQMIHYI	3	4	154	427.54	0.2	0.45	0	0	0	0	0.048	NoExpr	Pending	5	146526216	146526217	146526217	G	A	missense_variant
5-146491780-146491781-G-A		3	1	Gm6370	V70M	1	EYMVQMIHYI	3	4	154	427.54	0.2	0.45	0	0	0	0	0.063	NoExpr	Pending	5	146491780	146491781	146491781	G	A	missense_variant
8-15028516-15028517-G-C		1		Kbtbd11	G372A	1	NYLFLAGAV	8	1	154.62	436.606	0.4	1.1	1.333	0	0	0	0.237	NoExpr	Pending	8	15028516	15028517	15028517	G	C	missense_variant
8-93076192-93076193-C-T		2		Ces1b	D90N	1	SYPPMCSQNAV	9	2	154.78	196.38	0.3	0.4	0	0	0	0	0.176	NoExpr	Pending	8	93076192	93076193	93076193	C	T	missense_variant
4-147390493-147390494-C-T		1		Zfp978	T166I	1	SIISLNQGI	9	1	154.78	2169.05	0.73	10	3.874	0	0	0	0.072	NoExpr	Pending	4	147390493	147390494	147390494	C	T	missense_variant
4-146466247-146466248-C-T		2		Zfp992	T142I	1	SIISLNQGI	2	2	154.78	254.608	0.73	0.79	1.267	0	0	0	0.234	NoExpr	Pending	4	146466247	146466248	146466248	C	T	missense_variant
11-58529905-58529906-G-A		2		Olfr330	H27Y	1	KYSALLAVV	2	2	156.47	2496.64	0.58	6.4	0	0	0	0	0.034	NoExpr	Pending	11	58529905	58529906	58529906	G	A	missense_variant
12-37108954-37108955-C-T		3		Meox2	S42F	1	LFTSSSSCI	2	3	157.43	719.598	0.4	2.2	1.206	0	0	0	0.187	NoExpr	Pending	12	37108954	37108955	37108955	C	T	missense_variant
11-71122828-71122829-T-A		2		Nlrp1a	N532Y	1	EYQDMELLTHV	2	2	157.67	4526.88	0.23	5.1	0.059	0	0	0	0.079	NoExpr	Pending	11	71122828	71122829	71122829	T	A	missense_variant
3-102898878-102898879-T-G		1		Sycp1	M499L	1	HYLKQVEEL	9	1	157.78	279.12	0.4	0.48	1.739	0	0	0	0.295	NoExpr	Pending	3	102898878	102898879	102898879	T	G	missense_variant
2-5891296-5891297-C-T		1	2	Sec61a2	C45Y	1	YCQIPLFGI	1	3	158.13	325.837	0.9	1.5	17.83	0	0	0	0.412	NoExpr	Pending	2	5891296	5891297	5891297	C	T	missense_variant
9-109326626-109326627-C-T		2	1	Fbxw28	C359Y	1	IVYSTRFSL	3	3	158.17	194.267	0.7	0.92	0.028	0	0	0	0.12	NoExpr	Pending	9	109326626	109326627	109326627	C	T	missense_variant
11-71217979-71217980-G-A		2		Nlrp1b	H232Y	1	LYWSQRQPV	2	2	159.98	1866.26	0.3	3.5	2.359	0	0	0	0.143	NoExpr	Pending	11	71217979	71217980	71217980	G	A	missense_variant
7-38099525-38099526-T-G		2		Ccne1	D259A	1	AYVNATGEV	5	2	160.36	1365.99	0.5	2.5	24.318	0	0	0	0.058	NoExpr	Pending	7	38099525	38099526	38099526	T	G	missense_variant
2-10579224-10579225-G-A		2		Sfmbt2	A831T	1	AYQTDTSATQV	9	2	161.63	239.74	0.2	0.2	1.798	0	0	0	0.051	NoExpr	Pending	2	10579224	10579225	10579225	G	A	missense_variant
3-152380543-152380544-A-G		3	1	Usp33	N648S	1	SLNNLWYEF	1	4	161.75	194.908	0.7	0.7	31.385	0	0	0	0.071	NoExpr	Pending	3	152380543	152380544	152380544	A	G	missense_variant
7-85955962-85955963-T-C		1		Vmn2r74	I492M	1	MYMSKEMI	1	1	162.65	247.55	0.24	0.2	0	0	0	0	0.031	NoExpr	Pending	7	85955962	85955963	85955963	T	C	missense_variant
12-115604157-115604158-C-T		1	1	Ighv1-67	G45S	1	SYTFTDYAM	1	2	162.96	567.884	0.5	1.6	0	0	0	0	0.045	NoExpr	Pending	12	115604157	115604158	115604158	C	T	missense_variant
16-58872883-58872885-CT-TC		1		Olfr177	VD88-89VN	1	NFFSVNRRI	6	1	166.38	205.712	0.5	0.63	0	0	0	0	0.018	NoExpr	Pending	16	58872883	58872885	58872884	CT	TC	missense_variant
16-58916374-58916376-CT-TC		1		Olfr180	VD88-89VN	1	NFFSVNRRI	6	1	166.38	205.712	0.5	0.63	0	0	0	0	0.022	NoExpr	Pending	16	58916374	58916376	58916375	CT	TC	missense_variant
9-3001382-3001383-T-A		3	3	Gm10722	F153Y	1	FSFSMIYSF	7	6	167.11	100.462	0.7	0.4	0.048	0	0	0	0.04	NoExpr	Pending	9	3001382	3001383	3001383	T	A	missense_variant
1-171531183-171531185-GC-AA		1	1	Itln1	G138V	1	YKNPVYFDI	5	1	167.11	182.39	0.74	0.8	0.19	0	0	0	0.03	NoExpr	Pending	1	171531183	171531185	171531184	GC	AA,AC	missense_variant
10-22371417-22371418-C-T		1	2	Raet1d	T131I	1	IYPQSQGQI	9	3	168.69	2767.52	0.4	7.1	1.442	0	0	0	0.272	NoExpr	Pending	10	22371417	22371418	22371418	C	T	missense_variant
10-22181175-22181176-C-T		1	2	Raet1e	T133I	1	IYPQSQGQI	9	3	168.69	2767.52	0.4	7.1	0.87	0	0	0	0.175	NoExpr	Pending	10	22181175	22181176	22181176	C	T	missense_variant
2-131561347-131561348-A-G		2		Adra1d	V274A	1	VYVAARSTT	4	2	169.47	313.394	0.4	0.4	0	0	0	0	0.055	NoExpr	Pending	2	131561347	131561348	131561348	A	G	missense_variant
7-28814083-28814084-T-G		1		Hnrnpl	I203R	1	IYSRTTDVL	4	1	169.83	367.18	0.4	0.8	242.46	0	0	0	0.162	NoExpr	Pending	7	28814083	28814084	28814084	T	G	missense_variant
5-146170326-146170327-T-C		3	1	Gm6309	I70V	1	YVHQMIHYI	2	3	169.86	138.385	0.5	0.4	0	0	0	0	0.032	NoExpr	Pending	5	146170326	146170327	146170327	T	C	missense_variant
17-35380588-35380590-CC-GG		1		H2-Q4	S216W	1	AYLEGACVQWL	10	1	170.61	128.777	0.21	0.2	256.73	0	0	0	0.017	NoExpr	Pending	17	35380588	35380590	35380589	CC	GG	missense_variant
4-123917596-123917597-T-G		1	1	Rragc	F22V	1	SYGAADSVP	8	2	170.64	254.736	0.7	0.7	63.394	0	0	0	0.556	NoExpr	Pending	4	123917596	123917597	123917597	T	G	missense_variant
3-82039854-82039855-G-A		1		Gucy1b1	T349I	1	SDIPLHDAI	9	1	170.71	2939.41	0.6	8.8	0.807	0	0	0	0.298	NoExpr	Pending	3	82039854	82039855	82039855	G	A	missense_variant
8-21326914-21326915-G-A		1		Defa29	P21S	1	AFQVQADSI	8	1	171.04	353.256	0.5	0.7	0	0	0	0	0.045	NoExpr	Pending	8	21326914	21326915	21326915	G	A	missense_variant
12-114682674-114682675-C-T		3		Ighv1-18	R103H	1	AYMELHSLTSE	6	3	171.28	521.86	0.3	0.72	0	0	0	0	0.03	NoExpr	Pending	12	114682674	114682675	114682675	C	T	missense_variant
12-114708691-114708692-T-G		3		Ighv1-19	N103H	1	AYMELHSLTSE	6	3	171.28	954.06	0.3	1.3	0	0	0	0	0.041	NoExpr	Pending	12	114708691	114708692	114708692	T	G	missense_variant
12-115461046-115461047-C-G		3		Ighv1-62-3	Q101H	1	AYMHLSSL	4	3	171.32	100.95	0.25	0.14	0	0	0	0	0.09	NoExpr	Pending	12	115461046	115461047	115461047	C	G	missense_variant
15-47838487-47838488-A-G		1	1	Csmd3	L1733P	1	GYVPQGYSTL	4	2	172.43	140.78	0.25	0.2	0.041	0	0	0	0.157	NoExpr	Pending	15	47838487	47838488	47838488	A	G	missense_variant
7-80513276-80513277-G-A		1		Blm	P109S	1	KGTCSESSL	7	1	172.47	1391.91	0.8	6.7	10.083	0	0	0	0.227	NoExpr	Pending	7	80513276	80513277	80513277	G	A	missense_variant
9-123828645-123828646-C-G		2	1	Fyco1	A822P	1	KPQKQEQEL	2	2	172.47	1828.1	0.4	5.4	6.528	0	0	0	0.077	NoExpr	Pending	9	123828645	123828646	123828646	C	G	missense_variant
9-38473292-38473293-T-A		1		Olfr905	L182H	1	HYFCDIHPL	7	1	173.63	491.4	0.4	0.9	0	0	0	0	0.019	NoExpr	Pending	9	38473292	38473293	38473293	T	A	missense_variant
6-52729333-52729334-C-T		1		Tax1bp1	H107Y	1	CYVTYKGEI	5	1	174.35	104.847	0.8	0.4	144.71	0	0	0	0.186	NoExpr	Pending	6	52729333	52729334	52729334	C	T	missense_variant
5-7978488-7978489-T-G		1	2	Steap4	F356V	1	LGILGVFLF	6	3	174.92	144.82	0.8	0.6	1.978	0	0	0	0.156	NoExpr	Pending	5	7978488	7978489	7978489	T	G	missense_variant
17-37590083-37590084-C-T		3		Olfr114	G90S	1	SYISYGQCM	1	3	176.64	597.06	0.5	0.96	0	0	0	0	0.026	NoExpr	Pending	17	37590083	37590084	37590084	C	T	missense_variant
6-89747328-89747329-C-T		3	1	Vmn1r41	T284I	1	SYAIVSPFV	4	4	176.97	79.138	0.5	0.4	0	0	0	0	0.16	NoExpr	Pending	6	89747328	89747329	89747329	C	T	missense_variant
4-147755719-147755720-G-A		1	2	Zfp984	L225F	1	FTHQIHFSI	7	3	179.47	4786.3	0.8	7.2	20.836	0	0	0	0.076	NoExpr	Pending	4	147755719	147755720	147755720	G	A	missense_variant
9-32259654-32259655-C-T		2	1	Arhgap32	P1593S	1	LHAPSPSMI	5	3	180	594.415	0.4	0.95	0.021	0	0	0	0.496	NoExpr	Pending	9	32259654	32259655	32259655	C	T	missense_variant
X-155053804-155053805-A-T		2		Magea5	F197Y	1	IYMKGNYV	2	2	180.44	2689.06	0.3	3.5	0.07	0	0	0	0.323	NoExpr	Pending	X	155053804	155053805	155053805	A	T	missense_variant
7-47589881-47589882-C-T		1	1	Mrgpra3	D99N	1	TVNLLKFTL	3	2	181.06	1061.7	0.7	3.4	0.124	0	0	0	0.192	NoExpr	Pending	7	47589881	47589882	47589882	C	T	missense_variant
7-20883347-20883348-A-C		3	1	Vmn1r117	I258M	1	FYLLNCMCI	7	4	182.43	363.153	0.4	0.7	0	0	0	0	0.049	NoExpr	Pending	7	20883347	20883348	20883348	A	C	missense_variant
16-32754550-32754551-C-T		2		Muc4	S1475L	1	SHAPNMSSL	9	2	182.85	1222.43	0.6	3.1	0.047	0	0	0	0.069	NoExpr	Pending	16	32754550	32754551	32754551	C	T	missense_variant
13-52968117-52968118-C-G		5		Nfil3	S250T	1	SYMGTSFST	5	5	184.04	139.666	0.5	0.4	21.339	0	0	0	0.04	NoExpr	Pending	13	52968117	52968118	52968118	C	G	missense_variant
5-128797656-128797657-C-T		1		Rimbp2	G297E	1	SPTQVDSEI	8	1	186.25	274.641	0.6	1.3	0	0	0	0	0.038	NoExpr	Pending	5	128797656	128797657	128797657	C	T	missense_variant
16-43969362-43969363-C-T		1	2	Zdhhc23	G354D	1	ADMAYIFLI	2	3	188.73	209.329	0.8	0.8	0.129	0	0	0	0.288	NoExpr	Pending	16	43969362	43969363	43969363	C	T	missense_variant
13-3564569-3564570-A-T		2	1	Gdi2	H341L	1	IYVCMISFAL	10	3	190.51	4902.98	0.3	5.9	314.35	0	0	0	0.044	NoExpr	Pending	13	3564569	3564570	3564570	A	T	missense_variant
19-45741560-45741561-T-G		2		Fgf8	K116T	1	LYSRTSGTHV	8	2	190.63	747.55	0.2	0.6	0	0	0	0	0.062	NoExpr	Pending	19	45741560	45741561	45741561	T	G	missense_variant
14-46384000-46384001-T-G		1		Bmp4	N362T	1	LVTSVNSSI	3	1	191.47	225.99	0.6	0.7	0.401	0	0	0	0.074	NoExpr	Pending	14	46384000	46384001	46384001	T	G	missense_variant
7-3842299-3842300-A-G		1		Pira2	Y320H	1	HYEPRLSVL	1	1	192.79	51.179	0.4	0.2	8.62	0	0	0	0.065	NoExpr	Pending	7	3842299	3842300	3842300	A	G	missense_variant
7-104411593-104411594-C-T		3		Trim30a	S325N	1	NYNTVPVSEI	1	3	192.8	42.92	0.2	0.2	42.328	0	0	0	0.506	NoExpr	Pending	7	104411593	104411594	104411594	C	T	missense_variant

ID	H-2-Dd	H-2-Kd	H-2-Ld	Gene	AA Change	Num Passing Transcripts	Best Peptide	Pos	Num Passing Peptides	IC50 MT	IC50 WT	%ile MT	%ile WT	RNA Expr	RNA VAF	Allele Expr	RNA Depth	DNA VAF	Tier	Evaluation	CHROM	START	END	POS	REF_y	ALT_y	biotype
11-71124082-71124083-G-A		1		Nlrp1a	H114Y	1	AYMIDIQDL	2	1	193.76	3184.86	0.6	8	0.059	0	0	0	0.059	NoExpr	Pending	11	71124082	71124083	71124083	G	A	missense_variant
4-147755956-147755957-C-T		1		Zfp984	G146R	1	STISLNQRI	8	1	194.27	254.608	0.79	0.79	20.836	0	0	0	0.022	NoExpr	Pending	4	147755956	147755957	147755957	C	T	missense_variant
2-30113327-30113328-C-A		1	1	Zer1	G26C	1	CYLLDKETL	1	2	196.2	275.86	0.8	1	13.685	0	0	0	0.07	NoExpr	Pending	2	30113327	30113328	30113328	C	A	missense_variant
5-124130480-124130481-A-T		1		Pitpnm2	F548Y	1	TYNGQVCLI	2	1	196.38	2150.63	0.6	4.9	7.624	0	0	0	0.15	NoExpr	Pending	5	124130480	124130481	124130481	A	T	missense_variant
2-103566901-103566902-G-C		1		Abtb2	G59A	1	CYSASMNSR	4	1	196.38	274.322	0.64	1.1	0.551	0	0	0	0.203	NoExpr	Pending	2	103566901	103566902	103566902	G	C	missense_variant
13-21674751-21674752-G-T		2	2	Olfr1360	T64N	1	TPMYFFLNNL	8	4	196.38	180.097	0.3	0.3	0	0	0	0	0.271	NoExpr	Pending	13	21674751	21674752	21674752	G	T	missense_variant
1-85259729-85259731-TC-CT		1		C130026I21Rik	D111S	1	AYPSLKETL	4	1	197.39	565.98	0.6	1.2	108.69	0	0	0	0.017	NoExpr	Pending	1	85259729	85259731	85259730	TC	CT	missense_variant
6-121784364-121784366-AG-CA		1	1	Gm7298	QV1293-1294HM	1	GSFSQKFHM	8	2	198.15	4731.51	1	7.7	0	0	0	0	0.063	NoExpr	Pending	6	121784364	121784366	121784365	AG	CA	missense_variant
9-39237880-39237881-A-C		1		Olfr1537	L184R	1	HYFCDRLPL	6	1	199.28	395.16	0.8	0.9	0	0	0	0	0.031	NoExpr	Pending	9	39237880	39237881	39237881	A	C	missense_variant
7-4834243-4834244-T-G		4		Shisa7	K274T	1	LYNTMTPSNL	6	4	199.77	336.38	0.23	0.3	0	0	0	0	0.208	NoExpr	Pending	7	4834243	4834244	4834244	T	G	missense_variant
7-7394422-7394424-TG-CT		2	1	Vmn2r31	IN278-279ID	1	IYEETIDFI	7	3	200.03	119.43	0.46	0.2	0	0	0	0	0.081	NoExpr	Pending	7	7394422	7394424	7394423	TG	CT	missense_variant
2-182000505-182000506-G-C		1	1	Gm14496	V657L	1	ILQQTTFGL	9	2	200.68	1199.5	0.99	4.1	0	0	0	0	0.021	NoExpr	Pending	2	182000505	182000506	182000506	G	C	missense_variant
4-47049323-47049324-G-A		1		Anks6	H194Y	1	GYEAVVRLL	2	1	201.96	3571.87	0.6	7.3	1.374	0	0	0	0.547	NoExpr	Pending	4	47049323	47049324	47049324	G	A	missense_variant
7-11880408-11880409-C-T		1		Vmn1r75	L23F	1	LGIFGNSSI	4	1	202.81	201.878	0.6	0.6	0	0	0	0	0.169	NoExpr	Pending	7	11880408	11880409	11880409	C	T	missense_variant
8-108947744-108947745-G-A		2	2	Zfhx3	S1809N	1	IPNAEFQL	3	4	204.3	371.823	0.2	0.4	2.282	0	0	0	0.28	NoExpr	Pending	8	108947744	108947745	108947745	G	A	missense_variant
17-20029552-20029553-T-C		2	1	Vmn2r104	S819G	1	SILASGTAL	6	3	204.4	149.31	0.5	0.4	0	0	0	0	0.472	NoExpr	Pending	17	20029552	20029553	20029553	T	C	missense_variant
13-21132993-21132994-T-A		3		Olfr263	F73Y	1	CYTTSIVPQML	2	3	204.5	1562.1	0.3	3.6	0	0	0	0	0.049	NoExpr	Pending	13	21132993	21132994	21132994	T	A	missense_variant
3-36076046-36076047-G-A		3		Acad9	G213D	1	KYFILNDSK	7	3	205.71	138.909	0.8	0.7	31.057	0	0	0	0.291	NoExpr	Pending	3	36076046	36076047	36076047	G	A	missense_variant
16-58824442-58824443-T-C		1		Olfr175	N89D	1	NFFSVDRRI	6	1	205.71	166.376	0.63	0.5	0	0	0	0	0.082	NoExpr	Pending	16	58824442	58824443	58824443	T	C	missense_variant
5-48219744-48219745-A-C		1		Slit2	D409A	1	LYANKLQTV	3	1	206.17	2166.44	0.6	3.3	194.32	0	0	0	0.199	NoExpr	Pending	5	48219744	48219745	48219745	A	C	missense_variant
18-44276376-44276377-T-G		2		Npy6r	H288Q	1	WYQEMLMSC	3	2	207.28	343.764	0.5	1	0	0	0	0	0.047	NoExpr	Pending	18	44276376	44276377	44276377	T	G	missense_variant
7-63758015-63758016-C-T		1		Otud7a	T689I	1	AAAAAATAI	9	1	207.3	3289.2	0.69	9.5	0.179	0	0	0	0.107	NoExpr	Pending	7	63758015	63758016	63758016	C	T	missense_variant
2-36859574-36859575-A-C		3		Olfr351	L258V	1	AYVCPPSVV	3	3	207.65	126.29	0.4	0.4	0	0	0	0	0.071	NoExpr	Pending	2	36859574	36859575	36859575	A	C	missense_variant
2-87544979-87544980-G-A		1	1	Olfr1128	S188F	1	ALFCSDTSI	3	2	208.09	369.199	0.6	1.2	0	0	0	0	0.211	NoExpr	Pending	2	87544979	87544980	87544980	G	A	missense_variant
17-19394458-19394459-T-G		1		Vmn2r99	S814A	1	SILAASTAL	5	1	208.57	149.31	0.6	0.4	0	0	0	0	0.019	NoExpr	Pending	17	19394458	19394459	19394459	T	G	missense_variant
1-173130892-173130893-C-G		1	2	Olfr1408	G108A	1	FAITNCFLL	2	2	213.22	217.185	0.61	0.6	0	0	0	0	0.022	NoExpr	Pending	1	173130892	173130893	173130893	C	G	missense_variant
16-32753808-32753809-T-G		2	1	Muc4	I1228M	1	TSIQMLSTL	5	2	216.32	826.209	0.5	2.1	0.047	0	0	0	0.009	NoExpr	Pending	16	32753808	32753809	32753809	T	G	missense_variant
9-89603089-89603090-C-G		1	2	Minar1	A85P	1	PDIVTIFNL	1	3	216.47	206.933	1.2	0.9	0.011	0	0	0	0.135	NoExpr	Pending	9	89603089	89603090	89603090	C	G	missense_variant
10-128408556-128408557-C-T		1		Nabp2	G90S	1	LYTGRGSDL	7	1	216.82	572.915	0.48	1.7	53.521	0	0	0	0.071	NoExpr	Pending	10	128408556	128408557	128408557	C	T	missense_variant
4-19461711-19461712-G-A		1		Cngb3	R531K	1	LLLKLKSTI	4	1	217.9	379.542	0.6	0.7	0	0	0	0	0.028	NoExpr	Pending	4	19461711	19461712	19461712	G	A	missense_variant
2-91492595-91492596-G-A		1		Lrp4	D1142N	1	VYWTNTGTNR	5	1	218.04	347.68	0.4	0.6	0.365	0	0	0	0.355	NoExpr	Pending	2	91492595	91492596	91492596	G	A	missense_variant
12-113625540-113625542-CT-AC		1		Ighv5-6	K106S	1	LYLQMSSLS	9	1	218.32	174.618	0.58	0.56	0	0	0	0	0.048	NoExpr	Pending	12	113625540	113625542	113625541	CT	AC	missense_variant
7-5125817-5125818-C-T		2		Rasl2-9	E38K	1	KYVATLGVE	1	2	218.82	2618.73	1	6.8	0.401	0	0	0	0.041	NoExpr	Pending	7	5125817	5125818	5125818	C	T	missense_variant
13-34015248-34015249-G-C		4		Ripk1	A195P	1	YYMPPEHLND	4	4	219.05	157.9	0.33	0.23	29.541	0	0	0	0.038	NoExpr	Pending	13	34015248	34015249	34015249	G	C	missense_variant
17-37660141-37660142-T-G		4	1	Olfr117	N64H	1	YYFLTHLSFV	6	5	221.03	183	0.2	0.2	0	0	0	0	0.031	NoExpr	Pending	17	37660141	37660142	37660142	T	G	missense_variant
7-102984444-102984445-G-A		1		Olfr578	L240F	1	AFNTCISHI	2	1	221.2	1042.53	0.6	2.9	0	0	0	0	0.61	NoExpr	Pending	7	102984444	102984445	102984445	G	A	missense_variant
9-3001404-3001406-TG-CA		2	2	Gm10722	HV160-161HI	1	FSFLAIFHI	9	4	221.31	1022.89	0.9	3.2	0.048	0	0	0	0.032	NoExpr	Pending	9	3001404	3001406	3001405	TG	CA,CG	missense_variant
8-45935097-45935098-C-T		1		Ccdc110	L31F	1	SIFLSASKI	3	1	221.36	342.05	0.8	1.2	0	0	0	0	0.046	NoExpr	Pending	8	45935097	45935098	45935098	C	T	missense_variant
1-139733518-139733519-A-C		1		Gm4788	L531V	1	KYVDGEKLSVL	3	1	221.76	232.745	0.3	0.2	0	0	0	0	0.453	NoExpr	Pending	1	139733518	139733519	139733519	A	C	missense_variant
16-58872747-58872748-C-G		4	2	Olfr177	S134T	1	QYHTMMSKK	4	6	222.43	319.956	0.8	0.7	0	0	0	0	0.02	NoExpr	Pending	16	58872747	58872748	58872748	C	G	missense_variant
5-129697820-129697821-G-A		1		Septin14	L97F	1	TYEFLERNI	4	1	222.89	256.012	0.5	0.58	0.144	0	0	0	0.41	NoExpr	Pending	5	129697820	129697821	129697821	G	A	missense_variant
5-139393032-139393033-G-A		1	2	Gpr146	V197M	1	MPGLAVLYAL	1	3	223.07	296.82	0.2	0.4	8.367	0	0	0	0.406	NoExpr	Pending	5	139393032	139393033	139393033	G	A	missense_variant
9-27019335-27019336-C-T		1		Vps26b	G121E	1	SYTEQNVKL	4	1	223.4	166.441	0.7	0.5	13.551	0	0	0	0.271	NoExpr	Pending	9	27019335	27019336	27019336	C	T	missense_variant
3-73049203-73049204-G-A		1	1	Slitrk3	P745L	1	VGHVYEYIL	9	2	223.61	2781.92	0.92	12	0	0	0	0	0.415	NoExpr	Pending	3	73049203	73049204	73049204	G	A	missense_variant
12-116000076-116000078-TC-AA		2		Ighv1-85	E101L	1	AYMLLHSL	4	2	225.17	212.16	0.34	0.31	0	0	0	0	0.033	NoExpr	Pending	12	116000076	116000078	116000077	TC	AA	missense_variant
15-31594239-31594240-C-G		1	1	Cct5	V244L	1	HPQMPKKVL	9	2	226.46	2483.13	1	4.8	549.35	0	0	0	0.021	NoExpr	Pending	15	31594239	31594240	31594240	C	G	missense_variant
15-76537777-76537778-T-C		1		Fbxl6	Q187R	1	SRLQSLTLI	2	1	226.51	274.641	0.7	0.6	34.326	0	0	0	0.205	NoExpr	Pending	15	76537777	76537778	76537778	T	C	missense_variant
17-36168009-36168010-C-T		1		Gm8909	E88K	1	EYWERKTQI	6	1	226.6	298.717	0.4	0.5	8.461	0	0	0	0.023	NoExpr	Pending	17	36168009	36168010	36168010	C	T	missense_variant
17-35380584-35380585-C-G		1		H2-Q4	Q215E	1	AYLEGACVESL	9	1	227.45	128.777	0.5	0.2	256.73	0	0	0	0.029	NoExpr	Pending	17	35380584	35380585	35380585	C	G,A	missense_variant
9-3001256-3001257-T-A		1	1	Gm10722	F111Y	1	FLIYSDFLF	4	2	229	160.262	1	0.7	0.048	0	0	0	0.118	NoExpr	Pending	9	3001256	3001257	3001257	T	A	missense_variant
6-69775920-69775921-T-G		2		Igkv5-45	K59T	1	WYQQTSHES	5	2	229.61	1294.97	0.8	3.6	0	0	0	0	0.213	NoExpr	Pending	6	69775920	69775921	69775921	T	G	missense_variant
7-85955987-85955988-T-C		1		Vmn2r74	E484G	1	GHLPSGQQI	1	1	231.2	671.568	0.5	1.1	0	0	0	0	0.028	NoExpr	Pending	7	85955987	85955988	85955988	T	C	missense_variant
18-68408021-68408022-C-G		2		Mc2r	A67P	1	MYFFICSLPI	9	2	231.3	337.91	0.35	0.43	0	0	0	0	0.19	NoExpr	Pending	18	68408021	68408022	68408022	C	G	missense_variant
5-71641090-71641091-T-C		1	2	Gabra4	R156G	1	FRIMGNGTI	5	3	231.79	558.586	0.7	1.7	0	0	0	0	0.297	NoExpr	Pending	5	71641090	71641091	71641091	T	C	missense_variant
2-86639305-86639306-A-G		2		Olfr1084	V134A	1	LYTAIVSHK	4	2	232.32	429.625	0.8	1.3	0	0	0	0	0.049	NoExpr	Pending	2	86639305	86639306	86639306	A	G	missense_variant
X-20694540-20694541-G-T		1		Cdk16	G172V	1	TYIKLDKLV	9	1	233.39	1362.26	0.5	3.3	48.048	0	0	0	0.053	NoExpr	Pending	X	20694540	20694541	20694541	G	T	missense_variant
7-26611615-26611616-G-T		2	1	Vmn1r185	P155T	1	FIIFVYTFI	7	3	234.47	1059.47	0.7	3	0	0	0	0	0.079	NoExpr	Pending	7	26611615	26611616	26611616	G	T	missense_variant
14-50515313-50515314-A-G		1	2	Olfr742	I37V	1	VTFFVVYIL	5	3	235.96	200.676	1.3	1.1	0	0	0	0	0.063	NoExpr	Pending	14	50515313	50515314	50515314	A	G	missense_variant
4-146466246-146466247-A-T		2		Zfp992	T142S	1	SSISLNQGI	2	2	236.04	254.608	0.69	0.79	1.267	0	0	0	0.509	NoExpr	Pending	4	146466246	146466247	146466247	A	T	missense_variant
6-122082717-122082718-G-A		2		Mug2	A1352T	1	SYTGSRPTS	8	2	238.28	251.868	1	1.2	0	0	0	0	0.382	NoExpr	Pending	6	122082717	122082718	122082718	G	A	missense_variant
5-106892702-106892703-T-A		1	2	Hfm1	H732L	1	LKNPSLYGF	6	3	238.61	249.158	1.4	1.4	0	0	0	0	0.386	NoExpr	Pending	5	106892702	106892703	106892703	T	A	missense_variant
7-42136836-42136837-T-C		1	2	Vmn2r60	F355L	1	LPKLWHLFF	4	3	239.88	237.684	0.6	0.5	0	0	0	0	0.206	NoExpr	Pending	7	42136836	42136837	42136837	T	C	missense_variant

ID	H-2-Dd	H-2-Kd	H-2-Ld	Gene	AA Change	Num Passing Transcripts	Best Peptide	Pos	Num Passing Peptides	IC50 MT	IC50 WT	%ile MT	%ile WT	RNA Expr	RNA VAF	Allele Expr	RNA Depth	DNA VAF	Tier	Evaluation	CHROM	START	END	POS	REF_y	ALT_y	biotype
9-27010422-27010423-G-A		1	1	Vps26b	R269C	1	CYYLNLVLI	1	2	239.93	670.023	0.6	1.6	13.551	0	0	0	0.133	NoExpr	Pending	9	27010422	27010423	27010423	G	A	missense_variant
4-145622177-145622179-AG-GT		1		Gm13212	R62V	1	KYVKVLDQD	3	1	241.6	497.84	1.2	2.2	3.589	0	0	0	0.014	NoExpr	Pending	4	145622177	145622179	145622178	AG	GT	missense_variant
8-15998852-15998853-T-A		1		Csmd1	K2283M	1	MYQCHPGYTLI	1	1	241.68	157.06	0.4	0.23	0.011	0	0	0	0.167	NoExpr	Pending	8	15998852	15998853	15998853	T	A	missense_variant
19-11472464-11472465-T-C		1	3	Ms4a6c	Y74H	1	VPHFNSVFSVL	3	4	241.7	292.71	0.13	0.17	78.803	0	0	0	0.032	NoExpr	Pending	19	11472464	11472465	11472465	T	C	missense_variant
7-103328821-103328822-C-T		1	1	Olfr598	T112I	1	ILQGMESGI	1	2	242.15	338.135	0.7	1	0	0	0	0	0.021	NoExpr	Pending	7	103328821	103328822	103328822	C	T	missense_variant
11-114751822-114751823-C-T		1		Dnaic2	P417S	1	AYLSDGAWSS\	10	1	243.82	209.552	0.5	0.5	6.029	0	0	0	0.559	NoExpr	Pending	11	114751822	114751823	114751823	C	T	missense_variant
4-32707627-32707628-G-C		3		Mdn1	G1639A	1	VYIDAIGSGVT	5	3	243.82	306.024	0.5	0.6	9.587	0	0	0	0.26	NoExpr	Pending	4	32707627	32707628	32707628	G	C	missense_variant
13-62172900-62172901-G-A		1		Zfp808	R648Q	1	KAFSQHSTL	5	1	243.82	598.537	0.6	1.8	5.763	0	0	0	0.273	NoExpr	Pending	13	62172900	62172901	62172901	G	A	missense_variant
4-156334453-156334455-GT-AA		2	1	Vmn2r-ps159	V344K	1	KYPKDISHTI	4	3	246.78	182.056	0.38	0.4	0	0	0	0	0.066	NoExpr	Pending	4	156334453	156334455	156334454	GT	AA	missense_variant
2-84940987-84940988-T-A		2	1	Slc43a3	Y108N	1	IFFNTCATI	4	3	248.25	334.264	0.7	0.8	3.755	0	0	0	0.239	NoExpr	Pending	2	84940987	84940988	84940988	T	A	missense_variant
11-78287788-78287789-T-A		1		2610507B11Rik	F2006I	1	GGISVKEHI	9	1	248.37	5471.29	0.7	14	161	0	0	0	0.25	NoExpr	Pending	11	78287788	78287789	78287789	T	A	missense_variant
14-4558365-4558366-C-T		1		Gm3047	A196V	1	KNICVSSAK	5	1	249.51	392.726	1.1	1.5	0	0	0	0	0.073	NoExpr	Pending	14	4558365	4558366	4558366	C	T	missense_variant
X-74303855-74303856-C-A		1		Atp6ap1	F414L	1	SYASDCAGL	9	1	249.88	1535.54	0.6	4.2	194.82	0	0	0	0.041	NoExpr	Pending	X	74303855	74303856	74303856	C	A	missense_variant
7-9986358-9986359-C-G		2		Vmn2r49	V402L	1	IYNALHAI	5	2	249.98	298.47	0.37	0.27	0	0	0	0	0.023	NoExpr	Pending	7	9986358	9986359	9986359	C	G	missense_variant
19-53635751-53635752-A-C		2		Smc3	D733A	1	KFKASRASI	7	2	250.7	156.84	0.4	0.3	94.663	0	0	0	0.236	NoExpr	Pending	19	53635751	53635752	53635752	A	C	missense_variant
1-85610849-85610850-A-C		1		Sp140	K103Q	1	KVIFSQTHL	6	1	251.24	200.028	0.72	0.69	95.257	0	0	0	0.014	NoExpr	Pending	1	85610849	85610850	85610850	A	C	missense_variant
1-85610871-85610872-C-G		1		Sp140	P110R	1	AYRDLKETL	3	1	253.57	565.98	0.5	1.2	95.257	0	0	0	0.014	NoExpr	Pending	1	85610871	85610872	85610872	C	G	missense_variant
8-124794330-124794331-C-T		1		Trim67	S144L	1	LLCSSSSSI	1	1	253.57	110.676	0.8	0.3	0	0	0	0	0.085	NoExpr	Pending	8	124794330	124794331	124794331	C	T	missense_variant
2-86537987-86537988-A-C		1		Olfr1079	I309S	1	KYALRKTGKSI	10	1	255.88	904.93	0.2	0.33	0	0	0	0	0.089	NoExpr	Pending	2	86537987	86537988	86537988	A	C	missense_variant
6-126101834-126101835-G-A		1		Ntf3	T236I	1	IYVRALTSE	1	1	255.91	357.347	0.7	1.1	0	0	0	0	0.124	NoExpr	Pending	6	126101834	126101835	126101835	G	A	missense_variant
17-20540591-20540592-C-A		2	1	Vmn2r109	L834F	1	APKCYIILF	9	3	256.45	359.749	1.1	1.3	0	0	0	0	0.074	NoExpr	Pending	17	20540591	20540592	20540592	C	A	missense_variant
18-44168135-44168136-T-G		2		Spinkl	K45T	1	MYKSTSECSNI	5	2	258.54	1377.63	0.4	1.7	0	0	0	0	0.582	NoExpr	Pending	18	44168135	44168136	44168136	T	G	missense_variant
10-22371399-22371400-C-T		1	2	Raet1d	P125L	1	YPHLQVTMIYL	11	3	259.48	7421.79	0.15	3.7	1.442	0	0	0	0.266	NoExpr	Pending	10	22371399	22371400	22371400	C	T	missense_variant
10-22181157-22181158-C-T		1	2	Raet1e	P127L	1	YPHLQVTMIYL	11	3	259.48	7421.79	0.15	3.7	0.87	0	0	0	0.187	NoExpr	Pending	10	22181157	22181158	22181158	C	T	missense_variant
3-144916636-144916637-G-A		2		Clca4b	L556F	1	AYFSIPGTAEV	3	2	259.94	224.238	0.6	0.5	0	0	0	0	0.033	NoExpr	Pending	3	144916636	144916637	144916637	G	A	missense_variant
17-38208357-38208358-G-A		2		Olfr135	A38T	1	YPTTMIGNI	4	2	260.18	218.82	1.3	1.1	0	0	0	0	0.142	NoExpr	Pending	17	38208357	38208358	38208358	G	A	missense_variant
3-5412115-5412116-T-G		2	1	Zfhx4	F3239V	1	SYVSPQLPGTV	3	3	260.18	150.77	0.6	0.3	0.032	0	0	0	0.195	NoExpr	Pending	3	5412115	5412116	5412116	T	G	missense_variant
2-146109987-146109988-G-A		2		Cfap61	G797S	1	RYTSTVPSNL	4	2	260.3	304.93	0.4	0.43	0	0	0	0	0.227	NoExpr	Pending	2	146109987	146109988	146109988	G	A	missense_variant
17-23345958-23345959-T-G		2		Vmn2r115	I273M	1	MYGDTDSTLAV	1	2	261.14	268.762	0.6	0.6	0	0	0	0	0.024	NoExpr	Pending	17	23345958	23345959	23345959	T	C,G	missense_variant
17-23386930-23386931-T-G		2		Vmn2r116	I272M	1	MYGDTDSTLAV	1	2	261.14	268.762	0.6	0.6	0	0	0	0	0.04	NoExpr	Pending	17	23386930	23386931	23386931	T	G	missense_variant
17-45568433-45568435-CA-TG		1		Hsp90ab1	M620T	1	GYTMAKKHL	3	1	261.87	80.11	0.5	0.3	2213	0	0	0	0.065	NoExpr	Pending	17	45568433	45568435	45568434	CA	TG	missense_variant
2-167104677-167104678-C-T		1		Kcnb1	G750E	1	FEAEVHQYI	4	1	262.48	189.71	0.7	0.6	0.018	0	0	0	0.452	NoExpr	Pending	2	167104677	167104678	167104678	C	T	missense_variant
13-59474612-59474613-C-G		1		Agtpbp1	S933T	1	SNTPTAQSL	3	1	263.01	323.035	1.3	1.6	20.342	0	0	0	0.121	NoExpr	Pending	13	59474612	59474613	59474613	C	G	missense_variant
13-116303260-116303261-C-T		1		Isl1	V184M	1	KTTRMRTVL	5	1	263.01	388.269	1.3	2	0	0	0	0	0.271	NoExpr	Pending	13	116303260	116303261	116303261	C	T	missense_variant
1-171699598-171699599-C-T		2		Cd48	P198S	1	FYTCQVSNS	9	2	263.08	371.612	0.7	1.1	13.942	0	0	0	0.042	NoExpr	Pending	1	171699598	171699599	171699599	C	T	missense_variant
17-37299758-37299759-G-A		3	1	Olfr101	T221I	1	SYFYIIINL	7	3	264.4	38.823	0.6	0.2	0	0	0	0	0.028	NoExpr	Pending	17	37299758	37299759	37299759	G	A	missense_variant
2-89816453-89816454-A-G		4		Olfr1255	I43V	1	LYMITVSGNL	6	4	264.53	295.57	0.27	0.45	0	0	0	0	0.439	NoExpr	Pending	2	89816453	89816454	89816454	A	G	missense_variant
17-35471053-35471054-G-C		1		H2-Q10	W191S	1	AYLEAECVESL	10	1	265.39	271.686	0.4	0.6	11.753	0	0	0	0.063	NoExpr	Pending	17	35471053	35471054	35471054	G	C	missense_variant
17-80216999-80217000-C-G		3	1	Ttc39d	L363V	1	EVMWCHIFL	2	4	265.46	454.988	1.4	2.1	0.009	0	0	0	0.153	NoExpr	Pending	17	80216999	80217000	80217000	C	G	missense_variant
17-19811942-19811943-A-G		1	1	Vmn2r103	T660A	1	TTFAVTFAM	8	2	265.87	238.605	0.8	0.8	0	0	0	0	0.436	NoExpr	Pending	17	19811942	19811943	19811943	A	G	missense_variant
4-88827848-88827849-C-A		3		Ifna6	T145K	1	KYFHRITVYL	1	3	266.59	577.28	0.41	0.77	0	0	0	0	0.056	NoExpr	Pending	4	88827848	88827849	88827849	C	A	missense_variant
17-37590033-37590034-C-A		1	1	Olfr114	W106C	1	CFAWGEMAI	1	2	267.97	350.825	0.8	0.95	0	0	0	0	0.015	NoExpr	Pending	17	37590033	37590034	37590034	C	A	missense_variant
7-7205574-7205575-T-C		1		Zfp772	E90G	1	SGIHGMTQL	5	1	267.97	867.481	1.1	3.3	4.937	0	0	0	0.034	NoExpr	Pending	7	7205574	7205575	7205575	T	C	missense_variant
17-35471086-35471087-C-T		3		H2-Q10	T202M	1	RYLELGKEMLL	9	3	268.76	143.492	0.4	0.2	11.753	0	0	0	0.026	NoExpr	Pending	17	35471086	35471087	35471087	C	T	missense_variant
7-26611417-26611418-G-A		2		Vmn1r185	H221Y	1	QYIHSTHV	2	2	269.08	5004.2	0.37	3.5	0	0	0	0	0.068	NoExpr	Pending	7	26611417	26611418	26611418	G	A	missense_variant
12-115765432-115765433-C-T		3		Ighv8-13	R68Q	1	HYNPSLKSQL	9	3	269.14	460.45	0.24	0.36	0	0	0	0	0.049	NoExpr	Pending	12	115765432	115765433	115765433	C	T	missense_variant
1-84964237-84964238-T-G		1		AC167036.1	F190L	1	SLALLLSVI	4	1	271.08	272.327	0.8	0.8	0	0	0	0	0.021	NoExpr	Pending	1	84964237	84964238	84964238	T	G	missense_variant
9-37711664-37711666-TG-CA		2	1	Olfr160	FN204-205FD	1	VFFLAGFDI	8	3	271.69	223.607	1.3	0.9	0	0	0	0	0.027	NoExpr	Pending	9	37711664	37711666	37711665	TG	CA	missense_variant
6-57137805-57137806-A-C		2		Vmn1r11	S152R	1	AYTNVRETN	6	2	272.33	319.22	0.8	0.9	0	0	0	0	0.653	NoExpr	Pending	6	57137805	57137806	57137806	A	C	missense_variant
2-76892550-76892551-T-C		1		Ttn	K5680R	1	KYICQVRND	7	1	273.69	507.294	0.8	1.6	0.231	0	0	0	0.193	NoExpr	Pending	2	76892550	76892551	76892551	T	C	missense_variant
14-39472102-39472103-G-A		2	1	Nrg3	S233F	1	AYATSFYL	6	3	274.3	84.09	0.42	0.2	0	0	0	0	0.657	NoExpr	Pending	14	39472102	39472103	39472103	G	A	missense_variant
9-15352941-15352942-C-T		1		Cep295	S136N	1	LYLANLRHM	5	1	275.48	94.643	0.8	0.3	9.282	0	0	0	0.417	NoExpr	Pending	9	15352941	15352942	15352942	C	T	missense_variant
5-71623936-71623937-G-A		1		Gabra4	A351V	1	NYFTNIQMQKV	11	1	277.63	1133.26	0.2	1.5	0	0	0	0	0.258	NoExpr	Pending	5	71623936	71623937	71623937	G	A	missense_variant
7-20811666-20811668-AC-TG		1		Vmn1r114	KF173-174NI	1	NNTDSKSNI	8	1	278.03	10497.6	0.8	21	0	0	0	0	0.122	NoExpr	Pending	7	20811666	20811668	20811667	AC	TG	missense_variant
11-46577224-46577225-C-T		1	1	BC053393	S42F	1	SYPEFRILSFV	5	2	278.3	433.81	0.4	0.61	0	0	0	0	0.507	NoExpr	Pending	11	46577224	46577225	46577225	C	T	missense_variant
17-24157983-24157984-T-G		1	3	Amdhd2	M266L	1	FYGLIADGI	4	4	278.88	246.088	0.69	0.5	23.26	0	0	0	0.043	NoExpr	Pending	17	24157983	24157984	24157984	T	G	missense_variant
16-87437318-87437319-C-T		1		Rwdd2b	S61N	1	VYFTINVNL	8	1	280.02	155.51	0.8	0.6	7.614	0	0	0	0.507	NoExpr	Pending	16	87437318	87437319	87437319	C	T	missense_variant
7-8908508-8908509-A-G		1		Vmn2r40	F595S	1	SCFSAFTAL	1	1	280.71	700.261	0.8	1.6	0	0	0	0	0.047	NoExpr	Pending	7	8908508	8908509	8908509	A	G	missense_variant
11-57289373-57289374-C-T		5	1	Gria1	L717F	1	YFLESTMNEYI	2	6	280.93	2795.74	0.3	1.4	0.075	0	0	0	0.448	NoExpr	Pending	11	57289373	57289374	57289374	C	T	missense_variant
3-90056567-90056568-C-G		1		4933434E20Rik	A109G	1	YRMKALDGI	8	1	281.36	117.56	0.8	0.4	55.792	0	0	0	0.172	NoExpr	Pending	3	90056567	90056568	90056568	C	G	missense_variant
4-147678312-147678313-C-A		2	1	Zfp534	D21Y	1	KDVALYFSL	6	3	281.73	381.795	0.69	1.6	0.011	0	0	0	0.353	NoExpr	Pending	4	147678312	147678313	147678313	C	A	missense_variant
5-121426748-121426749-G-T		1		Naa25	A551S	1	GYLLTRYASSL	9	1	281.83	711.017	0.3	1.3	20.151	0	0	0	0.043	NoExpr	Pending	5	121426748	121426749	121426749	G	T	missense_variant

ID	H-2-Dd	H-2-Kd	H-2-Ld	Gene	AA Change	Num Passing Transcripts	Best Peptide	Pos	Num Passing Peptides	IC50 MT	IC50 WT	%ile MT	%ile WT	RNA Expr	RNA VAF	Allele Expr	RNA Depth	DNA VAF	Tier	Evaluation	CHROM	START	END	POS	REF_y	ALT_y	biotype
2-25906707-25906709-AA-TT		2		Kcnt1	K780L	1	AYGFLNKLI	5	2	282.21	1738.84	0.6	3.4	0	0	0	0	0.174	NoExpr	Pending	2	25906707	25906709	25906708	AA	TT	missense_variant
1-12872308-12872309-A-C		1	1	Slco5a1	I704M	1	AYIPTPMYF	7	2	282.66	450.049	0.6	1.2	0.031	0	0	0	0.25	NoExpr	Pending	1	12872308	12872309	12872309	A	C	missense_variant
11-119436165-119436166-T-G		1		Rnf213	F1661V	1	VYLNFYTAEQL	1	1	283.7	174.345	0.21	0.2	22.933	0	0	0	0.25	NoExpr	Pending	11	119436165	119436166	119436166	T	G	missense_variant
4-147390321-147390322-A-G		1		Zfp978	I109V	1	KCNESSNVI	8	1	284.62	347.608	0.8	1.2	3.874	0	0	0	0.295	NoExpr	Pending	4	147390321	147390322	147390322	A	G	missense_variant
9-45450582-45450583-G-A		1		Dscaml1	V214I	1	EYISVVSWE	3	1	286.48	1172.44	0.8	2.9	0	0	0	0	0.243	NoExpr	Pending	9	45450582	45450583	45450583	G	A	missense_variant
7-26611994-26611995-A-C		1	1	Vmn1r185	N28K	1	KFSLMFHYL	1	2	286.59	698.651	0.7	1.9	0	0	0	0	0.056	NoExpr	Pending	7	26611994	26611995	26611995	A	C	missense_variant
9-38378165-38378166-A-G		1		Olfr251	Q95R	1	SFVTERNTI	6	1	286.79	363.864	0.9	1.2	0	0	0	0	0.151	NoExpr	Pending	9	38378165	38378166	38378166	A	G	missense_variant
10-77548638-77548639-G-A		1	1	Itgb2	C198Y	1	KAYQPPFAF	3	2	288.29	207.297	1	1.2	67.846	0	0	0	0.286	NoExpr	Pending	10	77548638	77548639	77548639	G	A	missense_variant
2-85400458-85400459-G-A		2	1	Olfr992	H25Y	1	GQNESWYIL	7	3	288.96	1383.03	1.2	3.7	0	0	0	0	0.504	NoExpr	Pending	2	85400458	85400459	85400459	G	A	missense_variant
5-138248784-138248785-G-A		4		Nxpe5	D318N	1	YYQNRWHST	4	4	289.13	367.358	0.8	1.1	26.163	0	0	0	0.039	NoExpr	Pending	5	138248784	138248785	138248785	G	A	missense_variant
11-73187746-73187747-G-A		1	1	Ctns	P184L	1	LYIQEEFLL	1	1	289.91	864.682	1.6	3.9	12.251	0	0	0	0.48	NoExpr	Pending	11	73187746	73187747	73187747	G	A	missense_variant
17-36167930-36167932-GC-TT		1		Gm8909	A114K	1	YYNQSKGGS	6	1	289.91	340.993	1.4	1.7	8.461	0	0	0	0.067	NoExpr	Pending	17	36167930	36167932	36167931	GC	TT	missense_variant
X-73959161-73959162-A-T		1		Hcfc1	L155Q	1	CYLFGGQAN	7	1	291.13	1054.61	0.8	2.9	36.628	0	0	0	0.052	NoExpr	Pending	X	73959161	73959162	73959162	A	T	missense_variant
2-87628700-87628701-G-T	1	2	1	Olfr1131	K79N	1	CYSTAIGPNML	9	4	291.42	461.94	0.45	0.6	0	0	0	0	0.235	NoExpr	Pending	2	87628700	87628701	87628701	G	T	missense_variant
8-21095988-21095990-GG-AC		1		Defa38	P21V	1	AFQVQADVI	8	1	293.83	353.256	0.8	0.7	0	0	0	0	0.027	NoExpr	Pending	8	21095988	21095990	21095989	GG	AC	missense_variant
7-102753071-102753072-G-A		2		Olfr560	P286S	1	TYLLISPV	6	2	296.56	801.611	0.45	0.8	0	0	0	0	0.247	NoExpr	Pending	7	102753071	102753072	102753072	G	A	missense_variant
16-29260943-29260944-C-G		2	2	Atp13a5	A939P	1	QYLLQDVPI	8	4	297.23	157.493	0.52	0.4	0	0	0	0	0.237	NoExpr	Pending	16	29260943	29260944	29260944	C	G	missense_variant
12-55676671-55676672-T-C		2		Ralgapa1	T1690A	1	QYLGTASPECL	6	2	297.43	472.835	0.46	0.71	16.509	0	0	0	0.483	NoExpr	Pending	12	55676671	55676672	55676672	T	C	missense_variant
9-3024482-3024483-T-A		2	1	Gm10718	C156S	1	SGSFSFSMI	3	3	297.91	263.688	0.8	0.8	0	0	0	0	0.088	NoExpr	Pending	9	3024482	3024483	3024483	T	A	missense_variant
1-20520287-20520288-C-T		1		Pkhd1	G1745D	1	NYGCLDGRL	6	1	298.6	296.545	0.8	0.8	0.001	0	0	0	0.537	NoExpr	Pending	1	20520287	20520288	20520288	C	T	missense_variant
13-56251498-56251499-A-C		3		Neurog1	L145R	1	NYIWARAETL	6	3	298.69	183.13	0.2	0.27	0	0	0	0	0.09	NoExpr	Pending	13	56251498	56251499	56251499	A	C	missense_variant
17-35380098-35380100-AA-TC		1		H2-Q4	K115I	1	EYWERETQI	9	1	298.72	5274.78	0.5	12	256.73	0	0	0	0.035	NoExpr	Pending	17	35380098	35380100	35380099	AA	TC	missense_variant
12-115980898-115980899-A-C		1		Ighv1-84	Y52D	1	GYTFTDYDI	8	1	299.29	157.493	0.7	0.39	0	0	0	0	0.045	NoExpr	Pending	12	115980898	115980899	115980899	A	C	missense_variant
12-115067681-115067683-TA-CG		2		Ighv8-5	Y79R	1	RYNPSLKSRL	1	2	299.46	130.64	0.38	0.2	0	0	0	0	0.034	NoExpr	Pending	12	115067681	115067683	115067682	TA	CG	missense_variant
1-154448915-154448916-G-A		1		Cacna1e	T1256I	1	KIIKSLRVL	2	1	299.47	492.617	1.5	2.1	0	0	0	0	0.225	NoExpr	Pending	1	154448915	154448916	154448916	G	A	missense_variant
11-9577190-9577191-T-G		2		Abca13	F4535V	1	SRIVSSSDV	4	2	300.67	460.352	0.9	1.4	0.032	0	0	0	0.296	NoExpr	Pending	11	9577190	9577191	9577191	T	G	missense_variant
7-103155588-103155589-T-G		1		Olfr589	T53P	1	FVIKPESSL	5	1	300.67	128.777	1.3	0.6	0	0	0	0	0.261	NoExpr	Pending	7	103155588	103155589	103155589	T	G	missense_variant
2-85991848-85991849-T-G		1		Olfr1031	F11V	1	NFTSVTEVI	8	1	301.48	378.521	0.8	0.9	0	0	0	0	0.186	NoExpr	Pending	2	85991848	85991849	85991849	T	G	missense_variant
1-173860338-173860339-C-A		1	1	Mndal	S443I	1	KTQNTIYEI	6	2	301.88	438.359	1.2	1.8	124.57	0	0	0	0.135	NoExpr	Pending	1	173860338	173860339	173860339	C	A	missense_variant
1-66414347-66414348-A-T		4	2	Map2	K799I	1	YYINGTVMAPD	3	6	302.61	2339.71	0.7	4.5	0.094	0	0	0	0.199	NoExpr	Pending	1	66414347	66414348	66414348	A	T	missense_variant
X-79355059-79355060-A-C		1		Cfap47	L2332R	1	AFQPRTQNI	5	1	302.75	104.014	0.9	0.3	0	0	0	0	0.339	NoExpr	Pending	X	79355059	79355060	79355060	A	C	missense_variant
7-8368096-8368097-G-A		4	1	Vmn2r44	T650I	1	HPNRAICVL	6	5	303.97	434.34	0.5	0.9	0	0	0	0	0.031	NoExpr	Pending	7	8368096	8368097	8368097	G	A	missense_variant
5-95342668-95342669-T-G		1		Gm3259	F387V	1	FYNNDVSMCTL	6	1	304.56	353.64	0.46	0.5	0	0	0	0	0.032	NoExpr	Pending	5	95342668	95342669	95342669	T	G	missense_variant
5-52848759-52848760-A-T		1		Anapc4	L330F	1	GFKKLGQSI	2	1	304.85	1066.82	0.9	3	64.857	0	0	0	0.157	NoExpr	Pending	5	52848759	52848760	52848760	A	T	missense_variant
19-8909001-8909002-C-G		1		Ganab	P260A	1	AYGPTSVGL	1	1	305.99	1625.89	1	4.2	65.978	0	0	0	0.05	NoExpr	Pending	19	8909001	8909002	8909002	C	G	missense_variant
12-105222561-105222562-G-A		1		Tcl1	R31C	1	VYLDEFCRSWL	7	1	306.02	265.87	0.6	0.6	0	0	0	0	0.076	NoExpr	Pending	12	105222561	105222562	105222562	G	A	missense_variant
3-101439441-101439442-G-C		1	1	Igsf3	L564F	1	VTYSDSFDF	9	2	306.78	435.341	1.3	1.5	1.674	0	0	0	0.034	NoExpr	Pending	3	101439441	101439442	101439442	G	C	missense_variant
2-132879719-132879720-G-T		1		Lrrn4	P59H	1	LHAAGVTTL	2	1	307.86	944.257	1.2	2.7	0	0	0	0	0.39	NoExpr	Pending	2	132879719	132879720	132879720	G	T	missense_variant
14-14087496-14087497-T-G		1	1	Atxn7	H225Q	1	KPMQPVQQI	4	1	309.35	346.011	1.5	1.4	6.559	0	0	0	0.317	NoExpr	Pending	14	14087496	14087497	14087497	T	G	missense_variant
8-25596378-25596379-T-A		3	1	Letm2	L10F	1	SYNSFFAI	6	3	313.23	276.45	0.47	0.43	1.352	0	0	0	0.209	NoExpr	Pending	8	25596378	25596379	25596379	T	A	missense_variant
2-29949236-29949237-C-T		1	1	Gle1	H514Y	1	LYPKVGDLI	2	2	313.39	3305.67	0.5	8.3	30.994	0	0	0	0.033	NoExpr	Pending	2	29949236	29949237	29949237	C	T	missense_variant
2-145604547-145604548-G-A		3		Slc24a3	R292H	1	SHFPPKTHL	8	3	313.39	468.911	0.9	1.4	0.393	0	0	0	0.355	NoExpr	Pending	2	145604547	145604548	145604548	G	A	missense_variant
5-65825383-65825384-C-G		3	1	N4bp2	S1660R	1	KYLTRHSFR	5	4	313.71	88.973	1.2	0.3	6.819	0	0	0	0.087	NoExpr	Pending	5	65825383	65825384	65825384	C	G	missense_variant
7-85955964-85955965-T-A		1	1	Vmn2r74	I492L	1	LYMSKEMI	1	2	314.72	247.55	0.2	0.2	0	0	0	0	0.031	NoExpr	Pending	7	85955964	85955965	85955965	T	A	missense_variant
17-36168007-36168008-C-G		1		Gm8909	E88D	1	EYWERDTQI	6	1	314.96	298.717	0.5	0.5	8.461	0	0	0	0.023	NoExpr	Pending	17	36168007	36168008	36168008	C	G	missense_variant
17-35439638-35439639-G-C		1		H2-Q7	E84D	1	EYWERDTQI	6	1	314.96	298.717	0.5	0.5	7.924	0	0	0	0.063	NoExpr	Pending	17	35439638	35439639	35439639	G	C	missense_variant
2-88728951-88728952-A-G		1	1	Olfr1197	Y216H	1	SHILILYSL	2	2	316.1	420.562	1.3	1.8	0	0	0	0	0.233	NoExpr	Pending	2	88728951	88728952	88728952	A	G	missense_variant
6-4516913-4516914-G-A		1		Col1a2	M191I	1	KGVKGHSGI	9	1	316.12	1145.59	1.5	4	527.08	0	0	0	0.858	NoExpr	Pending	6	4516913	4516914	4516914	G	A	missense_variant
2-86958857-86958858-A-C		2		Olfr1099	F200C	1	ICIFAGSTL	2	2	317.15	153.2	0.9	0.3	0	0	0	0	0.213	NoExpr	Pending	2	86958857	86958858	86958858	A	C	missense_variant
12-113578601-113578602-T-G		1	1	Ighv5-2	R85S	1	DTMESRFII	5	2	319.03	581.875	1.3	2.3	0	0	0	0	0.015	NoExpr	Pending	12	113578601	113578602	113578602	T	G	missense_variant
17-51742704-51742705-C-G		1		Satb1	V574L	1	IYEQESNAL	9	1	323.04	714.644	0.9	1.9	0.476	0	0	0	0.07	NoExpr	Pending	17	51742704	51742705	51742705	C	G	missense_variant
5-96769650-96769651-G-A		1		Fras1	R3510K	1	FYDTVLWKTGI	8	1	323.5	323.504	0.4	0.4	0.162	0	0	0	0.494	NoExpr	Pending	5	96769650	96769651	96769651	G	A	missense_variant
11-3140172-3140173-C-T		4		Sfi1	A716T	1	IYYRQKEATAL	9	4	325.07	626.14	0.2	0.83	28.733	0	0	0	0.024	NoExpr	Pending	11	3140172	3140173	3140173	C	T	missense_variant
7-7479794-7479795-T-C		1		Vmn2r32	N60S	1	YFSKTLNFL	3	1	325.28	257.686	0.9	0.7	0	0	0	0	0.062	NoExpr	Pending	7	7479794	7479795	7479795	T	C	missense_variant
2-180733805-180733806-T-A		1		Slc17a9	S174T	1	TYSTVGAGTQV	9	1	325.46	337.33	0.4	0.4	2.214	0	0	0	0.279	NoExpr	Pending	2	180733805	180733806	180733806	T	A	missense_variant
2-87435373-87435374-T-A		1	1	Olfr1124	Y296N	1	TPMFNPMVNSL	9	2	326.54	146.43	0.2	0.2	0	0	0	0	0.286	NoExpr	Pending	2	87435373	87435374	87435374	T	A	missense_variant
11-96107512-96107513-G-A		3	1	Calcoco2	A28V	1	KFYVPRGDI	4	4	326.66	211.831	0.9	0.6	0	0	0	0	0.051	NoExpr	Pending	11	96107512	96107513	96107513	G	A	missense_variant
13-100427120-100427121-T-C		1		Naip1	D512G	1	FYLSLSSITPG	11	1	327.88	356.46	0.8	0.8	0.215	0	0	0	0.014	NoExpr	Pending	13	100427120	100427121	100427121	T	C	missense_variant
11-52032469-52032470-C-T		2	1	Cdkl3	H525Y	1	KCDRKEFYF	8	3	328.72	453.764	1.3	1.9	2.501	0	0	0	0.087	NoExpr	Pending	11	52032469	52032470	52032470	C	T	missense_variant
9-3024477-3024478-G-T		1	1	Gm10718	S154I	1	SIGCFSFSM	2	2	328.85	522.396	1.3	2.1	0	0	0	0	0.087	NoExpr	Pending	9	3024477	3024478	3024478	G	T	missense_variant
16-32754346-32754347-C-T		2	1	Muc4	T1407I	1	APITSIQIL	6	2	328.85	500.035	1.2	2	0.047	0	0	0	0.034	NoExpr	Pending	16	32754346	32754347	32754347	C	T	missense_variant
11-67205509-67205510-G-A		1		Myh1	E272K	1	TYLLKKSRV	5	1	330.01	291.44	1.6	1.1	1.728	0	0	0	0.626	NoExpr	Pending	11	67205509	67205510	67205510	G	A	missense_variant

ID	H-2-Dd	H-2-Kd	H-2-Ld	Gene	AA Change	Num Passing Transcripts	Best Peptide	Pos	Num Passing Peptides	IC50 MT	IC50 WT	%ile MT	%ile WT	RNA Expr	RNA VAF	Allele Expr	RNA Depth	DNA VAF	Tier	Evaluation	CHROM	START	END	POS	REF_y	ALT_y	biotype
2-86922884-86922885-C-A		4		Olfr1098	A216S	1	SSYVSILSTIL	1	4	331.18	480.72	0.5	0.5	0	0	0	0	0.049	NoExpr	Pending	2	86922884	86922885	86922885	C	A	missense_variant
3-108402733-108402734-T-C		2		Celsr2	D1560G	1	RHIDMAGFI	7	2	331.33	356.93	0.51	0.53	0.015	0	0	0	0.215	NoExpr	Pending	3	108402733	108402734	108402734	T	C	missense_variant
10-79017644-79017645-T-C		2	1	Olfr1351	F108L	1	YFLLLLSVL	6	3	331.33	427.819	0.6	0.6	0	0	0	0	0.028	NoExpr	Pending	10	79017644	79017645	79017645	T	C	missense_variant
11-71122814-71122817-CTC-GGT		1		Nlrp1a	E536T	1	LYENQDMTL	8	1	333.63	643.073	0.7	1.8	0.059	0	0	0	0.081	NoExpr	Pending	11	71122814	71122817	71122815	CTC	GGT	missense_variant
5-109047017-109047018-G-C		2		Vmn2r11	T814R	1	VYHSTRGRV	8	2	333.95	59.09	0.8	0.4	0	0	0	0	0.052	NoExpr	Pending	5	109047017	109047018	109047018	G	C	missense_variant
17-29627179-29627180-G-C		2		Rnf8	E305D	1	LYADQAQQQ	4	2	334.26	323.661	1	0.9	10.506	0	0	0	0.241	NoExpr	Pending	17	29627179	29627180	29627180	G	C	missense_variant
12-114946166-114946168-CC-TG		1	1	Ighv1-43	G45H	1	SCKASHYSF	6	2	336.51	292.415	1.4	1.2	0	0	0	0	0.026	NoExpr	Pending	12	114946166	114946168	114946167	CC	TG	missense_variant
X-74303847-74303848-G-T		1		Atp6ap1	A412S	1	SYASDCSGF	7	1	337.19	1535.54	0.75	4.2	194.82	0	0	0	0.04	NoExpr	Pending	X	74303847	74303848	74303848	G	T	missense_variant
12-114538690-114538692-CA-GT		2		Ighv1-7	W52T	1	GYTFTSYTM	8	2	337.51	1007.14	0.6	2.2	0	0	0	0	0.019	NoExpr	Pending	12	114538690	114538692	114538691	CA	GT	missense_variant
2-86131198-86131199-C-T		3	1	Olfr1039	V155M	1	STYIYGFTM	9	4	339.63	4120.98	1.4	5.8	0	0	0	0	0.194	NoExpr	Pending	2	86131198	86131199	86131199	C	T	missense_variant
16-58916367-58916368-C-T		1		Olfr180	R91K	1	NFFSVDRKI	8	1	339.83	205.712	1	0.63	0	0	0	0	0.027	NoExpr	Pending	16	58916367	58916368	58916368	C	T	missense_variant
5-129020894-129020895-G-A		1	1	Ran	V51I	1	EVHPLIFHT	6	2	341.19	399.945	1.4	1.6	546.14	0	0	0	0.026	NoExpr	Pending	5	129020894	129020895	129020895	G	A	missense_variant
6-40900068-40900069-G-A		1	1	Prss58	L9F	1	AFLCIFSTL	6	2	343.2	329.678	0.8	0.8	0	0	0	0	0.783	NoExpr	Pending	6	40900068	40900069	40900069	G	A	missense_variant
8-21096040-21096041-G-A		1		Defa38	L4F	1	KTFVLLSAL	3	1	344.7	482.072	1.5	1.6	0	0	0	0	0.021	NoExpr	Pending	8	21096040	21096041	21096041	G	A	missense_variant
2-98662377-98662379-CC-TC		1	2	Gm10801	P48S	1	SPFSVFLAI	1	2	344.7	1109	1.2	2	0.204	0	0	0	0.031	NoExpr	Pending	2	98662377	98662379	98662378	CC	TC,TT	missense_variant
10-102385080-102385081-G-A		1		Mgat4c	D75N	1	RYVHTFKNL	8	1	344.81	668.64	0.6	0.86	0	0	0	0	0.038	NoExpr	Pending	10	102385080	102385081	102385081	G	A	missense_variant
12-113611289-113611290-G-A		3		Ighv2-3	L82F	1	NYHSAFISRL	6	3	346.39	284.66	0.51	0.44	0	0	0	0	0.061	NoExpr	Pending	12	113611289	113611290	113611290	G	A	missense_variant
1-172317608-172317609-C-G		1		Igsf8	P231A	1	AYAERLASGEL	1	1	349.04	625.01	0.6	1.2	45.726	0	0	0	0.037	NoExpr	Pending	1	172317608	172317609	172317609	C	G	missense_variant
12-114538619-114538620-A-C		2		Ighv1-7	Y76D	1	GYINPSSGDTK	9	2	349.85	326.498	0.9	0.8	0	0	0	0	0.124	NoExpr	Pending	12	114538619	114538620	114538620	A	C	missense_variant
4-118933989-118933990-G-C		2		Olfr1328	A284G	1	FYNAFTGLL	7	2	350.83	774.622	1	2.3	0	0	0	0	0.027	NoExpr	Pending	4	118933989	118933990	118933990	G	C	missense_variant
19-42189578-42189579-C-G		1		Zfyve27	P387A	1	SFKVARSSM	5	1	350.83	831.936	0.8	1.7	21.878	0	0	0	0.062	NoExpr	Pending	19	42189578	42189579	42189579	C	G	missense_variant
9-45332507-45332508-C-G		1		Tmprss13	P181A	1	KYKEALESCPI	5	1	351.26	523.52	0.52	1.7	0	0	0	0	0.068	NoExpr	Pending	9	45332507	45332508	45332508	C	G	missense_variant
15-78254866-78254867-G-C		2		Ncf4	E141D	1	FYQSAYDAD	9	2	351.63	266.846	1	0.7	35.362	0	0	0	0.106	NoExpr	Pending	15	78254866	78254867	78254867	G	C	missense_variant
7-10159190-10159191-C-T		1	1	Vmn2r52	V674I	1	ITVILAFKF	1	2	352.23	616.354	1.7	2.4	0	0	0	0	0.011	NoExpr	Pending	7	10159190	10159191	10159191	C	T	missense_variant
8-105270613-105270614-A-C		2	2	Hsf4	K64T	1	LPQYFTHSNM	6	4	352.24	374.72	0.5	0.5	9.436	0	0	0	0.131	NoExpr	Pending	8	105270613	105270614	105270614	A	C	missense_variant
16-16868672-16868673-C-T		1		Vpreb1	G118R	1	YYCAVRLRS	6	1	352.24	432.636	1.3	1.9	0.273	0	0	0	0.468	NoExpr	Pending	16	16868672	16868673	16868673	C	T	missense_variant
1-167226427-167226428-G-A		2	1	Uck2	P247L	1	GYLNGYTL	8	3	352.51	1661.88	0.47	2.4	55.37	0	0	0	0.2	NoExpr	Pending	1	167226427	167226428	167226428	G	A	missense_variant
13-65296554-65296555-C-G		1		Zfp369	T504S	1	KTQSSRDPI	4	1	353.26	315.99	1	1.2	9.863	0	0	0	0.183	NoExpr	Pending	13	65296554	65296555	65296555	C	G	missense_variant
12-65055164-65055165-C-T		2		Prpf39	P339S	1	SYFHVKPL	1	2	353.39	896.314	0.27	0.7	21.084	0	0	0	0.344	NoExpr	Pending	12	65055164	65055165	65055165	C	T	missense_variant
1-84963776-84963777-G-A		2		AC167036.1	V37I	1	FSITTSLTI	9	2	354.13	2234.91	0.8	6	0	0	0	0	0.044	NoExpr	Pending	1	84963776	84963777	84963777	G	A,T	missense_variant
12-114896406-114896407-C-A		1		Ighv1-37	G61V	1	SHVKSLEWI	3	1	355.71	534.884	1	1.4	0	0	0	0	0.024	NoExpr	Pending	12	114896406	114896407	114896407	C	A	missense_variant
12-51365553-51365554-C-T		1		G2e3	S459F	1	NFQAVKENL	2	1	356.07	1709.59	0.7	5.1	12.517	0	0	0	0.346	NoExpr	Pending	12	51365553	51365554	51365554	C	T	missense_variant
1-174191043-174191044-G-A		1		Spta1	E527K	1	AFTAQKEKI	6	1	358.17	453.169	1.1	1.8	0.805	0	0	0	0.25	NoExpr	Pending	1	174191043	174191044	174191044	G	A	missense_variant
15-78297059-78297060-G-C		1		Csf2rb2	T44S	1	CYNDYSNRI	6	1	358.59	501.291	0.9	1.2	7.913	0	0	0	0.676	NoExpr	Pending	15	78297059	78297060	78297060	G	C	missense_variant
3-55255881-55255882-G-A		1		Dclk1	G132S	1	GESYVCSSI	7	1	359	948.615	1.1	2.8	0.158	0	0	0	0.261	NoExpr	Pending	3	55255881	55255882	55255882	G	A	missense_variant
10-83233649-83233650-C-T		1	2	Slc41a2	G546D	1	IPYLTALDDLL	8	3	359.68	141.87	0.07	0.06	10.757	0	0	0	0.522	NoExpr	Pending	10	83233649	83233650	83233650	C	T	missense_variant
11-3153459-3153460-C-T		1		Sfi1	R438Q	1	QYVHKRQWQQ	1	1	359.95	182.339	0.7	0.4	28.733	0	0	0	0.031	NoExpr	Pending	11	3153459	3153460	3153460	C	T	missense_variant
17-36167979-36167980-C-T		1		Gm8909	G98S	1	SFQGSLRNL	1	1	361.63	1528.48	1.1	2.8	8.461	0	0	0	0.048	NoExpr	Pending	17	36167979	36167980	36167980	C	T	missense_variant
7-5125778-5125780-CT-TG		1	1	Rasl2-9	LV50-51FI	1	EVHTFIFHT	5	2	362.24	445.656	1.7	1.8	0.401	0	0	0	0.036	NoExpr	Pending	7	5125778	5125780	5125779	CT	TG	missense_variant
9-38816146-38816147-G-T		2	2	Olfr922	V215F	1	ITIFVSYTF	4	4	362.94	470.793	1.4	1.7	0	0	0	0	0.047	NoExpr	Pending	9	38816146	38816147	38816147	G	T	missense_variant
7-21133364-21133365-A-C		2	2	Vmn1r122	F255C	1	FYLLNCICI	6	4	363.15	562.92	0.7	0.8	0	0	0	0	0.037	NoExpr	Pending	7	21133364	21133365	21133365	A	C	missense_variant
1-14887649-14887650-T-G		1	1	Trpa1	N695H	1	MVQHHRIEL	5	2	363.77	389.789	1.3	1.3	0.06	0	0	0	0.138	NoExpr	Pending	1	14887649	14887650	14887650	T	G	missense_variant
6-80021913-80021914-A-G		1		Lrrtm4	E103G	1	NYISSVDGD	8	1	364.83	358.996	1.2	1.1	0	0	0	0	0.136	NoExpr	Pending	6	80021913	80021914	80021914	A	G	missense_variant
16-35025907-35025908-C-T		1		Hacd2	P80L	1	LYYSIERLL	8	1	366.51	145.89	0.8	0.4	14.908	0	0	0	0.051	NoExpr	Pending	16	35025907	35025908	35025908	C	T	missense_variant
2-87269648-87269649-C-A		2		Olfr1116	F268L	1	LYTILIPAL	1	2	368.21	246.752	1	0.6	0	0	0	0	0.289	NoExpr	Pending	2	87269648	87269649	87269649	C	A	missense_variant
16-57133127-57133128-A-C		1		Tomm70a	K129Q	1	KYFQAGKYE	4	1	369.2	283.962	0.8	0.6	66.012	0	0	0	0.228	NoExpr	Pending	16	57133127	57133128	57133128	A	C	missense_variant
7-85624395-85624396-C-T		1	1	Vmn2r71	A806V	1	MVVVEVFCI	2	2	371.39	677.376	1.9	2.6	0	0	0	0	0.041	NoExpr	Pending	7	85624395	85624396	85624396	C	T	missense_variant
8-92996027-92996029-CT-TG		1	3	Slc6a2	P551L	1	TYLPWANWV	3	4	371.82	2740.81	1.9	9.3	0	0	0	0	0.276	NoExpr	Pending	8	92996027	92996029	92996028	CT	TG	missense_variant
18-60550219-60550220-A-C		1	1	Dctn4	N290T	1	TPTSIKFKI	1	2	373.1	299.473	1.5	1.2	28.215	0	0	0	0.385	NoExpr	Pending	18	60550219	60550220	60550220	A	C	missense_variant
8-21734542-21734543-A-C		1	2	Defa24	T3P	1	KPLILLSAL	2	2	373.25	7306.9	1.4	14	0	0	0	0	0.021	NoExpr	Pending	8	21734542	21734543	21734543	A	C	missense_variant
1-155789391-155789392-C-T		2		Qsox1	G268R	1	SYLRRLPGL	5	2	373.47	260.178	1.1	0.46	52.398	0	0	0	0.571	NoExpr	Pending	1	155789391	155789392	155789392	C	T	missense_variant
5-8397260-8397261-T-C		1	1	Dbf4	T650A	1	AFFSSPSTS	1	2	374.19	687.211	1.4	1.8	86.455	0	0	0	0.149	NoExpr	Pending	5	8397260	8397261	8397261	T	C	missense_variant
17-58967344-58967345-A-T		2		Pdzph1	S835T	1	SSAGGSTVI	7	2	375.05	257.093	1.3	0.8	0	0	0	0	0.116	NoExpr	Pending	17	58967344	58967345	58967345	A	T	missense_variant
3-98619430-98619431-T-C		2		Hsd3b5	H233R	1	VYVGNAAWAR	10	2	375.56	243.824	0.7	0.5	0	0	0	0	0.139	NoExpr	Pending	3	98619430	98619431	98619431	T	C	missense_variant
7-98161940-98161941-G-A		3		Capn5	P47L	1	LYYKGTPGL	9	3	375.87	3616.26	1.8	9	37.993	0	0	0	0.192	NoExpr	Pending	7	98161940	98161941	98161941	G	A	missense_variant
3-64086727-64086728-C-T		1		Vmn2r1	A165V	1	SLSVAVSRI	6	1	376.78	333.496	1.5	1.3	0.015	0	0	0	0.453	NoExpr	Pending	3	64086727	64086728	64086728	C	T	missense_variant
12-31240838-31240839-G-T		1		Gm6993	V24L	1	DYQYLLVTL	9	1	376.93	961.7	0.8	1.4	0	0	0	0	0.2	NoExpr	Pending	12	31240838	31240839	31240839	G	T	missense_variant
X-73448840-73448841-C-T		1		Haus7	S138N	1	KILDMMQNL	8	1	377.65	200.489	1.4	0.9	50.306	0	0	0	0.214	NoExpr	Pending	X	73448840	73448841	73448841	C	T	missense_variant
3-79480804-79480805-G-A		1		Fnip2	P843L	1	GYKGLSSEPV	5	1	384.09	525.658	0.32	0.37	10.255	0	0	0	0.44	NoExpr	Pending	3	79480804	79480805	79480805	G	A	missense_variant
18-6992834-6992835-C-T		2		Mkx	V150I	1	KYIQGNAERL	3	2	384.83	686.32	0.56	0.89	0.024	0	0	0	0.315	NoExpr	Pending	18	6992834	6992835	6992835	C	T	missense_variant
8-110866086-110866087-G-A		1		Cog4	V432I	1	EYFMRETI	8	1	385.15	478.035	0.2	0.34	46.564	0	0	0	0.791	NoExpr	Pending	8	110866086	110866087	110866087	G	A	missense_variant
1-44156942-44156943-G-A		2		Ercc5	S32N	1	INIWLNQAL	2	2	385.56	755.249	1.2	2.1	17.494	0	0	0	0.285	NoExpr	Pending	1	44156942	44156943	44156943	G	A	missense_variant

ID	H-2-Dd	H-2-Kd	H-2-Ld	Gene	AA Change	Num Passing Transcripts	Best Peptide	Pos	Num Passing Peptides	IC50 MT	IC50 WT	%ile MT	%ile WT	RNA Expr	RNA VAF	Allele Expr	RNA Depth	DNA VAF	Tier	Evaluation	CHROM	START	END	POS	REF_y	ALT_y	biotype
5-24388283-24388284-C-T		1		Atg9b	G374S	1	NYQVALANKSL	10	1	385.65	720.626	0.56	0.87	15.578	0	0	0	0.049	NoExpr	Pending	5	24388283	24388284	24388284	C	T	missense_variant
8-105949816-105949817-C-G		2		Slc12a4	G491A	1	KYGDAVSRNLV	5	2	386.16	1180	0.56	0.78	59.419	0	0	0	0.204	NoExpr	Pending	8	105949816	105949817	105949817	C	G	missense_variant
8-82864146-82864147-C-G		1		Rnf150	I46M	1	WYTAFVNMTYL	8	1	387.87	267.87	0.3	0.4	0.982	0	0	0	0.205	NoExpr	Pending	8	82864146	82864147	82864147	C	G	missense_variant
18-46850859-46850860-T-A		1		Lvrn	H223Q	1	LYQDEDELRAL	3	1	388.27	659.754	0.8	1.2	0.129	0	0	0	0.174	NoExpr	Pending	18	46850859	46850860	46850860	T	A	missense_variant
19-40723131-40723132-C-T		1		Entpd1	A132V	1	VYLAECMEL	1	1	388.83	254.84	1.2	0.8	10.299	0	0	0	0.224	NoExpr	Pending	19	40723131	40723132	40723132	C	T	missense_variant
7-26611382-26611384-GG-AT		1		Vmn1r185	S232Y	1	NYPESRATQNI	2	1	388.94	8571.13	0.4	8.6	0	0	0	0	0.046	NoExpr	Pending	7	26611382	26611384	26611383	GG	AT	missense_variant
12-113578603-113578604-T-C		1	1	Ighv5-2	R85G	1	DTMEGRFII	5	2	390.69	581.875	1.6	2.3	0	0	0	0	0.015	NoExpr	Pending	12	113578603	113578604	113578604	T	C	missense_variant
4-89691819-89691820-G-C		1		Dmrta1	C339S	1	SKGDVVQAI	1	1	391.98	574.46	1.2	1.9	0.01	0	0	0	0.344	NoExpr	Pending	4	89691819	89691820	89691820	G	C	missense_variant
2-111420896-111420897-C-T		1		Olfr1286	C18Y	1	LYSSRQLEI	2	1	392.11	9080.09	0.7	20	0	0	0	0	0.534	NoExpr	Pending	2	111420896	111420897	111420897	C	T	missense_variant
3-5402307-5402308-G-T		1	2	Zfhx4	D2509Y	1	RPMDMPYMIFY	11	3	392.45	409.84	0.2	0.27	0.032	0	0	0	0.277	NoExpr	Pending	3	5402307	5402308	5402308	G	T	missense_variant
9-114629057-114629058-T-C		2		Cnot10	T127A	1	QYAEAISVGEK	3	2	392.49	531.377	0.8	1.6	31.602	0	0	0	0.229	NoExpr	Pending	9	114629057	114629058	114629058	T	C	missense_variant
9-20286211-20286212-G-A		2		Olfr39	G179D	1	LTFSMDTEI	6	2	392.73	390.023	1.2	0.96	0	0	0	0	0.294	NoExpr	Pending	9	20286211	20286212	20286212	G	A	missense_variant
11-71122792-71122793-C-A		1		Nlrp1a	D544Y	1	HYLQGTIV	2	1	393.45	10527.3	0.4	21	0.059	0	0	0	0.079	NoExpr	Pending	11	71122792	71122793	71122793	C	A	missense_variant
17-36165685-36165686-A-C		1		Gm8909	S271A	1	KWAAVVVPL	4	1	395.6	591.916	1.1	1.2	8.461	0	0	0	0.015	NoExpr	Pending	17	36165685	36165686	36165686	A	C	missense_variant
17-35473270-35473271-T-G		1		H2-Q10	S270A	1	KWAAVVVPL	4	1	395.6	591.916	1.1	1.2	11.753	0	0	0	0.029	NoExpr	Pending	17	35473270	35473271	35473271	T	G	missense_variant
10-8207552-8207553-C-T		2	1	Ust	R353K	1	HYVKEQFHL	4	3	396.28	411.15	2.1	1.9	0.605	0	0	0	0.487	NoExpr	Pending	10	8207552	8207553	8207553	C	T	missense_variant
16-15775958-15775959-A-C		1		Prkdc	K2753T	1	KRGLMEQTL	8	1	396.52	1205.29	1.2	3.9	6.26	0	0	0	0.09	NoExpr	Pending	16	15775958	15775959	15775959	A	C	missense_variant
2-86347205-86347206-T-G		1		Olfr1055	M187L	1	FYCDSLPLILL	11	1	396.76	636.62	0.8	2.4	0	0	0	0	0.086	NoExpr	Pending	2	86347205	86347206	86347206	T	G	missense_variant
3-55477848-55477849-A-C		1		Dclk1	K360N	1	SSTNVCSSM	4	1	397.27	340.993	1.7	1.7	0.158	0	0	0	0.336	NoExpr	Pending	3	55477848	55477849	55477849	A	C	missense_variant
9-39217761-39217762-C-T		4	1	Olfr944	T135I	1	LYNVIMSYQ	5	5	397.27	222.89	1.2	0.6	0	0	0	0	0.285	NoExpr	Pending	9	39217761	39217762	39217762	C	T	missense_variant
6-42838692-42838693-G-A		1	3	Olfr449	V271I	1	KIISIFYAI	2	3	399.79	274.05	1.4	1.1	0	0	0	0	0.842	NoExpr	Pending	6	42838692	42838693	42838693	G	A	missense_variant
17-37382615-37382616-A-C		1	1	Olfr105-ps	Q16H	1	GYSDIHELNPI	6	2	400.6	759.819	1.1	1.2	0	0	0	0	0.245	NoExpr	Pending	17	37382615	37382616	37382616	A	C	missense_variant
7-45651030-45651031-C-T		1	3	Fut2	A106T	1	MHNTLAPIF	4	4	401.08	323.035	1.5	1.3	0.738	0	0	0	0.015	NoExpr	Pending	7	45651030	45651031	45651031	C	T	missense_variant
17-35470391-35470392-G-T		1	1	H2-Q10	R30S	1	SYFETSVSR	1	2	401.08	1911	1.3	2.6	11.753	0	0	0	0.022	NoExpr	Pending	17	35470391	35470392	35470392	G	T	missense_variant
17-37952445-37952446-C-A		2		Olfr761	D193Y	1	YFGVLEVSI	1	2	401.08	5371.43	0.9	9.2	0	0	0	0	0.027	NoExpr	Pending	17	37952445	37952446	37952446	C	A	missense_variant
8-70891658-70891659-A-C		1		Slc5a5	V139G	1	QYLGATML	4	1	401.29	406.874	0.3	0.3	0.083	0	0	0	0.195	NoExpr	Pending	8	70891658	70891659	70891659	A	C	missense_variant
6-132803729-132803730-T-C		1	1	Tas2r117	V277A	1	AYIAFPTFH	1	2	401.87	899.684	1.2	2.5	0	0	0	0	0.157	NoExpr	Pending	6	132803729	132803730	132803730	T	C	missense_variant
17-30656922-30656923-C-G		1	1	Dnah8	A508G	1	NGIRMIHSV	2	2	403.89	666.945	1.3	2.1	0.348	0	0	0	0.064	NoExpr	Pending	17	30656922	30656923	30656923	C	G	missense_variant
11-70316308-70316309-C-G		1	1	Alox12e	G557A	1	IPNAPCTM	4	2	405.44	1073.27	0.4	0.71	0.017	0	0	0	0.241	NoExpr	Pending	11	70316308	70316309	70316309	C	G	missense_variant
12-115193691-115193692-C-T		1		Ighv1-54	V112I	1	SLTSEDSAI	9	1	405.44	2270.34	2	9.1	0	0	0	0	0.029	NoExpr	Pending	12	115193691	115193692	115193692	C	T	missense_variant
12-115847897-115847899-CA-TC		1		Ighv1-76	AV111-112AI	1	SLTSEDSAI	9	1	405.44	2270.34	2	9.1	0	0	0	0	0.053	NoExpr	Pending	12	115847897	115847899	115847898	CA	TC	missense_variant
12-113661820-113661821-G-C		3		Ighv5-9	Q101E	1	LYLEMSSL	4	3	405.5	317.27	0.21	0.2	0	0	0	0	0.01	NoExpr	Pending	12	113661820	113661821	113661821	G	C	missense_variant
17-20030004-20030005-G-C		1		Vmn2r104	T668S	1	FTMALASVL	7	1	409.51	638.646	1.2	1.3	0	0	0	0	0.046	NoExpr	Pending	17	20030004	20030005	20030005	G	C	missense_variant
7-85624410-85624411-T-A		2		Vmn2r71	F811Y	1	VYCILASSAGL	2	2	409.85	3766.31	0.8	3.7	0	0	0	0	0.027	NoExpr	Pending	7	85624410	85624411	85624411	T	A	missense_variant
9-104123116-104123117-C-T		2		Acad11	A690V	1	LTLKVAHSI	5	2	410.29	762.535	1.7	2.3	6.327	0	0	0	0.119	NoExpr	Pending	9	104123116	104123117	104123117	C	T	missense_variant
16-17574522-17574523-G-T		1		Slc7a4	A349D	1	VYAMADDGL	6	1	411.4	315.689	1	0.53	0.182	0	0	0	0.217	NoExpr	Pending	16	17574522	17574523	17574523	G	T	missense_variant
7-5480797-5480798-C-G		2		Vmn2r28	S801T	1	VYHTTKGKV	4	2	411.84	378.02	1.1	0.9	0	0	0	0	0.029	NoExpr	Pending	7	5480797	5480798	5480798	C	G	missense_variant
12-113685542-113685543-C-T		1		Ighv2-5	V97I	1	IFFKMNSLQ	1	1	412.34	701.601	1.2	2.2	0	0	0	0	0.049	NoExpr	Pending	12	113685542	113685543	113685543	C	T	missense_variant
17-13073603-13073604-G-A		1	1	Tcp10b	D305N	1	SNQKVIYYY	2	2	414	579.202	1.7	2.3	0.017	0	0	0	0.038	NoExpr	Pending	17	13073603	13073604	13073604	G	A	missense_variant
2-86508740-86508741-T-A		1		Olfr1076	I94N	1	NYFCAVQLS	1	1	414.09	450.91	1.2	1.3	0	0	0	0	0.041	NoExpr	Pending	2	86508740	86508741	86508741	T	A	missense_variant
2-66317951-66317952-T-G		1		Scn1a	K1094T	1	TGSSVETYI	7	1	414.09	1574.93	1.2	4.3	0	0	0	0	0.196	NoExpr	Pending	2	66317951	66317952	66317952	T	G	missense_variant
14-53337566-53337567-G-A		1		Trav13n-3	E89K	1	ASNKSRSSL	4	1	414.31	690.383	1.8	2.2	0	0	0	0	0.038	NoExpr	Pending	14	53337566	53337567	53337567	G	A	missense_variant
2-86639294-86639296-GA-AT		2		Olfr1084	SH137-138SY	1	LYTVIVSYK	8	2	416	429.625	1.3	1.3	0	0	0	0	0.022	NoExpr	Pending	2	86639294	86639296	86639295	GA	AT	missense_variant
8-63927832-63927833-A-C		1		Sgo2b	V655G	1	FFTRGNGSL	5	1	416.16	228.171	0.96	0.64	0	0	0	0	0.363	NoExpr	Pending	8	63927832	63927833	63927833	A	C	missense_variant
7-105024326-105024327-G-A		1	1	Olfr675	L214F	1	LLLDVVFII	7	2	417.67	10491.3	1.8	20	0	0	0	0	0.321	NoExpr	Pending	7	105024326	105024327	105024327	G	A	missense_variant
11-71156153-71156154-T-G		1		Nlrp1b	N1127T	1	SYEAVRAET	9	1	417.92	468.911	0.96	1.4	2.359	0	0	0	0.037	NoExpr	Pending	11	71156153	71156154	71156154	T	G	missense_variant
10-127064639-127064640-G-A		1		Cdk4	V92M	1	KVTLMFEHI	5	1	419.85	590.323	1.3	2.3	424.02	0	0	0	0.132	NoExpr	Pending	10	127064639	127064640	127064640	G	A	missense_variant
16-25871091-25871092-G-C		1		Trp63	R436S	1	TYSQQQQQQ	3	1	420.01	970.711	1.3	2.9	0.218	0	0	0	0.193	NoExpr	Pending	16	25871091	25871092	25871092	G	C	missense_variant
18-73931082-73931083-C-T		1	1	Mapk4	S356N	1	YPVNLSSDL	4	2	420.1	487.316	1.4	2	0.015	0	0	0	0.327	NoExpr	Pending	18	73931082	73931083	73931083	C	T	missense_variant
12-116000164-116000165-G-C		1		Ighv1-85	P72A	1	IYARDGSTK	3	1	421.95	2513.39	0.78	6.4	0	0	0	0	0.026	NoExpr	Pending	12	116000164	116000165	116000165	G	C	missense_variant
X-102071364-102071365-C-T		2		Nhsl2	H108Y	1	AYSRSSWRQ	2	2	421.95	8876.87	1.6	19	0.563	0	0	0	0.749	NoExpr	Pending	X	102071364	102071365	102071365	C	T	missense_variant
12-114538628-114538629-T-C		1		Ighv1-7	S73G	1	GYINPGSGYTK	6	1	422.55	326.498	1.2	0.8	0	0	0	0	0.134	NoExpr	Pending	12	114538628	114538629	114538629	T	C	missense_variant
2-33565196-33565197-C-T		2		Lmx1b	M282I	1	GIMASYTPL	2	2	422.76	263.26	0.9	0.8	0.013	0	0	0	0.219	NoExpr	Pending	2	33565196	33565197	33565197	C	T	missense_variant
12-104130473-104130474-G-T		1		Serpina3b	A5S	1	AFISALGLL	4	1	422.92	282.657	1	0.8	0	0	0	0	0.025	NoExpr	Pending	12	104130473	104130474	104130474	G	T	missense_variant
6-130275858-130275859-T-C		1	2	Klra10	S151G	1	GTKCYYFIM	1	3	423.37	344.702	2.199	1.6	0	0	0	0	0.069	NoExpr	Pending	6	130275858	130275859	130275859	T	C	missense_variant
17-47869256-47869257-C-T		1		Foxp4	S586N	1	SYQAALAEN	9	1	424.71	376.782	1.3	0.61	17.21	0	0	0	0.498	NoExpr	Pending	17	47869256	47869257	47869257	C	T	missense_variant
1-107271842-107271843-C-T		1		Serpinb3c	S316N	1	SGMNSTQGL	4	1	425.85	359.948	1.4	0.9	0	0	0	0	0.231	NoExpr	Pending	1	107271842	107271843	107271843	C	T	missense_variant
1-174612523-174612524-T-G		1		Fmn2	L1169R	1	KRDSSPSRI	8	1	427.98	489.88	2.199	1.7	0	0	0	0	0.278	NoExpr	Pending	1	174612523	174612524	174612524	T	G	missense_variant
2-40697359-40697360-C-T		1		Lrp1b	S3961N	1	KRQANNGLI	6	1	427.98	437.342	2.1	1.6	0.007	0	0	0	0.46	NoExpr	Pending	2	40697359	40697360	40697360	C	T	missense_variant
6-28526128-28526129-G-A		1		Snd1	G169S	1	SEGNSSHTI	5	1	428.64	736.36	1.3	2.3	252.84	0	0	0	0.902	NoExpr	Pending	6	28526128	28526129	28526129	G	A	missense_variant
1-174391788-174391789-C-G		1		Olfr248	A240G	1	AFTPCGSHL	6	1	428.81	408.69	1.6	1.3	0	0	0	0	0.203	NoExpr	Pending	1	174391788	174391789	174391789	C	G	missense_variant
X-93593841-93593842-C-T		1	1	Pola1	C501Y	1	RKIKGPYWL	7	2	431.35	5114.82	1.8	6.3	50.812	0	0	0	0.261	NoExpr	Pending	X	93593841	93593842	93593842	C	T	missense_variant

ID	H-2-Dd	H-2-Kd	H-2-Ld	Gene	AA Change	Num Passing Transcripts	Best Peptide	Pos	Num Passing Peptides	IC50 MT	IC50 WT	%ile MT	%ile WT	RNA Expr	RNA VAF	Allele Expr	RNA Depth	DNA VAF	Tier	Evaluation	CHROM	START	END	POS	REF_y	ALT_y	biotype
9-103479866-103479867-C-A		1		Bfsp2	M120I	1	EYITKVHAL	3	1	431.59	240.93	0.8	1	0	0	0	0	0.225	NoExpr	Pending	9	103479866	103479867	103479867	C	A	missense_variant
10-40920378-40920379-C-T		1		Wasf1	A35V	1	TNISLVNII	6	1	431.61	382.023	1.2	1	2.071	0	0	0	0.402	NoExpr	Pending	10	40920378	40920379	40920379	C	T	missense_variant
9-39950300-39950301-G-T		2	1	Olfr975	L157I	1	LHASVITFL	6	3	432.3	469.85	0.8	0.9	0	0	0	0	0.213	NoExpr	Pending	9	39950300	39950301	39950301	G	T	missense_variant
11-110301544-110301545-T-G		1		Abca5	I714L	1	GYRLSMYL	8	1	433.11	388.29	0.25	0.3	10.108	0	0	0	0.177	NoExpr	Pending	11	110301544	110301545	110301545	T	G	missense_variant
7-12670590-12670591-T-G		1		Vmn2r55	N295T	1	PYQTSTVTEGV	4	1	433.22	883.597	0.91	1.5	0	0	0	0	0.132	NoExpr	Pending	7	12670590	12670591	12670591	T	G	missense_variant
9-106435842-106435843-C-T		1		Acy1	R126K	1	QYLEAVKRL	7	1	433.32	457.03	1.2	0.7	25.029	0	0	0	0.332	NoExpr	Pending	9	106435842	106435843	106435843	C	T	missense_variant
8-48276524-48276525-G-C		1		Tenm3	A1482G	1	CYQSGDGYG	9	1	433.6	308.503	0.89	0.9	19.62	0	0	0	0.139	NoExpr	Pending	8	48276524	48276525	48276525	G	C	missense_variant
1-164263402-164263403-C-G		1		Slc19a2	A461G	1	GSYFAAISVV	1	1	433.79	424.268	0.41	0.4	19.559	0	0	0	0.229	NoExpr	Pending	1	164263402	164263403	164263403	C	G	missense_variant
4-88602850-88602851-T-A		2	1	Ifna12	Y153F	1	KYFHRITVF	9	3	434.6	1528.48	1.2	2.9	0	0	0	0	0.054	NoExpr	Pending	4	88602850	88602851	88602851	T	A	missense_variant
4-88683321-88683322-T-A		2	1	Ifna2	Y153F	1	KYFHRITVF	9	3	434.6	1528.48	1.2	2.9	0	0	0	0	0.042	NoExpr	Pending	4	88683321	88683322	88683322	T	A	missense_variant
4-88835981-88835982-A-T		2	1	Ifna5	Y153F	1	KYFHRITVF	9	3	434.6	1528.48	1.2	2.9	0	0	0	0	0.021	NoExpr	Pending	4	88835981	88835982	88835982	A	T	missense_variant
4-88591927-88591928-T-A		2	1	Ifna9	Y153F	1	KYFHRITVF	9	3	434.6	1528.48	1.2	2.9	0	0	0	0	0.017	NoExpr	Pending	4	88591927	88591928	88591928	T	A	missense_variant
16-32754538-32754539-A-G		1		Muc4	N1471S	1	SHAPSMSSS	5	1	437.34	1222.43	1.2	3.1	0.047	0	0	0	0.074	NoExpr	Pending	16	32754538	32754539	32754539	A	G	missense_variant
13-100245924-100245925-C-T		2		Naip5	R92K	1	FYHTGVKL	7	2	437.99	393.061	0.25	0.23	2.729	0	0	0	0.021	NoExpr	Pending	13	100245924	100245925	100245925	C	T	missense_variant
17-35471031-35471032-C-A		1		H2-Q10	L184M	1	AYMEAECVEW	3	1	439.24	271.686	0.4	0.6	11.753	0	0	0	0.067	NoExpr	Pending	17	35471031	35471032	35471032	C	A	missense_variant
7-48871006-48871007-A-G	1	3		E2f8	I522T	1	TYLQPAQAQ	1	4	440.65	315.566	1.2	0.9	54.724	0	0	0	0.396	NoExpr	Pending	7	48871006	48871007	48871007	A	G	missense_variant
16-32755662-32755663-T-A		1		Muc4	L1846M	1	SHASSMSSS	6	1	440.65	480.96	1.3	1.5	0.047	0	0	0	0.01	NoExpr	Pending	16	32755662	32755663	32755663	T	A	missense_variant
3-130620788-130620789-C-T		3		Etnppl	S111F	1	CYFTNFGSE	6	3	441.84	291.246	1.4	0.8	0	0	0	0	0.5	NoExpr	Pending	3	130620788	130620789	130620789	C	T	missense_variant
14-56082935-56082936-T-G		1		Mcpt8	N179H	1	TYHDSIQL	3	1	442.1	1564.8	0.56	1.2	0.26	0	0	0	0.069	NoExpr	Pending	14	56082935	56082936	56082936	T	G	missense_variant
8-13319799-13319800-C-T		2		Tmco3	P604L	1	ILLRSSQYI	3	2	444.9	1670.75	0.8	3.7	12.652	0	0	0	0.268	NoExpr	Pending	8	13319799	13319800	13319800	C	T	missense_variant
4-113916156-113916157-T-A		1		Skint5	K330I	1	LLIFIQSSI	3	1	445.92	3532.56	1.3	6.4	0	0	0	0	0.037	NoExpr	Pending	4	113916156	113916157	113916157	T	A	missense_variant
12-114222980-114222981-C-T		2		Ighv7-4	R57H	1	YYMSWVHQP	7	2	446.91	696.429	1.1	1.8	0	0	0	0	0.086	NoExpr	Pending	12	114222980	114222981	114222981	C	T	missense_variant
18-34845348-34845349-C-T		1		Reep2	P94S	1	YRKFVHSTL	7	1	447.81	2951.82	1.4	7.5	0.255	0	0	0	0.147	NoExpr	Pending	18	34845348	34845349	34845349	C	T	missense_variant
17-18929572-18929573-C-T		2	1	Vmn2r97	H408Y	1	GVYAVAYSL	7	3	448.57	2146.99	1.4	6.1	0	0	0	0	0.013	NoExpr	Pending	17	18929572	18929573	18929573	C	T	missense_variant
17-35473276-35473277-G-A		1		H2-Q10	V272M	1	KWASVMVPL	6	1	450.05	591.916	1.1	1.2	11.753	0	0	0	0.039	NoExpr	Pending	17	35473276	35473277	35473277	G	A	missense_variant
6-132957684-132957685-T-C		1		Tas2r131	M54V	1	SRISSVLVL	8	1	451.77	652.654	1.3	1.8	0	0	0	0	0.128	NoExpr	Pending	6	132957684	132957685	132957685	T	C	missense_variant
8-26110759-26110760-A-G		2		Hook3	V40A	1	NGVAMSQVL	4	2	451.95	835.776	2	2.5	21.258	0	0	0	0.545	NoExpr	Pending	8	26110759	26110760	26110760	A	G	missense_variant
12-40036640-40036641-C-T		2		Arl4a	V36I	1	LDCAGKTTI	9	2	454.04	2704.52	1.3	7	8.721	0	0	0	0.062	NoExpr	Pending	12	40036640	40036641	40036641	C	T	missense_variant
10-36828795-36828796-G-A		1		Hs3st5	G32R	1	LYLVARVRSL	8	1	454.42	720.211	0.32	0.3	0	0	0	0	0.178	NoExpr	Pending	10	36828795	36828796	36828796	G	A	missense_variant
12-113625613-113625614-C-G		1		Ighv5-6	S82T	1	SYTYYPDTV	8	1	454.62	778.45	2.1	2.5	0	0	0	0	0.009	NoExpr	Pending	12	113625613	113625614	113625614	C	G	missense_variant
1-174449438-174449439-C-T		4		Olfr220	A272V	1	TYARPKLMYV	10	4	455.08	1824.25	0.2	1.7	0	0	0	0	0.589	NoExpr	Pending	1	174449438	174449439	174449439	C	T	missense_variant
12-113578606-113578607-C-T		1	1	Ighv5-2	E84K	1	DTMKRRFII	4	2	455.86	581.875	2.1	2.3	0	0	0	0	0.015	NoExpr	Pending	12	113578606	113578607	113578607	C	T	missense_variant
2-165304933-165304934-G-A		3		Elmo2	A241V	1	YAIALINVL	8	3	456.69	356.074	1.4	0.78	38.532	0	0	0	0.15	NoExpr	Pending	2	165304933	165304934	165304934	G	A	missense_variant
7-137459263-137459264-G-A		1	2	Glrx3	G209E	1	YPQLYVSEEL	8	3	457.83	599.55	0.6	0.5	141.34	0	0	0	0.276	NoExpr	Pending	7	137459263	137459264	137459264	G	A	missense_variant
17-34157316-34157317-G-T		1	1	H2-DMb1	V137F	1	EPVMLACYF	9	2	457.96	6439.17	1.3	5.4	5.829	0	0	0	0.101	NoExpr	Pending	17	34157316	34157317	34157317	G	T	missense_variant
17-34148621-34148622-G-T		1	1	H2-DMb2	V137F	1	EPVMLACYF	9	2	457.96	6439.17	1.3	5.4	6.663	0	0	0	0.031	NoExpr	Pending	17	34148621	34148622	34148622	G	T	missense_variant
4-21683512-21683513-A-G		1		Prdm13	V149A	1	FYRALRDAQ	8	1	458.42	839.963	1.4	2.5	0	0	0	0	0.111	NoExpr	Pending	4	21683512	21683513	21683513	A	G	missense_variant
12-113306429-113306430-A-G		1		Ighg2b	Y324H	1	KNHYLKKTI	3	1	459.29	337.357	1.4	1.4	0.046	0	0	0	0.056	NoExpr	Pending	12	113306429	113306430	113306430	A	G	missense_variant
8-22669668-22669669-C-G		1		Ikbkb	W434C	1	VWGQVCHSI	6	1	460.53	710.003	1.2	1.2	24.878	0	0	0	0.054	NoExpr	Pending	8	22669668	22669669	22669669	C	G	missense_variant
7-41288151-41288152-T-C		1		Gm5592	V286A	1	NTCAQVSQI	4	1	461.65	647.277	2.301	2.8	0	0	0	0	0.053	NoExpr	Pending	7	41288151	41288152	41288152	T	C	missense_variant
2-55437488-55437489-T-G		2		Kcnj3	F97V	1	TYTVAWLVM	8	2	462.66	623.864	1.3	1.5	0	0	0	0	0.213	NoExpr	Pending	2	55437488	55437489	55437489	T	G	missense_variant
6-71625787-71625788-G-A		1		Kdm3a	P105S	1	SPEVSEQVI	5	1	465.68	1534.94	2.1	5.3	40.947	0	0	0	0.065	NoExpr	Pending	6	71625787	71625788	71625788	G	A	missense_variant
9-3002219-3002220-T-A		1	2	Gm10722	S181T	1	SPYTRSYSVH	4	3	466.68	451.771	0.7	0.7	0.048	0	0	0	0.077	NoExpr	Pending	9	3002219	3002220	3002220	T	A	missense_variant
17-33999943-33999944-G-A		2	2	H2-K1	R42W	1	RPGLGEPWYM	8	4	466.68	6044.62	0.7	3.1	899.72	0	0	0	0.138	NoExpr	Pending	17	33999943	33999944	33999944	G	A	missense_variant
17-35265980-35265981-G-A		1		H2-D1	V273M	1	KWASVVMPL	7	1	468.02	591.916	0.8	1.2	1425.5	0	0	0	0.057	NoExpr	Pending	17	35265980	35265981	35265981	G	A	missense_variant
1-105741328-105741329-G-A		2		Relch	G1028D	1	FDTIMETVI	2	2	468.91	287.799	1.4	0.8	20.15	0	0	0	0.21	NoExpr	Pending	1	105741328	105741329	105741329	G	A	missense_variant
7-15555545-15555546-G-A		3	1	Obox1	G87S	1	KYVNKQTSPM	8	4	471.42	1410.05	0.49	1.4	0	0	0	0	0.1	NoExpr	Pending	7	15555545	15555546	15555546	G	A	missense_variant
17-17893094-17893095-C-G		1		Fpr2	L118V	1	AVIALDRCI	2	1	471.75	462.658	2.4	2.1	17.788	0	0	0	0.029	NoExpr	Pending	17	17893094	17893095	17893095	C	G	missense_variant
7-105600065-105600066-T-G		1		Hpx	N9H	1	ARTAVALHI	8	1	471.75	573.186	2.1	2.9	0.09	0	0	0	0.19	NoExpr	Pending	7	105600065	105600066	105600066	T	G	missense_variant
15-82042242-82042243-G-A		2		Snu13	R84C	1	PYVFVCSKQAL	6	2	471.93	501.049	1.2	1.2	248.37	0	0	0	0.024	NoExpr	Pending	15	82042242	82042243	82042243	G	A	missense_variant
15-55212194-55212195-G-A		1		Deptor	S265N	1	SFMSVNPSKE	6	1	473.54	460.66	0.63	0.64	2.505	0	0	0	0.913	NoExpr	Pending	15	55212194	55212195	55212195	G	A	missense_variant
12-113306426-113306427-A-G		2	1	Ighg2b	Y325H	1	KNYHLKKTI	4	3	474.53	337.357	1.4	1.4	0.046	0	0	0	0.063	NoExpr	Pending	12	113306426	113306427	113306427	A	G	missense_variant
16-32755656-32755657-T-C		1		Muc4	S1844P	1	SHAPSLSSS	4	1	476.88	480.96	1.3	1.5	0.047	0	0	0	0.01	NoExpr	Pending	16	32755656	32755657	32755657	T	C	missense_variant
17-36168002-36168003-T-C		1		Gm8909	Q90R	1	EYWERETRI	8	1	477.82	298.717	0.8	0.5	8.461	0	0	0	0.029	NoExpr	Pending	17	36168002	36168003	36168003	T	C	missense_variant
17-35439643-35439644-A-G		1		H2-Q7	Q86R	1	EYWERETRI	8	1	477.82	298.717	0.8	0.5	7.924	0	0	0	0.063	NoExpr	Pending	17	35439643	35439644	35439644	A	G	missense_variant
2-88025360-88025361-T-G		1	1	Olfr1161	L213R	1	RIILLSYMF	1	2	478.44	566.018	0.85	2.2	0	0	0	0	0.125	NoExpr	Pending	2	88025360	88025361	88025361	T	G	missense_variant
8-85171760-85171761-T-C		1	2	Cks1brt	L81P	1	HYMIHEPEPH	9	3	479.34	495.119	1.2	1.5	0	0	0	0	0.04	NoExpr	Pending	8	85171760	85171761	85171761	T	C	missense_variant
7-107129167-107129168-G-A		2		Olfr715	T75I	1	SINIVPQAL	2	2	480.94	759.397	2.301	3.7	0	0	0	0	0.265	NoExpr	Pending	7	107129167	107129168	107129168	G	A	missense_variant
8-21734548-21734549-A-G		1		Defa24	I5V	1	KTLVLLSAL	4	1	482.07	685.899	1.6	2.2	0	0	0	0	0.059	NoExpr	Pending	8	21734548	21734549	21734549	A	G	missense_variant
8-21734547-21734549-AA-TG		1		Defa24	LI4-5LV	1	KTLVLLSAL	4	1	482.07	685.899	1.6	2.2	0	0	0	0	0.033	NoExpr	Pending	8	21734547	21734549	21734548	AA	TG	missense_variant
4-88835957-88835958-A-T		2	1	Ifna5	K145I	1	SLLAVRIYF	7	3	482.07	1637.18	1.8	3.3	0	0	0	0	0.02	NoExpr	Pending	4	88835957	88835958	88835958	A	T	missense_variant
13-67683431-67683432-C-A		1		Zfp738	V3F	1	EFGAGSSAI	2	1	483.16	1746.18	1	4.8	6.759	0	0	0	0.025	NoExpr	Pending	13	67683431	67683432	67683432	C	A	missense_variant

ID	H-2-Dd	H-2-Kd	H-2-Ld	Gene	AA Change	Num Passing Transcripts	Best Peptide	Pos	Num Passing Peptides	IC50 MT	IC50 WT	%ile MT	%ile WT	RNA Expr	RNA VAF	Allele Expr	RNA Depth	DNA VAF	Tier	Evaluation	CHROM	START	END	POS	REF_y	ALT_y	biotype
4-3172365-3172366-G-A		2		Vmn1r2	G95E	1	VYISRITREV	9	2	483.57	454.423	0.4	0.6	0	0	0	0	0.024	NoExpr	Pending	4	3172365	3172366	3172366	G	A	missense_variant
12-113597644-113597645-C-T		1		Ighv5-4	A52T	1	SYTMSWVRQ	3	1	484.27	384.671	2.4	1.7	0	0	0	0	0.046	NoExpr	Pending	12	113597644	113597645	113597645	C	T	missense_variant
2-87034020-87034021-C-G		2	3	Olfr1105	A67P	1	KYFFLTHLP	9	5	484.27	791.152	1.2	2.1	0	0	0	0	0.154	NoExpr	Pending	2	87034020	87034021	87034021	C	G	missense_variant
16-32755090-32755091-T-C		2	1	Muc4	I1655T	1	APITSTQML	6	2	485.39	478.729	2.301	2.2	0.047	0	0	0	0.028	NoExpr	Pending	16	32755090	32755091	32755091	T	C	missense_variant
13-89607947-89607948-A-C		1		Hapln1	K290N	1	AQIANVGQI	5	1	487.32	1358.6	1.3	3.8	0	0	0	0	0.342	NoExpr	Pending	13	89607947	89607948	89607948	A	C	missense_variant
10-89506605-89506606-T-C		1		Nr1h4	Q4R	1	RFQGLENPI	1	1	487.85	579.549	0.69	1.2	0	0	0	0	0.2	NoExpr	Pending	10	89506605	89506606	89506606	T	C	missense_variant
17-18597846-18597847-T-C		1		Vmn2r96	M562T	1	GYLCFTAL	6	1	490.3	2063.33	0.5	1.2	0	0	0	0	0.029	NoExpr	Pending	17	18597846	18597847	18597847	T	C	missense_variant
7-26666093-26666094-C-T		1		Cyp2b23	E439K	1	KRICLGKGI	7	1	491.2	332.859	2.199	1.3	0	0	0	0	0.025	NoExpr	Pending	7	26666093	26666094	26666094	C	T	missense_variant
7-9986483-9986484-G-T		2		Vmn2r49	S360Y	1	NYEDSASNCK	2	2	493.08	5418.51	0.69	22	0	0	0	0	0.049	NoExpr	Pending	7	9986483	9986484	9986484	G	T	missense_variant
17-37590017-37590018-T-C		1		Olfr114	M112V	1	WFAWGEVAI	7	1	497.84	350.825	1.2	0.95	0	0	0	0	0.017	NoExpr	Pending	17	37590017	37590018	37590018	T	C	missense_variant
18-22920876-22920877-G-C		1		Nol4	P193A	1	DYNMAITMAYN	5	1	497.98	828.057	1.7	1.7	3.656	0	0	0	0.043	NoExpr	Pending	18	22920876	22920877	22920877	G	C	missense_variant
5-16302320-16302321-A-C		1	1	Cacna2d1	K356T	1	RANCNTIIM	6	2	498.88	431.519	1.3	1.7	11.374	0	0	0	0.097	NoExpr	Pending	5	16302320	16302321	16302321	A	C	missense_variant
17-20776350-20776351-C-T		1	1	Vmn1r228	A302T	1	WWVMNITTI	8	2	498.99	526.126	0.9	1.7	0	0	0	0	0.381	NoExpr	Pending	17	20776350	20776351	20776351	C	T	missense_variant
16-32752532-32752533-A-G		1	1	Muc4	T804A	1	APITSTQIL	1	1	500.04	531.377	2	1.3	0.047	0	0	0	0.19	NoExpr	Pending	16	32752532	32752533	32752533	A	G,T	missense_variant
7-103517987-103517988-A-C		1		Olfr611	L132R	1	RHYHSILTDTV	1	1	500.85	433.221	0.4	0.5	0	0	0	0	0.191	NoExpr	Pending	7	103517987	103517988	103517988	A	C	missense_variant
7-103538837-103538838-A-C		1		Olfr612	L132R	1	RHYHSILTDTV	1	1	500.85	433.221	0.4	0.5	0	0	0	0	0.063	NoExpr	Pending	7	103538837	103538838	103538838	A	C	missense_variant
7-45881541-45881542-T-A		1		Kdelr1	L132M	1	IYLESVAIM	9	1	502.45	338.135	1.2	0.7	186.43	0	0	0	0.217	NoExpr	Pending	7	45881541	45881542	45881542	T	A	missense_variant
1-182748902-182748903-G-C		2		Ccdc185	P74A	1	GYMTLARES	6	2	502.64	604.074	1.2	1.2	0	0	0	0	0.17	NoExpr	Pending	1	182748902	182748903	182748903	G	C	missense_variant
9-3359583-3359584-C-T		1		Alkbh8	T291I	1	RYLWIHGITPR	5	1	503.39	340.993	1	0.7	7.268	0	0	0	0.328	NoExpr	Pending	9	3359583	3359584	3359584	C	T	missense_variant
X-64178266-64178267-T-C		2		3830417A13Rik	F276S	1	SYASIVRQD	1	2	503.61	1130.47	1.8	2.5	0.113	0	0	0	0.368	NoExpr	Pending	X	64178266	64178267	64178267	T	C	missense_variant
11-58529560-58529561-A-G		1		Olfr330	Y142H	1	RYPVLMNHRV	8	1	503.78	767.88	0.2	0.6	0	0	0	0	0.025	NoExpr	Pending	11	58529560	58529561	58529561	A	G	missense_variant
3-40721631-40721632-T-C		1	1	Slc25a31	S203P	1	PYFGAYDTV	1	2	503.85	29.75	0.5	0.2	0.18	0	0	0	0.178	NoExpr	Pending	3	40721631	40721632	40721632	T	C	missense_variant
7-111079319-111079320-T-G		1		Eif4g2	K108N	1	SNLILKGVI	2	1	504.77	863.495	1.6	2.6	423.73	0	0	0	0.359	NoExpr	Pending	7	111079319	111079320	111079320	T	G	missense_variant
5-15030318-15030319-G-T		1		Gm17019	L160I	1	SRLLMEENI	9	1	506.13	1476.59	1.4	3.8	0	0	0	0	0.035	NoExpr	Pending	5	15030318	15030319	15030319	G	T	missense_variant
5-15473119-15473120-G-T		1		Gm21149	L160I	1	SRLLMEENI	9	1	506.13	1476.59	1.4	3.8	0.074	0	0	0	0.049	NoExpr	Pending	5	15473119	15473120	15473120	G	T	missense_variant
5-110893009-110893010-T-C		1		Ttc28	L88S	1	SYSNRSAAYM	1	1	507.92	2555.26	0.71	2.8	4.164	0	0	0	0.211	NoExpr	Pending	5	110893009	110893010	110893010	T	C	missense_variant
12-115294364-115294365-A-G		1		Ighv8-8	S19P	1	AYVLPQVTL	5	1	508.27	154.202	1.2	0.5	0	0	0	0	0.046	NoExpr	Pending	12	115294364	115294365	115294365	A	G	missense_variant
17-35380445-35380446-C-A		1		H2-Q4	D168E	1	AYEGRDYI	3	1	509.96	1112.95	0.71	1.4	256.73	0	0	0	0.032	NoExpr	Pending	17	35380445	35380446	35380446	C	A	missense_variant
11-77472850-77472851-C-T		1		Ankrd13b	A429T	1	IFHILNTRI	7	1	510.49	1763.03	1.2	5.5	15.361	0	0	0	0.216	NoExpr	Pending	11	77472850	77472851	77472851	C	T	missense_variant
7-7472558-7472559-T-C		1		Vmn2r32	T442A	1	AYFTNPLGDKV	1	1	511.54	487.394	1.7	1.4	0	0	0	0	0.023	NoExpr	Pending	7	7472558	7472559	7472559	T	C	missense_variant
1-152843263-152843264-C-T		1		Smg7	G912D	1	LYPALLDPL	7	1	511.99	325.282	1.5	0.9	26.68	0	0	0	0.64	NoExpr	Pending	1	152843263	152843264	152843264	C	T	missense_variant
5-15529187-15529188-A-T		1		Gm21190	F11I	1	LFARLCRLI	9	1	514.15	9960.02	1.3	21	0	0	0	0	0.12	NoExpr	Pending	5	15529187	15529188	15529188	A	T	missense_variant
7-18890252-18890253-G-A		1		Pglyrp1	V154I	1	GFLRSNYEI	9	1	515.24	1534.27	1.6	7.3	2.893	0	0	0	0.74	NoExpr	Pending	7	18890252	18890253	18890253	G	A	missense_variant
7-85624241-85624242-C-G		2		Vmn2r71	L755V	1	GYMGSVAL	6	2	517.9	371.57	0.71	0.55	0	0	0	0	0.021	NoExpr	Pending	7	85624241	85624242	85624242	C	G	missense_variant
16-31127336-31127337-A-G		1		Acap2	F281L	1	GYLFKRASNAL	11	1	519.9	3704.33	0.7	2.1	39.65	0	0	0	0.287	NoExpr	Pending	16	31127336	31127337	31127337	A	G	missense_variant
6-128559068-128559069-T-C		1		A2ml1	H720R	1	SISRAKVAI	4	1	520	794.804	2.5	2.8	0	0	0	0	0.223	NoExpr	Pending	6	128559068	128559069	128559069	T	C	missense_variant
2-86508576-86508578-GG-AA		1	1	Olfr1076	LV39-40LM	1	YLITLMGNL	6	2	520	585.724	2.301	1.8	0	0	0	0	0.027	NoExpr	Pending	2	86508576	86508578	86508577	GG	AA	missense_variant
6-136328830-136328831-C-T		1		Eif4a3l1	L97F	1	FSISVFQCL	6	1	522.5	524.916	1.7	1.7	0	0	0	0	0.338	NoExpr	Pending	6	136328830	136328831	136328831	C	T	missense_variant
10-129387749-129387750-G-T		1	1	Olfr784	G39V	1	NYILSLMVN	8	2	522.71	682.747	1.7	2.2	0	0	0	0	0.11	NoExpr	Pending	10	129387749	129387750	129387750	G	T	missense_variant
2-49947546-49947547-C-T		1	1	Lypd6b	P177L	1	LYLLVLAWL	1	2	523.91	1274.27	1.2	1.7	0	0	0	0	0.42	NoExpr	Pending	2	49947546	49947547	49947547	C	T	missense_variant
16-32751873-32751874-C-T		1		Muc4	T584I	1	KGTSSNPQI	9	1	525.66	7366.35	2.699	27	0.047	0	0	0	0.008	NoExpr	Pending	16	32751873	32751874	32751874	C	T	missense_variant
6-39522875-39522876-G-A		2		Dennd2a	P252S	1	RWYPPKSFI	7	2	528.56	3484.1	1.7	8.8	1.045	0	0	0	0.936	NoExpr	Pending	6	39522875	39522876	39522876	G	A	missense_variant
4-43835632-43835634-GT-CC		3		Olfr157	GL285-286GV	1	FYGVLTPML	4	3	529.77	807.402	1.5	1.9	0	0	0	0	0.054	NoExpr	Pending	4	43835632	43835634	43835633	GT	CC	missense_variant
7-85955954-85955955-G-A		1	1	Vmn2r74	S495F	1	IYMFKEMI	4	2	529.79	247.55	0.69	0.2	0	0	0	0	0.031	NoExpr	Pending	7	85955954	85955955	85955955	G	A	missense_variant
2-86181567-86181568-T-C		2		Olfr52	D181G	1	FYCDGMPLLRL	5	2	531.38	1145.59	1	2.1	0	0	0	0	0.209	NoExpr	Pending	2	86181567	86181568	86181568	T	C	missense_variant
9-124125121-124125122-A-C		1	2	Ccr5	N254T	1	TPYTIVLLL	4	3	531.9	514.71	2.1	1.4	40.848	0	0	0	0.036	NoExpr	Pending	9	124125121	124125122	124125122	A	C	missense_variant
1-85610881-85610882-G-C		1	1	Sp140	K113N	1	AYPDLNETL	6	2	532.07	565.98	1.6	1.2	95.257	0	0	0	0.193	NoExpr	Pending	1	85610881	85610882	85610882	G	C,T	missense_variant
18-23569662-23569663-G-C		1		Dtna	V84L	1	RLEALLSTI	5	1	533.65	317.147	1.6	0.9	3.999	0	0	0	0.162	NoExpr	Pending	18	23569662	23569663	23569663	G	C	missense_variant
2-172551490-172551491-C-T		1		Tfap2c	P84S	1	LYSPAPSLS	7	1	534.68	4594.73	1.8	8.8	0	0	0	0	0.383	NoExpr	Pending	2	172551490	172551491	172551491	C	T	missense_variant
11-103455479-103455480-T-C		1		Lrrc37a	I3187V	1	SVTVVVTVL	5	1	535.91	1455.76	2.6	3.9	0	0	0	0	0.056	NoExpr	Pending	11	103455479	103455480	103455480	T	C	missense_variant
1-93024632-93024633-G-A		2		Kif1a	A1308V	1	AKLPVSRSI	5	2	536.12	1133.08	1.8	2.4	0.066	0	0	0	0.304	NoExpr	Pending	1	93024632	93024633	93024633	G	A	missense_variant
13-106976817-106976818-G-C		1		Kif2a	S407R	1	RRHAVFQII	2	1	537.14	1032.98	1.8	1.7	26.02	0	0	0	0.039	NoExpr	Pending	13	106976817	106976818	106976818	G	C	missense_variant
3-130631732-130631733-G-T		2		Etnppl	G396W	1	YEMKGKWVL	7	2	537.16	437.342	1.7	2.2	0	0	0	0	0.178	NoExpr	Pending	3	130631732	130631733	130631733	G	T	missense_variant
12-114538612-114538613-T-C		1		Ighv1-7	K78R	1	GYINPSSGYTR	11	1	537.16	326.498	1.4	0.8	0	0	0	0	0.106	NoExpr	Pending	12	114538612	114538613	114538613	T	C	missense_variant
2-89193310-89193311-G-T		2		Olfr1226	S241Y	1	GYHITVVL	2	2	537.16	23699.6	0.56	25	0	0	0	0	0.218	NoExpr	Pending	2	89193310	89193311	89193311	G	T	missense_variant
7-140691593-140691594-C-T		1		Olfr45	R230C	1	SILRMCSAE	6	1	537.16	475.434	2.699	2.3	0	0	0	0	0.029	NoExpr	Pending	7	140691593	140691594	140691594	C	T	missense_variant
12-113736297-113736298-C-T		2		Ighv5-9-1	V65I	1	SYAMSWIRQ	7	2	539.62	384.671	2.5	1.7	0	0	0	0	0.03	NoExpr	Pending	12	113736297	113736298	113736298	C	T	missense_variant
10-82284746-82284747-C-G		1		4932415D10Rik	R4143T	1	IQQYSGTTL	8	1	539.83	1923.95	1.8	2.8	0	0	0	0	0.24	NoExpr	Pending	10	82284746	82284747	82284747	C	G	missense_variant
1-88359685-88359686-T-A		2		Trpm8	L797Q	1	NYFTDQWNV	6	2	542.7	482.072	2.199	2.3	0	0	0	0	0.034	NoExpr	Pending	1	88359685	88359686	88359686	T	A	missense_variant
9-3001415-3001416-G-C		1		Gm10722	W164S	1	FHVLQSTFL	6	1	543	1214.86	2.801	2.8	0.048	0	0	0	0.029	NoExpr	Pending	9	3001415	3001416	3001416	G	C	missense_variant
10-51725419-51725420-C-T		1		Rfx6	P646S	1	SAIASRGSV	1	1	543	3724.69	1.5	9.1	0	0	0	0	0.501	NoExpr	Pending	10	51725419	51725420	51725420	C	T	missense_variant
7-26611440-26611441-A-T		1		Vmn1r185	F213Y	1	LYKHKKKVQHI	2	1	543	4989.87	1	6.1	0	0	0	0	0.098	NoExpr	Pending	7	26611440	26611441	26611441	A	T	missense_variant

ID	H-2-Dd	H-2-Kd	H-2-Ld	Gene	AA Change	Num Passing Transcripts	Best Peptide	Pos	Num Passing Peptides	IC50 MT	IC50 WT	%ile MT	%ile WT	RNA Expr	RNA VAF	Allele Expr	RNA Depth	DNA VAF	Tier	Evaluation	CHROM	START	END	POS	REF_y	ALT_y	biotype
13-23217383-23217384-G-T		2	1	Vmn1r221	K4N	1	NYINKIIFLFM	1	3	543	306.024	1	0.6	0	0	0	0	0.146	NoExpr	Pending	13	23217383	23217384	23217384	G	T	missense_variant
12-114416642-114416643-T-C		2		Ighv6-5	M85V	1	VYLQVNSLR	5	2	544.62	387.338	1.8	0.91	0	0	0	0	0.224	NoExpr	Pending	12	114416642	114416643	114416643	T	C	missense_variant
1-107271843-107271844-T-C		1		Serpinb3c	S316G	1	SGMGSTQGL	4	1	544.83	359.948	1.8	0.9	0	0	0	0	0.229	NoExpr	Pending	1	107271843	107271844	107271844	T	C	missense_variant
11-99350948-99350949-G-A		1		Krt27	A42V	1	SCVFGGSSL	3	1	545.87	275.48	2.199	1.1	0	0	0	0	0.341	NoExpr	Pending	11	99350948	99350949	99350949	G	A	missense_variant
1-85591681-85591682-G-T		1		Sp110	P142T	1	TQISLPSHL	1	1	548.39	1746.18	1.3	2.6	36.982	0	0	0	0.048	NoExpr	Pending	1	85591681	85591682	85591682	G	T	missense_variant
14-4558360-4558361-C-G		1		Gm3047	I194M	1	KNMCASSAK	3	1	548.61	392.726	2.1	1.5	0	0	0	0	0.071	NoExpr	Pending	14	4558360	4558361	4558361	C	G	missense_variant
7-38522134-38522135-G-C		1		Gm5591	P170R	1	GYSLARSYQ	6	1	548.61	773.143	0.9	2	0	0	0	0	0.079	NoExpr	Pending	7	38522134	38522135	38522135	G	C	missense_variant
7-39408114-39408115-G-C		1		Gm5114	H693Q	1	SQSAQPTTL	2	1	548.91	228.498	1.3	0.6	0	0	0	0	0.034	NoExpr	Pending	7	39408114	39408115	39408115	G	C	missense_variant
17-36167948-36167949-T-C		1		Gm8909	H108R	1	RYYNQSAGGSI	1	1	548.91	666.931	1.1	1.2	8.461	0	0	0	0.054	NoExpr	Pending	17	36167948	36167949	36167949	T	C	missense_variant
7-5480932-5480933-C-A		1		Vmn2r28	C756F	1	FYCVLGYLAFL	10	1	548.91	828.057	1.1	1.5	0	0	0	0	0.035	NoExpr	Pending	7	5480932	5480933	5480933	C	A	missense_variant
7-10158943-10158944-C-A		1		Vmn2r52	C756F	1	FYCVLGYLAFL	10	1	548.91	828.057	1.1	1.5	0	0	0	0	0.044	NoExpr	Pending	7	10158943	10158944	10158944	C	A	missense_variant
12-58212757-58212758-G-A		2		Sstr1	G56S	1	SSAILISFI	1	2	549.66	1718.26	1.1	2.3	0	0	0	0	0.336	NoExpr	Pending	12	58212757	58212758	58212758	G	A	missense_variant
8-79690418-79690419-T-G		1		Abce1	N321T	1	GYVPTETL	7	1	549.69	1319.98	0.58	1.7	124.81	0	0	0	0.191	NoExpr	Pending	8	79690418	79690419	79690419	T	G	missense_variant
2-62764576-62764577-C-T		1	1	Kcnh7	M716I	1	TYTNGIDINMV	8	2	550.44	552.981	1.3	1.3	0.008	0	0	0	0.383	NoExpr	Pending	2	62764576	62764577	62764577	C	T	missense_variant
1-161060750-161060751-G-A		1	1	Dars2	L200F	1	MVMKMREYF	9	2	552.08	774.462	2.199	2.9	18.783	0	0	0	0.609	NoExpr	Pending	1	161060750	161060751	161060751	G	A	missense_variant
7-103506429-103506430-G-A		1	1	Olfr610	P172L	1	LLGRLTFQL	9	2	552.08	8912.51	2.199	18	0	0	0	0	0.248	NoExpr	Pending	7	103506429	103506430	103506430	G	A	missense_variant
1-84964326-84964327-G-A		1		AC167036.1	R220Q	1	SNIKAQRTV	6	1	552.41	380.417	1.7	1.1	0	0	0	0	0.016	NoExpr	Pending	1	84964326	84964327	84964327	G	A	missense_variant
7-85150570-85150571-A-C		1	1	Vmn2r67	H486Q	1	LPSQQQLYM	4	2	553.13	794.017	2.199	2.9	0	0	0	0	0.287	NoExpr	Pending	7	85150570	85150571	85150571	A	C	missense_variant
7-85410257-85410258-T-C		1		Vmn2r69	E481G	1	GHLPSGQQL	1	1	556.6	1714.31	1.5	3	0	0	0	0	0.028	NoExpr	Pending	7	85410257	85410258	85410258	T	C	missense_variant
15-80918576-80918577-A-C		1	1	Tnrc6b	N1527T	1	PYSASDTSF	7	2	559.86	964.029	1.8	2.7	9.651	0	0	0	0.086	NoExpr	Pending	15	80918576	80918577	80918577	A	C	missense_variant
1-171530501-171530502-G-C		3		Itln1	S226C	1	YYSPCGQRE	5	3	560.09	136.855	1.9	0.5	0.19	0	0	0	0.064	NoExpr	Pending	1	171530501	171530502	171530502	G	C,T	missense_variant
18-36004746-36004747-C-T		1		Psd2	A464V	1	KNEKLEWVI	8	1	560.92	437.342	2.801	2.2	0	0	0	0	0.061	NoExpr	Pending	18	36004746	36004747	36004747	C	T	missense_variant
13-92131447-92131448-G-A		1		Rasgrf2	A16V	1	RYNEGHALYLV	11	1	560.92	2289.62	1.1	3.7	0.164	0	0	0	0.676	NoExpr	Pending	13	92131447	92131448	92131448	G	A	missense_variant
12-114746399-114746400-C-T		1		Ighv1-22	G75D	1	GYINPNNDGTS	8	1	562.19	562.186	2.199	2.2	0	0	0	0	0.038	NoExpr	Pending	12	114746399	114746400	114746400	C	T	missense_variant
1-159324957-159324958-C-G		1		Cop1	P593A	1	YYDLRNTKQAI	10	1	562.34	340.97	0.54	0.4	39.306	0	0	0	0.14	NoExpr	Pending	1	159324957	159324958	159324958	C	G	missense_variant
2-88794939-88794940-C-T		1	2	Olfr1201	A186V	1	QPLLKLVCM	7	3	564.72	1209.27	1.2	2.5	0	0	0	0	0.377	NoExpr	Pending	2	88794939	88794940	88794940	C	T	missense_variant
8-21096001-21096002-C-T		1		Defa38	V17I	1	AFQIQADPI	4	1	565.05	353.256	1.2	0.7	0	0	0	0	0.025	NoExpr	Pending	8	21096001	21096002	21096002	C	T	missense_variant
1-85099520-85099521-A-G		1		A530032D15Rik	V108A	1	AYPDLKETL	1	1	565.98	1112.4	1.2	1.6	24.026	0	0	0	0.078	NoExpr	Pending	1	85099520	85099521	85099521	A	G	missense_variant
10-34403686-34403687-C-T		1		Nt5dc1	D120N	1	SNKPGVSDI	2	1	566.36	1032.98	2.4	4.2	20.263	0	0	0	0.516	NoExpr	Pending	10	34403686	34403687	34403687	C	T	missense_variant
4-88571258-88571259-C-G		1		Ifna14	K180N	1	SSANLLTSL	4	1	568.97	548.908	1.7	0.8	0	0	0	0	0.014	NoExpr	Pending	4	88571258	88571259	88571259	C	G	missense_variant
12-32205628-32205629-C-T		2		Pik3cg	D120N	1	RYQVVQTLNCL	9	2	571.1	461.61	0.6	0.64	8.963	0	0	0	0.351	NoExpr	Pending	12	32205628	32205629	32205629	C	T	missense_variant
7-7384870-7384871-G-C		1		Vmn2r31	T567R	1	QYANREQNKC	5	1	571.32	711.017	1.6	1	0	0	0	0	0.015	NoExpr	Pending	7	7384870	7384871	7384871	G	C	missense_variant
7-8368345-8368346-G-C		1		Vmn2r44	T567R	1	QYANREQNKC	5	1	571.32	711.017	1.6	1	0	0	0	0	0.042	NoExpr	Pending	7	8368345	8368346	8368346	G	C	missense_variant
12-104130483-104130484-G-T		1		Serpina3b	G8V	1	AFIAALVLL	7	1	573.14	282.657	1.7	0.8	0	0	0	0	0.025	NoExpr	Pending	12	104130483	104130484	104130484	G	T	missense_variant
9-119932693-119932694-C-T		1	1	Gorasp1	G91D	1	SNMWGDQGL	6	2	573.19	567.017	2.9	1.8	9.183	0	0	0	0.3	NoExpr	Pending	9	119932693	119932694	119932694	C	T	missense_variant
17-43087957-43087958-C-T		1		Tnfrsf21	P652S	1	VYSHLSDLL	6	1	574.24	615.304	1.9	2	5.445	0	0	0	0.367	NoExpr	Pending	17	43087957	43087958	43087958	C	T	missense_variant
X-74303850-74303851-G-A		1		Atp6ap1	G413S	1	SYASDCASF	8	1	575.34	1535.54	2.1	4.2	194.82	0	0	0	0.041	NoExpr	Pending	X	74303850	74303851	74303851	G	A	missense_variant
17-69287624-69287625-C-A		1		Epb41l3	A898E	1	HDQALAQEI	8	1	575.56	264.296	1.9	0.8	0.268	0	0	0	0.079	NoExpr	Pending	17	69287624	69287625	69287625	C	A	missense_variant
7-23984786-23984787-G-A		1		Vmn1r181	D226N	1	QYIFTLNQN	9	1	575.56	771.063	1.9	2.3	0	0	0	0	0.022	NoExpr	Pending	7	23984786	23984787	23984787	G	A	missense_variant
15-89190248-89190249-C-G		2		Dennd6b	A122P	1	SYNNKAPLPL	9	2	576.23	772.64	0.8	0.99	0.8	0	0	0	0.093	NoExpr	Pending	15	89190248	89190249	89190249	C	G	missense_variant
17-37590035-37590036-A-C		1	1	Olfr114	W106G	1	GFAWGEMAI	1	2	576.89	350.825	1.2	0.95	0	0	0	0	0.015	NoExpr	Pending	17	37590035	37590036	37590036	A	C	missense_variant
7-100878819-100878820-C-G		2		Arhgef17	G1738A	1	AYQSSVWLA	9	2	577.11	751.779	1.5	1.4	16.501	0	0	0	0.239	NoExpr	Pending	7	100878819	100878820	100878820	C	G	missense_variant
18-78109935-78109936-T-G		1	1	Slc14a1	I313L	1	LHAALGSLL	5	2	577.11	1023.51	1.7	1.7	60.892	0	0	0	0.071	NoExpr	Pending	18	78109935	78109936	78109936	T	G	missense_variant
7-86171680-86171681-T-C		1		Vmn2r75	K15R	1	FWFLRISII	5	1	578.44	1633.37	1.5	3	0	0	0	0	0.082	NoExpr	Pending	7	86171680	86171681	86171681	T	C	missense_variant
14-67724570-67724571-A-G		1		Kctd9	Y21C	1	KVVAVCGTL	6	1	579.42	579.421	1.4	2	29.141	0	0	0	0.031	NoExpr	Pending	14	67724570	67724571	67724571	A	G	missense_variant
4-24596142-24596143-C-T		1		Mms22l	S1074F	1	FYLEFKGSSPF	1	1	579.42	611.631	1.1	0.7	13.068	0	0	0	0.225	NoExpr	Pending	4	24596142	24596143	24596143	C	T	missense_variant
4-131799653-131799654-A-G		2		Ptpru	L669P	1	HYFGAEPA	7	2	579.42	855.376	0.9	1.2	0.017	0	0	0	0.268	NoExpr	Pending	4	131799653	131799654	131799654	A	G	missense_variant
14-53105270-53105271-G-A		1		Trav6n-5	A89T	1	TYNKETTSFHL	6	1	579.42	537.157	1.1	1	0	0	0	0	0.027	NoExpr	Pending	14	53105270	53105271	53105271	G	A	missense_variant
14-53133063-53133064-G-A		1		Trav6n-6	A91T	1	TYNKETTSFHL	6	1	579.42	537.157	1.1	1	0.126	0	0	0	0.151	NoExpr	Pending	14	53133063	53133064	53133064	G	A	missense_variant
16-32754592-32754593-C-G		1		Muc4	T1489S	1	LSSTSSSQI	7	1	580.89	847.403	1.9	2.1	0.047	0	0	0	0.031	NoExpr	Pending	16	32754592	32754593	32754593	C	G	missense_variant
8-125447857-125447858-C-T		1		Sipa1l2	S1227N	1	SSHSSNNTL	6	1	580.89	587.611	0.96	0.9	1.664	0	0	0	0.098	NoExpr	Pending	8	125447857	125447858	125447858	C	T	missense_variant
7-5488449-5488450-G-A		1		Vmn2r28	S266L	1	IMMLSTNVI	4	1	580.95	238.281	1.4	0.7	0	0	0	0	0.032	NoExpr	Pending	7	5488449	5488450	5488450	G	A	missense_variant
10-27459393-27459394-A-C		1		Lama2	I95S	1	QRHPSTNAI	5	1	581.11	2052.39	1.9	3.8	0.359	0	0	0	0.124	NoExpr	Pending	10	27459393	27459394	27459394	A	C	missense_variant
2-129609071-129609072-G-A		1		Sirpa	G84E	1	IYSFAEEYV	6	1	581.92	338.8	0.77	0.51	52.411	0	0	0	0.421	NoExpr	Pending	2	129609071	129609072	129609072	G	A	missense_variant
5-110379310-110379311-G-A		1		Fbrsl1	P330L	1	LGQVASSSL	1	1	582.22	1853.92	1.9	3.9	14.405	0	0	0	0.068	NoExpr	Pending	5	110379310	110379311	110379311	G	A	missense_variant
7-65663890-65663891-G-A		1		Tarsl2	E353K	1	TYWKGNPEM	4	1	585.18	1059.65	2	2.4	13.554	0	0	0	0.601	NoExpr	Pending	7	65663890	65663891	65663891	G	A	missense_variant
2-130041822-130041823-G-C		1		Tgm3	G467A	1	SFAATSSRN	3	1	585.72	1485.27	3	7.1	0.014	0	0	0	0.113	NoExpr	Pending	2	130041822	130041823	130041823	G	C	missense_variant
14-55105965-55105966-G-A		2	1	Ap1g2	L55F	1	FYVHMLGYP	1	3	587.84	966.251	1.2	2.5	10.541	0	0	0	0.123	NoExpr	Pending	14	55105965	55105966	55105966	G	A	missense_variant
11-98250138-98250139-C-T		1		Cdk12	T1402I	1	RFIRVPLAL	3	1	587.96	1239.3	1	2.5	14.32	0	0	0	0.117	NoExpr	Pending	11	98250138	98250139	98250139	C	T	missense_variant
X-104565824-104565825-G-C		2		Zdhhc15	P205A	1	KYWRGELASV	8	2	588.36	767.658	0.4	1.1	0.704	0	0	0	0.178	NoExpr	Pending	X	104565824	104565825	104565825	G	C	missense_variant
17-23291036-23291037-C-T		1		Vmn2r114	G823E	1	SSAGMLECI	7	1	591.68	411.235	1.9	1.4	0	0	0	0	0.066	NoExpr	Pending	17	23291036	23291037	23291037	C	T	missense_variant
17-23360024-23360025-G-A		1		Vmn2r115	G824E	1	SSAGMLECI	7	1	591.68	411.235	1.9	1.4	0	0	0	0	0.044	NoExpr	Pending	17	23360024	23360025	23360025	G	A	missense_variant

ID	H-2-Dd	H-2-Kd	H-2-Ld	Gene	AA Change	Num Passing Transcripts	Best Peptide	Pos	Num Passing Peptides	IC50 MT	IC50 WT	%ile MT	%ile WT	RNA Expr	RNA VAF	Allele Expr	RNA Depth	DNA VAF	Tier	Evaluation	CHROM	START	END	POS	REF_y	ALT_y	biotype
12-116000157-116000159-TC-GT		1		Ighv1-85	D74T	1	IYPRTGSTK	5	1	591.92	2513.39	1.8	6.4	0	0	0	0	0.023	NoExpr	Pending	12	116000157	116000159	116000158	TC	GT	missense_variant
10-128886413-128886414-C-T		1	1	Gdf11	V191I	1	RPVPRPATIYL	9	2	592.1	884.75	0.42	0.6	0.4	0	0	0	0.189	NoExpr	Pending	10	128886413	128886414	128886414	C	T	missense_variant
17-25242587-25242588-C-T		1		Tsr3	T300I	1	SNYSGAEEI	9	1	594.42	9751.92	1.3	15	27.842	0	0	0	0.046	NoExpr	Pending	17	25242587	25242588	25242588	C	T	missense_variant
3-30659084-30659085-G-A		2		Lrriq4	R445Q	1	LYIENNQLEQL	7	2	595.81	548.125	0.23	0.3	0	0	0	0	0.144	NoExpr	Pending	3	30659084	30659085	30659085	G	A	missense_variant
6-32163415-32163416-C-T		2		Plxna4	R1679Q	1	SEIYLTQLL	7	2	597.16	1371.17	1.7	3.8	0.158	0	0	0	0.791	NoExpr	Pending	6	32163415	32163416	32163416	C	T	missense_variant
12-113306433-113306434-T-G		1	1	Ighg2b	K322N	1	HEGLNNYYL	5	2	598.18	643.917	2.4	2.5	0.046	0	0	0	0.055	NoExpr	Pending	12	113306433	113306434	113306434	T	G	missense_variant
7-11162830-11162831-C-T		1		Zscan4d	G204E	1	SENEMDSLL	2	1	598.54	458.237	1.9	1.4	0	0	0	0	0.019	NoExpr	Pending	7	11162830	11162831	11162831	C	T	missense_variant
9-5321499-5321500-C-T		1		Casp4	H84Y	1	SYHGEANL	2	1	598.54	9338.1	0.38	4.1	38.704	0	0	0	0.292	NoExpr	Pending	9	5321499	5321500	5321500	C	T	missense_variant
12-115208513-115208514-C-T		2		Ighv1-55	C5Y	1	SYIILILVAAA	2	2	598.54	11604	2	20	0	0	0	0	0.017	NoExpr	Pending	12	115208513	115208514	115208514	C	T	missense_variant
4-43835629-43835630-G-C		3		Olfr157	L287V	1	LVTPMLNPI	2	3	602.68	583.566	1.9	1.7	0	0	0	0	0.054	NoExpr	Pending	4	43835629	43835630	43835630	G	C	missense_variant
17-23291793-23291794-A-G		2		Vmn2r114	C571R	1	QYANTEQNHR	10	2	603.34	788.642	0.8	1.2	0	0	0	0	0.044	NoExpr	Pending	17	23291793	23291794	23291794	A	G	missense_variant
9-107025337-107025338-G-C		1		Dock3	A291G	1	LYIVGHVI	5	1	604.36	693.78	0.67	0.46	0	0	0	0	0.038	NoExpr	Pending	9	107025337	107025338	107025338	G	C	missense_variant
12-113879112-113879113-C-T		1		Ighv2-9	M111I	1	SLQTDDTAI	9	1	605.05	2192.64	1.8	5.6	0	0	0	0	0.078	NoExpr	Pending	12	113879112	113879113	113879113	C	T	missense_variant
5-138988374-138988375-A-G		1		Pdgfa	V80A	1	APIRRKRSI	1	1	605.05	1210.85	3	3.4	28.704	0	0	0	0.111	NoExpr	Pending	5	138988374	138988375	138988375	A	G	missense_variant
7-86164094-86164095-C-T		1		Vmn2r75	E500K	1	KWATDMDQI	1	1	607.1	5834.44	2	13	0	0	0	0	0.49	NoExpr	Pending	7	86164094	86164095	86164095	C	T	missense_variant
2-90052006-90052007-C-T		1		Olfr140	G106R	1	AHLLRGTEI	5	1	608.96	267.01	2.5	1.2	0	0	0	0	0.412	NoExpr	Pending	2	90052006	90052007	90052007	C	T	missense_variant
6-57404710-57404711-A-G		1		Vmn1r19	N83S	1	FYISRVMRGL	4	1	609.68	799.89	0.72	0.93	0	0	0	0	0.229	NoExpr	Pending	6	57404710	57404711	57404711	A	G	missense_variant
11-71122820-71122821-A-T		1		Nlrp1a	D534E	1	LYENQEMEL	6	1	609.9	643.073	1.8	1.8	0.059	0	0	0	0.079	NoExpr	Pending	11	71122820	71122821	71122821	A	T	missense_variant
1-97723703-97723704-G-T		1		Ppip5k2	P968Q	1	IHIHRKSQL	8	1	611.07	753.807	1.5	0.8	24.347	0	0	0	0.028	NoExpr	Pending	1	97723703	97723704	97723704	G	T	missense_variant
4-28947572-28947573-A-G		2		Epha7	E615G	1	TYIDPETYGDP	9	2	611.63	767.658	1.2	1.4	0	0	0	0	0.252	NoExpr	Pending	4	28947572	28947573	28947573	A	G	missense_variant
11-73453653-73453654-T-A		1		Olfr380	K186M	1	LLMLACSDI	3	1	614.13	4179.17	2	8	0	0	0	0	0.236	NoExpr	Pending	11	73453653	73453654	73453654	T	A	missense_variant
7-141639453-141639454-C-T		2		Muc6	A1769T	1	PVIYTTSTI	8	2	616.72	650.264	1.8	2.1	0.02	0	0	0	0.066	NoExpr	Pending	7	141639453	141639454	141639454	C	T	missense_variant
1-6810623-6810624-A-G		1		St18	D447G	1	AMTQGKSQL	5	1	616.72	2168.15	2	5.8	0.101	0	0	0	0.239	NoExpr	Pending	1	6810623	6810624	6810624	A	G	missense_variant
12-118931855-118931856-C-T		3		Abcb5	A402T	1	SYPSRPSTKV	8	3	617.55	1223.6	0.5	1.4	0	0	0	0	0.439	NoExpr	Pending	12	118931855	118931856	118931856	C	T	missense_variant
1-166269457-166269458-G-A		1		Ildr2	G82E	1	SYCQDRMEESI	8	1	617.74	254.608	0.5	0.6	0.398	0	0	0	0.578	NoExpr	Pending	1	166269457	166269458	166269458	G	A	missense_variant
X-74303922-74303923-C-G		1		Atp6ap1	L437V	1	IFTYGVHMI	6	1	618.14	616.723	1.2	1.2	194.82	0	0	0	0.055	NoExpr	Pending	X	74303922	74303923	74303923	C	G	missense_variant
3-107213457-107213458-G-A		1		Cym	T277I	1	CPAVLDTGI	9	1	618.28	9185.23	3.1	29	0	0	0	0	0.46	NoExpr	Pending	3	107213457	107213458	107213458	G	A	missense_variant
7-7476901-7476902-G-C		1		Vmn2r32	L91V	1	RYPDVLPNMSL	5	1	618.28	883.597	0.7	0.4	0	0	0	0	0.15	NoExpr	Pending	7	7476901	7476902	7476902	G	C	missense_variant
17-42316784-42316785-C-G		2		Ptchd4	P46A	1	FFLTVAAVLTI	6	2	621.89	756.328	1.5	0.84	0.014	0	0	0	0.116	NoExpr	Pending	17	42316784	42316785	42316785	C	G	missense_variant
2-66508624-66508625-T-A		1		Scn9a	K1172N	1	GNGKVWWTI	2	1	622.43	1202.98	2	3.4	0	0	0	0	0.233	NoExpr	Pending	2	66508624	66508625	66508625	T	A	missense_variant
3-90349472-90349473-A-T		1		Gatad2b	N196I	1	KEIVVQKTPV	3	1	625.01	1989.19	1	3.6	19.306	0	0	0	0.167	NoExpr	Pending	3	90349472	90349473	90349473	A	T	missense_variant
12-103895049-103895050-G-T		1	1	Serpina1c	L402I	1	EEHTQSPIF	8	2	625.01	598.537	3	2.9	0.355	0	0	0	0.04	NoExpr	Pending	12	103895049	103895050	103895050	G	T	missense_variant
15-66541676-66541677-C-G		3		Tmem71	G182A	1	AYILPQSLR	1	3	626.74	1167.51	2	3.3	3.638	0	0	0	0.056	NoExpr	Pending	15	66541676	66541677	66541677	C	G	missense_variant
2-3462551-3462552-C-A		3		Suv39h2	D376Y	1	YYESDEFTV	1	3	626.99	7468.96	2	13	6.309	0	0	0	0.383	NoExpr	Pending	2	3462551	3462552	3462552	C	A	missense_variant
2-98667242-98667243-T-A		1		Gm10800	D20V	1	CHISRPTVV	9	1	629.72	5165.23	2.9	12	1.05	0	0	0	0.013	NoExpr	Pending	2	98667242	98667243	98667243	T	A	missense_variant
15-4912795-4912796-C-T		2		Mroh2b	H347Y	1	NYTTSIEKTVK	2	2	631.81	6591.36	1.4	18	0	0	0	0	0.055	NoExpr	Pending	15	4912795	4912796	4912796	C	T	missense_variant
17-27702736-27702737-A-G		1		Pacsin1	D31G	1	KRIDGGHRL	5	1	632.79	2313.45	2.301	6.2	0.032	0	0	0	0.06	NoExpr	Pending	17	27702736	27702737	27702737	A	G	missense_variant
12-103691891-103691892-C-G		1		Serpina1f	E251D	1	HYLFRVDDL	7	1	632.79	743.464	2.1	2	0	0	0	0	0.203	NoExpr	Pending	12	103691891	103691892	103691892	C	G	missense_variant
1-85610748-85610749-A-G		2		Sp140	E69G	1	EYQGTCKNL	4	2	632.79	748.618	1	1.2	95.257	0	0	0	0.054	NoExpr	Pending	1	85610748	85610749	85610749	A	G	missense_variant
13-100161675-100161677-TG-GA		4	1	Naip2	S617F	1	YLFRTCLLI	3	5	634.25	1125.28	2.1	3.2	22.922	0	0	0	0.029	NoExpr	Pending	13	100161675	100161677	100161676	TG	GA	missense_variant
16-44419262-44419263-G-A		2	1	Cfap44	V558I	1	AELHLKYIF	8	3	638.01	1806.47	2.4	3	0	0	0	0	0.5	NoExpr	Pending	16	44419262	44419263	44419263	G	A	missense_variant
10-83504132-83504133-G-C		1		Aldh1l2	P584A	1	KEALGACAI	3	1	638.4	5903.23	2.801	14	36.526	0	0	0	0.103	NoExpr	Pending	10	83504132	83504133	83504133	G	C	missense_variant
10-22371586-22371587-T-A		1		Raet1d	S187R	1	CRQKMDEFL	2	1	638.4	1227.69	2.1	3.4	1.442	0	0	0	0.318	NoExpr	Pending	10	22371586	22371587	22371587	T	A	missense_variant
7-5125843-5125844-C-T		1		Rasl2-9	R29H	1	KTTFMKHHL	7	1	638.4	937.756	2.699	4.1	0.401	0	0	0	0.04	NoExpr	Pending	7	5125843	5125844	5125844	C	T	missense_variant
6-73044834-73044835-C-A		1		Dnah6	E3501D	1	FVIDNLGKQFI	4	1	639.56	833.451	2.6	3	0.024	0	0	0	0.243	NoExpr	Pending	6	73044834	73044835	73044835	C	A	missense_variant
17-23387403-23387404-G-C		1		Vmn2r116	S430T	1	NYTCKKLYSYL	3	1	639.73	854.25	0.71	1.2	0	0	0	0	0.114	NoExpr	Pending	17	23387403	23387404	23387404	G	C	missense_variant
1-105678183-105678184-C-T		1		Relch	T183I	1	NRAGSISIL	8	1	642.82	298.717	2.1	0.8	20.15	0	0	0	0.221	NoExpr	Pending	1	105678183	105678184	105678184	C	T	missense_variant
12-115242907-115242908-T-A		2		Ighv1-56	K82M	1	YYNEMFKGK	5	2	643.07	14330.5	2.1	17	0	0	0	0	0.042	NoExpr	Pending	12	115242907	115242908	115242908	T	A	missense_variant
12-115861972-115861973-T-A		2		Ighv1-77	K82M	1	YYNEMFKGK	5	2	643.07	14330.5	2.1	17	0	0	0	0	0.04	NoExpr	Pending	12	115861972	115861973	115861973	T	A	missense_variant
7-103881604-103881605-T-C		1	1	Olfr66	I213V	1	LIIVFSYVL	4	2	645.4	520.991	2	2.1	0	0	0	0	0.046	NoExpr	Pending	7	103881604	103881605	103881605	T	C	missense_variant
X-152612667-152612668-T-G		1		Shroom2	N1442H	1	TYLSEEHL	7	1	645.41	763.133	0.5	0.7	0.443	0	0	0	0.688	NoExpr	Pending	X	152612667	152612668	152612668	T	G	missense_variant
6-131678761-131678762-G-A		1	1	Tas2r106	T42I	1	KFSIAGFIL	4	2	645.63	688.934	2.4	2.4	0	0	0	0	0.228	NoExpr	Pending	6	131678761	131678762	131678762	G	A	missense_variant
4-146893819-146893820-C-T		1		Gm21411	C29Y	1	EYLSFAQRTL	2	1	646.34	8297.38	0.7	15	0.009	0	0	0	0.029	NoExpr	Pending	4	146893819	146893820	146893820	C	T	missense_variant
7-12362283-12362284-C-T		1		Vmn1r84	D161N	1	TAINVIGPNTI	4	1	646.96	760.116	2.6	3.4	0	0	0	0	0.222	NoExpr	Pending	7	12362283	12362284	12362284	C	T	missense_variant
2-111983151-111983152-A-T		1		Olfr1309	H315Q	1	GYLRQYRKM	5	1	650.04	1527.88	0.85	4.2	0	0	0	0	0.179	NoExpr	Pending	2	111983151	111983152	111983152	A	T	missense_variant
7-102742199-102742200-C-T		1		Olfr78	D268N	1	RFGNSLNPI	7	1	650.05	1734.67	0.85	2	0	0	0	0	0.26	NoExpr	Pending	7	102742199	102742200	102742200	C	T	missense_variant
17-23477454-23477456-TG-CA		1		Vmn2r117	H326C	1	AFGQCHSEI	5	1	651	760.782	1.7	2.8	0	0	0	0	0.072	NoExpr	Pending	17	23477454	23477456	23477455	TG	CA	missense_variant
4-147678235-147678237-GT-TA		1		Zfp534	Y46L	1	LYMDVMLENL	10	1	652.08	3440.65	0.7	4.6	0.011	0	0	0	0.097	NoExpr	Pending	4	147678235	147678237	147678236	GT	TA	missense_variant
4-145534921-145534923-AC-TA		1		Zfp990	Y46L	1	LYMDVMLENL	10	1	652.08	3440.65	0.7	4.6	0.326	0	0	0	0.039	NoExpr	Pending	4	145534921	145534923	145534922	AC	TA	missense_variant
4-84276138-84276139-C-G		1		Bnc2	G916A	1	SDAASDEGI	4	1	652.65	1009.86	3.301	3.5	0.371	0	0	0	0.242	NoExpr	Pending	4	84276138	84276139	84276139	C	G	missense_variant
4-145622189-145622191-GA-TG		1	1	Gm13212	D66C	1	KYRKVLCQD	7	2	652.65	497.84	3.301	2.2	3.589	0	0	0	0.007	NoExpr	Pending	4	145622189	145622191	145622190	GA	TG	missense_variant
11-71122599-71122600-C-T		1		Nlrp1a	G608E	1	KFTAPEMVL	6	1	652.65	631.809	1.9	2	0.059	0	0	0	0.025	NoExpr	Pending	11	71122599	71122600	71122600	C	T	missense_variant

ID	H-2-Dd	H-2-Kd	H-2-Ld	Gene	AA Change	Num Passing Transcripts	Best Peptide	Pos	Num Passing Peptides	IC50 MT	IC50 WT	%ile MT	%ile WT	RNA Expr	RNA VAF	Allele Expr	RNA Depth	DNA VAF	Tier	Evaluation	CHROM	START	END	POS	REF_y	ALT_y	biotype
4-115532666-115532667-G-T		1		Cyp4a10	G500W	1	LVLKSKNWI	8	1	654.77	703.218	2.1	3.1	0	0	0	0	0.029	NoExpr	Pending	4	115532666	115532667	115532667	G	T	missense_variant
6-70518726-70518727-A-C		2		Igkv3-12	E79D	1	IYLASNLDS	8	2	656.28	641.342	2.1	1.7	0	0	0	0	0.233	NoExpr	Pending	6	70518726	70518727	70518727	A	C	missense_variant
12-112735255-112735256-C-T		1	1	Cep170b	P190S	1	SYSERPKDL	1	2	659.31	6824.8	1.9	11	12.418	0	0	0	0.446	NoExpr	Pending	12	112735255	112735256	112735256	C	T	missense_variant
7-40993461-40993462-C-G		1		4930433I11Rik	T185S	1	QYQDSTVSESI	8	1	659.75	659.754	1.2	1.2	0	0	0	0	0.057	NoExpr	Pending	7	40993461	40993462	40993462	C	G	missense_variant
11-69649177-69649178-G-A		1		Fxr2	S287N	1	NYLEFSEDSV	1	1	659.75	315.108	0.28	0.2	46.936	0	0	0	0.59	NoExpr	Pending	11	69649177	69649178	69649178	G	A	missense_variant
10-30647991-30647992-T-G		1		Ncoa7	S891R	1	SYFINGDIRSL	9	1	660.25	292.51	1.4	0.45	22.579	0	0	0	0.239	NoExpr	Pending	10	30647991	30647992	30647992	T	G	missense_variant
3-62419694-62419695-A-G		1	1	Arhgef26	K543R	1	RLLATNPSF	1	2	663.48	1131.96	2.5	3.6	0.421	0	0	0	0.118	NoExpr	Pending	3	62419694	62419695	62419695	A	G	missense_variant
11-59328678-59328679-A-C		1		Wnt9a	K177T	1	KYSSTFVKEFL	5	1	664.95	1672.99	0.71	0.79	0.544	0	0	0	0.273	NoExpr	Pending	11	59328678	59328679	59328679	A	C	missense_variant
4-129083798-129083799-G-A		2		Rnf19b	S602N	1	HYQLVSGNS	8	2	665.41	353.256	1.6	0.78	45.318	0	0	0	0.442	NoExpr	Pending	4	129083798	129083799	129083799	G	A	missense_variant
9-109274569-109274570-G-A		1		Fbxw14	H348Y	1	DYFGVSDKDV	2	1	665.98	8950.22	0.9	16	0	0	0	0	0.07	NoExpr	Pending	9	109274569	109274570	109274570	G	A	missense_variant
7-5480927-5480928-C-A		1		Vmn2r28	A758S	1	GYLACLSL	7	1	665.98	1048.24	0.44	0.68	0	0	0	0	0.031	NoExpr	Pending	7	5480927	5480928	5480928	C	A	missense_variant
7-10158938-10158939-C-A		1		Vmn2r52	A758S	1	GYLACLSL	7	1	665.98	1048.24	0.44	0.68	0	0	0	0	0.039	NoExpr	Pending	7	10158938	10158939	10158939	C	A	missense_variant
3-62488922-62488923-C-T		1		Dhx36	A438T	1	IYKERWPTYI	8	1	666.15	1723.69	0.86	2.6	34.856	0	0	0	0.365	NoExpr	Pending	3	62488922	62488923	62488923	C	T	missense_variant
10-26990809-26990810-C-T		1	1	Lama2	G2896D	1	NYTTRRIDPV	8	2	666.93	854.358	0.98	1.1	0.359	0	0	0	0.455	NoExpr	Pending	10	26990809	26990810	26990810	C	T	missense_variant
6-132980313-132980314-T-G		1		Tas2r109	K218Q	1	LHQRMQQHV	3	1	666.95	2225.89	1.4	5.1	0	0	0	0	0.063	NoExpr	Pending	6	132980313	132980314	132980314	T	G	missense_variant
8-71472239-71472240-G-A		1		Dda1	E44K	1	REYPSKQII	6	1	668.48	822.413	2.1	2.5	48.512	0	0	0	0.192	NoExpr	Pending	8	71472239	71472240	71472240	G	A	missense_variant
16-58636755-58636756-T-C		1		Ftdc2	N101S	1	SALLMAQSM	8	1	668.48	1259.19	2.1	3.5	0	0	0	0	0.077	NoExpr	Pending	16	58636755	58636756	58636756	T	C	missense_variant
11-58552173-58552174-G-A		1	1	Olfr328	H22Y	1	FSQSKYPAL	6	2	669.89	841.395	2.5	3	0	0	0	0	0.063	NoExpr	Pending	11	58552173	58552174	58552174	G	A	missense_variant
10-105413515-105413516-C-G		1	1	Tmtc2	A119P	1	YWTFMPGLM	6	2	670.29	503.605	1.8	1.6	0.122	0	0	0	0.087	NoExpr	Pending	10	105413515	105413516	105413516	C	G	missense_variant
19-5892550-5892551-C-T		1		Tigd3	A184T	1	LYRTVPGRV	4	1	673.38	487.63	1.3	1.3	0.73	0	0	0	0.235	NoExpr	Pending	19	5892550	5892551	5892551	C	T	missense_variant
12-114746396-114746398-CC-TA		1		Ighv1-22	G76Y	1	GYINPNNGYTS	9	1	674.19	562.186	2.199	2.2	0	0	0	0	0.032	NoExpr	Pending	12	114746396	114746398	114746397	CC	TA	missense_variant
10-76420486-76420487-A-G		1		Pcnt	F640L	1	KFAKEQDAL	9	1	674.19	5239.14	2.1	12	10.089	0	0	0	0.491	NoExpr	Pending	10	76420486	76420487	76420487	A	G	missense_variant
7-26611631-26611632-A-T		1	3	Vmn1r185	F149L	1	LIIFVYPFI	1	4	674.19	575.44	3.199	2.3	0	0	0	0	0.068	NoExpr	Pending	7	26611631	26611632	26611632	A	T	missense_variant
5-137380871-137380872-C-G		1	1	Zan	G5322A	1	EEQAATFIC	4	2	674.19	767.658	3.199	3.5	0.011	0	0	0	0.114	NoExpr	Pending	5	137380871	137380872	137380872	C	G	missense_variant
11-102946273-102946274-G-A		1		C1ql1	T63M	1	SGAPPPSML	8	1	674.67	320.081	2.6	1.2	0	0	0	0	0.385	NoExpr	Pending	11	102946273	102946274	102946274	G	A	missense_variant
12-40073324-40073325-C-T		1		Scin	A520T	1	RNLTSITRI	4	1	674.67	430.616	0.8	0.89	0.523	0	0	0	0.477	NoExpr	Pending	12	40073324	40073325	40073325	C	T	missense_variant
7-101836738-101836739-C-T		1		Inppl1	C5Y	1	VYGTPSPGGAL	2	1	677.19	10414	1	18	27.268	0	0	0	0.333	NoExpr	Pending	7	101836738	101836739	101836739	C	T	missense_variant
11-59099940-59099941-T-C		2		Obscn	E1602G	1	KGQQAHSEV	2	2	677.78	885.299	1.9	2.3	0.494	0	0	0	0.055	NoExpr	Pending	11	59099940	59099941	59099941	T	C	missense_variant
4-40190786-40190787-C-T		2		Aco1	A717V	1	SYGSRRGNDV	10	2	678.73	385.19	0.88	0.39	33.604	0	0	0	0.049	NoExpr	Pending	4	40190786	40190787	40190787	C	T	missense_variant
9-70125150-70125151-G-C		2		Fam81a	P20A	1	AYSSVSLVE	1	2	679.34	3972.74	2.199	7.9	0	0	0	0	0.109	NoExpr	Pending	9	70125150	70125151	70125151	G	C	missense_variant
1-85028735-85028736-T-C		2		AC167036.2	I37V	1	SLTVVIIAI	4	2	681.52	948.615	3.301	4.3	0	0	0	0	0.022	NoExpr	Pending	1	85028735	85028736	85028736	T	C	missense_variant
4-156338745-156338746-G-A		1	1	Vmn2r-ps159	V747I	1	SVIAFHIVL	7	2	682.07	3264.6	2.6	8	0	0	0	0	0.028	NoExpr	Pending	4	156338745	156338746	156338746	G	A	missense_variant
9-73075511-73075512-A-C		1		Rab27a	K33T	1	LYQYTDGTF	8	1	682.48	2239.19	1.3	3.7	1.034	0	0	0	0.309	NoExpr	Pending	9	73075511	73075512	73075512	A	C	missense_variant
17-14987606-14987607-A-G		1		9030025P20Rik	Y290C	1	CTMLLLSQL	1	1	682.75	449.014	2.199	1.3	0	0	0	0	0.013	NoExpr	Pending	17	14987606	14987607	14987607	A	G	missense_variant
7-7291660-7291661-G-C		1		Clcn4	T367S	1	IAVTAVSAI	7	1	682.75	1064.78	2.1	2.7	23.471	0	0	0	0.141	NoExpr	Pending	7	7291660	7291661	7291661	G	C	missense_variant
9-20588518-20588519-C-T		2		Zfp846	T15I	1	CYQVSVIFDDV	7	2	685.3	344.913	1.7	0.54	4.297	0	0	0	0.375	NoExpr	Pending	9	20588518	20588519	20588519	C	T	missense_variant
17-23640153-23640154-C-T		1		Mmp25	R173H	1	KKHTLTWSI	3	1	688.93	1376.93	1.4	3.2	0.45	0	0	0	0.165	NoExpr	Pending	17	23640153	23640154	23640154	C	T	missense_variant
7-35547979-35547980-G-A		1	1	Nudt19	L335F	1	SPYVYEIYMTF	11	2	688.93	819.146	0.75	0.78	34.054	0	0	0	0.659	NoExpr	Pending	7	35547979	35547980	35547980	G	A	missense_variant
7-3717411-3717412-A-T		1		Pirb	Y321N	1	EYNEPRLSVL	3	1	690.97	548.858	0.6	0.38	15.122	0	0	0	0.039	NoExpr	Pending	7	3717411	3717412	3717412	A	T	missense_variant
4-118869385-118869386-G-A		1		Olfr1331	V202I	1	HIIEMVDLI	9	1	691.97	4637.25	2	9.8	0	0	0	0	0.504	NoExpr	Pending	4	118869385	118869386	118869386	G	A	missense_variant
19-20640086-20640087-C-T		1		Aldh1a1	S461L	1	CYMMLLAQC	6	1	693.57	632.543	1.7	1.2	0.315	0	0	0	0.395	NoExpr	Pending	19	20640086	20640087	20640087	C	T	missense_variant
4-56946850-56946851-G-C		1		Tmem245	S187R	1	DYFSRLWIWTL	5	1	694.56	449.656	1.5	0.92	9.826	0	0	0	0.115	NoExpr	Pending	4	56946850	56946851	56946851	G	C	missense_variant
15-82454431-82454432-A-C		1		Cyp2d9	L222F	1	SLTEVSGFI	8	1	695.17	726.256	2	2.2	0	0	0	0	0.051	NoExpr	Pending	15	82454431	82454432	82454432	A	C	missense_variant
6-42912284-42912285-C-T		1		Olfr447	A254V	1	YGTVIVMYI	4	1	695.44	392.88	2.199	1.5	0	0	0	0	0.874	NoExpr	Pending	6	42912284	42912285	42912285	C	T	missense_variant
16-56257948-56257949-A-C		1		Impg2	T425P	1	TTTPTTTTI	4	1	697.04	638.396	1.7	1.7	0.037	0	0	0	0.297	NoExpr	Pending	16	56257948	56257949	56257949	A	C	missense_variant
2-130248713-130248714-G-A		1		Tmc2	S660N	1	CWAVMSNNV	7	1	698.65	265.62	1.3	0.6	0	0	0	0	0.403	NoExpr	Pending	2	130248713	130248714	130248714	G	A	missense_variant
5-111225997-111225998-C-G		1		Ttc28	A1069G	1	GKTVSYSSL	1	1	698.65	369.905	2.199	1.7	4.164	0	0	0	0.214	NoExpr	Pending	5	111225997	111225998	111225998	C	G	missense_variant
3-83033096-83033097-G-A		1		Fga	G686E	1	SYRGTAEDAL	7	1	701.66	389.85	0.91	0.56	0	0	0	0	0.22	NoExpr	Pending	3	83033096	83033097	83033097	G	A	missense_variant
17-18597855-18597856-G-A		1		Vmn2r96	G565E	1	CFMALESYA	6	1	701.88	575.559	1.6	1.5	0	0	0	0	0.021	NoExpr	Pending	17	18597855	18597856	18597856	G	A	missense_variant
2-98662266-98662267-T-A		3		Gm10801	F11I	1	SRILVISSF	3	3	703.22	1291.99	2.199	3.5	0.204	0	0	0	0.055	NoExpr	Pending	2	98662266	98662267	98662267	T	A	missense_variant
4-147178798-147178799-A-G		1		Zfp991	H211R	1	RYRSSKSDK	1	1	703.22	496.695	1.8	1.2	7.229	0	0	0	0.5	NoExpr	Pending	4	147178798	147178799	147178799	A	G	missense_variant
5-15030266-15030267-G-C		1		Gm17019	A177G	1	KEVQGDWAI	5	1	704.01	497.976	3	2.5	0	0	0	0	0.021	NoExpr	Pending	5	15030266	15030267	15030267	G	C	missense_variant
12-113685590-113685591-C-G		2		Ighv2-5	A81P	1	APFMSRLSI	2	2	708.09	933.448	2.199	2.7	0	0	0	0	0.031	NoExpr	Pending	12	113685590	113685591	113685591	C	G	missense_variant
1-186632484-186632485-T-C		1		Tgfb2	S271G	1	NYIIPNKGEEL	8	1	711.02	711.017	0.7	0.7	0.113	0	0	0	0.027	NoExpr	Pending	1	186632484	186632485	186632485	T	C	missense_variant
1-132046445-132046446-A-G		1		Mfsd4a	V382A	1	VFINVVGAV	8	1	711.64	1303.95	2.1	3.1	34.344	0	0	0	0.138	NoExpr	Pending	1	132046445	132046446	132046446	A	G	missense_variant
X-127063259-127063260-G-A		2		Gm382	V1023I	1	KRIKSLQME	3	2	711.66	2781.92	3.5	13	0	0	0	0	0.821	NoExpr	Pending	X	127063259	127063260	127063260	G	A	missense_variant
18-35213088-35213089-A-G		1		Lrrtm2	S387P	1	EYTKISSPS	8	1	711.85	605.466	2.199	1.4	2.388	0	0	0	0.187	NoExpr	Pending	18	35213088	35213089	35213089	A	G	missense_variant
17-46134990-46134991-C-T		1		Rsph9	E218K	1	KYDIPRGSWSI	1	1	711.98	1737.86	0.48	2	0.054	0	0	0	0.333	NoExpr	Pending	17	46134990	46134991	46134991	C	T	missense_variant
4-9537694-9537695-C-T		1		Asph	R361K	1	KYPQSPRAK	9	1	713	1312.47	3.4	5	75.51	0	0	0	0.171	NoExpr	Pending	4	9537694	9537695	9537695	C	T	missense_variant
12-113769970-113769971-T-C		1		Ighv2-9-1	N77D	1	TDYNSALKSRL	2	1	714.3	1351.7	3	3.1	0	0	0	0	0.026	NoExpr	Pending	12	113769970	113769971	113769971	T	C	missense_variant
6-97296288-97296289-G-A		1		Frmd4b	T714I	1	FFILSKSQR	3	1	717.94	1866.77	2.199	5.1	8.643	0	0	0	0.145	NoExpr	Pending	6	97296288	97296289	97296289	G	A	missense_variant
17-45568408-45568409-T-A		2		Hsp90ab1	N629Y	1	KHLEIYPDHPI	6	2	719.41	1003.51	1.3	1.3	2213	0	0	0	0.048	NoExpr	Pending	17	45568408	45568409	45568409	T	A	missense_variant

ID	H-2-Dd	H-2-Kd	H-2-Ld	Gene	AA Change	Num Passing Transcripts	Best Peptide	Pos	Num Passing Peptides	IC50 MT	IC50 WT	%ile MT	%ile WT	RNA Expr	RNA VAF	Allele Expr	RNA Depth	DNA VAF	Tier	Evaluation	CHROM	START	END	POS	REF_y	ALT_y	biotype
10-107494294-107494295-G-C		1		Myf6	A137G	1	KVEILRSGI	8	1	719.41	263.081	3.5	1.3	0.1	0	0	0	0.244	NoExpr	Pending	10	107494294	107494295	107494295	G	C	missense_variant
7-29103579-29103580-A-G		1		Ryr1	L676P	1	FPTAQATHL	2	1	719.41	611.069	2.199	1.8	0.538	0	0	0	0.31	NoExpr	Pending	7	29103579	29103580	29103580	A	G	missense_variant
6-58435015-58435016-G-A		1		Vmn1r30	P277L	1	AYPTITLLVQI	7	1	719.41	740.96	1.3	1.4	0	0	0	0	0.038	NoExpr	Pending	6	58435015	58435016	58435016	G	A	missense_variant
2-86122769-86122770-C-T		1	1	Olfr1038-ps	P282L	1	SVFYTVILM	8	2	719.45	1629.3	2.6	3.2	0	0	0	0	0.424	NoExpr	Pending	2	86122769	86122770	86122770	C	T	missense_variant
10-102544813-102544814-C-T		1		Rassf9	T19I	1	TRHKNRSPI	9	1	719.6	11805.7	1.2	19	0.131	0	0	0	0.624	NoExpr	Pending	10	102544813	102544814	102544814	C	T	missense_variant
2-164289936-164289937-G-A		1		Svs3a	V143M	1	GQMKSQTML	3	1	721.26	2361.89	2.199	6.2	0	0	0	0	0.07	NoExpr	Pending	2	164289936	164289937	164289937	G	A	missense_variant
5-36065346-36065347-A-C		1	1	Sorcs2	F355V	1	TVQDEYIVL	8	2	722.49	337.323	3.1	1.8	4.147	0	0	0	0.07	NoExpr	Pending	5	36065346	36065347	36065347	A	C	missense_variant
6-43116448-43116449-G-A		1		Olfr441	A236T	1	TFSTCSSHL	1	1	727.23	653.522	3.5	2.1	0	0	0	0	0.818	NoExpr	Pending	6	43116448	43116449	43116449	G	A	missense_variant
7-8471616-8471617-C-G		1		Vmn2r45	G804A	1	VYHSTKAKHM	7	1	727.58	419.643	0.7	0.6	0	0	0	0	0.111	NoExpr	Pending	7	8471616	8471617	8471617	C	G	missense_variant
17-20042712-20042713-T-G		1	1	Vmn2r104	Q162P	1	APIGTLLQL	2	2	727.78	18551.1	2.699	17	0	0	0	0	0.225	NoExpr	Pending	17	20042712	20042713	20042713	T	G	missense_variant
11-104289964-104289965-C-T		1		Mapt	P82L	1	AAAQLHTEI	5	1	727.93	1686.9	2	3.4	0.09	0	0	0	0.509	NoExpr	Pending	11	104289964	104289965	104289965	C	T	missense_variant
12-72157485-72157486-T-G		1		Ccdc175	K260N	1	TYYKKNELTRL	6	1	728	611.631	1.2	0.81	0	0	0	0	0.163	NoExpr	Pending	12	72157485	72157486	72157486	T	G	missense_variant
2-86217361-86217362-A-C		1		Olfr1046	F116C	1	IFIITELCI	8	1	728.22	656.281	2.1	1.6	0	0	0	0	0.041	NoExpr	Pending	2	86217361	86217362	86217362	A	C	missense_variant
16-56260477-56260478-C-G		2		Impg2	P882A	1	SVHYTEMAI	8	2	729.61	1452.41	3.1	3.3	0.037	0	0	0	0.056	NoExpr	Pending	16	56260477	56260478	56260478	C	G	missense_variant
15-50660861-50660862-C-G		1	1	Trps1	C1224S	1	CVHSGIVFL	4	2	730.85	732.825	2.9	2.7	13.874	0	0	0	0.148	NoExpr	Pending	15	50660861	50660862	50660862	C	G	missense_variant
9-21230766-21230767-C-T		1		Keap1	V604M	1	SGMGVAVTM	3	1	731.58	2276.46	2.199	6.1	26.899	0	0	0	0.27	NoExpr	Pending	9	21230766	21230767	21230767	C	T	missense_variant
16-32751954-32751956-AC-GA		1		Muc4	N611R	1	TETSSQRTI	7	1	732.98	467.832	2.199	1.4	0.047	0	0	0	0.013	NoExpr	Pending	16	32751954	32751956	32751955	AC	GA	missense_variant
11-60779684-60779685-T-G		2		Smcr8	I553S	1	AYADNEGAS	9	2	732.98	44.884	2.199	0.2	14.566	0	0	0	0.17	NoExpr	Pending	11	60779684	60779685	60779685	T	G	missense_variant
15-60919764-60919765-G-A		1		A1bg	A274V	1	GFSPTRDVI	8	1	733.42	490.072	1.5	0.8	0	0	0	0	0.881	NoExpr	Pending	15	60919764	60919765	60919765	G	A	missense_variant
11-45983392-45983393-G-A		1		Sox30	A377T	1	NAFMVWTRI	7	1	734.67	2938.26	2.301	7.5	0	0	0	0	0.181	NoExpr	Pending	11	45983392	45983393	45983393	G	A	missense_variant
4-139779672-139779673-C-T		1		Pax7	G351E	1	AYEARHSFS	3	1	736.36	853.277	2.199	2.5	0	0	0	0	0.171	NoExpr	Pending	4	139779672	139779673	139779673	C	T	missense_variant
5-94383664-94383665-T-G		1		AA792892	L136W	1	LYAWDLMPL	4	1	740.05	1143.56	1.7	1.5	0	0	0	0	0.03	NoExpr	Pending	5	94383664	94383665	94383665	T	G	missense_variant
8-14942625-14942626-C-T		1		Arhgef10	T288I	1	KKQLSHDLI	9	1	741.46	12366.9	2.199	24	11.633	0	0	0	0.625	NoExpr	Pending	8	14942625	14942626	14942626	C	T	missense_variant
6-86732975-86732976-G-C		2		Gmcl1	D63E	1	CYCHPESETDD	6	2	743.14	735.143	1.9	1.3	22.822	0	0	0	0.135	NoExpr	Pending	6	86732975	86732976	86732976	G	C	missense_variant
17-45568249-45568250-G-A		2		Hsp90ab1	R682C	1	CMIKLGLGI	1	2	743.14	1199.24	1.3	3.2	2213	0	0	0	0.079	NoExpr	Pending	17	45568249	45568250	45568250	G	A	missense_variant
17-34715849-34715850-G-C		2		Tnxb	E2478Q	1	QYQDTENQTQ	8	2	743.14	735.143	1.3	1.3	3.862	0	0	0	0.293	NoExpr	Pending	17	34715849	34715850	34715850	G	C	missense_variant
18-45685368-45685369-C-T		2		Kcnn2	T511I	1	ILIGSIHAL	1	2	743.46	957.767	1.5	2.2	0	0	0	0	0.298	NoExpr	Pending	18	45685368	45685369	45685369	C	T	missense_variant
5-109047050-109047051-G-C		1		Vmn2r11	T803S	1	SFLPVYHST	1	1	743.46	2787.79	2.301	4.8	0	0	0	0	0.052	NoExpr	Pending	5	109047050	109047051	109047051	G	C	missense_variant
X-165252383-165252384-A-T		3	1	Glra2	Y256N	1	NYLIQMYI	1	4	743.53	323.11	0.96	0.46	0	0	0	0	0.354	NoExpr	Pending	X	165252383	165252384	165252384	A	T	missense_variant
13-89705412-89705413-C-G		1		Vcan	G476A	1	ISQHATASQAV	7	1	743.75	1174.84	2.5	1.7	2.855	0	0	0	0.164	NoExpr	Pending	13	89705412	89705413	89705413	C	G	missense_variant
16-32751941-32751942-A-T		1		Muc4	T607S	1	TESSSQNTI	3	1	750.34	467.832	2.301	1.4	0.047	0	0	0	0.012	NoExpr	Pending	16	32751941	32751942	32751942	A	T	missense_variant
1-33777641-33777642-G-A		1		Zfp451	P409L	1	CHISEGSRL	9	1	750.34	5309.95	1.9	13	19.163	0	0	0	0.654	NoExpr	Pending	1	33777641	33777642	33777642	G	A	missense_variant
7-141639591-141639592-C-T		1		Muc6	E1723K	1	SVTPTSKVI	7	1	751.22	540.866	3.6	2.3	0.02	0	0	0	0.018	NoExpr	Pending	7	141639591	141639592	141639592	C	T	missense_variant
6-123315808-123315809-T-C		1		Vmn2r19	L270S	1	RFMERFSSTRV	8	1	751.22	828.057	1.3	1.5	0	0	0	0	0.406	NoExpr	Pending	6	123315808	123315809	123315809	T	C	missense_variant
11-116677607-116677608-G-A		1		St6galnac2	P343L	1	HYFEREKKLLI	9	1	753.15	633.695	0.5	0.6	0.248	0	0	0	0.269	NoExpr	Pending	11	116677607	116677608	116677608	G	A	missense_variant
7-23984778-23984779-T-C		1		Vmn1r181	L223P	1	QYIFTPNQD	6	1	753.51	771.063	2.301	2.3	0	0	0	0	0.032	NoExpr	Pending	7	23984778	23984779	23984779	T	C	missense_variant
2-122290606-122290607-G-A		1	1	Duox2	L775F	1	QFFAQVLDI	2	2	755.25	2642.96	2.301	6.9	0.034	0	0	0	0.211	NoExpr	Pending	2	122290606	122290607	122290607	G	A	missense_variant
19-11472453-11472454-C-T		1	1	Ms4a6c	P70L	1	LGIILASVL	9	2	756.99	6840.53	2.301	16	78.803	0	0	0	0.031	NoExpr	Pending	19	11472453	11472454	11472454	C	T	missense_variant
10-129625329-129625330-T-C		2		Olfr798	I244V	1	SHMVVVSIS	4	2	762.24	1219.24	2.6	3.4	0	0	0	0	0.107	NoExpr	Pending	10	129625329	129625330	129625330	T	C	missense_variant
7-23835106-23835107-A-T		1		Vmn1r176	V207E	1	IWSSVSMEI	8	1	762.43	839.963	1.4	1	0	0	0	0	0.193	NoExpr	Pending	7	23835106	23835107	23835107	A	T	missense_variant
1-85485033-85485034-T-A		3		AC147806.1	I37F	1	SITTSLTFV	8	3	764.29	1229.28	2.301	3.8	0	0	0	0	0.055	NoExpr	Pending	1	85485033	85485034	85485034	T	A,C	missense_variant
15-35709611-35709612-T-G		1		Vps13b	S1902A	1	TRSAARQAL	4	1	764.29	1143.56	2.301	3.2	18.069	0	0	0	0.129	NoExpr	Pending	15	35709611	35709612	35709612	T	G	missense_variant
8-88156500-88156501-A-G		1		Heatr3	T360A	1	LLAAQQTAL	3	1	766.06	921.001	2.4	3.2	53.067	0	0	0	0.23	NoExpr	Pending	8	88156500	88156501	88156501	A	G	missense_variant
12-101509249-101509250-A-G		1		Catsperb	E351G	1	STIKNGKSI	6	1	767.66	792.984	1.5	1.5	0	0	0	0	0.111	NoExpr	Pending	12	101509249	101509250	101509250	A	G	missense_variant
14-101913324-101913325-C-A		1		Lmo7	F1150L	1	SSLSVTTDL	9	1	767.66	6267.44	1.8	9.9	74.175	0	0	0	0.156	NoExpr	Pending	14	101913324	101913325	101913325	C	A	missense_variant
16-32752014-32752015-C-A		1		Muc4	P631Q	1	SSAQSTTHM	4	1	767.66	585.724	1.7	1.7	0.047	0	0	0	0.017	NoExpr	Pending	16	32752014	32752015	32752015	C	A	missense_variant
16-20344739-20344740-C-T		2		Abcc5	C1249Y	1	GYIKIDGIR	2	2	769.59	15497.4	2.301	28	16.578	0	0	0	0.06	NoExpr	Pending	16	20344739	20344740	20344740	C	T	missense_variant
12-114253804-114253805-G-T		1		Ighv3-4	N55K	1	KWIRQVSGS	1	1	769.59	1876.12	3.1	5.3	0	0	0	0	0.026	NoExpr	Pending	12	114253804	114253805	114253805	G	T	missense_variant
16-37868970-37868971-A-G		1		Lrrc58	N159S	1	AEIESLRSL	5	1	769.59	2422.48	2.4	6.3	21.868	0	0	0	0.3	NoExpr	Pending	16	37868970	37868971	37868971	A	G	missense_variant
18-37005864-37005865-A-C		1		Pcdha11	E182D	1	EYFSLDVPTTD	6	1	770.69	1354.82	2.801	2.9	0	0	0	0	0.191	NoExpr	Pending	18	37005864	37005865	37005865	A	C	missense_variant
17-12719893-12719894-A-C		1		Igf2r	F508V	1	VFINVCHRV	1	1	773.8	390.176	1.2	0.8	15.283	0	0	0	0.243	NoExpr	Pending	17	12719893	12719894	12719894	A	C	missense_variant
15-9149187-9149188-C-G		1		Lmbrd2	P53A	1	LALDVSTTI	2	1	774.62	587.611	1.8	1.6	17.018	0	0	0	0.123	NoExpr	Pending	15	9149187	9149188	9149188	C	G	missense_variant
6-122713538-122713539-C-T		1		Nanog	P301L	1	NYSVTLPGEI	6	1	775.3	1682.67	1	2	0	0	0	0	0.057	NoExpr	Pending	6	122713538	122713539	122713539	C	T	missense_variant
17-34958106-34958107-G-A		2		Hspa1b	R301W	1	FYTSITRAW	9	2	776.48	1325.14	1	2.3	10.32	0	0	0	0.048	NoExpr	Pending	17	34958106	34958107	34958107	G	A	missense_variant
X-94638018-94638019-C-G		1		Gspt2	A588G	1	GRLRTAGTI	1	1	782.1	361.626	1.3	0.95	10.644	0	0	0	0.321	NoExpr	Pending	X	94638018	94638019	94638019	C	G	missense_variant
3-151517481-151517482-A-G		2		Adgrl4	Y565C	1	IHLCLIVVGVI	4	2	782.91	1046.43	2.9	2.2	5.815	0	0	0	0.345	NoExpr	Pending	3	151517481	151517482	151517482	A	G	missense_variant
15-82391001-82391002-T-G		1		Cyp2d11	K248T	1	QGQTSFMAI	4	1	783.59	543.363	2	1.8	0	0	0	0	0.163	NoExpr	Pending	15	82391001	82391002	82391002	T	G	missense_variant
12-114708823-114708824-T-A		1		Ighv1-19	S59C	1	CHGKSLEWI	1	1	783.9	534.884	2.199	1.4	0	0	0	0	0.043	NoExpr	Pending	12	114708823	114708824	114708824	T	A	missense_variant
12-114746448-114746449-T-A		1		Ighv1-22	S59C	1	CHGKSLEWI	1	1	783.9	534.884	2.199	1.4	0	0	0	0	0.018	NoExpr	Pending	12	114746448	114746449	114746449	T	A	missense_variant
17-7804856-7804857-C-T		1	1	Fndc1	E40K	1	KVNAKTHSF	5	2	784.45	759.397	2.1	2.2	0.299	0	0	0	0.341	NoExpr	Pending	17	7804856	7804857	7804857	C	T	missense_variant
4-145622192-145622193-C-G		1		Gm13212	Q67E	1	KYRKVLDED	8	1	785.4	497.84	2.4	2.2	3.589	0	0	0	0.014	NoExpr	Pending	4	145622192	145622193	145622193	C	G	missense_variant
6-112749406-112749407-T-G		1		Srgap3	I519L	1	LPLVVESCI	1	1	785.4	562.458	2.4	1.8	9.064	0	0	0	0.088	NoExpr	Pending	6	112749406	112749407	112749407	T	G	missense_variant

ID	H-2-Dd	H-2-Kd	H-2-Ld	Gene	AA Change	Num Passing Transcripts	Best Peptide	Pos	Num Passing Peptides	IC50 MT	IC50 WT	%ile MT	%ile WT	RNA Expr	RNA VAF	Allele Expr	RNA Depth	DNA VAF	Tier	Evaluation	CHROM	START	END	POS	REF_y	ALT_y	biotype
13-62171235-62171237-AA-CG		1		Zfp808	Q93P	1	SHAPRHERI	4	1	787.21	819.47	1.4	1.2	5.763	0	0	0	0.025	NoExpr	Pending	13	62171235	62171237	62171236	AA	CG	missense_variant
13-27098647-27098648-C-G		1		Prl3d1	A124G	1	KHLVSGLTAL	6	1	788.37	628.985	0.9	0.46	0	0	0	0	0.024	NoExpr	Pending	13	27098647	27098648	27098648	C	G	missense_variant
13-12294368-12294369-G-T		1		Actn2	L273I	1	KVIAVNQEN	3	1	789.02	2084.92	3.6	9.1	0.458	0	0	0	0.19	NoExpr	Pending	13	12294368	12294369	12294369	G	T	missense_variant
4-88683345-88683346-T-A		2		Ifna2	K145I	1	IYFHRITVYL	1	2	790.47	266.59	1.1	0.41	0	0	0	0	0.058	NoExpr	Pending	4	88683345	88683346	88683346	T	A	missense_variant
2-58137903-58137904-C-T		1		Cytip	V190M	1	KWMELRSLH	3	1	790.84	2673.56	2.4	6.9	10.819	0	0	0	0.509	NoExpr	Pending	2	58137903	58137904	58137904	C	T	missense_variant
14-53635152-53635153-G-T		1		Trav13-2	A29S	1	QVQQSPSSL	7	1	790.84	4614.13	2.801	12	0	0	0	0	0.014	NoExpr	Pending	14	53635152	53635153	53635153	G	T	missense_variant
17-20113527-20113528-C-A		1		Fpr-rs7	L233F	1	GFVNSSRPL	2	1	791.35	6564.03	2.301	7.8	0	0	0	0	0.02	NoExpr	Pending	17	20113527	20113528	20113528	C	A	missense_variant
17-33997115-33997116-C-A		1		H2-K1	V310F	1	SNMATVAFL	8	1	792.67	792.984	2.4	1.9	899.72	0	0	0	0.012	NoExpr	Pending	17	33997115	33997116	33997116	C	A	missense_variant
4-88571581-88571582-T-C		1		Ifna14	K73E	1	QQIKEAQAI	5	1	792.67	1321.57	2.4	3.5	0	0	0	0	0.014	NoExpr	Pending	4	88571581	88571582	88571582	T	C	missense_variant
11-58529814-58529815-G-A		1		Olfr330	T57I	1	LILLVLSDI	9	1	792.67	13004.4	2.4	24	0	0	0	0	0.01	NoExpr	Pending	11	58529814	58529815	58529815	G	A	missense_variant
7-40993293-40993294-A-C		1		4930433I11Rik	D129A	1	SDNASLGGI	4	1	792.98	1145.75	3.9	4.9	0	0	0	0	0.087	NoExpr	Pending	7	40993293	40993294	40993294	A	C	missense_variant
11-109517419-109517420-G-A		1		Arsg	A133T	1	LRQEGYVTTMI	9	1	792.98	2192.64	2.801	4.3	1.697	0	0	0	0.344	NoExpr	Pending	11	109517419	109517420	109517420	G	A	missense_variant
3-53516991-53516992-G-A		1		Frem2	S3008F	1	WYIHTIYTVKF	11	1	792.98	1607.56	1.4	1.9	0.006	0	0	0	0.235	NoExpr	Pending	3	53516991	53516992	53516992	G	A	missense_variant
2-98667255-98667256-G-T		1		Gm10800	R16S	1	CHISSPTVDI	5	1	793.07	2198.63	0.6	2.4	1.05	0	0	0	0.018	NoExpr	Pending	2	98667255	98667256	98667256	G	T	missense_variant
7-49464780-49464781-T-A		1		Nav2	S838T	1	RQLATRGSSI	5	1	793.34	777.07	0.6	0.6	18.378	0	0	0	0.556	NoExpr	Pending	7	49464780	49464781	49464781	T	A	missense_variant
17-45568404-45568405-G-A		2		Hsp90ab1	P630L	1	LEINLDHPI	5	2	796.32	1845.4	1.9	5	2213	0	0	0	0.051	NoExpr	Pending	17	45568404	45568405	45568405	G	A	missense_variant
11-119293950-119293951-A-C		1	1	Eif4a3	H223Q	1	LPQEILEM	3	2	800	592.51	0.5	0.41	103.23	0	0	0	0.032	NoExpr	Pending	11	119293950	119293951	119293951	A	C	missense_variant
17-34958181-34958182-A-C		1		Hspa1b	S276A	1	KRTLSASTQ	6	1	800	822.413	3.301	3.3	10.32	0	0	0	0.236	NoExpr	Pending	17	34958181	34958182	34958182	A	C	missense_variant
6-57524931-57524932-T-G		1		Ppm1k	K82T	1	TYGKPIPKI	1	1	800	253.081	2.4	0.7	3.281	0	0	0	0.144	NoExpr	Pending	6	57524931	57524932	57524932	T	G	missense_variant
12-103587518-103587519-T-C		1		Ppp4r4	V412A	1	PEAPVRHTI	3	1	800	1585.22	1.6	3.6	0.011	0	0	0	0.235	NoExpr	Pending	12	103587518	103587519	103587519	T	C	missense_variant
5-138248815-138248816-G-A		1		Nxpe5	R328H	1	HSFPTVDSI	1	1	802.16	1089.58	2.301	3	26.163	0	0	0	0.039	NoExpr	Pending	5	138248815	138248816	138248816	G	A	missense_variant
14-53538269-53538270-G-A		1		Trav12-1	R4H	1	MNMHPVTSSV	4	1	802.53	727.232	1.3	1.2	0	0	0	0	0.044	NoExpr	Pending	14	53538269	53538270	53538270	G	A	missense_variant
6-41188956-41188957-A-T		1		Trbv20	L105F	1	AYLEDRGFYL	8	1	802.86	999.171	0.8	1	0.084	0	0	0	0.792	NoExpr	Pending	6	41188956	41188957	41188957	A	T	missense_variant
12-114222989-114222990-C-T		1		Ighv7-4	S54N	1	YYMNWVRQP	4	1	803.69	696.429	2.5	1.8	0	0	0	0	0.082	NoExpr	Pending	12	114222989	114222990	114222990	C	T	missense_variant
2-128676211-128676212-C-T		1	1	Anapc1	D241N	1	NPAVKIVFL	1	2	806.85	2054.82	2.4	4.6	53.236	0	0	0	0.474	NoExpr	Pending	2	128676211	128676212	128676212	C	T	missense_variant
5-3344457-3344458-G-C		1		Cdk6	R31P	1	AYGKVFKAP	9	1	807.72	908.364	3.301	3.6	39.729	0	0	0	0.06	NoExpr	Pending	5	3344457	3344458	3344458	G	C	missense_variant
3-39009783-39009784-G-A		1		Fat4	A4630T	1	SITPSDADI	3	1	810.33	598.537	4	3	0.174	0	0	0	0.184	NoExpr	Pending	3	39009783	39009784	39009784	G	A	missense_variant
2-109894548-109894549-T-C		1		Lin7c	V41A	1	KLQALQRAL	8	1	810.33	1039.3	1.6	2.2	76.142	0	0	0	0.175	NoExpr	Pending	2	109894548	109894549	109894549	T	C	missense_variant
11-71123708-71123710-GG-CA		1		Nlrp1a	T238M	1	SQMQPVHTL	3	1	810.33	1858.19	2.1	4.7	0.059	0	0	0	0.068	NoExpr	Pending	11	71123708	71123710	71123709	GG	CA	missense_variant
11-71217965-71217967-TC-CA		1		Nlrp1b	R236M	1	SQMQPVHTL	3	1	810.33	4429.97	2.1	8.4	2.359	0	0	0	0.189	NoExpr	Pending	11	71217965	71217967	71217966	TC	CA	missense_variant
11-3221174-3221175-C-T		1		Eif4enif1	S222L	1	SYTEEEPEWFL	11	1	812.29	2416.9	3.301	8.5	34.626	0	0	0	0.235	NoExpr	Pending	11	3221174	3221175	3221175	C	T	missense_variant
6-84575234-84575235-G-A		1		Cyp26b1	S307F	1	AYATTASASTF	11	1	812.62	783.213	0.34	2.3	6.167	0	0	0	0.865	NoExpr	Pending	6	84575234	84575235	84575235	G	A	missense_variant
7-3897221-3897222-G-A		1		Gm14548	S127L	1	SLLAQASPV	3	1	813	1079.17	2.301	2.9	4.646	0	0	0	0.037	NoExpr	Pending	7	3897221	3897222	3897222	G	A	missense_variant
8-43521737-43521738-T-G		1		Adam26b	I76L	1	KNLMSRNLL	9	1	816.75	319.956	2.5	0.9	0	0	0	0	0.089	NoExpr	Pending	8	43521737	43521738	43521738	T	G	missense_variant
2-127008876-127008877-G-A		1		Ap4e1	V42I	1	GLIRGITAL	6	1	820.84	863.495	1.7	1.8	10.302	0	0	0	0.12	NoExpr	Pending	2	127008876	127008877	127008877	G	A	missense_variant
19-6351192-6351193-T-C		1		Map4k2	S590P	1	RRFALPTKI	6	1	820.84	629.883	1.9	1.5	9.944	0	0	0	0.03	NoExpr	Pending	19	6351192	6351193	6351193	T	C	missense_variant
7-31375891-31375893-GA-CT		1		Scgb1b3	E56L	1	EYVKQYKDDPL	11	1	821.69	3454.02	2.5	9.2	0	0	0	0	0.028	NoExpr	Pending	7	31375891	31375893	31375892	GA	CT	missense_variant
5-98737674-98737675-G-A		1	1	Cfap299	G147E	1	EDFEVYFNE	9	2	821.92	1761.29	3	5.3	0.104	0	0	0	0.297	NoExpr	Pending	5	98737674	98737675	98737675	G	A	missense_variant
3-144759193-144759194-G-C		1		Clca3a1	P93A	1	EYLMAKRESYD	5	1	823.91	1249.17	3.699	4	34.84	0	0	0	0.023	NoExpr	Pending	3	144759193	144759194	144759194	G	C	missense_variant
14-121912246-121912247-G-C		1		Gpr18	A122G	1	RYMGIVQPK	4	1	826.51	302.91	1.2	0.75	0.843	0	0	0	0.057	NoExpr	Pending	14	121912246	121912247	121912247	G	C	missense_variant
5-15580673-15580674-A-C		1		Gm21083	N159T	1	SRLLMEETL	8	1	826.53	1476.59	1.9	3.8	0.062	0	0	0	0.221	NoExpr	Pending	5	15580673	15580674	15580674	A	C	missense_variant
15-58182880-58182881-C-A		1		Fbxo32	G311V	1	CYPRREQYV	9	1	827.47	4635.43	1.5	8.4	0.562	0	0	0	0.37	NoExpr	Pending	15	58182880	58182881	58182881	C	A	missense_variant
3-125683292-125683293-T-G		2		Ndst4	F564V	1	KYFELVPEQKD	6	2	828.06	819.146	2.199	1.5	1.996	0	0	0	0.045	NoExpr	Pending	3	125683292	125683293	125683293	T	G	missense_variant
1-85089561-85089562-C-T		1		A530032D15Rik	G188R	1	VWAPGRTFL	6	1	828.11	1004.82	2.1	2.9	24.026	0	0	0	0.06	NoExpr	Pending	1	85089561	85089562	85089562	C	T	missense_variant
12-114140962-114140963-C-A		1		Ighv9-3	G27V	1	AQIQLVQSV	9	1	828.11	3733.28	2.5	9.2	0	0	0	0	0.044	NoExpr	Pending	12	114140962	114140963	114140963	C	A	missense_variant
7-105368574-105368575-C-T		1		Olfr692	T83I	1	IDLGLATSI	1	1	828.44	805.65	1.5	2.2	0.019	0	0	0	0.458	NoExpr	Pending	7	105368574	105368575	105368575	C	T	missense_variant
13-4145267-4145268-C-G		1		Akr1c18	A64P	1	IGQPILSKI	4	1	830.35	539.622	2.301	1.8	0.545	0	0	0	0.057	NoExpr	Pending	13	4145267	4145268	4145268	C	G	missense_variant
2-131313165-131313166-A-G		2		Rnf24	I47T	1	CYLTRLRHQ	4	2	833.85	1574.31	2.5	4.3	11.271	0	0	0	0.096	NoExpr	Pending	2	131313165	131313166	131313166	A	G	missense_variant
15-101460176-101460178-CC-TG		2		Krt81	G398A	1	EYQEVMNSKLA	11	2	835.11	845.8	3.301	1.4	0	0	0	0	0.037	NoExpr	Pending	15	101460176	101460178	101460177	CC	TG	missense_variant
2-119070100-119070101-C-G		1		Knl1	S761C	1	CLAGTSKTI	1	1	836.1	570.506	2.1	1.4	13.323	0	0	0	0.292	NoExpr	Pending	2	119070100	119070101	119070101	C	G	missense_variant
12-115208227-115208229-CC-TT		2		Ighv1-55	G73N	1	IYPNSGSTNYN	4	2	837.07	2522.78	2.9	8.6	0	0	0	0	0.02	NoExpr	Pending	12	115208227	115208229	115208228	CC	TT	missense_variant
16-48565644-48565645-G-T		1		Morc1	D544Y	1	KYERERQLQ	2	1	838.03	35288.6	2.9	50	0	0	0	0	0.189	NoExpr	Pending	16	48565644	48565645	48565645	G	T	missense_variant
9-40782560-40782561-C-T		1		Clmp	T358I	1	VHHTTLTKAEI	11	1	842.24	14403.3	1	15	2.685	0	0	0	0.315	NoExpr	Pending	9	40782560	40782561	40782561	C	T	missense_variant
8-40999693-40999694-C-T		1		Mtus1	M1019I	1	SEIKKSHEI	9	1	845.45	3206.93	2.5	8.1	5.24	0	0	0	0.573	NoExpr	Pending	8	40999693	40999694	40999694	C	T	missense_variant
1-85484519-85484520-C-T		1		AC147806.1	R208Q	1	IYREGKQAGHF	7	1	846.99	1249.17	3.9	2.5	0	0	0	0	0.074	NoExpr	Pending	1	85484519	85484520	85484520	C	T	missense_variant
1-84964290-84964291-G-A		1		AC167036.1	R208Q	1	IYREGKQAGHF	7	1	846.99	1249.17	3.9	2.5	0	0	0	0	0.027	NoExpr	Pending	1	84964290	84964291	84964291	G	A	missense_variant
17-34907588-34907589-A-G		1		Ehmt2	N782S	1	SHLEVARYM	1	1	848.69	1995.68	2	3.2	80.788	0	0	0	0.14	NoExpr	Pending	17	34907588	34907589	34907589	A	G	missense_variant
11-65989859-65989860-T-G		1	1	Dnah9	N2637T	1	KFGTFPTTL	4	2	849.36	1132.64	2.5	3.2	0	0	0	0	0.263	NoExpr	Pending	11	65989859	65989860	65989860	T	G	missense_variant
4-116337488-116337489-G-A		1		Mast2	P202S	1	HGHTGNSSL	8	1	849.36	1393.99	2.5	2.1	44.641	0	0	0	0.219	NoExpr	Pending	4	116337488	116337489	116337489	G	A	missense_variant
3-138286841-138286842-G-C		1		Adh1	E240Q	1	KELGATQCI	7	1	849.69	2905.76	2.5	5.2	0.219	0	0	0	0.063	NoExpr	Pending	3	138286841	138286842	138286842	G	C	missense_variant
7-12041929-12041930-A-T		1		Vmn1r77	K211I	1	LYIHKRQVRNI	3	1	849.8	1806.68	0.31	3.3	0	0	0	0	0.056	NoExpr	Pending	7	12041929	12041930	12041930	A	T	missense_variant
1-66473470-66473471-A-C		1		Unc80	N180T	1	TFQTSMATV	4	1	855.38	784.45	4.199	3.9	0	0	0	0	0.225	NoExpr	Pending	1	66473470	66473471	66473471	A	C	missense_variant

ID	H-2-Dd	H-2-Kd	H-2-Ld	Gene	AA Change	Num Passing Transcripts	Best Peptide	Pos	Num Passing Peptides	IC50 MT	IC50 WT	%ile MT	%ile WT	RNA Expr	RNA VAF	Allele Expr	RNA Depth	DNA VAF	Tier	Evaluation	CHROM	START	END	POS	REF_y	ALT_y	biotype
1-88355257-88355258-C-G		1		Trpm8	L756V	1	AYVVLMDFHSV	4	1	855.85	1287.89	1.2	1.8	0	0	0	0	0.244	NoExpr	Pending	1	88355257	88355258	88355258	C	G	missense_variant
14-50414141-50414142-T-G		1		Olfr738	I199M	1	SLMEMTSST	3	1	857.55	549.87	2.4	1.8	0	0	0	0	0.041	NoExpr	Pending	14	50414141	50414142	50414142	T	G	missense_variant
1-116184441-116184442-T-G		1	1	Cntnap5a	S487R	1	IYSGKRYYF	6	2	858.68	1101.11	3.301	4.2	0	0	0	0	0.121	NoExpr	Pending	1	116184441	116184442	116184442	T	G	missense_variant
7-85955978-85955979-G-T		1		Vmn2r74	P487Q	1	EHLQSGQQI	4	1	863.16	671.568	1.4	1.1	0	0	0	0	0.029	NoExpr	Pending	7	85955978	85955979	85955979	G	T	missense_variant
6-136400878-136400879-A-G		1		E330021D16Rik	S318P	1	SIEPVILQI	4	1	864.68	953.119	4.301	4.8	0	0	0	0	0.829	NoExpr	Pending	6	136400878	136400879	136400879	A	G	missense_variant
3-9264685-9264686-T-G		1		Zbtb10	F368C	1	KICKAHKNI	3	1	864.68	476.884	3.301	1.8	0.167	0	0	0	0.325	NoExpr	Pending	3	9264685	9264686	9264686	T	G	missense_variant
7-10088027-10088028-C-T		1	1	Vmn2r51	V577I	1	CIQKGITFL	6	1	865.15	875.165	2.199	2.3	0	0	0	0	0.039	NoExpr	Pending	7	10088027	10088028	10088028	C	T	missense_variant
17-37151436-37151437-T-G		1		Olfr93	E178D	1	FLCDVPSLI	4	1	865.49	813.317	2.5	2	0	0	0	0	0.21	NoExpr	Pending	17	37151436	37151437	37151437	T	G	missense_variant
6-120952378-120952379-G-C		1		Mical3	A1842G	1	KRLHRGQII	6	1	867.14	814.873	2.699	2.6	4.197	0	0	0	0.397	NoExpr	Pending	6	120952378	120952379	120952379	G	C	missense_variant
7-8472031-8472032-G-C		1		Vmn2r45	L666V	1	VFTVAVSTV	6	1	869.7	845.454	1.3	0.9	0	0	0	0	0.02	NoExpr	Pending	7	8472031	8472032	8472032	G	C,A	missense_variant
14-69800781-69800782-G-C		1		Rhobtb2	P10A	1	DYERANVETI	5	1	869.88	1163.16	0.7	0.7	5.493	0	0	0	0.051	NoExpr	Pending	14	69800781	69800782	69800782	G	C	missense_variant
17-36056612-36056613-T-A		2		Gm11127	N278Y	1	YYTCHVHHEGL	1	2	870.38	1686.38	2.6	4.8	24.996	0	0	0	0.005	NoExpr	Pending	17	36056612	36056613	36056613	T	A	missense_variant
17-35383075-35383076-A-T		2		H2-Q4	N305Y	1	YYTCHVHHEGL	1	2	870.38	1686.38	2.6	4.8	256.73	0	0	0	0.054	NoExpr	Pending	17	35383075	35383076	35383076	A	T	missense_variant
17-36030860-36030861-A-G		2		H2-T23	Y282H	1	YYTCHVHHEGL	7	2	870.38	1276.5	2.6	4.4	76.781	0	0	0	0.039	NoExpr	Pending	17	36030860	36030861	36030861	A	G,C	missense_variant
2-88487514-88487515-C-T		1		Olfr1184	T261I	1	IFAYLRPPI	9	1	871.49	14726.2	2.301	19	0	0	0	0	0.365	NoExpr	Pending	2	88487514	88487515	88487515	C	T	missense_variant
2-86255419-86255420-T-G		1		Olfr1049	N91T	1	NFVVDKNTI	8	1	873.15	1791.68	2.6	4.6	0	0	0	0	0.031	NoExpr	Pending	2	86255419	86255420	86255420	T	G	missense_variant
7-12321454-12321455-A-C		1		Vmn1r83	L225R	1	KHIHSAQQR	9	1	873.49	119.47	3.301	0.36	0	0	0	0	0.251	NoExpr	Pending	7	12321454	12321455	12321455	A	C	missense_variant
12-113578609-113578611-TG-CA		1		Ighv5-2	TM82-83TV	1	YYPDTVERRFI	6	1	873.88	887.05	0.6	0.5	0	0	0	0	0.016	NoExpr	Pending	12	113578609	113578611	113578610	TG	CA	missense_variant
10-130034453-130034454-G-A		1		Olfr821	S276N	1	KGIALLNTS	7	1	874.09	375.868	4.301	1.9	0	0	0	0	0.637	NoExpr	Pending	10	130034453	130034454	130034454	G	A	missense_variant
4-132833090-132833091-G-A		1		Ppp1r8	R100W	1	IWLEPHKPQQI	2	1	874.09	1785.2	1.6	3.5	54.952	0	0	0	0.154	NoExpr	Pending	4	132833090	132833091	132833091	G	A	missense_variant
13-8836797-8836798-C-T		1		Wdr37	V318I	1	LYDVETSELI	10	1	875.91	2192.64	0.8	0.81	10.152	0	0	0	0.534	NoExpr	Pending	13	8836797	8836798	8836798	C	T	missense_variant
1-126025950-126025951-C-T		1		Nckap5	A1023T	1	HHALGTTHM	7	1	876.96	3937.4	3.1	14	0.012	0	0	0	0.39	NoExpr	Pending	1	126025950	126025951	126025951	C	T	missense_variant
12-114896432-114896434-AA-GT		2		Ighv1-37	F52Y	1	YYMNWVKQS	2	2	877.18	8032.38	2.6	18	0	0	0	0	0.022	NoExpr	Pending	12	114896432	114896434	114896433	AA	GT	missense_variant
13-63511819-63511820-A-C		1		Ptch1	S1340A	1	TATAMGSSV	2	1	877.18	743.173	2.301	2.2	10.315	0	0	0	0.126	NoExpr	Pending	13	63511819	63511820	63511820	A	C	missense_variant
X-74303841-74303842-G-A		1		Atp6ap1	D410N	1	SYASNCAGF	5	1	877.53	1535.54	2.6	4.2	194.82	0	0	0	0.035	NoExpr	Pending	X	74303841	74303842	74303842	G	A	missense_variant
7-141638885-141638886-G-A		1		Muc6	P1958L	1	LHLSQPSTV	1	1	880.15	3296.78	1.6	4.6	0.02	0	0	0	0.121	NoExpr	Pending	7	141638885	141638886	141638886	G	A	missense_variant
15-30647189-30647190-A-G		1		Ctnnd2	Q344R	1	PIHRLSSTI	4	1	881.63	1208.06	2.1	2.3	0.501	0	0	0	0.132	NoExpr	Pending	15	30647189	30647190	30647190	A	G	missense_variant
1-85635574-85635575-A-C		1		Sp140	N330T	1	NFLPTPPRI	5	1	881.91	1663.76	3.4	7.3	95.257	0	0	0	0.02	NoExpr	Pending	1	85635574	85635575	85635575	A	C	missense_variant
12-53947199-53947200-A-G		1		Npas3	H204R	1	DYVRPGDHV	4	1	882.45	1906.3	3.6	3.9	0.331	0	0	0	0.511	NoExpr	Pending	12	53947199	53947200	53947200	A	G	missense_variant
4-126163085-126163086-C-G		1		Sh3d21	E14D	1	YRAQTDDEL	6	1	883.26	899.684	2.801	2.9	0.32	0	0	0	0.062	NoExpr	Pending	4	126163085	126163086	126163086	C	G	missense_variant
16-32754904-32754905-C-T		1		Muc4	A1593V	1	SHVPSMSSS	3	1	883.6	437.342	2.4	1.2	0.047	0	0	0	0.038	NoExpr	Pending	16	32754904	32754905	32754905	C	T	missense_variant
18-36940296-36940297-C-T		2		Pcdha2	T327I	1	KGIPAMSGH	3	2	883.6	2314.55	4.398	12	0	0	0	0	0.154	NoExpr	Pending	18	36940296	36940297	36940297	C	T	missense_variant
6-71963681-71963682-T-G		1		Polr1a	I1000S	1	GYLQRCISKHL	8	1	884.87	884.871	1.2	1.4	35.688	0	0	0	0.154	NoExpr	Pending	6	71963681	71963682	71963682	T	G	missense_variant
2-112092469-112092470-G-A		1	1	Olfr1314	T77I	1	ICPKMIYDL	1	2	885.12	1569.75	2.6	4.6	0	0	0	0	0.219	NoExpr	Pending	2	112092469	112092470	112092470	G	A	missense_variant
11-59122858-59122859-C-T		1		Obscn	R1054H	1	KEQQAHSEV	6	1	885.3	807.402	2.301	2	0.494	0	0	0	0.042	NoExpr	Pending	11	59122858	59122859	59122859	C	T	missense_variant
12-110665717-110665718-T-C		1		Dync1h1	I4474T	1	HYTVPAGMTVT	11	1	887.05	539.18	2.199	0.21	55.899	0	0	0	0.084	NoExpr	Pending	12	110665717	110665718	110665718	T	C	missense_variant
2-129630101-129630102-T-G		1		Sirpa	V487G	1	TYADLDMGHLS	8	1	890.66	884.525	2.699	2.8	52.411	0	0	0	0.231	NoExpr	Pending	2	129630101	129630102	129630102	T	G	missense_variant
16-87722516-87722517-G-A		1		Bach1	R565H	1	IHRRSKNRI	2	1	893.49	1381.21	2.6	3.8	20.495	0	0	0	0.247	NoExpr	Pending	16	87722516	87722517	87722517	G	A	missense_variant
2-25269165-25269166-C-G		1		Tprn	A705G	1	SYTPKHSMG	9	1	895.55	1054.61	2.301	2.6	6.66	0	0	0	0.028	NoExpr	Pending	2	25269165	25269166	25269166	C	G	missense_variant
12-75966729-75966730-T-G		1		Syne2	F2898L	1	AYKDANVLERL	8	1	897.18	897.181	1.2	1.2	27.747	0	0	0	0.079	NoExpr	Pending	12	75966729	75966730	75966730	T	G	missense_variant
11-57769625-57769626-A-G		1		Galnt10	I297V	1	MYYKRVPI	6	1	898.67	1022.35	0.7	0.8	11.002	0	0	0	0.51	NoExpr	Pending	11	57769625	57769626	57769626	A	G	missense_variant
10-90049876-90049877-C-T		1		Anks1b	A145V	1	QYGHSEVVVVL	9	1	899.25	546.651	3.699	1.4	4.303	0	0	0	0.593	NoExpr	Pending	10	90049876	90049877	90049877	C	T	missense_variant
12-114829466-114829467-C-T		1		Ighv1-31	G50S	1	GYSFTSYYM	6	1	899.68	931.301	1.3	1.7	0	0	0	0	0.02	NoExpr	Pending	12	114829466	114829467	114829467	C	T	missense_variant
12-114946152-114946153-C-T		1		Ighv1-43	G50S	1	GYSFTSYYM	6	1	899.68	931.301	1.3	1.7	0	0	0	0	0.016	NoExpr	Pending	12	114946152	114946153	114946153	C	T	missense_variant
17-23345177-23345178-G-C		1		Vmn2r115	W108S	1	SESKLMSFM	1	1	899.68	1710.37	2.6	4.6	0	0	0	0	0.369	NoExpr	Pending	17	23345177	23345178	23345178	G	C	missense_variant
2-113525520-113525521-A-C		1		Fmn1	K867T	1	TSSSSQHTI	8	1	901.76	2958.63	2.1	6.4	4.02	0	0	0	0.081	NoExpr	Pending	2	113525520	113525521	113525521	A	C	missense_variant
17-13051936-13051937-C-A		1		Gpr31b	R115L	1	RYLLVVHPRL	4	1	902.46	244.76	1.3	0.38	0.285	0	0	0	0.063	NoExpr	Pending	17	13051936	13051937	13051937	C	A	missense_variant
9-106463368-106463369-G-A		1		Pcbp4	A388T	1	YTAKMATAN	7	1	902.93	4106.86	4.5	18	46.758	0	0	0	0.444	NoExpr	Pending	9	106463368	106463369	106463369	G	A	missense_variant
7-132759533-132759534-G-A		1		Fam53b	P255L	1	ANSTPASTL	9	1	908.01	8205.22	2.6	18	11.164	0	0	0	0.512	NoExpr	Pending	7	132759533	132759534	132759534	G	A	missense_variant
19-8978063-8978064-G-T		1		Eef1g	V426L	1	SWEGTFQHL	9	1	908.36	1567.99	1.9	3.2	612.4	0	0	0	0.055	NoExpr	Pending	19	8978063	8978064	8978064	G	T	missense_variant
1-118662019-118662020-C-T		1		Tfcp2l1	T270I	1	NNIPSPSYN	3	1	912.2	2371.87	2.699	6.2	0.051	0	0	0	0.142	NoExpr	Pending	1	118662019	118662020	118662020	C	T	missense_variant
14-50414145-50414146-A-C		1		Olfr738	M201L	1	IELTSSTLSSL	3	1	912.75	1076.17	2.1	2.3	0	0	0	0	0.029	NoExpr	Pending	14	50414145	50414146	50414146	A	C	missense_variant
1-63592636-63592637-C-T		1		Adam23	T824I	1	RRFDPIQQGPI	6	1	913.86	2659.99	3	3	0.131	0	0	0	0.57	NoExpr	Pending	1	63592636	63592637	63592637	C	T	missense_variant
2-66700872-66700873-C-A		1		Scn7a	M553I	1	IGIYTIEII	8	1	914.3	809.264	2.699	2.4	0.101	0	0	0	0.049	NoExpr	Pending	2	66700872	66700873	66700873	C	A	missense_variant
8-105375702-105375703-A-G		1		Plekhg4	N108S	1	AFSELESLL	7	1	914.66	2405.8	2.5	4.7	0.117	0	0	0	0.283	NoExpr	Pending	8	105375702	105375703	105375703	A	G	missense_variant
4-156334327-156334328-G-A		1		Vmn2r-ps159	V302I	1	WITTSQWDI	9	1	918.52	5471.29	3	14	0	0	0	0	0.01	NoExpr	Pending	4	156334327	156334328	156334328	G	A	missense_variant
1-51755732-51755733-C-T		1		Myo1b	S1066N	1	FLFNSDHLI	4	1	918.88	684.321	2.1	1.4	3.813	0	0	0	0.233	NoExpr	Pending	1	51755732	51755733	51755733	C	T	missense_variant
7-85955985-85955986-G-T		1		Vmn2r74	H485N	1	ENLPSGQQI	2	1	918.88	671.568	2.699	1.1	0	0	0	0	0.028	NoExpr	Pending	7	85955985	85955986	85955986	G	T	missense_variant
7-85620587-85620588-A-T		1	1	Vmn2r71	I436F	1	FDSWKMFSF	9	2	920.09	2772.24	3.1	6	0	0	0	0	0.033	NoExpr	Pending	7	85620587	85620588	85620588	A	T	missense_variant
8-3876306-3876307-C-T		1		Cd209d	A133T	1	WHNSTTTCQEL	7	1	922.68	1378.08	1.8	2.8	0.069	0	0	0	0.155	NoExpr	Pending	8	3876306	3876307	3876307	C	T	missense_variant
2-160957180-160957181-G-A		1		Chd6	P2328L	1	AGQTTLSTTHL	11	1	922.68	7208.66	1.7	14	12.424	0	0	0	0.047	NoExpr	Pending	2	160957180	160957181	160957181	G	A	missense_variant
14-53073258-53073259-T-C		1		Trav13d-4	F106L	1	SSQTTDSGTYL	11	1	922.68	2469.77	1.7	4.8	0	0	0	0	0.059	NoExpr	Pending	14	53073258	53073259	53073259	T	C	missense_variant

ID	H-2-Dd	H-2-Kd	H-2-Ld	Gene	AA Change	Num Passing Transcripts	Best Peptide	Pos	Num Passing Peptides	IC50 MT	IC50 WT	%ile MT	%ile WT	RNA Expr	RNA VAF	Allele Expr	RNA Depth	DNA VAF	Tier	Evaluation	CHROM	START	END	POS	REF_y	ALT_y	biotype
7-7312964-7312965-C-T		1		Vmn2r30	S623N	1	ANNRNLSYL	5	1	927.39	316.293	2.699	1	0.002	0	0	0	0.273	NoExpr	Pending	7	7312964	7312965	7312965	C	T	missense_variant
X-74303875-74303876-T-C		1	1	Atp6ap1	M421T	1	SPGIWTGLL	6	2	928.97	1035.33	3.199	2.8	194.82	0	0	0	0.037	NoExpr	Pending	X	74303875	74303876	74303876	T	C	missense_variant
17-37624371-37624373-CC-GT		1		Olfr116	AG87-88AR	1	SSLARSGYI	5	1	929.16	388.269	2.699	1.2	0	0	0	0	0.023	NoExpr	Pending	17	37624371	37624373	37624372	CC	GT	missense_variant
18-80972970-80972971-C-T		1		Sall3	E581K	1	GLNNTKSGI	6	1	929.16	1143.12	2.699	3.2	0	0	0	0	0.213	NoExpr	Pending	18	80972970	80972971	80972971	C	T	missense_variant
1-90214837-90214838-C-T		1		Ackr3	L340F	1	KYSAKTGFTKL	8	1	930.12	1139.58	0.7	0.7	56.08	0	0	0	0.523	NoExpr	Pending	1	90214837	90214838	90214838	C	T	missense_variant
1-85610765-85610766-G-A		1		Sp140	V75I	1	EYQETCKNLIP	10	1	937.57	2427.98	1.6	2.6	95.257	0	0	0	0.022	NoExpr	Pending	1	85610765	85610766	85610766	G	A,C	missense_variant
11-51593953-51593954-A-C		1		Phykpl	E247A	1	GGLFVADAI	8	1	939.92	2046.87	2.699	4.6	9.185	0	0	0	0.25	NoExpr	Pending	11	51593953	51593954	51593954	A	C	missense_variant
13-67413185-67413186-G-A		1		Zfp459	P48S	1	GLADSKSQL	7	1	939.92	8536.11	2.699	15	0.226	0	0	0	0.017	NoExpr	Pending	13	67413185	67413186	67413186	G	A	missense_variant
7-103328662-103328663-A-G		1		Olfr598	H59R	1	IIIPAERSL	7	1	940.29	553.681	2.699	1.8	0	0	0	0	0.049	NoExpr	Pending	7	103328662	103328663	103328663	A	G	missense_variant
10-58224914-58224915-A-C		1		AW822073	W6G	1	WQAGQEQAL	4	1	942.45	1285.55	2.699	3.1	0	0	0	0	0.07	NoExpr	Pending	10	58224914	58224915	58224915	A	C	missense_variant
5-136059811-136059812-C-T		1		Upk3bl	P55L	1	STFTLEQLL	8	1	942.45	740.048	2.699	2.2	11.108	0	0	0	0.587	NoExpr	Pending	5	136059811	136059812	136059812	C	T	missense_variant
1-85610751-85610752-C-T		1		Sp140	T70I	1	EYQEICKNLV	5	1	942.86	704.109	1.7	1	95.257	0	0	0	0.023	NoExpr	Pending	1	85610751	85610752	85610752	C	T	missense_variant
3-94343347-94343348-T-G		1		Them5	F73V	1	LYQEVLEKTKS	5	1	942.86	3454.02	1.7	6.7	0	0	0	0	0.117	NoExpr	Pending	3	94343347	94343348	94343348	T	G	missense_variant
16-32751866-32751867-C-T		1		Muc4	P582S	1	KGTSSNSQT	7	1	948.62	7366.35	4.5	27	0.047	0	0	0	0.008	NoExpr	Pending	16	32751866	32751867	32751867	C	T	missense_variant
9-73932847-73932848-C-T		1		Unc13c	M240I	1	CISQTHDVI	9	1	948.62	3598.24	3	9.2	9.353	0	0	0	0.244	NoExpr	Pending	9	73932847	73932848	73932848	C	T	missense_variant
2-76753052-76753053-C-T		1		Ttn	E20753K	1	KHQKVGDDAW	1	1	950.34	3033.45	2.9	5.8	0.231	0	0	0	0.451	NoExpr	Pending	2	76753052	76753053	76753053	C	T	missense_variant
6-5168386-5168387-T-A		1		Pon1	K340I	1	KGILLIGTV	3	1	951.17	6224.29	2.199	15	0	0	0	0	0.036	NoExpr	Pending	6	5168386	5168387	5168387	T	A	missense_variant
2-88486836-88486837-C-T		1		Olfr1184	A35V	1	CYLVILCGNLL	4	1	952.24	1987.97	1.5	0.7	0	0	0	0	0.422	NoExpr	Pending	2	88486836	88486837	88486837	C	T	missense_variant
17-13883818-13883819-G-C		1		Afdn	G1139A	1	LYNNSAQNA	9	1	952.99	809.264	1.5	2.5	41.044	0	0	0	0.064	NoExpr	Pending	17	13883818	13883819	13883819	G	C	missense_variant
8-110519286-110519287-A-G		1		Hydin	T2211A	1	APQVQISSSPL	1	1	953.12	1183.39	4.102	3.6	0	0	0	0	0.212	NoExpr	Pending	8	110519286	110519287	110519287	A	G	missense_variant
11-58529827-58529828-C-T		1		Olfr330	V53I	1	LLILSDTHL	3	1	953.37	4626.58	2.699	6.5	0	0	0	0	0.045	NoExpr	Pending	11	58529827	58529828	58529828	C	T	missense_variant
17-35266194-35266195-C-T		1		H2-D1	P302S	1	SSTDSYMVI	1	1	957.39	9910.37	2.801	20	1425.5	0	0	0	0.039	NoExpr	Pending	17	35266194	35266195	35266195	C	T	missense_variant
11-76076501-76076502-C-T		1		Vps53	S600N	1	FSTVISNSI	7	1	957.39	451.95	2.6	1.3	19.99	0	0	0	0.244	NoExpr	Pending	11	76076501	76076502	76076502	C	T	missense_variant
2-148700412-148700413-G-A		1		Napb	A209V	1	FKVALCHFI	3	1	959.6	484.273	2.1	1	2.369	0	0	0	0.396	NoExpr	Pending	2	148700412	148700413	148700413	G	A	missense_variant
17-33300998-33300999-T-C		1		Zfp955b	L68S	1	NYPQLQRNSR	9	1	961.34	2116.95	0.8	3.5	16.823	0	0	0	0.032	NoExpr	Pending	17	33300998	33300999	33300999	T	C	missense_variant
3-107458508-107458509-G-C		1		Kcnc4	P128A	1	CPADVCGAL	8	1	961.81	1914.65	3.1	3.4	0.009	0	0	0	0.074	NoExpr	Pending	3	107458508	107458509	107458509	G	C	missense_variant
X-73259107-73259108-A-C		1		Xlr3c	H138Q	1	KQAETLSNM	2	1	961.81	500.27	2.801	1.2	0	0	0	0	0.033	NoExpr	Pending	X	73259107	73259108	73259108	A	C	missense_variant
7-56328748-56328749-G-A		1		Oca2	R555H	1	IHVWRLTAQHI	10	1	963.49	1318.61	1	1	0	0	0	0	0.173	NoExpr	Pending	7	56328748	56328749	56328749	G	A	missense_variant
2-110745913-110745914-T-G		1		Ano3	Q431H	1	NESQVSHEI	7	1	970.71	620.998	2.801	2	0.006	0	0	0	0.298	NoExpr	Pending	2	110745913	110745914	110745914	T	G	missense_variant
10-130210347-130210348-T-G		1		Olfr827	S261R	1	SHLGVVRLL	7	1	970.71	305.14	2.801	0.8	0	0	0	0	0.231	NoExpr	Pending	10	130210347	130210348	130210348	T	G	missense_variant
5-75163746-75163747-G-A		1		Pdgfra	V88I	1	LFVTVLEVI	9	1	971.09	3900.23	1.4	5.2	23.453	0	0	0	0.432	NoExpr	Pending	5	75163746	75163747	75163747	G	A	missense_variant
12-114896418-114896419-T-A		1		Ighv1-37	K57M	1	GYFMNWVM	8	1	971.54	994.489	0.8	1.4	0	0	0	0	0.065	NoExpr	Pending	12	114896418	114896419	114896419	T	A	missense_variant
6-57926211-57926212-T-A		1		Vmn1r23	T194S	1	VGVMLTSSI	7	1	975.57	1521.46	2.5	3.5	0	0	0	0	0.157	NoExpr	Pending	6	57926211	57926212	57926212	T	A	missense_variant
4-147510858-147510860-AC-TA		1		Zfp982	Y46L	1	LYIDVMLENL	10	1	983.2	4174.84	0.9	7.3	1.461	0	0	0	0.058	NoExpr	Pending	4	147510858	147510860	147510859	AC	TA	missense_variant
4-68792849-68792850-G-A		1		Brinp1	P374S	1	CRHNSNHQL	5	1	984.22	3244.07	2.801	7.5	0	0	0	0	0.741	NoExpr	Pending	4	68792849	68792850	68792850	G	A	missense_variant
3-122549201-122549202-G-T		1		Fnbp1l	R410S	1	RRKKLQQSI	8	1	984.22	2153.23	2.801	5.8	3.675	0	0	0	0.122	NoExpr	Pending	3	122549201	122549202	122549202	G	T	missense_variant
X-60293649-60293650-C-T		1		Atp11c	G223R	1	YRFVRRISI	5	1	984.56	466.675	2.199	0.8	23.144	0	0	0	0.344	NoExpr	Pending	X	60293649	60293650	60293650	C	T	missense_variant
17-35320849-35320850-T-G		1		H2-Q1	S32A	1	RYFETAVS	6	1	984.56	1557.62	1.4	2	11.863	0	0	0	0.109	NoExpr	Pending	17	35320849	35320850	35320850	T	G	missense_variant
2-132879658-132879659-G-C		1		Lrrn4	C79W	1	SLESLPSWL	8	1	984.56	1290.38	2.801	3.5	0	0	0	0	0.145	NoExpr	Pending	2	132879658	132879659	132879659	G	C	missense_variant
7-25331086-25331087-C-G		1		Megf8	C488W	1	FYHLGWHQWV	6	1	989.6	613.42	1	0.57	23.991	0	0	0	0.095	NoExpr	Pending	7	25331086	25331087	25331087	C	G	missense_variant
9-112136557-112136558-C-T		1		Arpp21	G415E	1	AYSENEMGGQ	6	1	990.56	1015.57	1.8	2.1	0.025	0	0	0	0.483	NoExpr	Pending	9	112136557	112136558	112136558	C	T	missense_variant
2-86217338-86217339-C-T		1		Olfr1046	D124N	1	NRYVAICKPLL	1	1	992.45	978.836	3.1	2.9	0	0	0	0	0.041	NoExpr	Pending	2	86217338	86217339	86217339	C	T	missense_variant
12-16733403-16733404-G-A		1		Greb1	P110S	1	LRLVSISSESI	10	1	995.13	935.147	3.6	2.6	0	0	0	0	0.052	NoExpr	Pending	12	16733403	16733404	16733404	G	A	missense_variant
15-36234652-36234653-A-T		1		Spag1	K853I	1	LYLSIAERFK	5	1	995.27	2646.31	1.8	5.8	1.663	0	0	0	0.2	NoExpr	Pending	15	36234652	36234653	36234653	A	T	missense_variant
X-136734410-136734411-T-G		1		Morf4l2	K23Q	1	FKQPTRSNM	3	1	996	3786.69	2.1	7	312.97	0	0	0	0.133	NoExpr	Pending	X	136734410	136734411	136734411	T	G	missense_variant

Supplementary Table 4. The potential neoantigens in CT26-iPSC vaccined tumors.

ID	H-2-Dd	H-2-Kd	H-2-Ld	Gene	AA Change	Num Passing Transcripts	Best Peptide	Pos	Num Passing Peptides	IC50 MT	IC50 WT	%ile MT	%ile WT	RNA Expr	RNA VAF	Allele Expr	RNA Depth	DNA VAF	Tier	Evaluation	CHROM	START	END	POS	REF_y	ALT_y	biotype
9-19477584-19477585-C-T		5	1	Olfr850	V222I	1	SYIHIISSI	3	6	5.085	13.034	0.1	0.1	0	0	0	0	0.253	NoExpr	Pending	9	19477584	19477585	19477585	C	T	missense_variant
2-98662311-98662312-T-C		1	5	Gm10801	S26P	1	FPFSMIFSF	2	6	5.176	100.462	0.1	0.4	0.12	0	0	0	0.016	NoExpr	Pending	2	98662311	98662312	98662312	T	C	missense_variant
6-90269649-90269650-A-G		3	2	Vmn1r54	H182R	1	APMSYSFRL	8	5	14.633	9.289	0.2	0.1	0	0	0	0	0.043	NoExpr	Pending	6	90269649	90269650	90269650	A	G	missense_variant
5-94535033-94535034-A-C		4		Gm3139	Q62H	1	HYLPVGSLI	1	4	14.81	21.042	0.1	0.1	0	0	0	0	0.036	NoExpr	Pending	5	94535033	94535034	94535034	A	C	missense_variant
4-140827908-140827910-CA-TG		4		Padi1	YV306-307YM	1	LYMCSVTDI	3	4	16.01	36.37	0.1	0.2	0.131	0	0	0	0.213	NoExpr	Pending	4	140827908	140827910	140827909	CA	TG	missense_variant
11-87889593-87889594-C-T		1	3	Olfr462	V101I	1	GCMAQIFFF	6	4	20.027	24.333	0.2	0.2	0	0	0	0	0.031	NoExpr	Pending	11	87889593	87889594	87889594	C	T	missense_variant
6-141943448-141943449-G-C		4		Slco1a1	Q46E	1	VYMNSMLTEI	9	4	20.57	20.78	0.2	0.2	0	0	0	0	0.369	NoExpr	Pending	6	141943448	141943449	141943449	G	C	missense_variant
2-79333634-79333635-G-A		1	1	Cerkl	T447I	1	FPFVEIYII	6	2	21.429	32.584	0.2	0.2	0.018	0	0	0	0.363	NoExpr	Pending	2	79333634	79333635	79333635	G	A	missense_variant
15-57259135-57259136-T-A		5		Slc22a22	Q148H	1	KYYAHATSL	5	5	21.941	8.89	0.2	0.1	0	0	0	0	0.167	NoExpr	Pending	15	57259135	57259136	57259136	T	A	missense_variant
3-14770865-14770866-C-T		1		Car1	S131N	1	KYSNASEAI	4	1	22.712	16.915	0.2	0.1	0	0	0	0	0.521	NoExpr	Pending	3	14770865	14770866	14770866	C	T	missense_variant
14-50736547-50736548-C-A		1	3	Olfr749	V205F	1	YAQSSFFLF	7	3	23.442	870.623	0.2	2.6	0	0	0	0	0.079	NoExpr	Pending	14	50736547	50736548	50736548	C	A	missense_variant
19-13410933-13410934-C-T		1		Olfr1469	R122C	1	CYAAVCKPL	1	1	24.071	38.53	0.2	0.2	0	0	0	0	0.33	NoExpr	Pending	19	13410933	13410934	13410934	C	T	missense_variant
7-7241578-7241579-C-T		2		Vmn2r29	C432Y	1	HYLKVHSFL	2	2	24.271	519.111	0.1	1.6	0	0	0	0	0.039	NoExpr	Pending	7	7241578	7241579	7241579	C	T	missense_variant
14-50281886-50281887-C-T		1	1	Olfr732	R122H	1	HYIAICRPL	1	2	28.5	22.84	0.2	0.2	0	0	0	0	0.057	NoExpr	Pending	14	50281886	50281887	50281887	C	T	missense_variant
17-37299756-37299757-T-A		6	1	Olfr101	N222Y	1	SYFYIITYL	8	7	29.18	38.823	0.2	0.2	0	0	0	0	0.04	NoExpr	Pending	17	37299756	37299757	37299757	T	A	missense_variant
4-156331348-156331350-AG-TA		3	1	Vmn2r-ps159	PV56-57PI	1	FYLGAADTPI	10	4	30.19	81.403	0.1	0.1	0	0	0	0	0.06	NoExpr	Pending	4	156331348	156331350	156331349	AG	TA	missense_variant
17-36032407-36032408-G-A	1	3	3	H2-T23	R26W	1	SPHSLWYFT	6	7	30.761	30.549	0.2	0.2	36.919	0	0	0	0.141	NoExpr	Pending	17	36032407	36032408	36032408	G	T,A	missense_variant
17-38209033-38209034-C-T		4		Olfr135	P263L	1	MYLQPLSSI	6	4	31.48	36.96	0.1	0.1	0	0	0	0	0.027	NoExpr	Pending	17	38209033	38209034	38209034	C	T	missense_variant
19-33749463-33749464-G-A		1		Lipo2	P58S	1	GYILSINRI	5	1	31.496	99.83	0.1	0.23	0.002	0	0	0	0.492	NoExpr	Pending	19	33749463	33749464	33749464	G	A	missense_variant
16-58823835-58823836-C-T		1	2	Olfr175	S291N	1	IPLLNPFIYNL	10	3	31.74	95.29	0.03	0.05	0	0	0	0	0.326	NoExpr	Pending	16	58823835	58823836	58823836	C	T	missense_variant
2-85984169-85984170-T-C		2		Olfr1030	F110S	1	CYFSIAMVI	4	2	32.03	60.85	0.2	0.25	0	0	0	0	0.034	NoExpr	Pending	2	85984169	85984170	85984170	T	C	missense_variant
9-19360965-19360966-G-C		1	1	Olfr846	L130V	1	RYAAICHPV	9	2	32.589	18.96	0.2	0.2	0	0	0	0	0.264	NoExpr	Pending	9	19360965	19360966	19360966	G	C	missense_variant
2-87357847-87357848-C-A		1	1	Olfr1120	P135T	1	RYVAICNTL	8	2	34.03	38.331	0.2	0.3	0	0	0	0	0.07	NoExpr	Pending	2	87357847	87357848	87357848	C	A	missense_variant
11-120956420-120956421-G-A		1		Slc16a3	R145H	1	RYFNKRHPI	7	1	35.11	82.08	0.2	0.2	7.561	0	0	0	0.07	NoExpr	Pending	11	120956420	120956421	120956421	G	A	missense_variant
10-78879842-78879843-T-C		3		Olfr1355	S224P	1	SYFKIVPSI	7	3	35.339	4.548	0.2	0.1	0.027	0	0	0	0.038	NoExpr	Pending	10	78879842	78879843	78879843	T	C	missense_variant
11-71114434-71114435-G-T		5		Nlrp1a	H651Q	1	YYAVQSLCTTL	5	5	35.76	92.081	0.2	0.2	0	0	0	0	0.101	NoExpr	Pending	11	71114434	71114435	71114435	G	T	missense_variant
2-87071285-87071286-G-A		2		Olfr1107	P283S	1	FYTLVISML	7	2	35.817	280.711	0.2	0.66	0	0	0	0	0.396	NoExpr	Pending	2	87071285	87071286	87071286	G	A	missense_variant
17-37299642-37299644-GG-TA		2	1	Olfr101	IR259-260IS	1	TYISPASGSSL	4	3	36.67	39.46	0.1	0.1	0	0	0	0	0.023	NoExpr	Pending	17	37299642	37299644	37299643	GG	TA	missense_variant
14-52349728-52349729-G-A		5	2	Olfr1513	H106Y	1	FYYFLGSTV	3	7	36.83	22.71	0.2	0.2	0	0	0	0	0.064	NoExpr	Pending	14	52349728	52349729	52349729	G	A	missense_variant
11-58566583-58566584-T-C		3		Olfr224	I254V	1	YGAAVYTYI	5	3	36.905	104.054	0.2	0.3	0	0	0	0	0.016	NoExpr	Pending	11	58566583	58566584	58566584	T	C	missense_variant
9-39461642-39461643-T-G		6		Olfr954	F71V	1	YFLSSLSVI	8	6	37.09	40.559	0.2	0.2	0	0	0	0	0.227	NoExpr	Pending	9	39461642	39461643	39461643	T	G	missense_variant
7-43497859-43497860-A-G		2		4931406B18Rik	V316A	1	AYGAIASAL	1	2	37.71	57.84	0.2	0.2	0	0	0	0	0.379	NoExpr	Pending	7	43497859	43497860	43497860	A	G	missense_variant
10-129767874-129767875-A-C		1		Olfr808	K126N	1	RYVAICNPL	7	1	38.331	96.154	0.3	0.5	0	0	0	0	0.221	NoExpr	Pending	10	129767874	129767875	129767875	A	C	missense_variant
17-37299636-37299638-CA-TG		2		Olfr101	PA261-262PT	1	TYIRPTSGSSL	6	2	38.55	39.46	0.1	0.1	0	0	0	0	0.023	NoExpr	Pending	17	37299636	37299638	37299637	CA	TG	missense_variant
17-36032397-36032399-GT-AC		2	1	H2-T23	T29V	1	SPHSLRYFV	9	3	38.815	30.549	0.2	0.2	36.919	0	0	0	0.026	NoExpr	Pending	17	36032397	36032399	36032398	GT	AC,TG	missense_variant
13-13393294-13393295-G-C		2	2	Gpr137b	L67V	1	SYQSVFVFL	7	4	38.91	37.919	0.2	0.2	12.071	0	0	0	0.107	NoExpr	Pending	13	13393294	13393295	13393295	G	C	missense_variant
7-5481284-5481285-T-A		1	2	Vmn2r28	T639S	1	FCFLCSFFF	6	2	39.264	40.087	0.2	0.2	0	0	0	0	0.033	NoExpr	Pending	7	5481284	5481285	5481285	T	A	missense_variant
10-36993649-36993650-C-T		4	1	Hdac2	P228S	1	KYYAVNFSM	8	4	39.48	44.12	0.2	0.2	61.112	0	0	0	0.579	NoExpr	Pending	10	36993649	36993650	36993650	C	T	missense_variant
9-38402967-38402968-T-G		1	5	Olfr147	F31L	1	LPLLYLFLI	4	6	40.163	27.595	0.2	0.2	0	0	0	0	0.334	NoExpr	Pending	9	38402967	38402968	38402968	T	G	missense_variant
2-111315664-111315665-T-C	1	3	6	Olfr1280	L62P	1	FPLANLSFIDF	2	9	41.1	3987.09	0.13	2.3	0	0	0	0	0.02	NoExpr	Pending	2	111315664	111315665	111315665	T	C	missense_variant
4-49380300-49380301-G-A		2		Acnat2	P359L	1	AYLGAGHLI	3	2	41.313	298.6	0.2	0.7	0	0	0	0	0.612	NoExpr	Pending	4	49380300	49380301	49380301	G	A	missense_variant
9-39217775-39217776-A-G		5		Olfr944	I140V	1	SYQVYNSLI	4	5	41.347	53.03	0.2	0.2	0	0	0	0	0.279	NoExpr	Pending	9	39217775	39217776	39217776	A	G	missense_variant
16-96593076-96593077-T-G		2	2	Dscam	K2008N	1	NPYANSYTL	5	4	41.384	51.642	0.2	0.2	0.056	0	0	0	0.343	NoExpr	Pending	16	96593076	96593077	96593077	T	G	missense_variant
6-58955280-58955281-T-A		1		Fam13a	K369M	1	KYRPSHSDM	9	1	41.696	143.579	0.2	0.6	0.077	0	0	0	0.243	NoExpr	Pending	6	58955280	58955281	58955281	T	A	missense_variant
9-3025123-3025124-T-A		1	4	Gm10718	V196E	1	SEHFSFFTF	2	5	41.879	36.728	0.2	0.2	0	0	0	0	0.077	NoExpr	Pending	9	3025123	3025124	3025124	T	A	missense_variant
2-63980415-63980416-C-A		1		Fign	S170I	1	SYSSSTCGI	9	1	42.252	688.934	0.2	1.8	0	0	0	0	0.235	NoExpr	Pending	2	63980415	63980416	63980416	C	A	missense_variant
8-43569341-43569342-T-G		2		Adam26a	E370D	1	SYEDMYSVV	4	2	43.46	38.394	0.2	0.2	0	0	0	0	0.186	NoExpr	Pending	8	43569341	43569342	43569342	T	G	missense_variant
17-33999978-33999980-AC-GT		3	1	H2-K1	V30T	1	GPHSLRYFT	9	4	44.361	56.364	0.2	0.3	663.43	0	0	0	0.098	NoExpr	Pending	17	33999978	33999980	33999979	AC	GT	missense_variant
4-148472044-148472045-G-A		3	2	Mtor	V971M	1	MVQAITFIF	1	5	45.576	85.364	0.2	0.5	7.685	0	0	0	0.301	NoExpr	Pending	4	148472044	148472045	148472045	G	A	missense_variant
9-38047584-38047585-G-A		2		Olfr884	R121K	1	KYAAICKPL	1	2	45.576	47.592	0.3	0.3	0	0	0	0	0.519	NoExpr	Pending	9	38047584	38047585	38047585	G	A	missense_variant
17-37589975-37589976-C-T		1	1	Olfr114	V126I	1	RYIAICLPL	5	2	45.61	37.005	0.2	0.2	0	0	0	0	0.419	NoExpr	Pending	17	37589975	37589976	37589976	C	T	missense_variant
5-18267182-18267183-C-T		4		Gnai1	A301T	1	TYEEAATYI	7	4	46.73	74.97	0.2	0.4	0.093	0	0	0	0.297	NoExpr	Pending	5	18267182	18267183	18267183	C	T	missense_variant
7-107097554-107097555-T-A		1	2	Olfr17	L30H	1	EVQALLFHL	8	3	49.162	58.614	0.3	0.3	0	0	0	0	0.273	NoExpr	Pending	7	107097554	107097555	107097555	T	A	missense_variant
9-40100652-40100653-G-A		1	2	Olfr984	T279I	1	IPMLNPAIYTL	1	3	49.49	235.45	0.03	0.13	0	0	0	0	0.497	NoExpr	Pending	9	40100652	40100653	40100653	G	A	missense_variant
2-158851763-158851764-C-T		4		Dhx35	T646I	1	YYMRDVIAI	7	4	50.033	9.289	0.2	0.1	5.513	0	0	0	0.327	NoExpr	Pending	2	158851763	158851764	158851764	C	T	missense_variant
6-90269645-90269646-T-G		2	3	Vmn1r54	F181V	1	SVHLTVFIL	2	5	50.237	91.09	0.3	0.5	0	0	0	0	0.044	NoExpr	Pending	6	90269645	90269646	90269646	T	G	missense_variant
4-62499759-62499760-A-T		1	1	Hdhd3	F60I	1	AYRAQSHNI	9	2	51.297	1130.03	0.2	2.6	2.79	0	0	0	0.224	NoExpr	Pending	4	62499759	62499760	62499760	A	T	missense_variant
11-58566975-58566976-G-A		1		Olfr224	A123V	1	RYVVICHPL	4	1	51.54	37.919	0.3	0.2	0	0	0	0	0.092	NoExpr	Pending	11	58566975	58566976	58566976	G	A	missense_variant
12-113490809-113490810-T-A		5		Adam6b	L416M	1	YYNTSMTYK	6	5	51.554	59.032	0.3	0.3	0	0	0	0	0.029	NoExpr	Pending	12	113490809	113490810	113490810	T	A	missense_variant
3-103812188-103812189-G-C		3		Ap4b1	G73A	1	LYMATYAPL	4	3	51.81	115.45	0.2	0.2	12.932	0	0	0	0.117	NoExpr	Pending	3	103812188	103812189	103812189	G	C	missense_variant
4-115532645-115532646-C-T		1	4	Cyp4a10	L493F	1	VPMPLARFVL	8	5	51.895	471.752	0.17	0.6	0	0	0	0	0.029	NoExpr	Pending	4	115532645	115532646	115532646	C	T	missense_variant

16-58824247-58824248-G-T		4		Olfr175	L154M	1	AYIAGNMHSMI	7	4	52.16	61.3	0.1	0.1	0	0	0	0	0.073	NoExpr	Pending	16	58824247	58824248	58824248	G	T	missense_variant
12-113597884-113597885-C-G		1	1	Ighv5-4	L7F	1	MNFGLSFIF	7	2	52.34	1314.71	0.3	4.1	0	0	0	0	0.003	NoExpr	Pending	12	113597884	113597885	113597885	C	G	missense_variant
4-147755804-147755805-T-A		3		Zfp984	K196N	1	KYLTQRENL	8	3	52.459	80.869	0.2	0.3	15.937	0	0	0	0.368	NoExpr	Pending	4	147755804	147755805	147755805	T	A	missense_variant
14-119041595-119041596-T-A		1		Uggt2	K833I	1	IYNTVGVNI	1	1	53.62	23.05	0.2	0.2	0.312	0	0	0	0.21	NoExpr	Pending	14	119041595	119041596	119041596	T	A	missense_variant
12-114937284-114937285-G-A		5		Ighv1-42	P60L	1	YYMNWVKQSL	10	5	54.25	2326.4	0.2	1.8	0	0	0	0	0.277	NoExpr	Pending	12	114937284	114937285	114937285	G	A	missense_variant
18-21914986-21914987-C-A		2	1	Ccdc178	R782I	1	SYFNGYDIL	8	3	55.368	61.839	0.3	0.3	0	0	0	0	0.212	NoExpr	Pending	18	21914986	21914987	21914987	C	A	missense_variant
3-40910580-40910581-C-G		1		Abhd18	P86A	1	HYVPGIMAI	8	1	57.1	49.31	0.2	0.2	0.501	0	0	0	0.231	NoExpr	Pending	3	40910580	40910581	40910581	C	G	missense_variant
11-58757723-58757724-G-T		2	1	Olfr316	D20Y	1	YGHMDTFLF	1	3	57.412	155.955	0.3	0.8	0	0	0	0	0.075	NoExpr	Pending	11	58757723	58757724	58757724	G	T	missense_variant
19-39643354-39643355-T-A		3		Cyp2c67	N133Y	1	RYLGMGKRTI	2	3	57.94	2880.45	0.2	4.3	0	0	0	0	0.209	NoExpr	Pending	19	39643354	39643355	39643355	T	A	missense_variant
17-33999988-33999989-T-A	1	3	4	H2-K1	R27W	1	GPHSLWYFV	6	8	58.456	56.364	0.2	0.3	663.43	0	0	0	0.086	NoExpr	Pending	17	33999988	33999989	33999989	T	A,G	missense_variant
2-86978564-86978565-A-C		2		Olfr1100	I77S	1	SYSTVSTPKTL	6	2	59.09	96.18	0.2	0.2	0	0	0	0	0.241	NoExpr	Pending	2	86978564	86978565	86978565	A	C	missense_variant
17-37299650-37299651-G-A		5		Olfr101	T257I	1	IYIRPASGSSL	1	5	60.33	39.46	0.1	0.1	0	0	0	0	0.022	NoExpr	Pending	17	37299650	37299651	37299651	G	A	missense_variant
7-104564378-104564379-C-T		1	2	Olfr652	L53F	1	YFIFMERSL	2	3	60.407	211.393	0.3	1	0	0	0	0	0.32	NoExpr	Pending	7	104564378	104564379	104564379	C	T	missense_variant
13-22179086-22179087-A-T		4	1	Vmn1r191	C166S	1	SDNYSYFIL	5	5	60.814	56.364	0.3	0.3	0	0	0	0	0.127	NoExpr	Pending	13	22179086	22179087	22179087	A	T	missense_variant
17-37923941-37923943-CA-TG		1		Olfr128	AI125-126AV	1	RYVAVCLPL	5	1	61.12	102.603	0.3	0.5	0	0	0	0	0.019	NoExpr	Pending	17	37923941	37923943	37923942	CA	TG	missense_variant
5-130617937-130617938-C-T		2		Caln1	P114S	1	GYMSSEVEL	4	2	61.73	86.85	0.3	0.3	0.002	0	0	0	0.038	NoExpr	Pending	5	130617937	130617938	130617938	C	T	missense_variant
10-79247983-79247984-C-T		2		Vmn2r81	P64L	1	KYFLMDSDN	4	2	61.981	32.156	0.3	0.2	0	0	0	0	0.322	NoExpr	Pending	10	79247983	79247984	79247984	C	T	missense_variant
9-19249033-19249034-G-A		1	1	Olfr843	R122C	1	CYLAIVHPL	1	2	64.54	36.58	0.3	0.2	0	0	0	0	0.2	NoExpr	Pending	9	19249033	19249034	19249034	G	A	missense_variant
11-58529802-58529803-G-A		1	4	Olfr330	T61I	1	HLHIPMYFF	4	4	65.137	72.083	0.3	0.4	0	0	0	0	0.015	NoExpr	Pending	11	58529802	58529803	58529803	G	A	missense_variant
7-102659059-102659060-C-T		3	1	Olfr555	P80S	1	SSMLSIFWF	1	4	65.438	111.88	0.3	0.6	0	0	0	0	0.601	NoExpr	Pending	7	102659059	102659060	102659060	C	T	missense_variant
14-50736550-50736551-A-G		4	1	Olfr749	F204L	1	FYAQSSLVL	7	5	65.654	59.602	0.3	0.3	0	0	0	0	0.087	NoExpr	Pending	14	50736550	50736551	50736551	A	G	missense_variant
7-143536025-143536026-C-T		2		Nap1l4	V63I	1	SYIETLPKAI	10	2	65.68	164.32	0.2	0.3	60.915	0	0	0	0.34	NoExpr	Pending	7	143536025	143536026	143536026	C	T	missense_variant
6-29204633-29204634-T-C		1		Impdh1	Y282C	1	CQIAMVHYI	1	1	65.779	34.442	0.28	0.2	25.889	0	0	0	0.275	NoExpr	Pending	6	29204633	29204634	29204634	T	C	missense_variant
6-148842863-148842864-G-A		3		Caprin2	S1021F	1	KYFTFSGYL	3	3	65.842	115.15	0.3	0.4	1.879	0	0	0	0.611	NoExpr	Pending	6	148842863	148842864	148842864	G	A	missense_variant
19-4762288-4762289-A-G		4		Rbm4b	N242S	1	SYAEQTMSHL	1	4	66.07	212.94	0.2	0.32	5.602	0	0	0	0.367	NoExpr	Pending	19	4762288	4762289	4762289	A	G	missense_variant
2-180713220-180713221-C-T		1		Gid8	P7S	1	SYAEKSDEI	6	1	67.01	134.86	0.4	0.4	32.411	0	0	0	0.478	NoExpr	Pending	2	180713220	180713221	180713221	C	T	missense_variant
2-98662380-98662382-CC-TT		1	5	Gm10801	P49F	1	FPFFSVFLA	3	6	67.453	627.813	0.3	2.4	0.12	0	0	0	0.012	NoExpr	Pending	2	98662380	98662382	98662381	CC	TT,AC	missense_variant
14-32659999-32660000-T-G	1	1	4	3425401B19Rik	Q1336P	1	SPLAAPTFL	2	5	68.522	1179.86	0.3	2.8	0.025	0	0	0	0.25	NoExpr	Pending	14	32659999	32660000	32660000	T	G	missense_variant
17-57224868-57224869-C-T		1	1	C3	V254I	1	YYIDDPNGLEI	11	2	69.16	254.608	0.2	0.6	15.045	0	0	0	0.324	NoExpr	Pending	17	57224868	57224869	57224869	C	T	missense_variant
16-32751992-32751993-C-A		2		Muc4	P624T	1	SYATIMSSS	4	2	69.677	62.374	0.22	0.24	0.018	0	0	0	0.18	NoExpr	Pending	16	32751992	32751993	32751993	C	A,T	missense_variant
10-100064109-100064110-G-C		1	2	Kitl	M52I	1	LPNDYIITL	6	3	69.796	96.79	0.3	0.4	21.451	0	0	0	0.146	NoExpr	Pending	10	100064109	100064110	100064110	G	C	missense_variant
19-11472471-11472472-A-C		4	1	Ms4a6c	N76T	1	YFTSVFSVL	3	5	70.349	95.556	0.3	0.3	26.147	0	0	0	0.041	NoExpr	Pending	19	11472471	11472472	11472472	A	C	missense_variant
15-44526819-44526820-C-T		2		Pkhd1l1	P1444L	1	YRIFSVSSL	9	2	71.3	644.303	0.4	2.6	0.004	0	0	0	0.03	NoExpr	Pending	15	44526819	44526820	44526820	C	T	missense_variant
17-33269092-33269093-G-A		1		Olfr63	R123H	1	HYVAICRPL	1	1	71.795	59.09	0.4	0.4	0	0	0	0	0.363	NoExpr	Pending	17	33269092	33269093	33269093	G	A	missense_variant
9-20314065-20314066-C-T		3		Olfr18	A285T	1	VYLSSTVSL	6	3	72.576	65.133	0.4	0.3	0	0	0	0	0.315	NoExpr	Pending	9	20314065	20314066	20314066	C	T	missense_variant
19-13103021-13103022-G-A		1	1	Olfr1458	A94V	1	SYNVCASQM	4	2	72.626	39.72	0.2	0.2	0	0	0	0	0.462	NoExpr	Pending	19	13103021	13103022	13103022	G	A	missense_variant
14-14931283-14931284-G-C		1		Nek10	G752A	1	VYEPVPEAI	8	1	72.654	173.884	0.2	0.4	0	0	0	0	0.259	NoExpr	Pending	14	14931283	14931284	14931284	G	C	missense_variant
2-87213695-87213696-G-A		3		Olfr1113	G268E	1	FYESASITYL	3	3	72.89	86.52	0.2	0.2	0	0	0	0	0.377	NoExpr	Pending	2	87213695	87213696	87213696	G	A	missense_variant
9-38606772-38606773-T-G		2	3	Olfr914	F103V	1	MTQFYVFGF	6	5	73.365	51.895	0.4	0.22	0	0	0	0	0.133	NoExpr	Pending	9	38606772	38606773	38606773	T	G	missense_variant
10-116353855-116353856-G-A		2		Ptprb	A1520T	1	SYLEYRHNTSI	9	2	73.572	207.434	0.2	0.4	1.178	0	0	0	0.047	NoExpr	Pending	10	116353855	116353856	116353856	G	A	missense_variant
X-23958723-23958724-T-C		1		Gm4985	K71E	1	KYINLIAKLEL	10	1	74.11	113.097	0.2	0.29	0	0	0	0	0.467	NoExpr	Pending	X	23958723	23958724	23958724	T	C	missense_variant
11-115205094-115205095-C-T		3	1	Tmem104	T154I	1	IYLYGDLAI	1	3	75.381	86.38	0.2	0.3	2.643	0	0	0	0.319	NoExpr	Pending	11	115205094	115205095	115205095	C	T	missense_variant
9-39819802-39819803-C-T		1	3	Olfr970	L55F	1	HFHTPMYYF	2	4	75.48	94.805	0.4	0.5	0	0	0	0	0.213	NoExpr	Pending	9	39819802	39819803	39819803	C	T	missense_variant
X-100594004-100594005-G-A		2		P2ry4	H96Y	1	YYYAARNYW	8	2	75.525	77.999	0.2	0.3	0	0	0	0	0.051	NoExpr	Pending	X	100594004	100594005	100594005	G	A	missense_variant
7-10087866-10087867-C-T		2	1	Vmn2r51	M630I	1	SYLLLISLM	6	3	75.554	60.431	0.25	0.2	0	0	0	0	0.027	NoExpr	Pending	7	10087866	10087867	10087867	C	T	missense_variant
2-88446656-88446657-C-T		1	1	Olfr1182	D94N	1	NCMLQVFAM	1	2	75.683	112.561	0.4	0.7	0	0	0	0	0.023	NoExpr	Pending	2	88446656	88446657	88446657	C	T	missense_variant
1-173926891-173926892-C-T		1		Ifi203	E758K	1	SNTLSKSAI	6	1	76.048	93.56	0.4	0.4	138.65	0	0	0	0.226	NoExpr	Pending	1	173926891	173926892	173926892	C	T	missense_variant
12-115119784-115119785-A-G		1		Ighv1-50	L105P	1	AYMQLSSPT	8	1	76.606	123.905	0.2	0.4	0	0	0	0	0.051	NoExpr	Pending	12	115119784	115119785	115119785	A	G	missense_variant
12-115623197-115623198-A-G		1		Ighv1-69	L105P	1	AYMQLSSPT	8	1	76.606	123.905	0.2	0.4	0	0	0	0	0.01	NoExpr	Pending	12	115623197	115623198	115623198	A	G	missense_variant
7-108211730-108211731-T-G		3		Olfr487	K266T	1	IYVMPTSI	6	3	77.85	377.99	0.2	0.29	0	0	0	0	0.026	NoExpr	Pending	7	108211730	108211731	108211731	T	G	missense_variant
4-86583171-86583172-C-T		2		Haus6	A821T	1	SYETLKKSL	4	2	78.21	44.082	0.3	0.2	7.994	0	0	0	0.582	NoExpr	Pending	4	86583171	86583172	86583172	C	T	missense_variant
2-87358133-87358134-C-T		2		Olfr1120	T230I	1	SYSKIIAII	8	2	78.933	37.919	0.3	0.2	0	0	0	0	0.542	NoExpr	Pending	2	87358133	87358134	87358134	C	T	missense_variant
2-87846960-87846961-C-T		2		Olfr1150-ps1	T230I	1	SYSKIIAII	8	2	78.933	37.919	0.3	0.2	0	0	0	0	0.393	NoExpr	Pending	2	87846960	87846961	87846961	C	T	missense_variant
1-32414963-32414964-G-C		1		Khdrbs2	A144P	1	PYSRMSHAL	1	1	79.298	18.124	0.3	0.2	0	0	0	0	0.128	NoExpr	Pending	1	32414963	32414964	32414964	G	C	missense_variant
7-3717393-3717394-C-A		4		Pirb	V327L	1	YYEPRLSLL	8	4	79.816	51.179	0.3	0.2	5.132	0	0	0	0.025	NoExpr	Pending	7	3717393	3717394	3717394	C	A	missense_variant
10-84591235-84591236-A-C		3		Tcp11l2	K188T	1	NYVITTMGTI	9	3	79.95	445.8	0.2	0.61	6.259	0	0	0	0.251	NoExpr	Pending	10	84591235	84591236	84591236	A	C	missense_variant
11-103631333-103631334-G-C		1		Lyzl6	C145W	1	SYWMTGWHL	7	1	79.999	115.571	0.3	0.4	0	0	0	0	0.25	NoExpr	Pending	11	103631333	103631334	103631334	G	C	missense_variant
12-86058952-86058953-A-C		2		Tgfb3	F343V	1	YYANVCSGP	5	2	80.555	556.019	0.4	1.8	7.734	0	0	0	0.318	NoExpr	Pending	12	86058952	86058953	86058953	A	C	missense_variant
4-88683332-88683333-C-G		5		Ifna2	R149S	1	KYFHSITVYL	5	5	80.72	266.59	0.2	0.41	0	0	0	0	0.042	NoExpr	Pending	4	88683332	88683333	88683333	C	G	missense_variant
4-88835970-88835971-G-C		5		Ifna5	R149S	1	KYFHSITVYL	5	5	80.72	266.59	0.2	0.41	0	0	0	0	0.023	NoExpr	Pending	4	88835970	88835971	88835971	G	C	missense_variant
4-88591938-88591939-C-G		5	1	Ifna9	R149S	1	KYFHSITVYL	5	6	80.72	266.59	0.2	0.41	0	0	0	0	0.006	NoExpr	Pending	4	88591938	88591939	88591939	C	G	missense_variant
12-113702557-113702558-C-G		1	1	Ighv5-12	L7F	1	MNLGLSFIF	7	2	80.878	2031.56	0.4	5.8	0	0	0	0	0.035	NoExpr	Pending	12	113702557	113702558	113702558	C	G	missense_variant
14-19417287-19417288-G-A		1	2	Gm21738	S80L	1	FLFSMIFSF	2	3	81.283	100.462	0.4	0.4	0	0	0	0	0.026	NoExpr	Pending	14	19417287	19417288	19417288	G	A	missense_variant
17-35266221-35266222-G-A		4		H2-D1	V311I	1	SYMVIIAVL	6	4	81.487	82.431	0.3	0.3	1154.2	0	0	0	0.037	NoExpr	Pending	17	35266221	35266222	35266222	G	A	missense_variant
6-40464143-40464144-T-G		1		Wee2	M461R	1	FYGLLKNRI	8	1	82.241	79.632	0.2	0.2	0	0	0	0	0.177	NoExpr	Pending	6	40464143	40464144	40464144	T	G	missense_variant

4-100441382-100441383-A-T		5		Ror1	K651M	1	YYRVQSMSSL	7	5	83.537	89.14	0.2	0.2	0.037	0	0	0	0.33	NoExpr	Pending	4	100441382	100441383	100441383	A	T	missense_variant
12-40036461-40036463-AC-GT		1		Arl4a	G95D	1	SYTRCTDDI	8	1	83.61	91.09	0.3	0.3	7.724	0	0	0	0.009	NoExpr	Pending	12	40036461	40036463	40036462	AC	GC,GT	missense_variant
2-89283062-89283063-T-G		1	3	Olfr1229	K23N	1	NPNVQNIIF	6	4	84.446	74.97	0.3	0.34	0	0	0	0	0.202	NoExpr	Pending	2	89283062	89283063	89283063	T	G	missense_variant
9-89090620-89090621-T-A		1		Trim43b	K160I	1	NYVEIERRTTL	5	1	85.483	241.32	0.2	0.31	0.024	0	0	0	0.098	NoExpr	Pending	9	89090620	89090621	89090621	T	A	missense_variant
11-58684276-58684277-C-T		1	2	Olfr320	L135F	1	RPLHYPVFM	8	3	86.099	190.108	0.4	0.8	0	0	0	0	0.041	NoExpr	Pending	11	58684276	58684277	58684277	C	T	missense_variant
6-42838383-42838384-T-G		2		Olfr449	F168V	1	IYFISRLSV	9	2	86.43	839.963	0.4	1.3	0	0	0	0	0.245	NoExpr	Pending	6	42838383	42838384	42838384	T	G	missense_variant
10-127658676-127658677-G-A		1		Stat6	M703I	1	SFQSLEESI	9	1	86.714	442.1	0.3	0.8	38.451	0	0	0	0.144	NoExpr	Pending	10	127658676	127658677	127658677	G	A	missense_variant
10-121615051-121615052-C-G		1		Xpot	A148P	1	LYLRILMPI	8	1	88	58.088	0.2	0.2	37.614	0	0	0	0.117	NoExpr	Pending	10	121615051	121615052	121615052	C	G	missense_variant
6-146396964-146396965-G-A		1	2	Itpr2	L366F	1	NDIASFFEL	6	3	89.501	138.302	0.4	0.6	3.618	0	0	0	0.263	NoExpr	Pending	6	146396964	146396965	146396965	G	A	missense_variant
1-58752442-58752443-G-C		1	3	Cflar	A302P	1	MPQHQDYDS	2	4	89.743	1104.08	0.4	3.5	47.479	0	0	0	0.141	NoExpr	Pending	1	58752442	58752443	58752443	G	C	missense_variant
9-109722218-109722219-A-C		3		Fbxw26	F337V	1	GYLTASVSL	7	3	90.11	113.097	0.4	0.6	0	0	0	0	0.292	NoExpr	Pending	9	109722218	109722219	109722219	A	C	missense_variant
6-80021759-80021760-G-A		1		Lrrtm4	A52T	1	VYCESHTFADI	7	1	91.09	148.226	0.2	0.3	0	0	0	0	0.043	NoExpr	Pending	6	80021759	80021760	80021760	G	A	missense_variant
11-71114444-71114445-T-G		2		Nlrp1a	Y648S	1	SYSAVHSLC	3	2	91.22	64.282	0.3	0.22	0	0	0	0	0.098	NoExpr	Pending	11	71114444	71114445	71114445	T	G	missense_variant
7-7312953-7312954-G-C		3		Vmn2r30	L627V	1	SYVLLMSLM	3	3	91.641	60.431	0.3	0.2	0	0	0	0	0.3	NoExpr	Pending	7	7312953	7312954	7312954	G	C	missense_variant
7-49464889-49464890-T-C		1		Nav2	V874A	1	GYMSDGDAL	8	1	93.083	214.93	0.5	0.6	4.528	0	0	0	0.125	NoExpr	Pending	7	49464889	49464890	49464890	T	C	missense_variant
17-23900506-23900509-TAA-CTC	1	3		Dcpp2	YN98-99YS	1	TYSPSALTQI	3	4	93.38	65.44	0.2	0.2	0	0	0	0	0.027	NoExpr	Pending	17	23900506	23900509	23900507	TAA	CTC	missense_variant
10-52101838-52101839-A-T		4		Ros1	L1458H	1	LYATNTSHTL	8	4	93.6	194.09	0.2	0.3	0	0	0	0	0.215	NoExpr	Pending	10	52101838	52101839	52101839	A	T	missense_variant
2-111983699-111983700-C-G		1	2	Olfr1309	A133P	1	RYVPICKPL	4	3	94.095	96.154	0.5	0.5	0	0	0	0	0.272	NoExpr	Pending	2	111983699	111983700	111983700	C	G	missense_variant
9-50913501-50913502-G-C		1	3	Sik2	A328G	1	YNHFAGIYF	6	4	94.095	108.306	0.4	0.6	2.686	0	0	0	0.097	NoExpr	Pending	9	50913501	50913502	50913502	G	C	missense_variant
3-127562336-127562337-A-G		1		Zgrf1	N404S	1	SFCKSSSVL	5	1	94.095	242.01	0.3	0.6	4.147	0	0	0	0.044	NoExpr	Pending	3	127562336	127562337	127562337	A	G	missense_variant
6-57002713-57002714-A-C		2	1	Vmn1r6	K120N	1	LNNYMIYAF	2	3	94.189	202.768	0.5	0.9	0	0	0	0	0.209	NoExpr	Pending	6	57002713	57002714	57002714	A	C	missense_variant
9-3450127-3450128-C-T		1	2	Cwf19l2	L610F	1	DINQNRFFM	7	3	95.94	2558.59	0.5	4.4	6.845	0	0	0	0.512	NoExpr	Pending	9	3450127	3450128	3450128	C	T	missense_variant
17-20114053-20114054-G-A		2		Fpr-rs7	T58I	1	IYVAGFRMTHI	11	2	97.6	1179.53	0.2	2.3	0	0	0	0	0.051	NoExpr	Pending	17	20114053	20114054	20114054	G	A	missense_variant
2-86988394-86988395-A-C		2		Olfr1101	S260R	1	MYMRPRSSYTI	6	2	98.43	93.15	0.2	0.2	0	0	0	0	0.179	NoExpr	Pending	2	86988394	86988395	86988395	A	C	missense_variant
18-49723694-49723695-A-C		1	1	Dtwd2	I175S	1	VYPSTISLI	7	2	98.46	676.488	0.3	2.2	4.456	0	0	0	0.139	NoExpr	Pending	18	49723694	49723695	49723695	A	C	missense_variant
9-66462954-66462955-G-A		2		Herc1	G3008R	1	RYRSNGSYV	1	2	98.59	115.571	0.4	0.6	5.163	0	0	0	0.583	NoExpr	Pending	9	66462954	66462955	66462955	G	A	missense_variant
12-115861900-115861901-G-T		1		Ighv1-77	T106K	1	AYMQLSSLK	9	1	98.648	123.905	0.4	0.4	0	0	0	0	0.071	NoExpr	Pending	12	115861900	115861901	115861901	G	T	missense_variant
5-14676235-14676236-C-T		1		Pclo	P1703S	1	SHGPLLSTI	7	1	98.876	659.754	0.3	2.1	0	0	0	0	0.419	NoExpr	Pending	5	14676235	14676236	14676236	C	T	missense_variant
3-113629900-113629901-C-A		4		Rnpc3	A52S	1	KYFGSQSVR	5	4	98.915	172.687	0.3	0.5	8.703	0	0	0	0.25	NoExpr	Pending	3	113629900	113629901	113629901	C	A	missense_variant
X-102520774-102520775-T-G		3		Phka1	K1092N	1	FYQNVWKIL	4	3	99.86	61.389	0.3	0.3	2.818	0	0	0	0.212	NoExpr	Pending	X	102520774	102520775	102520775	T	G	missense_variant
9-44818767-44818768-C-T		2		Kmt2a	M3414I	1	AAITAASSI	3	2	100.48	127.391	0.4	0.3	2.708	0	0	0	0.236	NoExpr	Pending	9	44818767	44818768	44818768	C	T	missense_variant
6-57978920-57978921-A-C		3	1	Vmn1r25	F128V	1	VVYIWFYNF	2	4	101.5	184.036	0.5	0.8	0	0	0	0	0.022	NoExpr	Pending	6	57978920	57978921	57978921	A	C	missense_variant
10-94579064-94579065-G-A		2		Tmcc3	G241R	1	AYGRSATIV	4	2	101.63	197.738	0.4	0.4	2.861	0	0	0	0.188	NoExpr	Pending	10	94579064	94579065	94579065	G	A	missense_variant
8-20944652-20944653-C-T		2		AY761185	D20N	1	FYVQANSTQ	6	2	101.88	107.421	0.3	0.4	0	0	0	0	0.549	NoExpr	Pending	8	20944652	20944653	20944653	C	T	missense_variant
1-85610757-85610759-AG-GC		3		Sp140	K72S	1	EYQETCSNL	7	3	101.92	748.618	0.3	1.2	54.864	0	0	0	0.011	NoExpr	Pending	1	85610757	85610759	85610758	AG	GC,GG	missense_variant
3-116582634-116582635-C-G		5		Trmt13	S369T	1	YYFQRMSTW	8	5	102.12	103.774	0.3	0.34	8.563	0	0	0	0.025	NoExpr	Pending	3	116582634	116582635	116582635	C	G	missense_variant
12-115193725-115193726-C-A		2		Ighv1-54	M100I	1	AYIQLSSLT	3	2	102.35	123.905	0.3	0.4	0	0	0	0	0.002	NoExpr	Pending	12	115193725	115193726	115193726	C	A	missense_variant
12-115242852-115242853-C-A		2		Ighv1-56	M100I	1	AYIQLSSLT	3	2	102.35	123.905	0.3	0.4	0	0	0	0	0.019	NoExpr	Pending	12	115242852	115242853	115242853	C	A	missense_variant
12-115861917-115861918-C-A		2		Ighv1-77	M100I	1	AYIQLSSLT	3	2	102.35	123.905	0.3	0.4	0	0	0	0	0.065	NoExpr	Pending	12	115861917	115861918	115861918	C	A	missense_variant
11-71109048-71109049-G-A		1	2	Nlrp1a	P762S	1	KPHVMVSTM	7	2	102.6	27.102	0.4	0.2	0	0	0	0	0.023	NoExpr	Pending	11	71109048	71109049	71109049	G	A	missense_variant
7-85624374-85624375-G-A		1		Vmn2r71	S799N	1	VYHNTKGKI	4	1	103.58	77.137	0.3	0.3	0	0	0	0	0.024	NoExpr	Pending	7	85624374	85624375	85624375	G	A	missense_variant
2-87489670-87489671-A-C		1		Pramel7	I426R	1	NYVRVEIFI	4	1	103.77	242.153	0.3	0.5	0	0	0	0	0.226	NoExpr	Pending	2	87489670	87489671	87489671	A	C	missense_variant
17-22572068-22572070-TT-AC		1	2	Vmn2r111	IN82-83MY	1	YALALAFSM	9	3	103.95	348.201	0.4	0.8	0	0	0	0	0.03	NoExpr	Pending	17	22572068	22572070	22572069	TT	AC	missense_variant
12-113716789-113716791-GT-TA		3		Ighv2-6	T77Y	1	TYYNSALKSRL	2	3	104.35	976.967	0.2	2.2	0	0	0	0	0.029	NoExpr	Pending	12	113716789	113716791	113716790	GT	TA,TC	missense_variant
15-41096161-41096162-G-A		2		Zfpm2	M212I	1	SRLQAASHI	9	2	104.49	396.36	0.3	1.2	13.804	0	0	0	0.146	NoExpr	Pending	15	41096161	41096162	41096162	G	A	missense_variant
7-140336052-140336053-T-G		2		Olfr527	L64V	1	YFFLVNVAI	7	2	105.02	105.017	0.4	0.5	0	0	0	0	0.171	NoExpr	Pending	7	140336052	140336053	140336053	T	G	missense_variant
11-71181766-71181767-C-G		1	1	Nlrp1b	A417P	1	QPSSLSYSF	2	2	105.2	1672.09	0.4	4.1	2.122	0	0	0	0.016	NoExpr	Pending	11	71181766	71181767	71181767	C	G	missense_variant
11-36047471-36047472-A-T		2		Tenm2	L1458H	1	DYSLSKHAI	7	2	105.5	246.752	0.3	0.4	0	0	0	0	0.263	NoExpr	Pending	11	36047471	36047472	36047472	A	T	missense_variant
16-59216159-59216160-A-C		2		Olfr199	L151R	1	AYLRGALHL	4	2	105.7	170.612	0.3	0.6	0	0	0	0	0.231	NoExpr	Pending	16	59216159	59216160	59216160	A	C	missense_variant
2-76789486-76789487-C-T		1		Ttn	V14200I	1	FRIAAENAI	3	1	106.23	515.537	0.3	1	0.056	0	0	0	0.4	NoExpr	Pending	2	76789486	76789487	76789487	C	T	missense_variant
9-79853746-79853747-C-G		2		Filip1	Q168H	1	TYRRMLEHL	8	2	107.14	101.99	0.5	0.5	0.665	0	0	0	0.077	NoExpr	Pending	9	79853746	79853747	79853747	C	G	missense_variant
11-60762537-60762538-C-T		3		Top3a	V82I	1	LYGQNITMI	6	3	107.71	113.308	0.3	0.3	3.22	0	0	0	0.57	NoExpr	Pending	11	60762537	60762538	60762538	C	T	missense_variant
11-74206597-74206598-A-G		1		Olfr43	F206S	1	MYLGVGVSSV	8	1	108.01	251.868	0.1	0.27	0	0	0	0	0.21	NoExpr	Pending	11	74206597	74206598	74206598	A	G	missense_variant
2-98662320-98662321-A-C		3	1	Gm10801	M29L	1	FSFSLIFSF	5	4	108.85	100.462	0.5	0.4	0.12	0	0	0	0.014	NoExpr	Pending	2	98662320	98662321	98662321	A	C	missense_variant
13-68689306-68689307-A-T		2	1	Adcy2	L641H	1	AFHSLIFIL	3	3	108.89	270.29	0.5	1.1	0.026	0	0	0	0.189	NoExpr	Pending	13	68689306	68689307	68689307	A	T	missense_variant
10-75638159-75638160-G-A	2	1		Susd2	H654Y	1	KYNPNFKPL	2	3	109.39	1815.73	0.5	6.8	0.78	0	0	0	0.246	NoExpr	Pending	10	75638159	75638160	75638160	G	A	missense_variant
2-87833826-87833827-G-C		3		Olfr1148	V263L	1	FYGSATLTYL	7	3	109.47	113.49	0.2	0.2	0	0	0	0	0.211	NoExpr	Pending	2	87833826	87833827	87833827	G	C	missense_variant
5-61809303-61809304-G-T		2		G6pd2	A141S	1	FYLSLPPTV	4	2	109.49	101.499	0.5	0.4	0.025	0	0	0	0.137	NoExpr	Pending	5	61809303	61809304	61809304	G	T	missense_variant
2-129208196-129208197-C-T		3		Slc20a1	T425I	1	SYTSYIMAI	6	3	110.68	97.1	0.3	0.3	74.495	0	0	0	0.429	NoExpr	Pending	2	129208196	129208197	129208197	C	T	missense_variant
8-69707007-69707008-C-A		2		Zfp869	R305M	1	KFFRSHMTL	7	2	110.68	103.3	0.3	0.3	12.8	0	0	0	0.222	NoExpr	Pending	8	69707007	69707008	69707008	C	A	missense_variant
17-35380616-35380617-G-T		2		H2-Q4	K225N	1	RYLELGNETLL	7	2	111.1	143.492	0.2	0.2	130.46	0	0	0	0.069	NoExpr	Pending	17	35380616	35380617	35380617	G	T	missense_variant
18-84013927-84013928-T-C		2		Tshz1	N785S	1	LYKISSSML	6	2	111.5	125.05	0.3	0.3	11.33	0	0	0	0.308	NoExpr	Pending	18	84013927	84013928	84013928	T	C	missense_variant
16-30308613-30308614-G-A		1	2	Gp5	T414I	1	LPQLIQVLL	5	3	111.64	149.624	0.5	0.7	0.085	0	0	0	0.31	NoExpr	Pending	16	30308613	30308614	30308614	G	A	missense_variant
2-98662306-98662307-G-A		2		Gm10801	C24Y	1	RYFSFSMI	2	2	111.82	6258.35	0.2	9	0.12	0	0	0	0.028	NoExpr	Pending	2	98662306	98662307	98662307	G	A	missense_variant
1-59199148-59199149-G-T		4		Als2	L675I	1	SYIALVDKNI	3	4	111.89	137.49	0.2	0.2	5.015	0	0	0	0.206	NoExpr	Pending	1	59199148	59199149	59199149	G	T	missense_variant

12-59160181-59160182-G-A		3		Mia2	A317T	1	IYAAKLNTSL	8	3	111.91	869.49	0.2	1.2	25.177	0	0	0	0.426	NoExpr	Pending	12	59160181	59160182	59160182	G	A	missense_variant
4-111982118-111982119-G-T		3		Skint7	M203I	1	FYMKITLLL	5	3	112.25	33.78	0.4	0.2	0	0	0	0	0.025	NoExpr	Pending	4	111982118	111982119	111982119	G	T	missense_variant
2-87252095-87252096-T-C		3		Olfr1115	I53T	1	FFIYMSTLL	7	3	112.76	597.159	0.2	1.5	0	0	0	0	0.089	NoExpr	Pending	2	87252095	87252096	87252096	T	C	missense_variant
2-86508768-86508769-C-A		2	1	Olfr1076	F103L	1	AVQLSLFSM	6	3	113.76	77.983	0.5	0.4	0	0	0	0	0.029	NoExpr	Pending	2	86508768	86508769	86508769	C	A	missense_variant
11-71181702-71181703-C-T		4		Nlrp1b	C438Y	1	EFFASMSYI	8	4	113.78	222.377	0.2	0.6	2.122	0	0	0	0.054	NoExpr	Pending	11	71181702	71181703	71181703	C	T	missense_variant
2-129037998-129037999-G-A		3	1	Vinac1	S669F	1	LPVEQLFYT	7	4	114.03	3863.67	0.5	7.4	0	0	0	0	0.136	NoExpr	Pending	2	129037998	129037999	129037999	G	A	missense_variant
3-154256586-154256587-G-A		3		Slc44a5	A386T	1	SYWAVTTVF	7	3	114.58	776.712	0.3	1.4	0.008	0	0	0	0.021	NoExpr	Pending	3	154256586	154256587	154256587	G	A	missense_variant
6-40571579-40571580-C-T		2	2	Olfr460	L65F	1	YFFLVHFSI	7	3	115.57	114.62	0.59	0.5	0	0	0	0	0.845	NoExpr	Pending	6	40571579	40571580	40571580	C	T	missense_variant
2-98662318-98662319-C-T		2	3	Gm10801	S28F	1	FSFFMIFSF	4	5	115.61	100.462	0.5	0.4	0.12	0	0	0	0.044	NoExpr	Pending	2	98662318	98662319	98662319	C	T	missense_variant
7-10274335-10274336-A-T		2		Vmn1r66	L257I	1	AFYTLSSII	9	2	115.9	291.66	0.3	0.6	0	0	0	0	0.211	NoExpr	Pending	7	10274335	10274336	10274336	A	T	missense_variant
12-114479302-114479303-C-T		2		Ighv10-1	V21M	1	FYQGVHCEM	9	2	116.17	133.77	0.2	0.3	0	0	0	0	0.154	NoExpr	Pending	12	114479302	114479303	114479303	C	T	missense_variant
7-26611642-26611643-G-C		1	1	Vmn1r185	L146V	1	LHMVMHFII	4	1	117.22	138.357	0.6	0.6	0	0	0	0	0.066	NoExpr	Pending	7	26611642	26611643	26611643	G	C	missense_variant
10-116314083-116314084-G-C		3		Ptprb	G200A	1	VYINASTV	5	3	117.49	149.93	0.14	0.21	1.178	0	0	0	0.252	NoExpr	Pending	10	116314083	116314084	116314084	G	C	missense_variant
X-143101994-143101995-G-T		2		Rtl9	R801I	1	SITPVYGTI	2	2	118.1	92.276	0.6	0.4	0	0	0	0	0.75	NoExpr	Pending	X	143101994	143101995	143101995	G	T	missense_variant
5-11773148-11773149-G-A		1		Gm8926	R95K	1	FYEQLKSTK	6	1	118.33	116.974	0.4	0.4	0	0	0	0	0.04	NoExpr	Pending	5	11773148	11773149	11773149	G	A	missense_variant
6-132803745-132803747-GA-TT		2	2	Tas2r117	PT282-283PS	1	FPSFHSYIL	3	4	118.58	169.044	0.2	0.3	0	0	0	0	0.114	NoExpr	Pending	6	132803745	132803747	132803746	GA	TT	missense_variant
12-115145530-115145531-T-A		2		Ighv1-52	S103C	1	AYMQLCSLT	6	2	119.15	123.905	0.3	0.4	0	0	0	0	0.015	NoExpr	Pending	12	115145530	115145531	115145531	T	A	missense_variant
12-115359183-115359184-T-A		2		Ighv1-61	S103C	1	AYMQLCSLT	6	2	119.15	123.905	0.3	0.4	0	0	0	0	0.093	NoExpr	Pending	12	115359183	115359184	115359184	T	A	missense_variant
6-70961016-70961017-G-A		4	2	Foxi3	S385N	1	SPFYNFNM	7	6	120	574.93	0.1	0.1	0	0	0	0	0.07	NoExpr	Pending	6	70961016	70961017	70961017	G	A	missense_variant
15-101528756-101528757-T-C		1		Krt84	T324A	1	SHISEASVI	6	1	120.02	186.78	0.4	0.48	0	0	0	0	0.029	NoExpr	Pending	15	101528756	101528757	101528757	T	C	missense_variant
5-71869471-71869472-A-C		3		Gabrb1	N105T	1	SYSGIPLTL	8	3	120.25	230.192	0.4	0.84	0.004	0	0	0	0.298	NoExpr	Pending	5	71869471	71869472	71869472	A	C	missense_variant
X-138220466-138220467-C-G		2		Il1rapl2	R147G	1	CYNSRIGYL	7	2	120.45	125.49	0.3	0.3	0	0	0	0	0.14	NoExpr	Pending	X	138220466	138220467	138220467	C	G	missense_variant
8-110415777-110415778-A-C		3	1	Hydin	E763A	1	YPYAKTIQL	4	4	120.73	216.686	0.6	0.7	0	0	0	0	0.153	NoExpr	Pending	8	110415777	110415778	110415778	A	C	missense_variant
7-43815408-43815409-A-C		1		Klk7	K236T	1	VYTQVCTYK	7	1	121.98	765.755	0.4	1.4	0.08	0	0	0	0.346	NoExpr	Pending	7	43815408	43815409	43815409	A	C	missense_variant
2-178403761-178403762-C-A		1	1	Sycp2	D22Y	1	NYFKPLLAL	2	2	122	5167.26	0.39	14	0.011	0	0	0	0.312	NoExpr	Pending	2	178403761	178403762	178403762	C	A	missense_variant
10-130210776-130210777-G-A		1	2	Olfr827	L118F	1	QLFLYAFFM	7	3	123.32	3000.81	0.59	4.8	0	0	0	0	0.632	NoExpr	Pending	10	130210776	130210777	130210777	G	A	missense_variant
12-115193709-115193710-T-A		1		Ighv1-54	T106S	1	AYMQLSSLS	9	1	123.34	123.905	0.4	0.4	0	0	0	0	0.003	NoExpr	Pending	12	115193709	115193710	115193710	T	A	missense_variant
12-115242836-115242837-T-A		1		Ighv1-56	T106S	1	AYMQLSSLS	9	1	123.34	123.905	0.4	0.4	0	0	0	0	0.019	NoExpr	Pending	12	115242836	115242837	115242837	T	A	missense_variant
12-115861901-115861902-T-A		1		Ighv1-77	T106S	1	AYMQLSSLS	9	1	123.34	123.905	0.4	0.4	0	0	0	0	0.056	NoExpr	Pending	12	115861901	115861902	115861902	T	A	missense_variant
9-18856953-18856954-G-A		1		Olfr829	V110I	1	TYGGCLTQI	9	1	123.63	423.79	0.4	1.4	0	0	0	0	0.506	NoExpr	Pending	9	18856953	18856954	18856954	G	A	missense_variant
7-101504601-101504602-G-C		1		Gm45837	G485A	1	IRIPADQAI	8	1	123.67	295.978	0.4	0.8	4.217	0	0	0	0.379	NoExpr	Pending	7	101504601	101504602	101504602	G	C	missense_variant
1-85610789-85610790-G-T		1		Sp140	D83Y	1	YYILSNVQK	2	1	124.24	5435.76	0.5	18	54.864	0	0	0	0.009	NoExpr	Pending	1	85610789	85610790	85610790	G	T	missense_variant
5-86906598-86906599-A-C		2	3	Ugt2b34	F108V	1	LPKQSVWGYFL	6	5	124.63	77.16	0.17	0.14	0	0	0	0	0.047	NoExpr	Pending	5	86906598	86906599	86906599	A	C	missense_variant
12-91825362-91825363-C-T		2		Sel1l	A299T	1	RYWTGIGVL	4	2	124.87	187.107	0.4	0.4	19.425	0	0	0	0.423	NoExpr	Pending	12	91825362	91825363	91825363	C	T	missense_variant
9-39184494-39184495-T-C		1	5	Olfr943	F106L	1	GCMAQLYLF	8	5	125.27	44.857	0.6	0.2	0	0	0	0	0.269	NoExpr	Pending	9	39184494	39184495	39184495	T	C	missense_variant
13-49068232-49068233-T-C		1	1	Wnk2	D1184G	1	TYMVEHGFI	7	2	125.97	165.21	0.23	0.3	0	0	0	0	0.058	NoExpr	Pending	13	49068232	49068233	49068233	T	C	missense_variant
14-60027953-60027954-T-G		1		Atp8a2	K311T	1	WYIKTMDTN	5	1	127.43	1120.11	0.4	2.1	0	0	0	0	0.307	NoExpr	Pending	14	60027953	60027954	60027954	T	G	missense_variant
14-50736516-50736517-C-T		3		Olfr749	R215Q	1	AYILQSYI	5	3	128.95	50.88	0.18	0.1	0	0	0	0	0.026	NoExpr	Pending	14	50736516	50736517	50736517	C	T	missense_variant
12-40036678-40036679-A-G		1	1	Arl4a	I23T	1	SFQSFHTVI	7	2	129.21	1054.61	0.4	2.5	7.724	0	0	0	0.026	NoExpr	Pending	12	40036678	40036679	40036679	A	G	missense_variant
12-115567247-115567248-G-C		5		Ighv8-11	R87G	1	KYYNTALKSGL	10	5	129.45	80.856	0.2	0.2	0	0	0	0	0.11	NoExpr	Pending	12	115567247	115567248	115567248	G	C	missense_variant
7-23984770-23984772-AT-CC		1	1	Vmn1r181	IF220-221IL	1	HRQRMQYIL	9	2	130.02	92.683	0.6	0.5	0	0	0	0	0.047	NoExpr	Pending	7	23984770	23984772	23984771	AT	CC	missense_variant
17-35471076-35471077-G-C		2		H2-Q10	G199R	1	RYLELRKETLL	6	2	130.18	143.492	0.2	0.2	5.017	0	0	0	0.025	NoExpr	Pending	17	35471076	35471077	35471077	G	C	missense_variant
6-57956170-57956171-T-G		2	1	Vmn1r24	K121Q	1	LKQYIIYAF	3	3	131.59	613.762	0.8	2.5	0	0	0	0	0.429	NoExpr	Pending	6	57956170	57956171	57956171	T	G	missense_variant
12-98815984-98815985-T-G		2		Eml5	D1396A	1	HYLNDGDAI	8	2	131.72	437.17	0.3	0.61	1.907	0	0	0	0.275	NoExpr	Pending	12	98815984	98815985	98815985	T	G	missense_variant
2-87693491-87693492-A-T		1	1	Olfr1136	L130H	1	NPHMYAVDM	3	2	131.83	265.461	0.3	1.1	0	0	0	0	0.275	NoExpr	Pending	2	87693491	87693492	87693492	A	T	missense_variant
7-42011872-42011873-C-A		2		Vmn2r59	R839S	1	CYIILLSPE	7	2	131.85	472.413	0.4	1.3	0	0	0	0	0.147	NoExpr	Pending	7	42011872	42011873	42011873	C	A	missense_variant
4-156334442-156334443-C-T		1		Vmn2r-ps159	A340V	1	KFMQTMNTV	9	1	133.03	762.237	0.4	2.3	0	0	0	0	0.03	NoExpr	Pending	4	156334442	156334443	156334443	C	T	missense_variant
14-53363953-53363954-G-A		2		Trav13n-4	G60E	1	FYQRPEGRL	6	2	133.85	123.42	0.4	0.4	0	0	0	0	0.063	NoExpr	Pending	14	53363953	53363954	53363954	G	A	missense_variant
11-58551585-58551586-A-T		4		Olfr328	S218T	1	SYTSILLTV	3	4	135.94	166.96	0.5	0.7	0	0	0	0	0.057	NoExpr	Pending	11	58551585	58551586	58551586	A	T	missense_variant
7-7384459-7384460-A-T		3	1	Vmn2r31	I704K	1	NYIIPKCSL	6	4	139.4	185.038	0.33	0.4	0	0	0	0	0.055	NoExpr	Pending	7	7384459	7384460	7384460	A	T	missense_variant
14-37092018-37092019-G-A		1		Cdhr1	A150V	1	ENIPVGSSI	5	1	139.99	274.322	0.4	0.8	0	0	0	0	0.883	NoExpr	Pending	14	37092018	37092019	37092019	G	A	missense_variant
17-35321045-35321046-A-G		1		H2-Q1	E97G	1	RFQGSLSTL	4	1	140.39	108.77	0.3	0.3	6.324	0	0	0	0.022	NoExpr	Pending	17	35321045	35321046	35321046	A	G	missense_variant
14-50298648-50298649-A-C		1		Olfr733	V220G	1	SYAGILCHV	4	1	140.42	158.168	0.6	0.6	0	0	0	0	0.294	NoExpr	Pending	14	50298648	50298649	50298649	A	C	missense_variant
1-188415854-188415855-C-T		1		Ush2a	T993I	1	SYFRFDPLI	9	1	140.42	2178.16	0.6	9.2	0	0	0	0	0.649	NoExpr	Pending	1	188415854	188415855	188415855	C	T	missense_variant
14-34343681-34343682-G-A		1		Glud1	V546I	1	AYVNAIEKI	9	1	141.15	398.24	0.4	1.4	192.83	0	0	0	0.85	NoExpr	Pending	14	34343681	34343682	34343682	G	A	missense_variant
10-81362244-81362245-G-C		1	3	Mfsd12	G320A	1	SAFFSSFLM	2	4	143.88	163.305	0.5	0.5	6.792	0	0	0	0.101	NoExpr	Pending	10	81362244	81362245	81362245	G	C	missense_variant
7-7312827-7312828-C-T		1		Vmn2r30	V669I	1	VFTVAVSTI	9	1	144.3	869.7	0.4	1.3	0	0	0	0	0.353	NoExpr	Pending	7	7312827	7312828	7312828	C	T	missense_variant
17-35471049-35471050-G-C		1		H2-Q10	E190Q	1	AYLEAECVQWL	9	1	145.05	271.686	0.3	0.6	5.017	0	0	0	0.035	NoExpr	Pending	17	35471049	35471050	35471050	G	C	missense_variant
2-85976087-85976088-A-C		2	1	Olfr1029	S282R	1	RPLLNPLIYSL	1	3	145.52	158.68	0.1	0.2	0	0	0	0	0.254	NoExpr	Pending	2	85976087	85976088	85976088	A	C	missense_variant
2-88682892-88682893-G-T		1	2	Olfr1195	L280I	1	FINPLIYTL	2	3	145.88	220.206	0.7	0.8	0	0	0	0	0.158	NoExpr	Pending	2	88682892	88682893	88682893	G	T	missense_variant
17-37624365-37624366-C-T		3		Olfr116	G90S	1	SSLAGSSYI	7	3	145.91	388.269	0.39	1.2	0	0	0	0	0.021	NoExpr	Pending	17	37624365	37624366	37624366	C	T	missense_variant
7-21350126-21350127-T-C		2	1	Vmn1r128	F252S	1	SYLLNCICI	1	3	146.63	363.153	0.6	0.7	0	0	0	0	0.125	NoExpr	Pending	7	21350126	21350127	21350127	T	C	missense_variant
2-88412285-88412286-G-A		2		Olfr1180	P124L	1	RYVAICRLL	8	2	148	59.09	0.5	0.4	0	0	0	0	0.478	NoExpr	Pending	2	88412285	88412286	88412286	G	A	missense_variant
7-103565110-103565111-C-A		1	1	Olfr616	S56I	1	ILHEPQYIF	1	2	148.23	138.909	0.8	0.8	0	0	0	0	0.288	NoExpr	Pending	7	103565110	103565111	103565111	C	A	missense_variant
X-74303872-74303874-GG-TT	1	1	2	Atp6ap1	W420F	1	IFMGLLTTL	2	4	148.62	239.04	0.4	0.5	115.79	0	0	0	0.043	NoExpr	Pending	X	74303872	74303874	74303873	GG	TT	missense_variant

5-32739345-32739347-GC-AA		2		Gm20671	TR283-284TC	1	LYKSVPTCL	8	2	148.68	105.502	0.4	0.3	0	0	0	0	0.025	NoExpr	Pending	5	32739345	32739347	32739346	GC	AA	missense_variant
19-11472482-11472483-T-A		4	2	Ms4a6c	S80T	1	YFNSVFTVL	7	6	149.03	95.556	0.4	0.3	26.147	0	0	0	0.039	NoExpr	Pending	19	11472482	11472483	11472483	T	A	missense_variant
6-65704293-65704294-A-C		2		Ndnf	H519P	1	KYFPSQNLQ	4	2	150.06	286.589	0.4	0.8	0.119	0	0	0	0.036	NoExpr	Pending	6	65704293	65704294	65704294	A	C	missense_variant
5-103529659-103529660-T-C		1	2	Ptpn13	S715P	1	PYFRLEHYL	1	3	151.04	18.96	0.4	0.2	6.968	0	0	0	0.244	NoExpr	Pending	5	103529659	103529660	103529660	T	C	missense_variant
17-35264086-35264088-AA-CT		3		H2-D1	N198L	1	RYLKLGNATL	5	3	152.21	310.83	0.21	0.47	1154.2	0	0	0	0.013	NoExpr	Pending	17	35264086	35264088	35264087	AA	CT	missense_variant
12-115847938-115847939-G-A		2		Ighv1-76	A98V	1	VYMQLSSL	1	2	152.31	100.95	0.22	0.14	0	0	0	0	0.038	NoExpr	Pending	12	115847938	115847939	115847939	G	A	missense_variant
12-115861924-115861925-G-A		2		Ighv1-77	A98V	1	VYMQLSSL	1	2	152.31	100.95	0.22	0.14	0	0	0	0	0.009	NoExpr	Pending	12	115861924	115861925	115861925	G	A	missense_variant
7-14491915-14491917-CC-GT		3	1	Sult2a7	W33Y	1	SYPKSGMTYL	9	4	153.12	276.91	0.3	0.5	0	0	0	0	0.072	NoExpr	Pending	7	14491915	14491917	14491916	CC	GT	missense_variant
4-147613774-147613775-G-A		1		Zfp979	T159I	1	NRLKVSSII	8	1	153.14	62.964	0.6	0.3	16	0	0	0	0.39	NoExpr	Pending	4	147613774	147613775	147613775	G	A	missense_variant
5-146526216-146526217-G-A		3	1	Gm3404	V70M	1	EYMVQMIHYI	3	4	154	427.54	0.2	0.45	0	0	0	0	0.069	NoExpr	Pending	5	146526216	146526217	146526217	G	A	missense_variant
5-146491780-146491781-G-A		3	1	Gm6370	V70M	1	EYMVQMIHYI	3	4	154	427.54	0.2	0.45	0	0	0	0	0.058	NoExpr	Pending	5	146491780	146491781	146491781	G	A	missense_variant
8-15028516-15028517-G-C		1		Kbtbd11	G372A	1	NYLFLAGAV	8	1	154.62	436.606	0.4	1.1	0.368	0	0	0	0.182	NoExpr	Pending	8	15028516	15028517	15028517	G	C	missense_variant
8-93076192-93076193-C-T		2		Ces1b	D90N	1	SYPPMCSQNAV	9	2	154.78	196.38	0.3	0.4	0	0	0	0	0.279	NoExpr	Pending	8	93076192	93076193	93076193	C	T	missense_variant
4-147390493-147390494-C-T		1		Zfp978	T166I	1	SIISLNQGI	9	1	154.78	2169.05	0.73	10	3.726	0	0	0	0.035	NoExpr	Pending	4	147390493	147390494	147390494	C	T	missense_variant
4-146466247-146466248-C-T		2		Zfp992	T142I	1	SIISLNQGI	2	2	154.78	254.608	0.73	0.79	0.548	0	0	0	0.464	NoExpr	Pending	4	146466247	146466248	146466248	C	T	missense_variant
7-108423047-108423048-C-G		2	2	Olfr497	A159G	1	GSLIMFYFF	1	4	155.54	219.195	0.7	0.9	0	0	0	0	0.043	NoExpr	Pending	7	108423047	108423048	108423048	C	G	missense_variant
11-58529905-58529906-G-A		2		Olfr330	H27Y	1	KYSALLAVV	2	2	156.47	2496.64	0.58	6.4	0	0	0	0	0.017	NoExpr	Pending	11	58529905	58529906	58529906	G	A	missense_variant
17-35380605-35380606-G-A		2		H2-Q4	E222K	1	RYLKLGKETL	4	2	156.95	272.88	0.23	0.3	130.46	0	0	0	0.059	NoExpr	Pending	17	35380605	35380606	35380606	G	A	missense_variant
12-37108954-37108955-C-T		3		Meox2	S42F	1	LFTSSSSCI	2	3	157.43	719.598	0.4	2.2	1.05	0	0	0	0.395	NoExpr	Pending	12	37108954	37108955	37108955	C	T	missense_variant
3-102898878-102898879-T-G		1		Sycp1	M499L	1	HYLKQVEEL	9	1	157.78	279.12	0.4	0.48	0.132	0	0	0	0.193	NoExpr	Pending	3	102898878	102898879	102898879	T	G	missense_variant
2-5891296-5891297-C-T		1	2	Sec61a2	C45Y	1	YCQIPLFGI	1	3	158.13	325.837	0.9	1.5	4.789	0	0	0	0.445	NoExpr	Pending	2	5891296	5891297	5891297	C	T	missense_variant
9-109326626-109326627-C-T		2	1	Fbxw28	C359Y	1	IVYSTRFSL	3	3	158.17	194.267	0.7	0.92	0.009	0	0	0	0.163	NoExpr	Pending	9	109326626	109326627	109326627	C	T	missense_variant
7-38099525-38099526-T-G		2		Ccne1	D259A	1	AYVNATGEV	5	2	160.36	1365.99	0.5	2.5	15.885	0	0	0	0.101	NoExpr	Pending	7	38099525	38099526	38099526	T	G	missense_variant
3-14481405-14481406-C-T		1		Slc7a12	L204F	1	IFQVSYSYL	2	1	161.47	565.054	0.4	1.2	0	0	0	0	0.046	NoExpr	Pending	3	14481405	14481406	14481406	C	T	missense_variant
12-115604157-115604158-C-T		1	1	Ighv1-67	G45S	1	SYTFTDYAM	1	2	162.96	567.884	0.5	1.6	0	0	0	0	0.039	NoExpr	Pending	12	115604157	115604158	115604158	C	T	missense_variant
17-36030681-36030682-G-T		1		H2-T23	S299Y	1	PYTVSNMVI	2	1	165.23	6872.11	0.32	16	36.919	0	0	0	0.036	NoExpr	Pending	17	36030681	36030682	36030682	G	T	missense_variant
16-58872883-58872885-CT-TC		1		Olfr177	VD88-89VN	1	NFFSVNRRI	6	1	166.38	205.712	0.5	0.63	0	0	0	0	0.03	NoExpr	Pending	16	58872883	58872885	58872884	CT	TC	missense_variant
16-58916374-58916376-CT-TC		1		Olfr180	VD88-89VN	1	NFFSVNRRI	6	1	166.38	205.712	0.5	0.63	0	0	0	0	0.012	NoExpr	Pending	16	58916374	58916376	58916375	CT	TC	missense_variant
9-59827201-59827202-C-T		1	1	Myo9a	S689F	1	SRNAFVFGM	7	2	166.96	4989.87	0.9	9.5	5.329	0	0	0	0.05	NoExpr	Pending	9	59827201	59827202	59827202	C	T	missense_variant
10-22371417-22371418-C-T		1	2	Raet1d	T131I	1	IYPQSQGQI	9	3	168.69	2767.52	0.4	7.1	0.357	0	0	0	0.336	NoExpr	Pending	10	22371417	22371418	22371418	C	T	missense_variant
10-22181175-22181176-C-T		1	2	Raet1e	T133I	1	IYPQSQGQI	9	3	168.69	2767.52	0.4	7.1	0.469	0	0	0	0.162	NoExpr	Pending	10	22181175	22181176	22181176	C	T	missense_variant
5-146502908-146502909-C-T		3		4930449I24Rik	A100V	1	TYVSTWEVL	3	3	169.46	116.84	0.5	0.3	0	0	0	0	0.1	NoExpr	Pending	5	146502908	146502909	146502909	C	T	missense_variant
2-131561347-131561348-A-G		2		Adra1d	V274A	1	VYVAARSTT	4	2	169.47	313.394	0.4	0.4	0.126	0	0	0	0.07	NoExpr	Pending	2	131561347	131561348	131561348	A	G	missense_variant
7-28814083-28814084-T-G		1		Hnrnpl	I203R	1	IYSRTTDVL	4	1	169.83	367.18	0.4	0.8	185.06	0	0	0	0.15	NoExpr	Pending	7	28814083	28814084	28814084	T	G	missense_variant
5-146170326-146170327-T-C		3	1	Gm6309	I70V	1	YVHQMIHYI	2	3	169.86	138.385	0.5	0.4	0	0	0	0	0.036	NoExpr	Pending	5	146170326	146170327	146170327	T	C	missense_variant
17-35380588-35380590-CC-GG		1		H2-Q4	S216W	1	AYLEGACVQWI	10	1	170.61	128.777	0.21	0.2	130.46	0	0	0	0.033	NoExpr	Pending	17	35380588	35380590	35380589	CC	GG	missense_variant
4-123917596-123917597-T-G		1	1	Rragc	F22V	1	SYGAADSVP	8	2	170.64	254.736	0.7	0.7	46.1	0	0	0	0.306	NoExpr	Pending	4	123917596	123917597	123917597	T	G	missense_variant
3-82039854-82039855-G-A		1		Gucy1b1	T349I	1	SDIPLHDAI	9	1	170.71	2939.41	0.6	8.8	0.333	0	0	0	0.479	NoExpr	Pending	3	82039854	82039855	82039855	G	A	missense_variant
12-114682674-114682675-C-T		3		Ighv1-18	R103H	1	AYMELHSLTSE	6	3	171.28	521.86	0.3	0.72	0	0	0	0	0.033	NoExpr	Pending	12	114682674	114682675	114682675	C	T	missense_variant
12-114708691-114708692-T-G		3		Ighv1-19	N103H	1	AYMELHSLTSE	6	3	171.28	954.06	0.3	1.3	0	0	0	0	0.035	NoExpr	Pending	12	114708691	114708692	114708692	T	G	missense_variant
12-114829306-114829308-CG-TG		3		Ighv1-31	R103H	1	AYMELHSLTSE	6	3	171.28	521.86	0.3	0.72	0	0	0	0	0.192	NoExpr	Pending	12	114829306	114829308	114829307	CG	TG,TT	missense_variant
12-115208142-115208143-C-A		3		Ighv1-55	Q101H	1	AYMHLSSL	4	3	171.32	100.95	0.25	0.14	0	0	0	0	0.087	NoExpr	Pending	12	115208142	115208143	115208143	C	A,G,T	missense_variant
12-115461046-115461047-C-G		3		Ighv1-62-3	Q101H	1	AYMHLSSL	4	3	171.32	100.95	0.25	0.14	0	0	0	0	0.077	NoExpr	Pending	12	115461046	115461047	115461047	C	G,T	missense_variant
17-35471070-35471071-G-C		2		H2-Q10	E197Q	1	RYLQLGKETL	4	2	172.13	272.88	0.25	0.3	5.017	0	0	0	0.022	NoExpr	Pending	17	35471070	35471071	35471071	G	C	missense_variant
15-47838487-47838488-A-G		1	1	Csmd3	L1733P	1	GYVPQGYSTL	4	2	172.43	140.78	0.25	0.2	0.005	0	0	0	0.148	NoExpr	Pending	15	47838487	47838488	47838488	A	G	missense_variant
7-80513276-80513277-G-A		1		Blm	P109S	1	KGTCSESSL	7	1	172.47	1391.91	0.8	6.7	5.552	0	0	0	0.353	NoExpr	Pending	7	80513276	80513277	80513277	G	A	missense_variant
9-123828645-123828646-C-G		2	1	Fyco1	A822P	1	KPQKQEQEL	2	2	172.47	1828.1	0.4	5.4	2.016	0	0	0	0.111	NoExpr	Pending	9	123828645	123828646	123828646	C	G	missense_variant
7-38519255-38519256-G-A		1	1	Gm5591	T731I	1	ANISQSSAM	3	2	172.62	448.838	0.6	1.7	0	0	0	0	0.154	NoExpr	Pending	7	38519255	38519256	38519256	G	A	missense_variant
9-38473292-38473293-T-A		1		Olfr905	L182H	1	HYFCDIHPL	7	1	173.63	491.4	0.4	0.9	0	0	0	0	0.028	NoExpr	Pending	9	38473292	38473293	38473293	T	A	missense_variant
6-52729333-52729334-C-T		1		Tax1bp1	H107Y	1	CYVTYKGEI	5	1	174.35	104.847	0.8	0.4	94.297	0	0	0	0.173	NoExpr	Pending	6	52729333	52729334	52729334	C	T	missense_variant
12-113578539-113578540-C-T		1		Ighv5-2	R106K	1	LYLQMSSLK	9	1	174.62	321.433	0.56	0.85	0	0	0	0	0.021	NoExpr	Pending	12	113578539	113578540	113578540	C	T	missense_variant
5-7978488-7978489-T-G		1	2	Steap4	F356V	1	LGILGVFLF	6	3	174.92	144.82	0.8	0.6	0.818	0	0	0	0.174	NoExpr	Pending	5	7978488	7978489	7978489	T	G	missense_variant
X-96527522-96527523-C-G		1	1	Heph	I696M	1	PDNPGIFEM	9	2	176.24	260.178	1	1.4	0.031	0	0	0	0.081	NoExpr	Pending	X	96527522	96527523	96527523	C	G	missense_variant
17-37590083-37590084-C-T		3		Olfr114	G90S	1	SYISYGQCM	1	3	176.64	597.06	0.5	0.96	0	0	0	0	0.029	NoExpr	Pending	17	37590083	37590084	37590084	C	T	missense_variant
6-89747328-89747329-C-T		3	1	Vmn1r41	T284I	1	SYAIVSPFV	4	4	176.97	79.138	0.5	0.4	0	0	0	0	0.117	NoExpr	Pending	6	89747328	89747329	89747329	C	T	missense_variant
4-147755719-147755720-G-A		1	2	Zfp984	L225F	1	FTHQIHFSI	7	3	179.47	4786.3	0.8	7.2	15.937	0	0	0	0.102	NoExpr	Pending	4	147755719	147755720	147755720	G	A	missense_variant
9-32259654-32259655-C-T		2	1	Arhgap32	P1593S	1	LHAPSPSMI	5	3	180	594.415	0.4	0.95	0.023	0	0	0	0.554	NoExpr	Pending	9	32259654	32259655	32259655	C	T	missense_variant
X-155053804-155053805-A-T		2		Magea5	F197Y	1	IYMKGNYV	2	2	180.44	2689.06	0.3	3.5	0	0	0	0	0.352	NoExpr	Pending	X	155053804	155053805	155053805	A	T	missense_variant
7-47589881-47589882-C-T		1	1	Mrgpra3	D99N	1	TVNLLKFTL	3	2	181.06	1061.7	0.7	3.4	0	0	0	0	0.241	NoExpr	Pending	7	47589881	47589882	47589882	C	T	missense_variant
17-35264096-35264097-C-A		2		H2-D1	A201E	1	RYLKNGNETL	8	2	183.1	310.83	0.27	0.47	1154.2	0	0	0	0.009	NoExpr	Pending	17	35264096	35264097	35264097	C	A	missense_variant
2-86553787-86553788-A-T		1	1	Olfr1080	L112H	1	LFIGSEHFI	7	2	184.54	427.652	0.5	1.2	0	0	0	0	0.044	NoExpr	Pending	2	86553787	86553788	86553788	A	T	missense_variant
5-128797656-128797657-C-T		1		Rimbp2	G297E	1	SPTQVDSEI	8	1	186.25	274.641	0.6	1.3	0	0	0	0	0.039	NoExpr	Pending	5	128797656	128797657	128797657	C	T	missense_variant
16-43969362-43969363-C-T		1	2	Zdhhc23	G354D	1	ADMAYIFLI	2	3	188.73	209.329	0.8	0.8	0	0	0	0	0.267	NoExpr	Pending	16	43969362	43969363	43969363	C	T	missense_variant
7-26611663-26611664-G-A		1	1	Vmn1r185	L139F	1	SFMWVLHML	2	2	188.84	660.83	0.45	2.4	0	0	0	0	0.043	NoExpr	Pending	7	26611663	26611664	26611664	G	A	missense_variant
6-41355508-41355509-A-C		1		Try10	N33T	1	GYTCRETSV	7	1	188.91	318.61	0.6	0.9	0	0	0	0	0.021	NoExpr	Pending	6	41355508	41355509	41355509	A	C	missense_variant

14-46384000-46384001-T-G		1		Bmp4	N362T	1	LVTSVNSSI	3	1	191.47	225.99	0.6	0.7	0.348	0	0	0	0.074	NoExpr	Pending	14	46384000	46384001	46384001	T	G	missense_variant
6-48908805-48908806-C-G		1		Aoc1	S748R	1	RYNGTYKPV	1	1	192.02	113.47	0.4	0.5	0	0	0	0	0.061	NoExpr	Pending	6	48908805	48908806	48908806	C	G	missense_variant
16-63546172-63546173-T-G		1		Epha3	K971N	1	KIISTINAL	7	1	192.18	482.072	0.9	1.8	0	0	0	0	0.046	NoExpr	Pending	16	63546172	63546173	63546173	T	G	missense_variant
6-66637703-66637704-T-G		2	1	Vmn1r34	M17L	1	YFQAGLGAL	9	3	192.18	254.608	0.3	0.5	0	0	0	0	0.062	NoExpr	Pending	6	66637703	66637704	66637704	T	G	missense_variant
7-104411593-104411594-C-T		3		Trim30a	S325N	1	NYNTVPVSEI	1	3	192.8	42.92	0.2	0.2	14.606	0	0	0	0.585	NoExpr	Pending	7	104411593	104411594	104411594	C	T	missense_variant
4-147755956-147755957-C-T		1		Zfp984	G146R	1	STISLNQRI	8	1	194.27	254.608	0.79	0.79	15.937	0	0	0	0.033	NoExpr	Pending	4	147755956	147755957	147755957	C	T	missense_variant
7-85136411-85136412-C-T		1		Vmn2r67	S795N	1	VYHNTKGKTMV	4	1	194.41	226.039	0.38	0.3	0	0	0	0	0.02	NoExpr	Pending	7	85136411	85136412	85136412	C	T	missense_variant
2-30113327-30113328-C-A		1	1	Zer1	G26C	1	CYLLDKETL	1	2	196.2	275.86	0.8	1	5.688	0	0	0	0.091	NoExpr	Pending	2	30113327	30113328	30113328	C	A	missense_variant
5-124130480-124130481-A-T		1		Pitpnm2	F548Y	1	TYNGQVCLI	2	1	196.38	2150.63	0.6	4.9	4.365	0	0	0	0.223	NoExpr	Pending	5	124130480	124130481	124130481	A	T	missense_variant
2-103566901-103566902-G-C		1		Abtb2	G59A	1	CYSASMNSR	4	1	196.38	274.322	0.64	1.1	0.203	0	0	0	0.232	NoExpr	Pending	2	103566901	103566902	103566902	G	C	missense_variant
13-21674751-21674752-G-T		2	2	Olfr1360	T64N	1	TPMYFFLNNL	8	4	196.38	180.097	0.3	0.3	0	0	0	0	0.233	NoExpr	Pending	13	21674751	21674752	21674752	G	T	missense_variant
1-85259729-85259731-TC-CT		1		C130026I21Rik	D111S	1	AYPSLKETL	4	1	197.39	565.98	0.6	1.2	77.454	0	0	0	0.011	NoExpr	Pending	1	85259729	85259731	85259730	TC	CT	missense_variant
6-121784364-121784366-AG-CA		1	1	Gm7298	QV1293-1294HM	1	GSFSQKFHM	8	2	198.15	4731.51	1	7.7	0	0	0	0	0.063	NoExpr	Pending	6	121784364	121784366	121784365	AG	CA	missense_variant
7-4834243-4834244-T-G		4		Shisa7	K274T	1	LYNTMTPSNL	6	4	199.77	336.38	0.23	0.3	0	0	0	0	0.164	NoExpr	Pending	7	4834243	4834244	4834244	T	G	missense_variant
7-7394422-7394424-TG-CT		2	1	Vmn2r31	IN278-279ID	1	IYEETIDFI	7	3	200.03	119.43	0.46	0.2	0	0	0	0	0.062	NoExpr	Pending	7	7394422	7394424	7394423	TG	CT	missense_variant
18-6069918-6069919-C-T		4		Arhgap12	R284H	1	CYYYNHTTQ	6	4	200.95	183.269	0.6	0.6	8.436	0	0	0	0.031	NoExpr	Pending	18	6069918	6069919	6069919	C	T	missense_variant
4-47049323-47049324-G-A		1		Anks6	H194Y	1	GYEAVVRLL	2	1	201.96	3571.87	0.6	7.3	0.907	0	0	0	0.571	NoExpr	Pending	4	47049323	47049324	47049324	G	A	missense_variant
7-11880408-11880409-C-T		1		Vmn1r75	L23F	1	LGIFGNSSI	4	1	202.81	201.878	0.6	0.6	0	0	0	0	0.245	NoExpr	Pending	7	11880408	11880409	11880409	C	T	missense_variant
8-108947744-108947745-G-A		2	2	Zfhx3	S1809N	1	IPNAEFQL	3	4	204.3	371.823	0.2	0.4	1.272	0	0	0	0.237	NoExpr	Pending	8	108947744	108947745	108947745	G	A	missense_variant
17-20029552-20029553-T-C		2	1	Vmn2r104	S819G	1	SILASGTAL	6	3	204.4	149.31	0.5	0.4	0	0	0	0	0.373	NoExpr	Pending	17	20029552	20029553	20029553	T	C	missense_variant
13-21132993-21132994-T-A		3		Olfr263	F73Y	1	CYTTSIVPQML	2	3	204.5	1562.1	0.3	3.6	0	0	0	0	0.032	NoExpr	Pending	13	21132993	21132994	21132994	T	A	missense_variant
14-75283348-75283349-A-C		1		Cpb2	K401N	1	RYINPTCAEAL	4	1	204.59	270.77	0.2	0.42	0	0	0	0	0.031	NoExpr	Pending	14	75283348	75283349	75283349	A	C	missense_variant
2-98662347-98662348-C-T		1	2	Gm10801	H38Y	1	SFLAIFYVL	7	3	205.04	1422.36	1.1	3.5	0.12	0	0	0	0.017	NoExpr	Pending	2	98662347	98662348	98662348	C	T	missense_variant
11-98156651-98156652-G-A		2		Med1	S1106F	1	SHSSSSSFL	8	2	205.46	128.902	0.7	0.4	5.819	0	0	0	0.026	NoExpr	Pending	11	98156651	98156652	98156652	G	A	missense_variant
3-36076046-36076047-G-A		3		Acad9	G213D	1	KYFILNDSK	7	3	205.71	138.909	0.8	0.7	17.994	0	0	0	0.272	NoExpr	Pending	3	36076046	36076047	36076047	G	A	missense_variant
16-58824442-58824443-T-C		1		Olfr175	N89D	1	NFFSVDRRI	6	1	205.71	166.376	0.63	0.5	0	0	0	0	0.079	NoExpr	Pending	16	58824442	58824443	58824443	T	C	missense_variant
6-123741583-123741584-C-T		1	4	Vmn2r23	S632F	1	SYLLLVFLM	7	4	205.98	6966.27	0.9	15	0	0	0	0	0.02	NoExpr	Pending	6	123741583	123741584	123741584	C	T	missense_variant
5-48219744-48219745-A-C		1		Slit2	D409A	1	LYANKLQTV	3	1	206.17	2166.44	0.6	3.3	50.312	0	0	0	0.149	NoExpr	Pending	5	48219744	48219745	48219745	A	C	missense_variant
7-63758015-63758016-C-T		1		Otud7a	T689I	1	AAAAAATAI	9	1	207.3	3289.2	0.69	9.5	0.139	0	0	0	0.184	NoExpr	Pending	7	63758015	63758016	63758016	C	T	missense_variant
2-36859574-36859575-A-C		3		Olfr351	L258V	1	AYVCPPSVV	3	3	207.65	126.29	0.4	0.4	0	0	0	0	0.041	NoExpr	Pending	2	36859574	36859575	36859575	A	C	missense_variant
2-87544979-87544980-G-A		1	1	Olfr1128	S188F	1	ALFCSDTSI	3	2	208.09	369.199	0.6	1.2	0	0	0	0	0.218	NoExpr	Pending	2	87544979	87544980	87544980	G	A	missense_variant
2-86553789-86553790-C-G		1	1	Olfr1080	E111D	1	FIGSDLFIL	5	2	208.45	197.697	0.9	1.1	0	0	0	0	0.052	NoExpr	Pending	2	86553789	86553790	86553790	C	G	missense_variant
17-19394458-19394459-T-G		1		Vmn2r99	S814A	1	SILAASTAL	5	1	208.57	149.31	0.6	0.4	0	0	0	0	0.028	NoExpr	Pending	17	19394458	19394459	19394459	T	G	missense_variant
1-173480953-173480954-A-T		1		Ifi206	F492Y	1	SYQLTPSRW	2	1	211.39	1923.49	0.51	3.3	0.197	0	0	0	0.065	NoExpr	Pending	1	173480953	173480954	173480954	A	T	missense_variant
7-26611671-26611672-G-A		1	2	Vmn1r185	S136F	1	CFISLMWVL	2	3	211.88	968.479	0.72	3.7	0	0	0	0	0.043	NoExpr	Pending	7	26611671	26611672	26611672	G	A	missense_variant
3-5329501-5329502-A-G		2		Zfhx4	Y1026C	1	YYCAVCDCS	8	2	214.14	87.92	1.1	0.33	0.012	0	0	0	0.043	NoExpr	Pending	3	5329501	5329502	5329502	A	G	missense_variant
9-89603089-89603090-C-G		1	2	Minar1	A85P	1	PDIVTIFNL	1	3	216.47	206.933	1.2	0.9	0.012	0	0	0	0.192	NoExpr	Pending	9	89603089	89603090	89603090	C	G	missense_variant
10-128408556-128408557-C-T		1		Nabp2	G90S	1	LYTGRGSDL	7	1	216.82	572.915	0.48	1.7	52.09	0	0	0	0.053	NoExpr	Pending	10	128408556	128408557	128408557	C	T	missense_variant
2-91492595-91492596-G-A		1		Lrp4	D1142N	1	VYWTNTGTNR	5	1	218.04	347.68	0.4	0.6	0.163	0	0	0	0.419	NoExpr	Pending	2	91492595	91492596	91492596	G	A	missense_variant
7-5125817-5125818-C-T		2		Rasl2-9	E38K	1	KYVATLGVE	1	2	218.82	2618.73	1	6.8	0.236	0	0	0	0.046	NoExpr	Pending	7	5125817	5125818	5125818	C	T	missense_variant
7-102984444-102984445-G-A		1		Olfr578	L240F	1	AFNTCISHI	2	1	221.2	1042.53	0.6	2.9	0	0	0	0	0.505	NoExpr	Pending	7	102984444	102984445	102984445	G	A	missense_variant
1-139733518-139733519-A-C		1		Gm4788	L531V	1	KYVDGEKLSVL	3	1	221.76	232.745	0.3	0.2	0	0	0	0	0.47	NoExpr	Pending	1	139733518	139733519	139733519	A	C	missense_variant
5-129697820-129697821-G-A		1		Septin14	L97F	1	TYEFLERNI	4	1	222.89	256.012	0.5	0.58	0.092	0	0	0	0.312	NoExpr	Pending	5	129697820	129697821	129697821	G	A	missense_variant
5-139393032-139393033-G-A		1	2	Gpr146	V197M	1	MPGLAVLYAL	1	3	223.07	296.82	0.2	0.4	3.115	0	0	0	0.476	NoExpr	Pending	5	139393032	139393033	139393033	G	A	missense_variant
9-27019335-27019336-C-T		1		Vps26b	G121E	1	SYTEQNVKL	4	1	223.4	166.441	0.7	0.5	9.249	0	0	0	0.378	NoExpr	Pending	9	27019335	27019336	27019336	C	T	missense_variant
16-32753785-32753786-G-T		2	1	Muc4	A1221S	1	SPITSIQIL	1	2	223.61	328.852	0.49	1.2	0.018	0	0	0	0.022	NoExpr	Pending	16	32753785	32753786	32753786	G	T	missense_variant
3-73049203-73049204-G-A		1	1	Slitrk3	P745L	1	VGHVYEYIL	9	2	223.61	2781.92	0.92	12	0	0	0	0	0.474	NoExpr	Pending	3	73049203	73049204	73049204	G	A	missense_variant
15-76537777-76537778-T-C		1		Fbxl6	Q187R	1	SRLQSLTLI	2	1	226.51	274.641	0.7	0.6	29.379	0	0	0	0.217	NoExpr	Pending	15	76537777	76537778	76537778	T	C	missense_variant
17-36168009-36168010-C-T		1		Gm8909	E88K	1	EYWERKTQI	6	1	226.6	298.717	0.4	0.5	5.931	0	0	0	0.053	NoExpr	Pending	17	36168009	36168010	36168010	C	T	missense_variant
13-58337676-58337677-C-G		2		Kif27	A490P	1	KYQCALPADQV	7	2	227.12	301.912	0.4	0.32	0.007	0	0	0	0.044	NoExpr	Pending	13	58337676	58337677	58337677	C	G	missense_variant
16-32754702-32754703-G-A		1	1	Muc4	A1526T	1	TPITSTHIL	1	2	228.5	214.136	0.6	0.8	0.018	0	0	0	0.007	NoExpr	Pending	16	32754702	32754703	32754703	G	A	missense_variant
9-3001256-3001257-T-A		1	1	Gm10722	F111Y	1	FLIYSDFLF	4	2	229	160.262	1	0.7	0	0	0	0	0.119	NoExpr	Pending	9	3001256	3001257	3001257	T	A	missense_variant
6-69775920-69775921-T-G		2		Igkv5-45	K59T	1	WYQQTSHES	5	2	229.61	1294.97	0.8	3.6	0	0	0	0	0.217	NoExpr	Pending	6	69775920	69775921	69775921	T	G	missense_variant
18-68408021-68408022-C-G		2		Mc2r	A67P	1	MYFFICSLPI	9	2	231.3	337.91	0.35	0.43	0	0	0	0	0.145	NoExpr	Pending	18	68408021	68408022	68408022	C	G	missense_variant
5-71641090-71641091-T-C		1	2	Gabra4	R156G	1	FRIMGNGTI	5	3	231.79	558.586	0.7	1.7	0	0	0	0	0.247	NoExpr	Pending	5	71641090	71641091	71641091	T	C	missense_variant
2-86639305-86639306-A-G		2		Olfr1084	V134A	1	LYTAIVSHK	4	2	232.32	429.625	0.8	1.3	0	0	0	0	0.053	NoExpr	Pending	2	86639305	86639306	86639306	A	G	missense_variant
19-43704204-43704205-G-T		1		Entpd7	L66F	1	RYFARVGDL	3	1	233.36	332.7	0.5	0.7	1.605	0	0	0	0.048	NoExpr	Pending	19	43704204	43704205	43704205	G	T	missense_variant
7-26611615-26611616-G-T		2	1	Vmn1r185	P155T	1	FIIFVYTFI	7	3	234.47	1059.47	0.7	3	0	0	0	0	0.079	NoExpr	Pending	7	26611615	26611616	26611616	G	T	missense_variant
14-50515313-50515314-A-G		1	2	Olfr742	I37V	1	VTFFVVYIL	5	3	235.96	200.676	1.3	1.1	0	0	0	0	0.072	NoExpr	Pending	14	50515313	50515314	50515314	A	G	missense_variant
4-147178590-147178591-A-T		2		Zfp991	T142S	1	SSISLNQGI	2	2	236.04	254.608	0.69	0.79	5.091	0	0	0	0.667	NoExpr	Pending	4	147178590	147178591	147178591	A	T	missense_variant
4-146466246-146466247-A-T		2		Zfp992	T142S	1	SSISLNQGI	2	2	236.04	254.608	0.69	0.79	0.548	0	0	0	0.417	NoExpr	Pending	4	146466246	146466247	146466247	A	T	missense_variant
2-98662305-98662307-TG-AA		2		Gm10801	C24N	1	SRNFSFSMI	3	2	237.19	239.933	0.7	0.8	0.12	0	0	0	0.012	NoExpr	Pending	2	98662305	98662307	98662306	TG	AA	missense_variant
6-122082717-122082718-G-A		2		Mug2	A1352T	1	SYTGSRPTS	8	2	238.28	251.868	1	1.2	0	0	0	0	0.433	NoExpr	Pending	6	122082717	122082718	122082718	G	A	missense_variant
5-106892702-106892703-T-A		1	2	Hfm1	H732L	1	LKNPSLYGF	6	3	238.61	249.158	1.4	1.4	0.005	0	0	0	0.669	NoExpr	Pending	5	106892702	106892703	106892703	T	A	missense_variant
7-42136836-42136837-T-C		1	2	Vmn2r60	F355L	1	LPKLWHLFF	4	3	239.88	237.684	0.6	0.5	0	0	0	0	0.184	NoExpr	Pending	7	42136836	42136837	42136837	T	C	missense_variant

9-27010422-27010423-G-A		1	1	Vps26b	R269C	1	CYYLNLVLI	1	2	239.93	670.023	0.6	1.6	9.249	0	0	0	0.226	NoExpr	Pending	9	27010422	27010423	27010423	G	A	missense_variant
8-69803533-69803534-G-C		1	2	Atp13a1	A801P	1	LPLEHALCL	2	3	241.2	6078.97	1	9.5	13.249	0	0	0	0.035	NoExpr	Pending	8	69803533	69803534	69803534	G	C	missense_variant
8-15998852-15998853-T-A		1		Csmd1	K2283M	1	MYQCHPGYTLL	1	1	241.68	157.06	0.4	0.23	0	0	0	0	0.259	NoExpr	Pending	8	15998852	15998853	15998853	T	A	missense_variant
19-11472464-11472465-T-C		1	3	Ms4a6c	Y74H	1	VPHFNSVFSVL	3	4	241.7	292.71	0.13	0.17	26.147	0	0	0	0.041	NoExpr	Pending	19	11472464	11472465	11472465	T	C	missense_variant
7-103328821-103328822-C-T		1	1	Olfr598	T112I	1	ILQGMESGI	1	2	242.15	338.135	0.7	1	0	0	0	0	0.042	NoExpr	Pending	7	103328821	103328822	103328822	C	T	missense_variant
11-114751822-114751823-C-T		1		Dnaic2	P417S	1	AYLSDGAWSSV	10	1	243.82	209.552	0.5	0.5	2.121	0	0	0	0.614	NoExpr	Pending	11	114751822	114751823	114751823	C	T	missense_variant
4-32707627-32707628-G-C		3		Mdn1	G1639A	1	VYIDAIGSGVT	5	3	243.82	306.024	0.5	0.6	3.122	0	0	0	0.205	NoExpr	Pending	4	32707627	32707628	32707628	G	C	missense_variant
13-62172900-62172901-G-A		1		Zfp808	R648Q	1	KAFSQHSTL	5	1	243.82	598.537	0.6	1.8	4.296	0	0	0	0.444	NoExpr	Pending	13	62172900	62172901	62172901	G	A	missense_variant
4-88602856-88602857-G-C		4	1	Ifna12	T151S	1	KYFHRISVYL	7	4	246.47	266.59	0.38	0.41	0	0	0	0	0.036	NoExpr	Pending	4	88602856	88602857	88602857	G	C	missense_variant
2-84940987-84940988-T-A		2	1	Slc43a3	Y108N	1	IFFNTCATI	4	3	248.25	334.264	0.7	0.8	2.597	0	0	0	0.263	NoExpr	Pending	2	84940987	84940988	84940988	T	A	missense_variant
11-78287788-78287789-T-A		1		2610507B11Rik	F2006I	1	GGISVKEHI	9	1	248.37	5471.29	0.7	14	51.913	0	0	0	0.296	NoExpr	Pending	11	78287788	78287789	78287789	T	A	missense_variant
14-4558365-4558366-C-T		1		Gm3047	A196V	1	KNICVSSAK	5	1	249.51	392.726	1.1	1.5	0	0	0	0	0.1	NoExpr	Pending	14	4558365	4558366	4558366	C	T	missense_variant
X-74303855-74303856-C-A		1		Atp6ap1	F414L	1	SYASDCAGL	9	1	249.88	1535.54	0.6	4.2	115.79	0	0	0	0.033	NoExpr	Pending	X	74303855	74303856	74303856	C	A	missense_variant
7-9986358-9986359-C-G		2		Vmn2r49	V402L	1	IYNALHAI	5	2	249.98	298.47	0.37	0.27	0	0	0	0	0.026	NoExpr	Pending	7	9986358	9986359	9986359	C	G	missense_variant
19-53635751-53635752-A-C		2		Smc3	D733A	1	KFKASRASI	7	2	250.7	156.84	0.4	0.3	45.699	0	0	0	0.101	NoExpr	Pending	19	53635751	53635752	53635752	A	C	missense_variant
19-53232137-53232138-G-C		2	1	Add3	K167N	1	TYISVRISN	9	3	253.57	179.924	0.7	0.6	14.006	0	0	0	0.047	NoExpr	Pending	19	53232137	53232138	53232138	G	C	missense_variant
6-126101834-126101835-G-A		1		Ntf3	T236I	1	IYVRALTSE	1	1	255.91	357.347	0.7	1.1	0	0	0	0	0.041	NoExpr	Pending	6	126101834	126101835	126101835	G	A	missense_variant
17-20540591-20540592-C-A		2	1	Vmn2r109	L834F	1	APKCYIILF	9	3	256.45	359.749	1.1	1.3	0	0	0	0	0.058	NoExpr	Pending	17	20540591	20540592	20540592	C	A	missense_variant
9-3025108-3025109-C-T		2	3	Gm10718	S191F	1	SVLFFSPYF	9	5	257.63	3647.54	1.1	9.1	0	0	0	0	0.115	NoExpr	Pending	9	3025108	3025109	3025109	C	T	missense_variant
18-44168135-44168136-T-G		2		Spinkl	K45T	1	MYKSTSECSN	5	2	258.54	1377.63	0.4	1.7	0	0	0	0	0.544	NoExpr	Pending	18	44168135	44168136	44168136	T	G	missense_variant
12-111669944-111669945-A-G		1	1	Ckb	C283R	1	GYILTRPSNL	6	2	258.61	245.1	0.2	0.38	5.226	0	0	0	0.051	NoExpr	Pending	12	111669944	111669945	111669945	A	G	missense_variant
10-22371399-22371400-C-T		1	2	Raet1d	P125L	1	YPHLQVTMIYL	11	3	259.48	7421.79	0.15	3.7	0.357	0	0	0	0.359	NoExpr	Pending	10	22371399	22371400	22371400	C	T	missense_variant
10-22181157-22181158-C-T		1	2	Raet1e	P127L	1	YPHLQVTMIYL	11	3	259.48	7421.79	0.15	3.7	0.469	0	0	0	0.177	NoExpr	Pending	10	22181157	22181158	22181158	C	T	missense_variant
17-38208357-38208358-G-A		2		Olfr135	A38T	1	YPTTMIGNI	4	2	260.18	218.82	1.3	1.1	0	0	0	0	0.141	NoExpr	Pending	17	38208357	38208358	38208358	G	A	missense_variant
3-5412115-5412116-T-G		2	1	Zfhx4	F3239V	1	SYVSPQLPGTV	3	3	260.18	150.77	0.6	0.3	0.012	0	0	0	0.261	NoExpr	Pending	3	5412115	5412116	5412116	T	G	missense_variant
2-146109987-146109988-G-A		2		Cfap61	G797S	1	RYTSTVPSNL	4	2	260.3	304.93	0.4	0.43	0.01	0	0	0	0.163	NoExpr	Pending	2	146109987	146109988	146109988	G	A	missense_variant
17-23345958-23345959-T-G		2		Vmn2r115	I273M	1	MYGDTDSTLAV	1	2	261.14	268.762	0.6	0.6	0	0	0	0	0.023	NoExpr	Pending	17	23345958	23345959	23345959	T	C,G	missense_variant
17-45568433-45568435-CA-TG		1		Hsp90ab1	M620T	1	GYTMAKKHL	3	1	261.87	80.11	0.5	0.3	1571.3	0	0	0	0.043	NoExpr	Pending	17	45568433	45568435	45568434	CA	TG	missense_variant
7-45651032-45651033-T-C		3	1	Fut2	N105S	1	SALAPIFRI	1	4	262.32	316.104	1.3	1.7	0.52	0	0	0	0.019	NoExpr	Pending	7	45651032	45651033	45651033	T	C	missense_variant
2-167104677-167104678-C-T		1		Kcnb1	G750E	1	FEAEVHQYI	4	1	262.48	189.71	0.7	0.6	0.008	0	0	0	0.464	NoExpr	Pending	2	167104677	167104678	167104678	C	T	missense_variant
3-104085275-104085276-C-G		1		Magi3	E293D	1	NYMMRDDNL	7	1	262.48	266.47	0.6	0.6	2.582	0	0	0	0.111	NoExpr	Pending	3	104085275	104085276	104085276	C	G	missense_variant
13-59474612-59474613-C-G		1		Agtpbp1	S933T	1	SNTPTAQSL	3	1	263.01	323.035	1.3	1.6	9.431	0	0	0	0.066	NoExpr	Pending	13	59474612	59474613	59474613	C	G	missense_variant
13-116303260-116303261-C-T		1		Isl1	V184M	1	KTTRMRTVL	5	1	263.01	388.269	1.3	2	0	0	0	0	0.178	NoExpr	Pending	13	116303260	116303261	116303261	C	T	missense_variant
1-171699598-171699599-C-T		2		Cd48	P198S	1	FYTCQVSNS	9	2	263.08	371.612	0.7	1.1	8.577	0	0	0	0.022	NoExpr	Pending	1	171699598	171699599	171699599	C	T	missense_variant
17-37299758-37299759-G-A		3	1	Olfr101	T221I	1	SYFYIIINL	7	3	264.4	38.823	0.6	0.2	0	0	0	0	0.04	NoExpr	Pending	17	37299758	37299759	37299759	G	A	missense_variant
1-171573824-171573825-G-C		2		Cd244a	W69S	1	SYNDGPSWSNY	1	2	264.51	461.62	0.2	0.21	0.896	0	0	0	0.052	NoExpr	Pending	1	171573824	171573825	171573825	G	C	missense_variant
2-89816453-89816454-A-G		4		Olfr1255	I43V	1	LYMITVSGNL	6	4	264.53	295.57	0.27	0.45	0	0	0	0	0.443	NoExpr	Pending	2	89816453	89816454	89816454	A	G	missense_variant
17-35471053-35471054-G-C		1		H2-Q10	W191S	1	AYLEAECVESL	10	1	265.39	271.686	0.4	0.6	5.017	0	0	0	0.036	NoExpr	Pending	17	35471053	35471054	35471054	G	C	missense_variant
17-80216999-80217000-C-G		3	1	Ttc39d	L363V	1	EVMWCHIFL	2	4	265.46	454.988	1.4	2.1	0.01	0	0	0	0.182	NoExpr	Pending	17	80216999	80217000	80217000	C	G	missense_variant
17-19811942-19811943-A-G		1	1	Vmn2r103	T660A	1	TTFAVTFAM	8	2	265.87	238.605	0.8	0.8	0	0	0	0	0.427	NoExpr	Pending	17	19811942	19811943	19811943	A	G	missense_variant
4-88827848-88827849-C-A		3		Ifna6	T145K	1	KYFHRITVYL	1	3	266.59	577.28	0.41	0.77	0	0	0	0	0.052	NoExpr	Pending	4	88827848	88827849	88827849	C	A	missense_variant
18-32863222-32863223-T-C		2		Wdr36	F775L	1	LYLKLEEGL	1	2	266.85	180.87	0.7	0.3	44.327	0	0	0	0.079	NoExpr	Pending	18	32863222	32863223	32863223	T	C	missense_variant
17-37590033-37590034-C-A		1	1	Olfr114	W106C	1	CFAWGEMAI	1	2	267.97	350.825	0.8	0.95	0	0	0	0	0.016	NoExpr	Pending	17	37590033	37590034	37590034	C	A	missense_variant
3-148823702-148823703-C-G		4		Adgrl2	G1159A	1	YSSATQSRI	4	4	268.76	358.996	1.3	1.4	2.653	0	0	0	0.039	NoExpr	Pending	3	148823702	148823703	148823703	C	G	missense_variant
17-35471086-35471087-C-T		3		H2-Q10	T202M	1	RYLELGKEMLL	9	3	268.76	143.492	0.4	0.2	5.017	0	0	0	0.043	NoExpr	Pending	17	35471086	35471087	35471087	C	T	missense_variant
17-35380621-35380622-C-T		3		H2-Q4	T227M	1	RYLELGKEMLL	9	3	268.76	143.492	0.4	0.2	130.46	0	0	0	0.036	NoExpr	Pending	17	35380621	35380622	35380622	C	T	missense_variant
12-115765432-115765433-C-T		3		Ighv8-13	R68Q	1	HYNPSLKSQL	9	3	269.14	460.45	0.24	0.36	0	0	0	0	0.034	NoExpr	Pending	12	115765432	115765433	115765433	C	T	missense_variant
7-9976860-9976861-T-C		1	1	Vmn2r49	K648R	1	LPNRVICVL	4	2	269.67	207.74	0.7	0.7	0	0	0	0	0.038	NoExpr	Pending	7	9976860	9976861	9976861	T	C	missense_variant
1-84964237-84964238-T-G		1		AC167036.1	F190L	1	SLALLLSVI	4	1	271.08	272.327	0.8	0.8	0	0	0	0	0.008	NoExpr	Pending	1	84964237	84964238	84964238	T	G	missense_variant
1-85028274-85028275-A-C		1		AC167036.2	F190L	1	SLALLLSVI	4	1	271.08	272.327	0.8	0.8	0	0	0	0	0.037	NoExpr	Pending	1	85028274	85028275	85028275	A	C	missense_variant
13-81440119-81440120-C-G		1		Adgrv1	G4449A	1	GYVLHASSV	6	1	271.69	280.649	1.3	1.4	0.021	0	0	0	0.028	NoExpr	Pending	13	81440119	81440120	81440120	C	G	missense_variant
9-37711664-37711666-TG-CA		2	1	Olfr160	FN204-205FD	1	VFFLAGFDI	8	3	271.69	223.607	1.3	0.9	0	0	0	0	0.047	NoExpr	Pending	9	37711664	37711666	37711665	TG	CA	missense_variant
12-115847919-115847920-G-T		2		Ighv1-76	S104R	1	AYMQLSRL	7	2	271.73	100.95	0.42	0.14	0	0	0	0	0.05	NoExpr	Pending	12	115847919	115847920	115847920	G	T	missense_variant
12-115861905-115861906-G-C		2		Ighv1-77	S104R	1	AYMQLSRL	7	2	271.73	100.95	0.42	0.14	0	0	0	0	0.035	NoExpr	Pending	12	115861905	115861906	115861906	G	C	missense_variant
6-57137805-57137806-A-C		2		Vmn1r11	S152R	1	AYTNVRETN	6	2	272.33	319.22	0.8	0.9	0	0	0	0	0.644	NoExpr	Pending	6	57137805	57137806	57137806	A	C	missense_variant
2-76892550-76892551-T-C		1		Ttn	K5680R	1	KYICQVRND	7	1	273.69	507.294	0.8	1.6	0.056	0	0	0	0.209	NoExpr	Pending	2	76892550	76892551	76892551	T	C	missense_variant
14-39472102-39472103-G-A		2	1	Nrg3	S233F	1	AYATSFYL	6	3	274.3	84.09	0.42	0.2	0	0	0	0	0.816	NoExpr	Pending	14	39472102	39472103	39472103	G	A	missense_variant
9-15352941-15352942-C-T		1		Cep295	S136N	1	LYLANLRHM	5	1	275.48	94.643	0.8	0.3	4.325	0	0	0	0.428	NoExpr	Pending	9	15352941	15352942	15352942	C	T	missense_variant
15-99986085-99986086-A-G		1		Larp4	Y68C	1	EYEVMCSPS	6	1	277.06	740.14	0.7	1.1	30.133	0	0	0	0.029	NoExpr	Pending	15	99986085	99986086	99986086	A	G	missense_variant
5-71623936-71623937-G-A		1		Gabra4	A351V	1	NYFTNIQMQKV	11	1	277.63	1133.26	0.2	1.5	0	0	0	0	0.255	NoExpr	Pending	5	71623936	71623937	71623937	G	A	missense_variant
7-20811666-20811668-AC-TG		1		Vmn1r114	KF173-174NI	1	NNTDSKSNI	8	1	278.03	10497.6	0.8	21	0	0	0	0	0.136	NoExpr	Pending	7	20811666	20811668	20811667	AC	TG	missense_variant
11-46577224-46577225-C-T		1	1	BC053393	S42F	1	SYPEFRILSFV	5	2	278.3	433.81	0.4	0.61	0	0	0	0	0.482	NoExpr	Pending	11	46577224	46577225	46577225	C	T	missense_variant
X-3194934-3194935-G-T		2		Btbd35f24	L270I	1	LYINPCWNGTM	3	2	279.82	246.477	0.43	0.5	0	0	0	0	0.193	NoExpr	Pending	X	3194934	3194935	3194935	G	T	missense_variant
16-87437318-87437319-C-T		1		Rwdd2b	S61N	1	VYFTINVNL	8	1	280.02	155.51	0.8	0.6	4.873	0	0	0	0.543	NoExpr	Pending	16	87437318	87437319	87437319	C	T	missense_variant
2-36568684-36568685-C-T		1	4	Olfr344	S29F	1	PENQVIYSF	9	5	280.65	2844.46	1.6	12	0	0	0	0	0.031	NoExpr	Pending	2	36568684	36568685	36568685	C	T	missense_variant

11-57289373-57289374-C-T		5	1	Gria1	L717F	1	YFLESTMNEYI	2	6	280.93	2795.74	0.3	1.4	0.016	0	0	0	0.593	NoExpr	Pending	11	57289373	57289374	57289374	C	T	missense_variant
3-90056567-90056568-C-G		1		4933434E20Rik	A109G	1	YRMKALDGI	8	1	281.36	117.56	0.8	0.4	53.51	0	0	0	0.194	NoExpr	Pending	3	90056567	90056568	90056568	C	G	missense_variant
4-147678312-147678313-C-A		2	1	Zfp534	D21Y	1	KDVALYFSL	6	3	281.73	381.795	0.69	1.6	0.043	0	0	0	0.326	NoExpr	Pending	4	147678312	147678313	147678313	C	A	missense_variant
2-25906707-25906709-AA-TT		2		Kcnt1	K780L	1	AYGFLNKLI	5	2	282.21	1738.84	0.6	3.4	0	0	0	0	0.231	NoExpr	Pending	2	25906707	25906709	25906708	AA	TT	missense_variant
1-12872308-12872309-A-C		1	1	Slco5a1	I704M	1	AYIPTPMYF	7	2	282.66	450.049	0.6	1.2	0	0	0	0	0.102	NoExpr	Pending	1	12872308	12872309	12872309	A	C	missense_variant
11-119436165-119436166-T-G		1		Rnf213	F1661V	1	VYLNFYTAEQL	1	1	283.7	174.345	0.21	0.2	5.721	0	0	0	0.26	NoExpr	Pending	11	119436165	119436166	119436166	T	G	missense_variant
4-147390321-147390322-A-G		1		Zfp978	I109V	1	KCNESSNVI	8	1	284.62	347.608	0.8	1.2	3.726	0	0	0	0.304	NoExpr	Pending	4	147390321	147390322	147390322	A	G	missense_variant
16-32752924-32752925-A-C		2	1	Muc4	Q934H	1	TSIHMLSTL	4	2	285.82	216.317	0.6	0.5	0.018	0	0	0	0.027	NoExpr	Pending	16	32752924	32752925	32752925	A	C	missense_variant
9-45450582-45450583-G-A		1		Dscaml1	V214I	1	EYISVVSWE	3	1	286.48	1172.44	0.8	2.9	0	0	0	0	0.186	NoExpr	Pending	9	45450582	45450583	45450583	G	A	missense_variant
9-38378165-38378166-A-G		1		Olfr251	Q95R	1	SFVTERNTI	6	1	286.79	363.864	0.9	1.2	0	0	0	0	0.252	NoExpr	Pending	9	38378165	38378166	38378166	A	G	missense_variant
10-77548638-77548639-G-A		1	1	Itgb2	C198Y	1	KAYQPPFAF	3	2	288.29	207.297	1	1.2	24.023	0	0	0	0.284	NoExpr	Pending	10	77548638	77548639	77548639	G	A	missense_variant
2-85400458-85400459-G-A		2	1	Olfr992	H25Y	1	GQNESWYIL	7	3	288.96	1383.03	1.2	3.7	0	0	0	0	0.371	NoExpr	Pending	2	85400458	85400459	85400459	G	A	missense_variant
11-73187746-73187747-G-A		1	1	Ctns	P184L	1	LYIQEEFLL	1	1	289.91	864.682	1.6	3.9	6.519	0	0	0	0.535	NoExpr	Pending	11	73187746	73187747	73187747	G	A	missense_variant
17-36167930-36167932-GC-TT		1		Gm8909	A114K	1	YYNQSKGGS	6	1	289.91	340.993	1.4	1.7	5.931	0	0	0	0.08	NoExpr	Pending	17	36167930	36167932	36167931	GC	TT	missense_variant
2-87628700-87628701-G-T	1	2	1	Olfr1131	K79N	1	CYSTAIGPNML	9	4	291.42	461.94	0.45	0.6	0	0	0	0	0.232	NoExpr	Pending	2	87628700	87628701	87628701	G	T	missense_variant
1-58125045-58125046-G-C		1		Aox3	R158S	1	CRCTGYSPI	7	1	293.83	975.192	0.61	2.4	0	0	0	0	0.03	NoExpr	Pending	1	58125045	58125046	58125046	G	C	missense_variant
7-102753071-102753072-G-A		2		Olfr560	P286S	1	TYLLISPV	6	2	296.56	801.611	0.45	0.8	0	0	0	0	0.259	NoExpr	Pending	7	102753071	102753072	102753072	G	A	missense_variant
12-114914645-114914647-GC-AC		2		Ighv1-39	QL101-102QF	1	AYMQFNSL	5	2	296.88	148.46	0.45	0.21	0	0	0	0	0.037	NoExpr	Pending	12	114914645	114914647	114914646	GC	AC,AT	missense_variant
16-29260943-29260944-C-G		2	2	Atp13a5	A939P	1	QYLLQDVPI	8	4	297.23	157.493	0.52	0.4	0	0	0	0	0.25	NoExpr	Pending	16	29260943	29260944	29260944	C	G	missense_variant
12-55676671-55676672-T-C		2		Ralgapa1	T1690A	1	QYLGTASPECL	6	2	297.43	472.835	0.46	0.71	6.158	0	0	0	0.505	NoExpr	Pending	12	55676671	55676672	55676672	T	C	missense_variant
1-20520287-20520288-C-T		1		Pkhd1	G1745D	1	NYGCLDGRL	6	1	298.6	296.545	0.8	0.8	0.002	0	0	0	0.606	NoExpr	Pending	1	20520287	20520288	20520288	C	T	missense_variant
1-154448915-154448916-G-A		1		Cacna1e	T1256I	1	KIIKSLRVL	2	1	299.47	492.617	1.5	2.1	0	0	0	0	0.171	NoExpr	Pending	1	154448915	154448916	154448916	G	A	missense_variant
17-35380584-35380585-C-A		1		H2-Q4	Q215K	1	AYLEGACVKSL	9	1	299.77	128.777	0.4	0.2	130.46	0	0	0	0.076	NoExpr	Pending	17	35380584	35380585	35380585	C	A,G	missense_variant
11-9577190-9577191-T-G		2		Abca13	F4535V	1	SRIVSSSDV	4	2	300.67	460.352	0.9	1.4	0.011	0	0	0	0.246	NoExpr	Pending	11	9577190	9577191	9577191	T	G	missense_variant
7-103155588-103155589-T-G		1		Olfr589	T53P	1	FVIKPESSL	5	1	300.67	128.777	1.3	0.6	0	0	0	0	0.292	NoExpr	Pending	7	103155588	103155589	103155589	T	G	missense_variant
2-85991848-85991849-T-G		1		Olfr1031	F11V	1	NFTSVTEVI	8	1	301.48	378.521	0.8	0.9	0	0	0	0	0.205	NoExpr	Pending	2	85991848	85991849	85991849	T	G	missense_variant
1-173860338-173860339-C-A		1	1	Mndal	S443I	1	KTQNTIYEI	6	2	301.88	438.359	1.2	1.8	91.682	0	0	0	0.143	NoExpr	Pending	1	173860338	173860339	173860339	C	A	missense_variant
1-66414347-66414348-A-T		4	2	Map2	K799I	1	YYINGTVMAPD	3	6	302.61	2339.71	0.7	4.5	0.017	0	0	0	0.225	NoExpr	Pending	1	66414347	66414348	66414348	A	T	missense_variant
X-79355059-79355060-A-C		1		Cfap47	L2332R	1	AFQPRTQNI	5	1	302.75	104.014	0.9	0.3	0	0	0	0	0.429	NoExpr	Pending	X	79355059	79355060	79355060	A	C	missense_variant
7-8368096-8368097-G-A		4	1	Vmn2r44	T650I	1	HPNRAICVL	6	5	303.97	434.34	0.5	0.9	0	0	0	0	0.022	NoExpr	Pending	7	8368096	8368097	8368097	G	A	missense_variant
5-52848759-52848760-A-T		1		Anapc4	L330F	1	GFKKLGQSI	2	1	304.85	1066.82	0.9	3	41.553	0	0	0	0.185	NoExpr	Pending	5	52848759	52848760	52848760	A	T	missense_variant
12-105222561-105222562-G-A		1		Tcl1	R31C	1	VYLDEFCRSWL	7	1	306.02	265.87	0.6	0.6	0	0	0	0	0.051	NoExpr	Pending	12	105222561	105222562	105222562	G	A	missense_variant
3-101439441-101439442-G-C		1	1	Igsf3	L564F	1	VTYSDSFDF	9	2	306.78	435.341	1.3	1.5	0.702	0	0	0	0.065	NoExpr	Pending	3	101439441	101439442	101439442	G	C	missense_variant
6-17571461-17571462-T-G		1		Met	L1295V	1	IYLVQGRRL	4	1	307.79	547.15	0.52	0.7	23.781	0	0	0	0.026	NoExpr	Pending	6	17571461	17571462	17571462	T	G	missense_variant
2-132879719-132879720-G-T		1		Lrrn4	P59H	1	LHAAGVTTL	2	1	307.86	944.257	1.2	2.7	0	0	0	0	0.521	NoExpr	Pending	2	132879719	132879720	132879720	G	T	missense_variant
14-14087496-14087497-T-G		1	1	Atxn7	H225Q	1	KPMQPVQQI	4	1	309.35	346.011	1.5	1.4	2.263	0	0	0	0.188	NoExpr	Pending	14	14087496	14087497	14087497	T	G	missense_variant
16-88671403-88671404-G-A		2		Krtap27-1	A84V	1	QNSRVEQTI	5	2	310.52	511.988	0.9	1.6	0	0	0	0	0.054	NoExpr	Pending	16	88671403	88671404	88671404	G	A	missense_variant
10-41523226-41523227-G-A		1		Cd164	R104H	1	NHTDLCSVI	2	1	311.24	409.345	0.6	1.2	84.859	0	0	0	0.043	NoExpr	Pending	10	41523226	41523227	41523227	G	A	missense_variant
9-49023933-49023934-C-T	1	3		Usp28	R439W	1	KYGSGPSWF	8	4	311.95	319.22	0.75	0.9	4.554	0	0	0	0.055	NoExpr	Pending	9	49023933	49023934	49023934	C	T	missense_variant
8-25596378-25596379-T-A		3	1	Letm2	L10F	1	SYNSFFAI	6	3	313.23	276.45	0.47	0.43	1.045	0	0	0	0.229	NoExpr	Pending	8	25596378	25596379	25596379	T	A	missense_variant
2-145604547-145604548-G-A		3		Slc24a3	R292H	1	SHFPPKTHL	8	3	313.39	468.911	0.9	1.4	0.105	0	0	0	0.36	NoExpr	Pending	2	145604547	145604548	145604548	G	A	missense_variant
5-65825383-65825384-C-G		3	1	N4bp2	S1660R	1	KYLTRHSFR	5	4	313.71	88.973	1.2	0.3	2.576	0	0	0	0.13	NoExpr	Pending	5	65825383	65825384	65825384	C	G	missense_variant
19-11521762-11521764-AG-CA		2	2	Ms4a6b	SV80-81SI	1	HFTSVFSIL	8	3	314.84	282.657	0.8	0.7	9.701	0	0	0	0.049	NoExpr	Pending	19	11521762	11521764	11521763	AG	CA	missense_variant
17-36168007-36168008-C-G		1		Gm8909	E88D	1	EYWERDTQI	6	1	314.96	298.717	0.5	0.5	5.931	0	0	0	0.053	NoExpr	Pending	17	36168007	36168008	36168008	C	G	missense_variant
2-88728951-88728952-A-G		1	1	Olfr1197	Y216H	1	SHILILYSL	2	2	316.1	420.562	1.3	1.8	0	0	0	0	0.243	NoExpr	Pending	2	88728951	88728952	88728952	A	G	missense_variant
6-4516913-4516914-G-A		1		Col1a2	M191I	1	KGVKGHSGI	9	1	316.12	1145.59	1.5	4	211.55	0	0	0	0.887	NoExpr	Pending	6	4516913	4516914	4516914	G	A	missense_variant
2-86958857-86958858-A-C		2		Olfr1099	F200C	1	ICIFAGSTL	2	2	317.15	153.2	0.9	0.3	0	0	0	0	0.314	NoExpr	Pending	2	86958857	86958858	86958858	A	C	missense_variant
9-26894302-26894303-C-A		2		Gm1110	G351V	1	DYNALVSEV	9	2	317.88	2223.77	0.5	3.9	0	0	0	0	0.079	NoExpr	Pending	9	26894302	26894303	26894303	C	A	missense_variant
9-122378952-122378953-G-C		2		Abhd5	G304A	1	IDANSGTSI	3	2	320.69	1082.08	0.9	2.5	14.1	0	0	0	0.036	NoExpr	Pending	9	122378952	122378953	122378953	G	C	missense_variant
17-51742704-51742705-C-G		1		Satb1	V574L	1	IYEQESNAL	9	1	323.04	714.644	0.9	1.9	0.092	0	0	0	0.041	NoExpr	Pending	17	51742704	51742705	51742705	C	G	missense_variant
5-96769650-96769651-G-A		1		Fras1	R3510K	1	FYDTVLWKTGI	8	1	323.5	323.504	0.4	0.4	0.136	0	0	0	0.476	NoExpr	Pending	5	96769650	96769651	96769651	G	A	missense_variant
11-3140172-3140173-C-T		4		Sfi1	A716T	1	IYYRQKEATAL	9	4	325.07	626.14	0.2	0.83	12.698	0	0	0	0.015	NoExpr	Pending	11	3140172	3140173	3140173	C	T	missense_variant
7-7479794-7479795-T-C		1		Vmn2r32	N60S	1	YFSKTLNFL	3	1	325.28	257.686	0.9	0.7	0	0	0	0	0.054	NoExpr	Pending	7	7479794	7479795	7479795	T	C	missense_variant
1-189657513-189657514-G-A		2		Cenpf	H1374Y	1	FYDKANHSSV	2	2	325.46	1937.53	0.2	0.7	14.05	0	0	0	0.048	NoExpr	Pending	1	189657513	189657514	189657514	G	A	missense_variant
2-180733805-180733806-T-A		1		Slc17a9	S174T	1	TYSTVGAGTQV	9	1	325.46	337.33	0.4	0.4	1	0	0	0	0.274	NoExpr	Pending	2	180733805	180733806	180733806	T	A	missense_variant
5-36967404-36967405-A-G		2		Wfs1	I714T	1	KYVRVTETD	8	2	325.53	889.734	0.8	2.4	5.2	0	0	0	0.031	NoExpr	Pending	5	36967404	36967405	36967405	A	G	missense_variant
2-87435373-87435374-T-A		1	1	Olfr1124	Y296N	1	TPMFNPMVNSI	9	2	326.54	146.43	0.2	0.2	0	0	0	0	0.422	NoExpr	Pending	2	87435373	87435374	87435374	T	A	missense_variant
11-96107512-96107513-G-A		3	1	Calcoco2	A28V	1	KFYVPRGDI	4	4	326.66	211.831	0.9	0.6	0	0	0	0	0.045	NoExpr	Pending	11	96107512	96107513	96107513	G	A	missense_variant
15-102982069-102982070-G-T		3		Hoxc9	D140Y	1	YYMYGSPGEL	1	3	328	2121.28	0.49	2.4	0.092	0	0	0	0.174	NoExpr	Pending	15	102982069	102982070	102982070	G	T	missense_variant
16-32754346-32754347-C-T		2	1	Muc4	T1407I	1	APITSIQIL	6	2	328.85	500.035	1.2	2	0.018	0	0	0	0.058	NoExpr	Pending	16	32754346	32754347	32754347	C	T	missense_variant
11-67205509-67205510-G-A		1		Myh1	E272K	1	TYLLKKSRV	5	1	330.01	291.44	1.6	1.1	1.093	0	0	0	0.564	NoExpr	Pending	11	67205509	67205510	67205510	G	A	missense_variant
11-54352116-54352117-G-C		1		Acsl6	M651I	1	TYQELCIKKEL	7	1	330.1	143.02	0.32	0.2	0.051	0	0	0	0.046	NoExpr	Pending	11	54352116	54352117	54352117	G	C	missense_variant
8-19906445-19906446-C-T		1		Gm31371	T144I	1	SSISPLSSL	3	1	330.1	864.682	0.9	2.4	0	0	0	0	0.049	NoExpr	Pending	8	19906445	19906446	19906446	C	T	missense_variant
2-86922884-86922885-C-A		4		Olfr1098	A216S	1	SSYVSILSTIL	1	4	331.18	480.72	0.5	0.5	0	0	0	0	0.034	NoExpr	Pending	2	86922884	86922885	86922885	C	A	missense_variant
3-108402733-108402734-T-C		2		Celsr2	D1560G	1	RHIDMAGFI	7	2	331.33	356.93	0.51	0.53	0.008	0	0	0	0.392	NoExpr	Pending	3	108402733	108402734	108402734	T	C	missense_variant

5-109047017-109047018-G-C		2		Vmn2r11	T814R	1	VYHSTRGRV	8	2	333.95	59.09	0.8	0.4	0	0	0	0	0.04	NoExpr	Pending	5	109047017	109047018	109047018	G	C	missense_variant
17-29627179-29627180-G-C		2		Rnf8	E305D	1	LYADQAQQQ	4	2	334.26	323.661	1	0.9	11.251	0	0	0	0.242	NoExpr	Pending	17	29627179	29627180	29627180	G	C	missense_variant
X-74303847-74303848-G-T		1		Atp6ap1	A412S	1	SYASDCSGF	7	1	337.19	1535.54	0.75	4.2	115.79	0	0	0	0.042	NoExpr	Pending	X	74303847	74303848	74303848	G	T	missense_variant
16-58824093-58824095-GA-AT		1	2	Olfr175	S205I	1	SGIVQTFSI	3	2	337.32	1085.26	0.91	2.8	0	0	0	0	0.012	NoExpr	Pending	16	58824093	58824095	58824094	GA	AT	missense_variant
12-114538690-114538692-CA-GT		2		Ighv1-7	W52T	1	GYTFTSYTM	8	2	337.51	1007.14	0.6	2.2	0	0	0	0	0.022	NoExpr	Pending	12	114538690	114538692	114538691	CA	GT	missense_variant
2-86131198-86131199-C-T		3	1	Olfr1039	V155M	1	STYIYGFTM	9	4	339.63	4120.98	1.4	5.8	0	0	0	0	0.23	NoExpr	Pending	2	86131198	86131199	86131199	C	T	missense_variant
16-58916367-58916368-C-T		1		Olfr180	R91K	1	NFFSVDRKI	8	1	339.83	205.712	1	0.63	0	0	0	0	0.023	NoExpr	Pending	16	58916367	58916368	58916368	C	T	missense_variant
6-40900068-40900069-G-A		1	1	Prss58	L9F	1	AFLCIFSTL	6	2	343.2	329.678	0.8	0.8	0	0	0	0	0.837	NoExpr	Pending	6	40900068	40900069	40900069	G	A	missense_variant
2-98662377-98662379-CC-TC		1	2	Gm10801	P48S	1	SPFSVFLAI	1	2	344.7	1109	1.2	2	0.12	0	0	0	0.023	NoExpr	Pending	2	98662377	98662379	98662378	CC	TC,TT	missense_variant
10-102385080-102385081-G-A		1		Mgat4c	D75N	1	RYVHTFKNL	8	1	344.81	668.64	0.6	0.86	0	0	0	0	0.043	NoExpr	Pending	10	102385080	102385081	102385081	G	A	missense_variant
12-113611289-113611290-G-A		3		Ighv2-3	L82F	1	NYHSAFISRL	6	3	346.39	284.66	0.51	0.44	0	0	0	0	0.065	NoExpr	Pending	12	113611289	113611290	113611290	G	A	missense_variant
15-78254866-78254867-G-C		2		Ncf4	E141D	1	FYQSAYDAD	9	2	351.63	266.846	1	0.7	26.748	0	0	0	0.073	NoExpr	Pending	15	78254866	78254867	78254867	G	C	missense_variant
8-105270613-105270614-A-C		2	2	Hsf4	K64T	1	LPQYFTHSNM	6	4	352.24	374.72	0.5	0.5	7.76	0	0	0	0.194	NoExpr	Pending	8	105270613	105270614	105270614	A	C	missense_variant
16-16868672-16868673-C-T		1		Vpreb1	G118R	1	YYCAVRLRS	6	1	352.24	432.636	1.3	1.9	0.337	0	0	0	0.61	NoExpr	Pending	16	16868672	16868673	16868673	C	T	missense_variant
1-167226427-167226428-G-A		2	1	Uck2	P247L	1	GYLNGYTL	8	3	352.51	1661.88	0.47	2.4	42.334	0	0	0	0.173	NoExpr	Pending	1	167226427	167226428	167226428	G	A	missense_variant
13-65296554-65296555-C-G		1		Zfp369	T504S	1	KTQSSRDPI	4	1	353.26	315.99	1	1.2	4.175	0	0	0	0.128	NoExpr	Pending	13	65296554	65296555	65296555	C	G	missense_variant
12-65055164-65055165-C-T		2		Prpf39	P339S	1	SYFHVKPL	1	2	353.39	896.314	0.27	0.7	12.18	0	0	0	0.333	NoExpr	Pending	12	65055164	65055165	65055165	C	T	missense_variant
1-84963776-84963777-G-A		2		AC167036.1	V37I	1	FSITTSLTI	9	2	354.13	2234.91	0.8	6	0	0	0	0	0.065	NoExpr	Pending	1	84963776	84963777	84963777	G	A,T	missense_variant
12-114896406-114896407-C-A		1		Ighv1-37	G61V	1	SHVKSLEWI	3	1	355.71	534.884	1	1.4	0	0	0	0	0.011	NoExpr	Pending	12	114896406	114896407	114896407	C	A	missense_variant
12-51365553-51365554-C-T		1		G2e3	S459F	1	NFQAVKENL	2	1	356.07	1709.59	0.7	5.1	5.55	0	0	0	0.32	NoExpr	Pending	12	51365553	51365554	51365554	C	T	missense_variant
14-4635225-4635226-G-A		2		Gm8159	R148H	1	YSIKQKHHL	8	2	356.07	520.002	1.6	2.6	0	0	0	0	0.14	NoExpr	Pending	14	4635225	4635226	4635226	G	A	missense_variant
1-174191043-174191044-G-A		1		Spta1	E527K	1	AFTAQKEKI	6	1	358.17	453.169	1.1	1.8	0.01	0	0	0	0.206	NoExpr	Pending	1	174191043	174191044	174191044	G	A	missense_variant
15-78297059-78297060-G-C		1		Csf2rb2	T44S	1	CYNDYSNRI	6	1	358.59	501.291	0.9	1.2	3.122	0	0	0	0.74	NoExpr	Pending	15	78297059	78297060	78297060	G	C	missense_variant
3-55255881-55255882-G-A		1		Dclk1	G132S	1	GESYVCSSI	7	1	359	948.615	1.1	2.8	0.131	0	0	0	0.462	NoExpr	Pending	3	55255881	55255882	55255882	G	A	missense_variant
10-83233649-83233650-C-T		1	2	Slc41a2	G546D	1	IPYLTALDDLL	8	3	359.68	141.87	0.07	0.06	5.301	0	0	0	0.609	NoExpr	Pending	10	83233649	83233650	83233650	C	T	missense_variant
11-3153459-3153460-C-T		1		Sfi1	R438Q	1	QYVHKRQWQQ	1	1	359.95	182.339	0.7	0.4	12.698	0	0	0	0.016	NoExpr	Pending	11	3153459	3153460	3153460	C	T	missense_variant
12-115648055-115648056-A-C		2		Ighv8-12	S83A	1	RYNPALKSRL	5	2	360.34	299.46	0.49	0.38	0	0	0	0	0.008	NoExpr	Pending	12	115648055	115648056	115648056	A	C	missense_variant
11-98400221-98400222-G-C		2		Pgap3	S58R	1	IYMRLAGWT	4	2	360.8	285.161	0.7	0.77	2.121	0	0	0	0.091	NoExpr	Pending	11	98400221	98400222	98400222	G	C	missense_variant
17-36167979-36167980-C-T		1		Gm8909	G98S	1	SFQGSLRNL	1	1	361.63	1528.48	1.1	2.8	5.931	0	0	0	0.065	NoExpr	Pending	17	36167979	36167980	36167980	C	T	missense_variant
7-5125778-5125780-CT-TG		1	1	Rasl2-9	LV50-51FI	1	EVHTFIFHT	5	2	362.24	445.656	1.7	1.8	0.236	0	0	0	0.039	NoExpr	Pending	7	5125778	5125780	5125779	CT	TG	missense_variant
9-38816146-38816147-G-T		2	2	Olfr922	V215F	1	ITIFVSYTF	4	4	362.94	470.793	1.4	1.7	0	0	0	0	0.036	NoExpr	Pending	9	38816146	38816147	38816147	G	T	missense_variant
7-21133364-21133365-A-C		2	2	Vmn1r122	F255C	1	FYLLNCICI	6	4	363.15	562.92	0.7	0.8	0	0	0	0	0.062	NoExpr	Pending	7	21133364	21133365	21133365	A	C	missense_variant
1-14887649-14887650-T-G		1	1	Trpa1	N695H	1	MVQHHRIEL	5	2	363.77	389.789	1.3	1.3	0.026	0	0	0	0.155	NoExpr	Pending	1	14887649	14887650	14887650	T	G	missense_variant
1-171573831-171573832-T-A		1		Cd244a	N71K	1	VYKDGPSWSN	3	1	363.81	461.62	0.5	0.21	0.896	0	0	0	0.134	NoExpr	Pending	1	171573831	171573832	171573832	T	A	missense_variant
6-80021913-80021914-A-G		1		Lrrtm4	E103G	1	NYISSVDGD	8	1	364.83	358.996	1.2	1.1	0	0	0	0	0.159	NoExpr	Pending	6	80021913	80021914	80021914	A	G	missense_variant
2-87269648-87269649-C-A		2		Olfr1116	F268L	1	LYTILIPAL	1	2	368.21	246.752	1	0.6	0	0	0	0	0.23	NoExpr	Pending	2	87269648	87269649	87269649	C	A	missense_variant
14-52390484-52390485-A-G	1	1	1	Olfr1511	V96A	1	NFTPASKAI	8	3	369.2	676.488	0.76	1.4	0	0	0	0	0.031	NoExpr	Pending	14	52390484	52390485	52390485	A	G	missense_variant
16-57133127-57133128-A-C		1		Tomm70a	K129Q	1	KYFQAGKYE	4	1	369.2	283.962	0.8	0.6	48.119	0	0	0	0.263	NoExpr	Pending	16	57133127	57133128	57133128	A	C	missense_variant
7-85624395-85624396-C-T		1	1	Vmn2r71	A806V	1	MVVVEVFCI	2	2	371.39	677.376	1.9	2.6	0	0	0	0	0.027	NoExpr	Pending	7	85624395	85624396	85624396	C	T	missense_variant
8-92996027-92996029-CT-TG		1	3	Slc6a2	P551L	1	TYLPWANWV	3	4	371.82	2740.81	1.9	9.3	0	0	0	0	0.306	NoExpr	Pending	8	92996027	92996029	92996028	CT	TG	missense_variant
18-60550219-60550220-A-C		1	1	Dctn4	N290T	1	TPTSIKFKI	1	2	373.1	299.473	1.5	1.2	18.155	0	0	0	0.378	NoExpr	Pending	18	60550219	60550220	60550220	A	C	missense_variant
1-155789391-155789392-C-T		2		Qsox1	G268R	1	SYLRRLPGL	5	2	373.47	260.178	1.1	0.46	23.2	0	0	0	0.641	NoExpr	Pending	1	155789391	155789392	155789392	C	T	missense_variant
5-8397260-8397261-T-C		1	1	Dbf4	T650A	1	AFFSSPSTS	1	2	374.19	687.211	1.4	1.8	64.601	0	0	0	0.156	NoExpr	Pending	5	8397260	8397261	8397261	T	C	missense_variant
17-58967344-58967345-A-T		2		Pdzph1	S835T	1	SSAGGSTVI	7	2	375.05	257.093	1.3	0.8	0	0	0	0	0.155	NoExpr	Pending	17	58967344	58967345	58967345	A	T	missense_variant
3-98619430-98619431-T-C		2		Hsd3b5	H233R	1	VYVGNAAWAR	10	2	375.56	243.824	0.7	0.5	0	0	0	0	0.175	NoExpr	Pending	3	98619430	98619431	98619431	T	C	missense_variant
7-98161940-98161941-G-A		3		Capn5	P47L	1	LYYKGTPGL	9	3	375.87	3616.26	1.8	9	11.475	0	0	0	0.245	NoExpr	Pending	7	98161940	98161941	98161941	G	A	missense_variant
12-114746405-114746407-TT-AA		1		Ighv1-22	N73F	1	GYINPFNGGTS	6	1	375.87	562.186	0.8	2.2	0	0	0	0	0.054	NoExpr	Pending	12	114746405	114746407	114746406	TT	AA	missense_variant
3-64086727-64086728-C-T		1		Vmn2r1	A165V	1	SLSVAVSRI	6	1	376.78	333.496	1.5	1.3	0	0	0	0	0.262	NoExpr	Pending	3	64086727	64086728	64086728	C	T	missense_variant
X-73448840-73448841-C-T		1		Haus7	S138N	1	KILDMMQNL	8	1	377.65	200.489	1.4	0.9	42.369	0	0	0	0.285	NoExpr	Pending	X	73448840	73448841	73448841	C	T	missense_variant
6-132803761-132803762-A-G		1	1	Tas2r117	I288V	1	FPTFHSYVL	8	2	382.88	169.044	0.4	0.3	0	0	0	0	0.065	NoExpr	Pending	6	132803761	132803762	132803762	A	G	missense_variant
12-115593306-115593307-A-C		1		Ighv1-66	Y52D	1	GYSFTSYDI	8	1	383.05	186.03	0.9	0.3	0	0	0	0	0.04	NoExpr	Pending	12	115593306	115593307	115593307	A	C	missense_variant
3-79480804-79480805-G-A		1		Fnip2	P843L	1	GYKGLSSEPV	5	1	384.09	525.658	0.32	0.37	4.29	0	0	0	0.401	NoExpr	Pending	3	79480804	79480805	79480805	G	A	missense_variant
17-27431511-27431512-G-C		2	1	Grm4	L837V	1	VYMPKVYII	1	2	384.09	293.062	2	1.6	0	0	0	0	0.054	NoExpr	Pending	17	27431511	27431512	27431512	G	C	missense_variant
17-23401616-23401617-A-T		1	1	Vmn2r116	N775I	1	RNLPGAFIE	8	2	384.09	299.473	2	1.7	0	0	0	0	0.018	NoExpr	Pending	17	23401616	23401617	23401617	A	T	missense_variant
18-6992834-6992835-C-T		2		Mkx	V150I	1	KYIQGNAERL	3	2	384.83	686.32	0.56	0.89	0	0	0	0	0.327	NoExpr	Pending	18	6992834	6992835	6992835	C	T	missense_variant
8-110866086-110866087-G-A		1		Cog4	V432I	1	EYFMRETI	8	1	385.15	478.035	0.2	0.34	28.128	0	0	0	0.886	NoExpr	Pending	8	110866086	110866087	110866087	G	A	missense_variant
1-44156942-44156943-G-A		2		Ercc5	S32N	1	INIWLNQAL	2	2	385.56	755.249	1.2	2.1	9.77	0	0	0	0.17	NoExpr	Pending	1	44156942	44156943	44156943	G	A	missense_variant
8-105949816-105949817-C-G		2		Slc12a4	G491A	1	KYGDAVSRNL	5	2	386.16	1180	0.56	0.78	31.134	0	0	0	0.211	NoExpr	Pending	8	105949816	105949817	105949817	C	G	missense_variant
8-82864146-82864147-C-G		1		Rnf150	I46M	1	WYTAFVNMTY	8	1	387.87	267.87	0.3	0.4	0.277	0	0	0	0.089	NoExpr	Pending	8	82864146	82864147	82864147	C	G	missense_variant
18-46850859-46850860-T-A		1		Lvrn	H223Q	1	LYQDEDELRAI	3	1	388.27	659.754	0.8	1.2	0.111	0	0	0	0.209	NoExpr	Pending	18	46850859	46850860	46850860	T	A	missense_variant
19-40723131-40723132-C-T		1		Entpd1	A132V	1	VYLAECMEL	1	1	388.83	254.84	1.2	0.8	3.248	0	0	0	0.191	NoExpr	Pending	19	40723131	40723132	40723132	C	T	missense_variant
4-89691819-89691820-G-C		1		Dmrta1	C339S	1	SKGDVVQAI	1	1	391.98	574.46	1.2	1.9	0.008	0	0	0	0.347	NoExpr	Pending	4	89691819	89691820	89691820	G	C	missense_variant
2-111420896-111420897-C-T		1		Olfr1286	C18Y	1	LYSSRQLEI	2	1	392.11	9080.09	0.7	20	0	0	0	0	0.538	NoExpr	Pending	2	111420896	111420897	111420897	C	T	missense_variant
3-5402307-5402308-G-T		1	2	Zfhx4	D2509Y	1	RPMDMPYMIFY	11	3	392.45	409.84	0.2	0.27	0.012	0	0	0	0.246	NoExpr	Pending	3	5402307	5402308	5402308	G	T	missense_variant
5-125454549-125454550-C-G		1		Bri3bp	P187A	1	KYEGEAEHAV	6	1	392.49	525.658	0.6	0.9	6.864	0	0	0	0.063	NoExpr	Pending	5	125454549	125454550	125454550	C	G	missense_variant

9-114629057-114629058-T-C		2		Cnot10	T127A	1	QYAEAISVGEK	3	2	392.49	531.377	0.8	1.6	20.452	0	0	0	0.205	NoExpr	Pending	9	114629057	114629058	114629058	T	C	missense_variant
9-20286211-20286212-G-A		2		Olfr39	G179D	1	LTFSMDTEI	6	2	392.73	390.023	1.2	0.96	0	0	0	0	0.158	NoExpr	Pending	9	20286211	20286212	20286212	G	A	missense_variant
1-46325767-46325768-C-T		1		Dnah7b	P3570S	1	SYPSSNFPV	5	1	394.69	1062.03	1.2	3.5	0	0	0	0	0.03	NoExpr	Pending	1	46325767	46325768	46325768	C	T	missense_variant
17-36165685-36165686-A-C		1		Gm8909	S271A	1	KWAAVVVPL	4	1	395.6	591.916	1.1	1.2	5.931	0	0	0	0.024	NoExpr	Pending	17	36165685	36165686	36165686	A	C	missense_variant
17-35473270-35473271-T-G		1		H2-Q10	S270A	1	KWAAVVVPL	4	1	395.6	591.916	1.1	1.2	5.017	0	0	0	0.036	NoExpr	Pending	17	35473270	35473271	35473271	T	G	missense_variant
10-8207552-8207553-C-T		2	1	Ust	R353K	1	HYVKEQFHL	4	3	396.28	411.15	2.1	1.9	0.197	0	0	0	0.564	NoExpr	Pending	10	8207552	8207553	8207553	C	T	missense_variant
2-86347205-86347206-T-G		1		Olfr1055	M187L	1	FYCDSLPLILL	11	1	396.76	636.62	0.8	2.4	0	0	0	0	0.186	NoExpr	Pending	2	86347205	86347206	86347206	T	G	missense_variant
3-55477848-55477849-A-C		1		Dclk1	K360N	1	SSTNVCSSM	4	1	397.27	340.993	1.7	1.7	0.131	0	0	0	0.409	NoExpr	Pending	3	55477848	55477849	55477849	A	C	missense_variant
9-39217761-39217762-C-T		4	1	Olfr944	T135I	1	LYNVIMSYQ	5	5	397.27	222.89	1.2	0.6	0	0	0	0	0.27	NoExpr	Pending	9	39217761	39217762	39217762	C	T	missense_variant
6-42838692-42838693-G-A		1	3	Olfr449	V271I	1	KIISIFYAI	2	3	399.79	274.05	1.4	1.1	0	0	0	0	0.841	NoExpr	Pending	6	42838692	42838693	42838693	G	A	missense_variant
4-32738698-32738699-G-A		2		Mdn1	G3551S	1	RYRSRSSRTAL	7	2	399.83	314.688	0.5	0.55	3.122	0	0	0	0.072	NoExpr	Pending	4	32738698	32738699	32738699	G	A	missense_variant
17-37382615-37382616-A-C		1	1	Olfr105-ps	Q16H	1	GYSDIHELNPI	6	2	400.6	759.819	1.1	1.2	0	0	0	0	0.223	NoExpr	Pending	17	37382615	37382616	37382616	A	C	missense_variant
7-45651030-45651031-C-T		1	3	Fut2	A106T	1	MHNTLAPIF	4	4	401.08	323.035	1.5	1.3	0.52	0	0	0	0.023	NoExpr	Pending	7	45651030	45651031	45651031	C	T	missense_variant
17-37952445-37952446-C-A		2		Olfr761	D193Y	1	YFGVLEVSI	1	2	401.08	5371.43	0.9	9.2	0	0	0	0	0.019	NoExpr	Pending	17	37952445	37952446	37952446	C	A	missense_variant
8-70891658-70891659-A-C		1		Slc5a5	V139G	1	QYLGATML	4	1	401.29	406.874	0.3	0.3	0.039	0	0	0	0.268	NoExpr	Pending	8	70891658	70891659	70891659	A	C	missense_variant
6-132803729-132803730-T-C		1	1	Tas2r117	V277A	1	AYIAFPTFH	1	2	401.87	899.684	1.2	2.5	0	0	0	0	0.101	NoExpr	Pending	6	132803729	132803730	132803730	T	C	missense_variant
17-30656922-30656923-C-G		1	1	Dnah8	A508G	1	NGIRMIHSV	2	2	403.89	666.945	1.3	2.1	0.115	0	0	0	0.086	NoExpr	Pending	17	30656922	30656923	30656923	C	G	missense_variant
11-70316308-70316309-C-G		1	1	Alox12e	G557A	1	IPNAPCTM	4	2	405.44	1073.27	0.4	0.71	0	0	0	0	0.177	NoExpr	Pending	11	70316308	70316309	70316309	C	G	missense_variant
12-113306413-113306414-G-C		2		Ighg2b	T329S	1	KNYYLKKSI	8	2	405.44	337.357	2	1.4	0	0	0	0	0.026	NoExpr	Pending	12	113306413	113306414	113306414	G	C	missense_variant
12-115193691-115193692-C-T		1		Ighv1-54	V112I	1	SLTSEDSAI	9	1	405.44	2270.34	2	9.1	0	0	0	0	0.028	NoExpr	Pending	12	115193691	115193692	115193692	C	T	missense_variant
12-115208111-115208113-CC-TC		1		Ighv1-55	AV111-112AI	1	SLTSEDSAI	9	1	405.44	2270.34	2	9.1	0	0	0	0	0.059	NoExpr	Pending	12	115208111	115208113	115208112	CC	TC,GA	missense_variant
12-115847897-115847899-CA-TC		1		Ighv1-76	AV111-112AI	1	SLTSEDSAI	9	1	405.44	2270.34	2	9.1	0	0	0	0	0.039	NoExpr	Pending	12	115847897	115847899	115847898	CA	TC	missense_variant
5-129132765-129132766-C-T		1		Adgrd1	R318C	1	CFQRPGTVL	1	1	405.45	713.91	1.3	1.2	0.758	0	0	0	0.029	NoExpr	Pending	5	129132765	129132766	129132766	C	T	missense_variant
9-3024516-3024517-C-T		1		Gm10718	A167V	1	SMIFSFLVI	8	1	405.59	244.957	1.2	0.7	0	0	0	0	0.031	NoExpr	Pending	9	3024516	3024517	3024517	C	T	missense_variant
12-115868811-115868812-G-C		2		Ighv1-78	S104R	1	AYMQLNRLT	7	2	406.53	122.487	1.1	0.4	0	0	0	0	0.035	NoExpr	Pending	12	115868811	115868812	115868812	G	C	missense_variant
1-171573832-171573833-G-A		2		Cd244a	D72N	1	VYNNGPSWSN	4	2	407.88	461.62	0.3	0.21	0.896	0	0	0	0.051	NoExpr	Pending	1	171573832	171573833	171573833	G	A	missense_variant
17-20030004-20030005-G-C		1		Vmn2r104	T668S	1	FTMALASVL	7	1	409.51	638.646	1.2	1.3	0	0	0	0	0.041	NoExpr	Pending	17	20030004	20030005	20030005	G	C	missense_variant
9-104123116-104123117-C-T		2		Acad11	A690V	1	LTLKVAHSI	5	2	410.29	762.535	1.7	2.3	2.665	0	0	0	0.061	NoExpr	Pending	9	104123116	104123117	104123117	C	T	missense_variant
3-116923537-116923538-C-T		1		Palmd	D437N	1	GYNGVIHAE	3	1	411.4	1546.18	1.2	4.3	1.34	0	0	0	0.046	NoExpr	Pending	3	116923537	116923538	116923538	C	T	missense_variant
16-17574522-17574523-G-T		1		Slc7a4	A349D	1	VYAMADDGL	6	1	411.4	315.689	1	0.53	0.045	0	0	0	0.277	NoExpr	Pending	16	17574522	17574523	17574523	G	T	missense_variant
7-5480797-5480798-C-G		2		Vmn2r28	S801T	1	VYHTTKGKV	4	2	411.84	378.02	1.1	0.9	0	0	0	0	0.018	NoExpr	Pending	7	5480797	5480798	5480798	C	G	missense_variant
12-113685542-113685543-C-T		1		Ighv2-5	V97I	1	IFFKMNSLQ	1	1	412.34	701.601	1.2	2.2	0	0	0	0	0.037	NoExpr	Pending	12	113685542	113685543	113685543	C	T	missense_variant
17-13073603-13073604-G-A		1	1	Tcp10b	D305N	1	SNQKVIYYY	2	2	414	579.202	1.7	2.3	0.013	0	0	0	0.039	NoExpr	Pending	17	13073603	13073604	13073604	G	A	missense_variant
17-35321057-35321058-C-A		1		H2-Q1	T101N	1	RFQESLSNL	8	1	414.07	108.77	0.58	0.3	6.324	0	0	0	0.023	NoExpr	Pending	17	35321057	35321058	35321058	C	A	missense_variant
2-66317951-66317952-T-G		1		Scn1a	K1094T	1	TGSSVETYI	7	1	414.09	1574.93	1.2	4.3	0	0	0	0	0.232	NoExpr	Pending	2	66317951	66317952	66317952	T	G	missense_variant
14-53337566-53337567-G-A		1		Trav13n-3	E89K	1	ASNKSRSSL	4	1	414.31	690.383	1.8	2.2	0	0	0	0	0.044	NoExpr	Pending	14	53337566	53337567	53337567	G	A	missense_variant
2-86639294-86639296-GA-AT		2		Olfr1084	SH137-138SY	1	LYTVIVSYK	8	2	416	429.625	1.3	1.3	0	0	0	0	0.022	NoExpr	Pending	2	86639294	86639296	86639295	GA	AT	missense_variant
8-63927832-63927833-A-C		1		Sgo2b	V655G	1	FFTRGNGSL	5	1	416.16	228.171	0.96	0.64	0	0	0	0	0.252	NoExpr	Pending	8	63927832	63927833	63927833	A	C	missense_variant
7-105024326-105024327-G-A		1	1	Olfr675	L214F	1	LLLDVVFII	7	2	417.67	10491.3	1.8	20	0	0	0	0	0.25	NoExpr	Pending	7	105024326	105024327	105024327	G	A	missense_variant
11-71156153-71156154-T-G		1		Nlrp1b	N1127T	1	SYEAVRAET	9	1	417.92	468.911	0.96	1.4	2.122	0	0	0	0.037	NoExpr	Pending	11	71156153	71156154	71156154	T	G	missense_variant
10-127064639-127064640-G-A		1		Cdk4	V92M	1	KVTLMFEHI	5	1	419.85	590.323	1.3	2.3	387.78	0	0	0	0.148	NoExpr	Pending	10	127064639	127064640	127064640	G	A	missense_variant
16-25871091-25871092-G-C		1		Trp63	R436S	1	TYSQQQQQQ	3	1	420.01	970.711	1.3	2.9	0.038	0	0	0	0.184	NoExpr	Pending	16	25871091	25871092	25871092	G	C	missense_variant
18-73931082-73931083-C-T		1	1	Mapk4	S356N	1	YPVNLSSDL	4	2	420.1	487.316	1.4	2	0	0	0	0	0.333	NoExpr	Pending	18	73931082	73931083	73931083	C	T	missense_variant
X-102071364-102071365-C-T		2		Nhsl2	H108Y	1	AYSRSSWRQ	2	2	421.95	8876.87	1.6	19	0.174	0	0	0	0.783	NoExpr	Pending	X	102071364	102071365	102071365	C	T	missense_variant
1-85591759-85591760-G-A		3	1	Sp110	P116S	1	SPLTLLEDL	1	4	422.48	1121.07	1.9	3.3	20.029	0	0	0	0.018	NoExpr	Pending	1	85591759	85591760	85591760	G	A	missense_variant
12-115193818-115193821-CAC-ATA		2		Ighv1-54	V69Y	1	GYINPGSGGTN	2	2	422.55	6829.04	1.2	13	0	0	0	0	0.032	NoExpr	Pending	12	115193818	115193821	115193819	CAC	ATA	missense_variant
2-33565196-33565197-C-T		2		Lmx1b	M282I	1	GIMASYTPL	2	2	422.76	263.26	0.9	0.8	0	0	0	0	0.103	NoExpr	Pending	2	33565196	33565197	33565197	C	T	missense_variant
17-47869256-47869257-C-T		1		Foxp4	S586N	1	SYQAALAEN	9	1	424.71	376.782	1.3	0.61	11.023	0	0	0	0.408	NoExpr	Pending	17	47869256	47869257	47869257	C	T	missense_variant
1-107271842-107271843-C-T		1		Serpinb3c	S316N	1	SGMNSTQGL	4	1	425.85	359.948	1.4	0.9	0	0	0	0	0.235	NoExpr	Pending	1	107271842	107271843	107271843	C	T	missense_variant
1-174612523-174612524-T-G		1		Fmn2	L1169R	1	KRDSSPSRI	8	1	427.98	489.88	2.199	1.7	0	0	0	0	0.193	NoExpr	Pending	1	174612523	174612524	174612524	T	G	missense_variant
2-40697359-40697360-C-T		1		Lrp1b	S3961N	1	KRQANNGLI	6	1	427.98	437.342	2.1	1.6	0.022	0	0	0	0.453	NoExpr	Pending	2	40697359	40697360	40697360	C	T	missense_variant
6-28526128-28526129-G-A		1		Snd1	G169S	1	SEGNSSHTI	5	1	428.64	736.36	1.3	2.3	152.16	0	0	0	0.946	NoExpr	Pending	6	28526128	28526129	28526129	G	A	missense_variant
1-174391788-174391789-C-G		1		Olfr248	A240G	1	AFTPCGSHL	6	1	428.81	408.69	1.6	1.3	0	0	0	0	0.165	NoExpr	Pending	1	174391788	174391789	174391789	C	G	missense_variant
7-85410214-85410215-C-A		1		Vmn2r69	M495I	1	LYMSKEII	7	1	429.19	314.72	0.45	0.2	0	0	0	0	0.046	NoExpr	Pending	7	85410214	85410215	85410215	C	A	missense_variant
X-93593841-93593842-C-T		1	1	Pola1	C501Y	1	RKIKGPYWL	7	2	431.35	5114.82	1.8	6.3	23.522	0	0	0	0.384	NoExpr	Pending	X	93593841	93593842	93593842	C	T	missense_variant
9-103479866-103479867-C-A		1		Bfsp2	M120I	1	EYITKVHAL	3	1	431.59	240.93	0.8	1	0.095	0	0	0	0.254	NoExpr	Pending	9	103479866	103479867	103479867	C	A	missense_variant
10-40920378-40920379-C-T		1		Wasf1	A35V	1	TNISLVNII	6	1	431.61	382.023	1.2	1	1.056	0	0	0	0.608	NoExpr	Pending	10	40920378	40920379	40920379	C	T	missense_variant
9-39950300-39950301-G-T		2	1	Olfr975	L157I	1	LHASVITFL	6	3	432.3	469.85	0.8	0.9	0	0	0	0	0.236	NoExpr	Pending	9	39950300	39950301	39950301	G	T	missense_variant
17-35264065-35264066-T-A		1		H2-D1	W191R	1	AYLEGECVERL	10	1	432.64	344.702	0.5	0.6	1154.2	0	0	0	0.025	NoExpr	Pending	17	35264065	35264066	35264066	T	A	missense_variant
11-110301544-110301545-T-G		1		Abca5	I714L	1	GYRLSMYL	8	1	433.11	388.29	0.25	0.3	3.228	0	0	0	0.264	NoExpr	Pending	11	110301544	110301545	110301545	T	G	missense_variant
7-12670590-12670591-T-G		1		Vmn2r55	N295T	1	PYQTSTVTEGV	4	1	433.22	883.597	0.91	1.5	0	0	0	0	0.152	NoExpr	Pending	7	12670590	12670591	12670591	T	G	missense_variant
9-106435842-106435843-C-T		1		Acy1	R126K	1	QYLEAVKRL	7	1	433.32	457.03	1.2	0.7	19.705	0	0	0	0.253	NoExpr	Pending	9	106435842	106435843	106435843	C	T	missense_variant
8-48276524-48276525-G-C		1		Tenm3	A1482G	1	CYQSGDGYG	9	1	433.6	308.503	0.89	0.9	4.183	0	0	0	0.157	NoExpr	Pending	8	48276524	48276525	48276525	G	C	missense_variant
1-165139186-165139187-C-T		2		Tbx19	A373T	1	VHTSTSGTI	3	2	433.77	360.795	0.8	0.6	0	0	0	0	0.052	NoExpr	Pending	1	165139186	165139187	165139187	C	T	missense_variant
1-164263402-164263403-C-G		1		Slc19a2	A461G	1	GSYFAAISVV	1	1	433.79	424.268	0.41	0.4	12.739	0	0	0	0.158	NoExpr	Pending	1	164263402	164263403	164263403	C	G	missense_variant

4-88602850-88602851-T-A		2	1	Ifna12	Y153F	1	KYFHRITVF	9	3	434.6	1528.48	1.2	2.9	0	0	0	0	0.03	NoExpr	Pending	4	88602850	88602851	88602851	T	A	missense_variant
4-88683321-88683322-T-A		2	1	Ifna2	Y153F	1	KYFHRITVF	9	3	434.6	1528.48	1.2	2.9	0	0	0	0	0.037	NoExpr	Pending	4	88683321	88683322	88683322	T	A	missense_variant
4-88835981-88835982-A-T		2	1	Ifna5	Y153F	1	KYFHRITVF	9	3	434.6	1528.48	1.2	2.9	0	0	0	0	0.024	NoExpr	Pending	4	88835981	88835982	88835982	A	T	missense_variant
12-115067757-115067758-C-T		1		Ighv8-5	G54S	1	SLSTSNMSI	8	1	437.34	1062.03	1	2.5	0	0	0	0	0.052	NoExpr	Pending	12	115067757	115067758	115067758	C	T	missense_variant
17-35471031-35471032-C-A		1		H2-Q10	L184M	1	AYMEAECVEWI	3	1	439.24	271.686	0.4	0.6	5.017	0	0	0	0.037	NoExpr	Pending	17	35471031	35471032	35471032	C	A	missense_variant
7-48871006-48871007-A-G	1	3		E2f8	I522T	1	TYLQPAQAQ	1	4	440.65	315.566	1.2	0.9	32.029	0	0	0	0.468	NoExpr	Pending	7	48871006	48871007	48871007	A	G	missense_variant
3-130620788-130620789-C-T		3		Etnppl	S111F	1	CYFTNFGSE	6	3	441.84	291.246	1.4	0.8	0	0	0	0	0.586	NoExpr	Pending	3	130620788	130620789	130620789	C	T	missense_variant
11-100041024-100041025-C-T		1		Krt34	E123K	1	KNARLVVQI	1	1	442.1	3460.11	1.5	7.3	0	0	0	0	0.037	NoExpr	Pending	11	100041024	100041025	100041025	C	T	missense_variant
5-16246668-16246669-C-A		1		Cacna2d1	T177N	1	IYEGSNIVL	6	1	444.9	724.87	1	1.2	7.938	0	0	0	0.071	NoExpr	Pending	5	16246668	16246669	16246669	C	A	missense_variant
8-13319799-13319800-C-T		2		Tmco3	P604L	1	ILLRSSQYI	3	2	444.9	1670.75	0.8	3.7	5.542	0	0	0	0.221	NoExpr	Pending	8	13319799	13319800	13319800	C	T	missense_variant
2-87192486-87192487-T-G		2		Olfr1112	L267V	1	TYVRPKSSH	3	2	445.75	287.799	1.2	0.8	0	0	0	0	0.032	NoExpr	Pending	2	87192486	87192487	87192487	T	G	missense_variant
17-35264062-35264063-G-A		1		H2-D1	E190K	1	AYLEGECVKWL	9	1	446.91	344.702	0.56	0.6	1154.2	0	0	0	0.025	NoExpr	Pending	17	35264062	35264063	35264063	G	A	missense_variant
12-114222980-114222981-C-T		2		Ighv7-4	R57H	1	YYMSWVHQP	7	2	446.91	696.429	1.1	1.8	0	0	0	0	0.034	NoExpr	Pending	12	114222980	114222981	114222981	C	T	missense_variant
18-34845348-34845349-C-T		1		Reep2	P94S	1	YRKFVHSTL	7	1	447.81	2951.82	1.4	7.5	0.141	0	0	0	0.202	NoExpr	Pending	18	34845348	34845349	34845349	C	T	missense_variant
17-35323257-35323258-G-A		1		H2-Q1	V269M	1	KWASVMVPL	6	1	450.05	591.916	1.1	1.2	6.324	0	0	0	0.031	NoExpr	Pending	17	35323257	35323258	35323258	G	A	missense_variant
17-35473276-35473277-G-A		1		H2-Q10	V272M	1	KWASVMVPL	6	1	450.05	591.916	1.1	1.2	5.017	0	0	0	0.037	NoExpr	Pending	17	35473276	35473277	35473277	G	A	missense_variant
6-132957684-132957685-T-C		1		Tas2r131	M54V	1	SRISSVLVL	8	1	451.77	652.654	1.3	1.8	0	0	0	0	0.113	NoExpr	Pending	6	132957684	132957685	132957685	T	C	missense_variant
8-26110759-26110760-A-G		2		Hook3	V40A	1	NGVAMSQVL	4	2	451.95	835.776	2	2.5	8.408	0	0	0	0.669	NoExpr	Pending	8	26110759	26110760	26110760	A	G	missense_variant
10-36828795-36828796-G-A		1		Hs3st5	G32R	1	LYLVARVRSL	8	1	454.42	720.211	0.32	0.3	0	0	0	0	0.231	NoExpr	Pending	10	36828795	36828796	36828796	G	A	missense_variant
12-113625613-113625614-C-G		1		Ighv5-6	S82T	1	SYTYYPDTV	8	1	454.62	778.45	2.1	2.5	0	0	0	0	0.013	NoExpr	Pending	12	113625613	113625614	113625614	C	G	missense_variant
1-174449438-174449439-C-T		4		Olfr220	A272V	1	TYARPKLMYV	10	4	455.08	1824.25	0.2	1.7	0	0	0	0	0.64	NoExpr	Pending	1	174449438	174449439	174449439	C	T	missense_variant
2-165304933-165304934-G-A		3		Elmo2	A241V	1	YAIALINVL	8	3	456.69	356.074	1.4	0.78	18.861	0	0	0	0.216	NoExpr	Pending	2	165304933	165304934	165304934	G	A	missense_variant
7-137459263-137459264-G-A		1	2	Glrx3	G209E	1	YPQLYVSEEL	8	3	457.83	599.55	0.6	0.5	145.98	0	0	0	0.382	NoExpr	Pending	7	137459263	137459264	137459264	G	A	missense_variant
17-34157316-34157317-G-T		1	1	H2-DMb1	V137F	1	EPVMLACYF	9	2	457.96	6439.17	1.3	5.4	1.435	0	0	0	0.059	NoExpr	Pending	17	34157316	34157317	34157317	G	T	missense_variant
17-34148621-34148622-G-T		1	1	H2-DMb2	V137F	1	EPVMLACYF	9	2	457.96	6439.17	1.3	5.4	2.435	0	0	0	0.02	NoExpr	Pending	17	34148621	34148622	34148622	G	T	missense_variant
2-86413927-86413929-AT-GC		2		Olfr1061	D41G	1	IYLTSLVGNL	8	2	457.99	438.53	0.58	0.61	0	0	0	0	0.025	NoExpr	Pending	2	86413927	86413929	86413928	AT	GC	missense_variant
12-113306429-113306430-A-G		1		Ighg2b	Y324H	1	KNHYLKKTI	3	1	459.29	337.357	1.4	1.4	0	0	0	0	0.013	NoExpr	Pending	12	113306429	113306430	113306430	A	G	missense_variant
7-41288151-41288152-T-C		1		Gm5592	V286A	1	NTCAQVSQI	4	1	461.65	647.277	2.301	2.8	0	0	0	0	0.024	NoExpr	Pending	7	41288151	41288152	41288152	T	C	missense_variant
4-118753784-118753785-A-C		1	1	Olfr1338	F251C	1	SHLTVVTCFYI	8	2	462.07	459.949	0.8	0.8	0	0	0	0	0.026	NoExpr	Pending	4	118753784	118753785	118753785	A	C	missense_variant
2-55437488-55437489-T-G		2		Kcnj3	F97V	1	TYTVAWLVM	8	2	462.66	623.864	1.3	1.5	0	0	0	0	0.243	NoExpr	Pending	2	55437488	55437489	55437489	T	G	missense_variant
2-181590077-181590078-T-A		1		Zfp512b	K124M	1	IYGLMYHYQ	5	1	463.72	10333.8	1.4	15	5.485	0	0	0	0.042	NoExpr	Pending	2	181590077	181590078	181590078	T	A	missense_variant
6-71625787-71625788-G-A		1		Kdm3a	P105S	1	SPEVSEQVI	5	1	465.68	1534.94	2.1	5.3	13.389	0	0	0	0.029	NoExpr	Pending	6	71625787	71625788	71625788	G	A	missense_variant
17-33999943-33999944-G-A		2	2	H2-K1	R42W	1	RPGLGEPWYMI	8	4	466.68	6044.62	0.7	3.1	663.43	0	0	0	0.123	NoExpr	Pending	17	33999943	33999944	33999944	G	A	missense_variant
17-35265980-35265981-G-A		1		H2-D1	V273M	1	KWASVVMPL	7	1	468.02	591.916	0.8	1.2	1154.2	0	0	0	0.064	NoExpr	Pending	17	35265980	35265981	35265981	G	A	missense_variant
1-105741328-105741329-G-A		2		Relch	G1028D	1	FDTIMETVI	2	2	468.91	287.799	1.4	0.8	8.032	0	0	0	0.159	NoExpr	Pending	1	105741328	105741329	105741329	G	A	missense_variant
2-98662349-98662350-C-A		1	1	Gm10801	H38Q	1	IFQVLHWTF	3	2	470.79	482.87	1.7	1.6	0.12	0	0	0	0.023	NoExpr	Pending	2	98662349	98662350	98662350	C	A	missense_variant
7-15555545-15555546-G-A		3	1	Obox1	G87S	1	KYVNKQTSPM	8	4	471.42	1410.05	0.49	1.4	0	0	0	0	0.084	NoExpr	Pending	7	15555545	15555546	15555546	G	A	missense_variant
17-17893094-17893095-C-G		1		Fpr2	L118V	1	AVIALDRCI	2	1	471.75	462.658	2.4	2.1	6.036	0	0	0	0.027	NoExpr	Pending	17	17893094	17893095	17893095	C	G	missense_variant
7-105600065-105600066-T-G		1		Hpx	N9H	1	ARTAVALHI	8	1	471.75	573.186	2.1	2.9	0.116	0	0	0	0.214	NoExpr	Pending	7	105600065	105600066	105600066	T	G	missense_variant
15-55212194-55212195-G-A		1		Deptor	S265N	1	SFMSVNPSKEI	6	1	473.54	460.66	0.63	0.64	1.164	0	0	0	0.891	NoExpr	Pending	15	55212194	55212195	55212195	G	A	missense_variant
12-113306426-113306427-A-G		2	1	Ighg2b	Y325H	1	KNYHLKKTI	4	3	474.53	337.357	1.4	1.4	0	0	0	0	0.013	NoExpr	Pending	12	113306426	113306427	113306427	A	G	missense_variant
17-36168002-36168003-T-C		1		Gm8909	Q90R	1	EYWERETRI	8	1	477.82	298.717	0.8	0.5	5.931	0	0	0	0.048	NoExpr	Pending	17	36168002	36168003	36168003	T	C	missense_variant
2-88025360-88025361-T-G		1	1	Olfr1161	L213R	1	RIILLSYMF	1	2	478.44	566.018	0.85	2.2	0	0	0	0	0.098	NoExpr	Pending	2	88025360	88025361	88025361	T	G	missense_variant
7-7174599-7174600-G-C		3		Zfp418	E38Q	1	VYFSQEQWE	7	3	478.92	1637.8	1.5	4.5	0.029	0	0	0	0.039	NoExpr	Pending	7	7174599	7174600	7174600	G	C	missense_variant
8-85171760-85171761-T-C		1	2	Cks1brt	L81P	1	HYMIHEPEPHI	9	3	479.34	495.119	1.2	1.5	0.012	0	0	0	0.057	NoExpr	Pending	8	85171760	85171761	85171761	T	C	missense_variant
7-107129167-107129168-G-A		2		Olfr715	T75I	1	SINIVPQAL	2	2	480.94	759.397	2.301	3.7	0	0	0	0	0.229	NoExpr	Pending	7	107129167	107129168	107129168	G	A	missense_variant
8-21734548-21734549-A-G		1		Defa24	I5V	1	KTLVLLSAL	4	1	482.07	685.899	1.6	2.2	0	0	0	0	0.088	NoExpr	Pending	8	21734548	21734549	21734549	A	G	missense_variant
4-88835957-88835958-A-T		2	1	Ifna5	K145I	1	SLLAVRIYF	7	3	482.07	1637.18	1.8	3.3	0	0	0	0	0.023	NoExpr	Pending	4	88835957	88835958	88835958	A	T	missense_variant
11-105798799-105798800-A-C		1		Tanc2	M263L	1	LYLLPRPNSV	4	1	482.07	442.1	0.35	0.24	0.938	0	0	0	0.345	NoExpr	Pending	11	105798799	105798800	105798800	A	C	missense_variant
13-67683431-67683432-C-A		1		Zfp738	V3F	1	EFGAGSSAI	2	1	483.16	1746.18	1	4.8	2.953	0	0	0	0.029	NoExpr	Pending	13	67683431	67683432	67683432	C	A	missense_variant
4-3172365-3172366-G-A		2		Vmn1r2	G95E	1	VYISRITREV	9	2	483.57	454.423	0.4	0.6	0	0	0	0	0.029	NoExpr	Pending	4	3172365	3172366	3172366	G	A	missense_variant
12-113597644-113597645-C-T		1		Ighv5-4	A52T	1	SYTMSWVRQ	3	1	484.27	384.671	2.4	1.7	0	0	0	0	0.038	NoExpr	Pending	12	113597644	113597645	113597645	C	T	missense_variant
2-87034020-87034021-C-G		2	3	Olfr1105	A67P	1	KYFFLTHLP	9	5	484.27	791.152	1.2	2.1	0	0	0	0	0.162	NoExpr	Pending	2	87034020	87034021	87034021	C	G	missense_variant
16-32755090-32755091-T-C		2	1	Muc4	I1655T	1	APITSTQML	6	2	485.39	478.729	2.301	2.2	0.018	0	0	0	0.03	NoExpr	Pending	16	32755090	32755091	32755091	T	C	missense_variant
16-32752920-32752921-T-C		2	1	Muc4	I933T	1	APITSTQML	6	2	485.39	478.729	2.301	2.2	0.018	0	0	0	0.048	NoExpr	Pending	16	32752920	32752921	32752921	T	C	missense_variant
13-89607947-89607948-A-C		1		Hapln1	K290N	1	AQIANVGQI	5	1	487.32	1358.6	1.3	3.8	0	0	0	0	0.25	NoExpr	Pending	13	89607947	89607948	89607948	A	C	missense_variant
10-89506605-89506606-T-C		1		Nr1h4	Q4R	1	RFQGLENPI	1	1	487.85	579.549	0.69	1.2	0.029	0	0	0	0.198	NoExpr	Pending	10	89506605	89506606	89506606	T	C	missense_variant
17-18597846-18597847-T-C		1		Vmn2r96	M562T	1	GYLCFTAL	6	1	490.3	2063.33	0.5	1.2	0	0	0	0	0.048	NoExpr	Pending	17	18597846	18597847	18597847	T	C	missense_variant
17-35321055-35321056-C-G		1		H2-Q1	S100R	1	RFQESLRTL	7	1	491.62	108.77	0.8	0.3	6.324	0	0	0	0.023	NoExpr	Pending	17	35321055	35321056	35321056	C	G	missense_variant
7-9986483-9986484-G-T		2		Vmn2r49	S360Y	1	NYEDSASNCK	2	2	493.08	5418.51	0.69	22	0	0	0	0	0.035	NoExpr	Pending	7	9986483	9986484	9986484	G	T	missense_variant
17-37590017-37590018-T-C		1		Olfr114	M112V	1	WFAWGEVAI	7	1	497.84	350.825	1.2	0.95	0	0	0	0	0.017	NoExpr	Pending	17	37590017	37590018	37590018	T	C	missense_variant
18-22920876-22920877-G-C		1		Nol4	P193A	1	DYNMAITMAYN	5	1	497.98	828.057	1.7	1.7	1.251	0	0	0	0.046	NoExpr	Pending	18	22920876	22920877	22920877	G	C	missense_variant
5-16302320-16302321-A-C		1	1	Cacna2d1	K356T	1	RANCNTIIM	6	2	498.88	431.519	1.3	1.7	7.938	0	0	0	0.131	NoExpr	Pending	5	16302320	16302321	16302321	A	C	missense_variant
17-20776350-20776351-C-T		1	1	Vmn1r228	A302T	1	WWVMNITTI	8	2	498.99	526.126	0.9	1.7	0	0	0	0	0.429	NoExpr	Pending	17	20776350	20776351	20776351	C	T	missense_variant
16-32753801-32753802-T-C		1	1	Muc4	I1226T	1	APITSTQIL	6	1	500.04	328.852	2	1.2	0.018	0	0	0	0.023	NoExpr	Pending	16	32753801	32753802	32753802	T	C	missense_variant

7-103517987-103517988-A-C		1		Olfr611	L132R	1	RHYHSILTDTV	1	1	500.85	433.221	0.4	0.5	0	0	0	0	0.19	NoExpr	Pending	7	103517987	103517988	103517988	A	C	missense_variant
7-103538837-103538838-A-C		1		Olfr612	L132R	1	RHYHSILTDTV	1	1	500.85	433.221	0.4	0.5	0	0	0	0	0.022	NoExpr	Pending	7	103538837	103538838	103538838	A	C	missense_variant
10-129691945-129691946-T-G		2	2	Olfr803	T32P	1	FVFLLIPYI	7	4	501.19	2765.86	2	4.2	0	0	0	0	0.038	NoExpr	Pending	10	129691945	129691946	129691946	T	G	missense_variant
7-45881541-45881542-T-A		1		Kdelr1	L132M	1	IYLESVAIM	9	1	502.45	338.135	1.2	0.7	127.19	0	0	0	0.225	NoExpr	Pending	7	45881541	45881542	45881542	T	A	missense_variant
1-182748902-182748903-G-C		2		Ccdc185	P74A	1	GYMTLARES	6	2	502.64	604.074	1.2	1.2	0	0	0	0	0.25	NoExpr	Pending	1	182748902	182748903	182748903	G	C	missense_variant
9-3359583-3359584-C-T		1		Alkbh8	T291I	1	RYLWIHGITPR	5	1	503.39	340.993	1	0.7	4.783	0	0	0	0.275	NoExpr	Pending	9	3359583	3359584	3359584	C	T	missense_variant
13-67474287-67474288-C-A		1	1	Zfp874b	R297M	1	SYLREHYRM	9	1	503.39	9862.8	2.6	20	3.445	0	0	0	0.085	NoExpr	Pending	13	67474287	67474288	67474288	C	A	missense_variant
X-64178266-64178267-T-C		2		3830417A13Rik	F276S	1	SYASIVRQD	1	2	503.61	1130.47	1.8	2.5	0.036	0	0	0	0.405	NoExpr	Pending	X	64178266	64178267	64178267	T	C	missense_variant
11-58529560-58529561-A-G		1		Olfr330	Y142H	1	RYPVLMNHRV	8	1	503.78	767.88	0.2	0.6	0	0	0	0	0.076	NoExpr	Pending	11	58529560	58529561	58529561	A	G	missense_variant
3-40721631-40721632-T-C		1	1	Slc25a31	S203P	1	PYFGAYDTV	1	2	503.85	29.75	0.5	0.2	0.07	0	0	0	0.284	NoExpr	Pending	3	40721631	40721632	40721632	T	C	missense_variant
7-111079319-111079320-T-G		1		Eif4g2	K108N	1	SNLILKGVI	2	1	504.77	863.495	1.6	2.6	264.25	0	0	0	0.274	NoExpr	Pending	7	111079319	111079320	111079320	T	G	missense_variant
5-15473119-15473120-G-T		1		Gm21149	L160I	1	SRLLMEENI	9	1	506.13	1476.59	1.4	3.8	0.032	0	0	0	0.051	NoExpr	Pending	5	15473119	15473120	15473120	G	T	missense_variant
9-44826937-44826938-T-G		1		Kmt2a	I1892L	1	LYLGQNEWTHV	3	1	507.52	398.44	0.71	0.58	2.708	0	0	0	0.039	NoExpr	Pending	9	44826937	44826938	44826938	T	G	missense_variant
5-110893009-110893010-T-C		1		Ttc28	L88S	1	SYSNRSAAYM	1	1	507.92	2555.26	0.71	2.8	0.995	0	0	0	0.217	NoExpr	Pending	5	110893009	110893010	110893010	T	C	missense_variant
12-115294364-115294365-A-G		1		Ighv8-8	S19P	1	AYVLPQVTL	5	1	508.27	154.202	1.2	0.5	0	0	0	0	0.041	NoExpr	Pending	12	115294364	115294365	115294365	A	G	missense_variant
17-35380445-35380446-C-A		1		H2-Q4	D168E	1	AYEGRDYI	3	1	509.96	1112.95	0.71	1.4	130.46	0	0	0	0.028	NoExpr	Pending	17	35380445	35380446	35380446	C	A,T	missense_variant
11-77472850-77472851-C-T		1		Ankrd13b	A429T	1	IFHILNTRI	7	1	510.49	1763.03	1.2	5.5	9.862	0	0	0	0.236	NoExpr	Pending	11	77472850	77472851	77472851	C	T	missense_variant
1-152843263-152843264-C-T		1		Smg7	G912D	1	LYPALLDPL	7	1	511.99	325.282	1.5	0.9	12.633	0	0	0	0.689	NoExpr	Pending	1	152843263	152843264	152843264	C	T	missense_variant
5-15529187-15529188-A-T		1		Gm21190	F11I	1	LFARLCRLI	9	1	514.15	9960.02	1.3	21	0.007	0	0	0	0.109	NoExpr	Pending	5	15529187	15529188	15529188	A	T	missense_variant
7-18890252-18890253-G-A		1		Pglyrp1	V154I	1	GFLRSNYEI	9	1	515.24	1534.27	1.6	7.3	4.665	0	0	0	0.666	NoExpr	Pending	7	18890252	18890253	18890253	G	A	missense_variant
17-23478397-23478398-G-A		2		Vmn2r117	H107Y	1	CYWKSKYNSF	2	2	516.91	1414.85	0.2	1.5	0	0	0	0	0.036	NoExpr	Pending	17	23478397	23478398	23478398	G	A	missense_variant
3-92418939-92418940-G-A		1		Sprr2j-ps	A41T	1	LLQSTQSPI	5	1	517.71	534.675	1.2	1.3	0	0	0	0	0.037	NoExpr	Pending	3	92418939	92418940	92418940	G	A	missense_variant
15-9589510-9589511-C-A		2		Spef2	A1556S	1	VYRALSVSV	8	2	518.82	647.93	2.199	2	0	0	0	0	0.033	NoExpr	Pending	15	9589510	9589511	9589511	C	A	missense_variant
12-114140887-114140889-CC-GA		1		Ighv9-3	G52S	1	GYTFTTYSM	8	1	518.91	1433	1.2	3.3	0	0	0	0	0.007	NoExpr	Pending	12	114140887	114140889	114140888	CC	GA	missense_variant
16-31127336-31127337-A-G		1		Acap2	F281L	1	GYLFKRASNAL	11	1	519.9	3704.33	0.7	2.1	13.735	0	0	0	0.269	NoExpr	Pending	16	31127336	31127337	31127337	A	G	missense_variant
6-128559068-128559069-T-C		1		A2ml1	H720R	1	SISRAKVAI	4	1	520	794.804	2.5	2.8	0	0	0	0	0.211	NoExpr	Pending	6	128559068	128559069	128559069	T	C	missense_variant
2-86508576-86508578-GG-AA		1	1	Olfr1076	LV39-40LM	1	YLITLMGNL	6	2	520	585.724	2.301	1.8	0	0	0	0	0.045	NoExpr	Pending	2	86508576	86508578	86508577	GG	AA	missense_variant
18-19987101-19987102-G-T		2		Dsc3	N166K	1	KYTVFYSIS	1	2	520.1	1327.67	2.199	3.7	0	0	0	0	0.031	NoExpr	Pending	18	19987101	19987102	19987102	G	T	missense_variant
6-136328830-136328831-C-T		1		Eif4a3l1	L97F	1	FSISVFQCL	6	1	522.5	524.916	1.7	1.7	0	0	0	0	0.344	NoExpr	Pending	6	136328830	136328831	136328831	C	T	missense_variant
10-129387749-129387750-G-T		1	1	Olfr784	G39V	1	NYILSLMVN	8	2	522.71	682.747	1.7	2.2	0	0	0	0	0.177	NoExpr	Pending	10	129387749	129387750	129387750	G	T	missense_variant
17-37280225-37280226-A-G		2		Olfr99	F65L	1	YFFLSQLSL	9	2	522.71	4221.77	1.7	6.1	0.593	0	0	0	0.025	NoExpr	Pending	17	37280225	37280226	37280226	A	G	missense_variant
2-49947546-49947547-C-T		1	1	Lypd6b	P177L	1	LYLLVLAWL	1	2	523.91	1274.27	1.2	1.7	0	0	0	0	0.386	NoExpr	Pending	2	49947546	49947547	49947547	C	T	missense_variant
4-111980303-111980304-G-T		1		Skint7	R93L	1	EYANLTEFV	5	1	525.73	1697.62	1	2.6	0	0	0	0	0.02	NoExpr	Pending	4	111980303	111980304	111980304	G	T	missense_variant
6-39522875-39522876-G-A		2		Dennd2a	P252S	1	RWYPPKSFI	7	2	528.56	3484.1	1.7	8.8	0.36	0	0	0	0.844	NoExpr	Pending	6	39522875	39522876	39522876	G	A	missense_variant
5-34236007-34236008-C-A		1		Zfyve28	A105S	1	SECLAAGSI	1	1	529.77	937.756	2.4	3.2	0.024	0	0	0	0.066	NoExpr	Pending	5	34236007	34236008	34236008	C	A	missense_variant
2-86181567-86181568-T-C		2		Olfr52	D181G	1	FYCDGMPLLRL	5	2	531.38	1145.59	1	2.1	0	0	0	0	0.246	NoExpr	Pending	2	86181567	86181568	86181568	T	C	missense_variant
13-62172975-62172976-T-C		1		Zfp808	V673A	1	KAFSQYSNL	2	1	531.38	375.868	1.2	0.93	4.296	0	0	0	0.306	NoExpr	Pending	13	62172975	62172976	62172976	T	C	missense_variant
1-85610881-85610883-GG-CG		1	1	Sp140	KE113-114NE	1	AYPDLNETL	6	2	532.07	565.98	1.6	1.2	54.864	0	0	0	0.203	NoExpr	Pending	1	85610881	85610883	85610882	GG	CG,TG,TC	missense_variant
11-77104174-77104175-A-C		1		Efcab5	M1190R	1	KRVKMIQNV	2	1	532.43	798.473	1.9	2.1	0.04	0	0	0	0.018	NoExpr	Pending	11	77104174	77104175	77104175	A	C	missense_variant
18-23569662-23569663-G-C		1		Dtna	V84L	1	RLEALLSTI	5	1	533.65	317.147	1.6	0.9	3.488	0	0	0	0.172	NoExpr	Pending	18	23569662	23569663	23569663	G	C	missense_variant
2-172551490-172551491-C-T		1		Tfap2c	P84S	1	LYSPAPSLS	7	1	534.68	4594.73	1.8	8.8	0	0	0	0	0.464	NoExpr	Pending	2	172551490	172551491	172551491	C	T	missense_variant
11-103455479-103455480-T-C		1		Lrrc37a	I3187V	1	SVTVVVTVL	5	1	535.91	1455.76	2.6	3.9	0	0	0	0	0.068	NoExpr	Pending	11	103455479	103455480	103455480	T	C	missense_variant
1-93024632-93024633-G-A		2		Kif1a	A1308V	1	AKLPVSRSI	5	2	536.12	1133.08	1.8	2.4	0.048	0	0	0	0.213	NoExpr	Pending	1	93024632	93024633	93024633	G	A	missense_variant
3-130631732-130631733-G-T		2		Etnppl	G396W	1	YEMKGKWVL	7	2	537.16	437.342	1.7	2.2	0	0	0	0	0.129	NoExpr	Pending	3	130631732	130631733	130631733	G	T	missense_variant
12-114538612-114538613-T-C		1		Ighv1-7	K78R	1	GYINPSSGYTR	11	1	537.16	326.498	1.4	0.8	0	0	0	0	0.137	NoExpr	Pending	12	114538612	114538613	114538613	T	C	missense_variant
2-89193310-89193311-G-T		2		Olfr1226	S241Y	1	GYHITVVL	2	2	537.16	23699.6	0.56	25	0	0	0	0	0.227	NoExpr	Pending	2	89193310	89193311	89193311	G	T	missense_variant
12-113736297-113736298-C-T		2		Ighv5-9-1	V65I	1	SYAMSWIRQ	7	2	539.62	384.671	2.5	1.7	0	0	0	0	0.055	NoExpr	Pending	12	113736297	113736298	113736298	C	T	missense_variant
10-82284746-82284747-C-G		1		4932415D10Rik	R4143T	1	IQQYSGTTL	8	1	539.83	1923.95	1.8	2.8	0	0	0	0	0.204	NoExpr	Pending	10	82284746	82284747	82284747	C	G	missense_variant
10-96041527-96041528-C-T		1		Eea1	T1368I	1	SCGKCFSVI	9	1	542.33	9338.13	2.199	24	22.462	0	0	0	0.027	NoExpr	Pending	10	96041527	96041528	96041528	C	T	missense_variant
10-51725419-51725420-C-T		1		Rfx6	P646S	1	SAIASRGSV	1	1	543	3724.69	1.5	9.1	0	0	0	0	0.551	NoExpr	Pending	10	51725419	51725420	51725420	C	T	missense_variant
13-23217383-23217384-G-T		2	1	Vmn1r221	K4N	1	NYINKIIFLFM	1	3	543	306.024	1	0.6	0	0	0	0	0.146	NoExpr	Pending	13	23217383	23217384	23217384	G	T	missense_variant
12-114416642-114416643-T-C		2		Ighv6-5	M85V	1	VYLQVNSLR	5	2	544.62	387.338	1.8	0.91	0	0	0	0	0.231	NoExpr	Pending	12	114416642	114416643	114416643	T	C	missense_variant
1-107271843-107271844-T-C		1		Serpinb3c	S316G	1	SGMGSTQGL	4	1	544.83	359.948	1.8	0.9	0	0	0	0	0.169	NoExpr	Pending	1	107271843	107271844	107271844	T	C	missense_variant
11-99350948-99350949-G-A		1		Krt27	A42V	1	SCVFGGSSL	3	1	545.87	275.48	2.199	1.1	0	0	0	0	0.335	NoExpr	Pending	11	99350948	99350949	99350949	G	A	missense_variant
1-85591681-85591682-G-T		1		Sp110	P142T	1	TQISLPSHL	1	1	548.39	1746.18	1.3	2.6	20.029	0	0	0	0.039	NoExpr	Pending	1	85591681	85591682	85591682	G	T	missense_variant
14-4558360-4558361-C-G		1		Gm3047	I194M	1	KNMCASSAK	3	1	548.61	392.726	2.1	1.5	0	0	0	0	0.098	NoExpr	Pending	14	4558360	4558361	4558361	C	G	missense_variant
7-39408114-39408115-G-C		1		Gm5114	H693Q	1	SQSAQPTTL	2	1	548.91	228.498	1.3	0.6	0	0	0	0	0.027	NoExpr	Pending	7	39408114	39408115	39408115	G	C	missense_variant
17-36167948-36167949-T-C		1		Gm8909	H108R	1	RYYNQSAGGSH	1	1	548.91	666.931	1.1	1.2	5.931	0	0	0	0.077	NoExpr	Pending	17	36167948	36167949	36167949	T	C	missense_variant
2-86162328-86162329-C-T		1	1	Olfr1043	V207M	1	LLSMAGFNM	9	2	548.91	3832	1.3	8.6	0	0	0	0	0.027	NoExpr	Pending	2	86162328	86162329	86162329	C	T	missense_variant
7-5480932-5480933-C-A		1		Vmn2r28	C756F	1	FYCVLGYLAFL	10	1	548.91	828.057	1.1	1.5	0	0	0	0	0.031	NoExpr	Pending	7	5480932	5480933	5480933	C	A	missense_variant
7-10158943-10158944-C-A		1		Vmn2r52	C756F	1	FYCVLGYLAFL	10	1	548.91	828.057	1.1	1.5	0	0	0	0	0.036	NoExpr	Pending	7	10158943	10158944	10158944	C	A	missense_variant
12-58212757-58212758-G-A		2		Sstr1	G56S	1	SSAILISFI	1	2	549.66	1718.26	1.1	2.3	0	0	0	0	0.454	NoExpr	Pending	12	58212757	58212758	58212758	G	A	missense_variant
8-79690418-79690419-T-G		1		Abce1	N321T	1	GYVPTETL	7	1	549.69	1319.98	0.58	1.7	74.352	0	0	0	0.212	NoExpr	Pending	8	79690418	79690419	79690419	T	G	missense_variant
2-62764576-62764577-C-T		1	1	Kcnh7	M716I	1	TYTNGIDINMV	8	2	550.44	552.981	1.3	1.3	0.005	0	0	0	0.404	NoExpr	Pending	2	62764576	62764577	62764577	C	T	missense_variant
1-171573843-171573844-T-A		1		Cd244a	S75R	1	VYNDGPRWSN	7	1	550.66	461.62	0.5	0.21	0.896	0	0	0	0.05	NoExpr	Pending	1	171573843	171573844	171573844	T	A	missense_variant

1-161060750-161060751-G-A		1	1	Dars2	L200F	1	MVMKMREYF	9	2	552.08	774.462	2.199	2.9	12.725	0	0	0	0.617	NoExpr	Pending	1	161060750	161060751	161060751	G	A	missense_variant
7-103506429-103506430-G-A		1	1	Olfr610	P172L	1	LLGRLTFQL	9	2	552.08	8912.51	2.199	18	0	0	0	0	0.296	NoExpr	Pending	7	103506429	103506430	103506430	G	A	missense_variant
1-85484483-85484484-C-T		1		AC147806.1	R220Q	1	SNIKAQRTV	6	1	552.41	380.417	1.7	1.1	0	0	0	0	0.012	NoExpr	Pending	1	85484483	85484484	85484484	C	T	missense_variant
1-84964326-84964327-G-A		1		AC167036.1	R220Q	1	SNIKAQRTV	6	1	552.41	380.417	1.7	1.1	0	0	0	0	0.021	NoExpr	Pending	1	84964326	84964327	84964327	G	A	missense_variant
7-85150570-85150571-A-C		1	1	Vmn2r67	H486Q	1	LPSQQQLYM	4	2	553.13	794.017	2.199	2.9	0	0	0	0	0.255	NoExpr	Pending	7	85150570	85150571	85150571	A	C	missense_variant
7-85410257-85410258-T-C		1		Vmn2r69	E481G	1	GHLPSGQQL	1	1	556.6	1714.31	1.5	3	0	0	0	0	0.044	NoExpr	Pending	7	85410257	85410258	85410258	T	C	missense_variant
12-113879201-113879202-G-A		3		Ighv2-9	L82F	1	NYNSAFMSRL	6	3	559.13	510.3	0.55	0.54	0	0	0	0	0.015	NoExpr	Pending	12	113879201	113879202	113879202	G	A	missense_variant
15-80918576-80918577-A-C		1	1	Tnrc6b	N1527T	1	PYSASDTSF	7	2	559.86	964.029	1.8	2.7	2.842	0	0	0	0.048	NoExpr	Pending	15	80918576	80918577	80918577	A	C	missense_variant
1-171530501-171530502-G-C		3		Itln1	S226C	1	YYSPCGQRE	5	3	560.09	136.855	1.9	0.5	0.383	0	0	0	0.061	NoExpr	Pending	1	171530501	171530502	171530502	G	C,T	missense_variant
18-36004746-36004747-C-T		1		Psd2	A464V	1	KNEKLEWVI	8	1	560.92	437.342	2.801	2.2	0.01	0	0	0	0.06	NoExpr	Pending	18	36004746	36004747	36004747	C	T	missense_variant
13-92131447-92131448-G-A		1		Rasgrf2	A16V	1	RYNEGHALYL\	11	1	560.92	2289.62	1.1	3.7	0.063	0	0	0	0.737	NoExpr	Pending	13	92131447	92131448	92131448	G	A	missense_variant
1-159324957-159324958-C-G		1		Cop1	P593A	1	YYDLRNTKQAI	10	1	562.34	340.97	0.54	0.4	20.172	0	0	0	0.132	NoExpr	Pending	1	159324957	159324958	159324958	C	G	missense_variant
1-46680779-46680780-T-G		1		Dnah7c	V2797G	1	WGIAMDSYD	2	1	563.75	779.992	1.8	2.4	0.066	0	0	0	0.028	NoExpr	Pending	1	46680779	46680780	46680780	T	G	missense_variant
2-88794939-88794940-C-T		1	2	Olfr1201	A186V	1	QPLLKLVCM	7	3	564.72	1209.27	1.2	2.5	0	0	0	0	0.384	NoExpr	Pending	2	88794939	88794940	88794940	C	T	missense_variant
1-85099520-85099521-A-G		1		A530032D15Rik	V108A	1	AYPDLKETL	1	1	565.98	1112.4	1.2	1.6	13.981	0	0	0	0.076	NoExpr	Pending	1	85099520	85099521	85099521	A	G	missense_variant
10-34403686-34403687-C-T		1		Nt5dc1	D120N	1	SNKPGVSDI	2	1	566.36	1032.98	2.4	4.2	7.435	0	0	0	0.542	NoExpr	Pending	10	34403686	34403687	34403687	C	T	missense_variant
1-85484922-85484923-C-T		1		AC147806.1	V74I	1	SYCGLGVI	8	1	568.33	1617.47	0.76	1.9	0	0	0	0	0.028	NoExpr	Pending	1	85484922	85484923	85484923	C	T	missense_variant
12-32205628-32205629-C-T		2		Pik3cg	D120N	1	RYQVVQTLNCI	9	2	571.1	461.61	0.6	0.64	1.836	0	0	0	0.388	NoExpr	Pending	12	32205628	32205629	32205629	C	T	missense_variant
7-8368345-8368346-G-C		1		Vmn2r44	T567R	1	QYANREQNKC	5	1	571.32	711.017	1.6	1	0	0	0	0	0.038	NoExpr	Pending	7	8368345	8368346	8368346	G	C	missense_variant
10-79194418-79194419-A-T		1	1	Vmn2r80	K693M	1	APGRMIRWLL	5	2	572.89	1111.65	0.6	1	0	0	0	0	0.03	NoExpr	Pending	10	79194418	79194419	79194419	A	T	missense_variant
9-119932693-119932694-C-T		1	1	Gorasp1	G91D	1	SNMWGDQGL	6	2	573.19	567.017	2.9	1.8	5.246	0	0	0	0.255	NoExpr	Pending	9	119932693	119932694	119932694	C	T	missense_variant
17-43087957-43087958-C-T		1		Tnfrsf21	P652S	1	VYSHLSDLL	6	1	574.24	615.304	1.9	2	2.877	0	0	0	0.387	NoExpr	Pending	17	43087957	43087958	43087958	C	T	missense_variant
X-74303850-74303851-G-A		1		Atp6ap1	G413S	1	SYASDCASF	8	1	575.34	1535.54	2.1	4.2	115.79	0	0	0	0.038	NoExpr	Pending	X	74303850	74303851	74303851	G	A	missense_variant
17-69287624-69287625-C-A		1		Epb41l3	A898E	1	HDQALAQEI	8	1	575.56	264.296	1.9	0.8	0.167	0	0	0	0.054	NoExpr	Pending	17	69287624	69287625	69287625	C	A	missense_variant
7-23984786-23984787-G-A		1		Vmn1r181	D226N	1	QYIFTLNQN	9	1	575.56	771.063	1.9	2.3	0	0	0	0	0.023	NoExpr	Pending	7	23984786	23984787	23984787	G	A	missense_variant
15-89190248-89190249-C-G		2		Dennd6b	A122P	1	SYNNKAPLPL	9	2	576.23	772.64	0.8	0.99	0.495	0	0	0	0.041	NoExpr	Pending	15	89190248	89190249	89190249	C	G	missense_variant
17-37590035-37590036-A-C		1	1	Olfr114	W106G	1	GFAWGEMAI	1	2	576.89	350.825	1.2	0.95	0	0	0	0	0.015	NoExpr	Pending	17	37590035	37590036	37590036	A	C	missense_variant
7-100878819-100878820-C-G		2		Arhgef17	G1738A	1	AYQSSVWLA	9	2	577.11	751.779	1.5	1.4	4.56	0	0	0	0.181	NoExpr	Pending	7	100878819	100878820	100878820	C	G	missense_variant
18-78109935-78109936-T-G		1	1	Slc14a1	I313L	1	LHAALGSLL	5	2	577.11	1023.51	1.7	1.7	39.08	0	0	0	0.087	NoExpr	Pending	18	78109935	78109936	78109936	T	G	missense_variant
7-86171680-86171681-T-C		1		Vmn2r75	K15R	1	FWFLRISII	5	1	578.44	1633.37	1.5	3	0	0	0	0	0.099	NoExpr	Pending	7	86171680	86171681	86171681	T	C	missense_variant
14-67724570-67724571-A-G		1		Kctd9	Y21C	1	KVVAVCGTL	6	1	579.42	579.421	1.4	2	19.062	0	0	0	0.033	NoExpr	Pending	14	67724570	67724571	67724571	A	G	missense_variant
4-24596142-24596143-C-T		1		Mms22l	S1074F	1	FYLEFKGSSPF	1	1	579.42	611.631	1.1	0.7	8.676	0	0	0	0.327	NoExpr	Pending	4	24596142	24596143	24596143	C	T	missense_variant
4-131799653-131799654-A-G		2		Ptpru	L669P	1	HYFGAEPA	7	2	579.42	855.376	0.9	1.2	0.012	0	0	0	0.207	NoExpr	Pending	4	131799653	131799654	131799654	A	G	missense_variant
14-53105270-53105271-G-A		1		Trav6n-5	A89T	1	TYNKETTSFHL	6	1	579.42	537.157	1.1	1	0	0	0	0	0.023	NoExpr	Pending	14	53105270	53105271	53105271	G	A	missense_variant
14-53133063-53133064-G-A		1		Trav6n-6	A91T	1	TYNKETTSFHL	6	1	579.42	537.157	1.1	1	0	0	0	0	0.109	NoExpr	Pending	14	53133063	53133064	53133064	G	A	missense_variant
8-125447857-125447858-C-T		1		Sipa1l2	S1227N	1	SSHSSNNTL	6	1	580.89	587.611	0.96	0.9	0.639	0	0	0	0.123	NoExpr	Pending	8	125447857	125447858	125447858	C	T	missense_variant
10-27459393-27459394-A-C		1		Lama2	I95S	1	QRHPSTNAI	5	1	581.11	2052.39	1.9	3.8	0.191	0	0	0	0.04	NoExpr	Pending	10	27459393	27459394	27459394	A	C	missense_variant
2-129609071-129609072-G-A		1		Sirpa	G84E	1	IYSFAEEYV	6	1	581.92	338.8	0.77	0.51	19.421	0	0	0	0.393	NoExpr	Pending	2	129609071	129609072	129609072	G	A	missense_variant
7-65663890-65663891-G-A		1		Tarsl2	E353K	1	TYWKGNPEM	4	1	585.18	1059.65	2	2.4	6.741	0	0	0	0.582	NoExpr	Pending	7	65663890	65663891	65663891	G	A	missense_variant
2-180191286-180191287-G-A		3		Lama5	H1585Y	1	CYEAGTMAS	2	3	585.72	9346.08	1.9	14	22.128	0	0	0	0.085	NoExpr	Pending	2	180191286	180191287	180191287	G	A	missense_variant
2-130041822-130041823-G-C		1		Tgm3	G467A	1	SFAATSSRN	3	1	585.72	1485.27	3	7.1	0.013	0	0	0	0.11	NoExpr	Pending	2	130041822	130041823	130041823	G	C	missense_variant
17-23345903-23345904-A-G		1		Vmn2r115	K255R	1	LYMSRAEV	5	1	587.58	691.93	0.6	0.7	0	0	0	0	0.052	NoExpr	Pending	17	23345903	23345904	23345904	A	G	missense_variant
11-98250138-98250139-C-T		1		Cdk12	T1402I	1	RFIRVPLAL	3	1	587.96	1239.3	1	2.5	4.609	0	0	0	0.051	NoExpr	Pending	11	98250138	98250139	98250139	C	T	missense_variant
X-104565824-104565825-G-C		2		Zdhhc15	P205A	1	KYWRGELASV	8	2	588.36	767.658	0.4	1.1	0.361	0	0	0	0.28	NoExpr	Pending	X	104565824	104565825	104565825	G	C	missense_variant
1-171573842-171573843-G-T		1	1	Cd244a	S75I	1	WYNDGPIWSNV	7	2	590.04	461.62	0.6	0.21	0.896	0	0	0	0.132	NoExpr	Pending	1	171573842	171573843	171573843	G	T	missense_variant
5-121572793-121572794-C-G		1		Aldh2	G324A	1	CCAASRTFV	4	1	591.68	1099.23	1.7	3.1	107.76	0	0	0	0.073	NoExpr	Pending	5	121572793	121572794	121572794	C	G	missense_variant
17-23291036-23291037-C-T		1		Vmn2r114	G823E	1	SSAGMLECI	7	1	591.68	411.235	1.9	1.4	0	0	0	0	0.064	NoExpr	Pending	17	23291036	23291037	23291037	C	T	missense_variant
17-23360024-23360025-G-A		1		Vmn2r115	G824E	1	SSAGMLECI	7	1	591.68	411.235	1.9	1.4	0	0	0	0	0.02	NoExpr	Pending	17	23360024	23360025	23360025	G	A	missense_variant
17-23401760-23401761-G-A		1		Vmn2r116	G823E	1	SSAGMLECI	7	1	591.68	411.235	1.9	1.4	0	0	0	0	0.023	NoExpr	Pending	17	23401760	23401761	23401761	G	A	missense_variant
10-128886413-128886414-C-T		1	1	Gdf11	V191I	1	RPVPRPATIYL	9	2	592.1	884.75	0.42	0.6	0.381	0	0	0	0.2	NoExpr	Pending	10	128886413	128886414	128886414	C	T	missense_variant
9-3024530-3024531-C-G		2	2	Gm10718	L172V	1	IFHVVQWTF	5	4	592.1	494.117	1.8	1.7	0	0	0	0	0.028	NoExpr	Pending	9	3024530	3024531	3024531	C	G	missense_variant
2-157269529-157269530-A-G		1		Mroh8	I168T	1	STERLVQSI	2	1	592.1	359.948	1.9	1.8	0.081	0	0	0	0.046	NoExpr	Pending	2	157269529	157269530	157269530	A	G	missense_variant
4-113931810-113931811-C-A		2		Skint5	S289I	1	KIIQSSSQS	2	2	592.1	902.926	3	4.5	0	0	0	0	0.025	NoExpr	Pending	4	113931810	113931811	113931811	C	A	missense_variant
17-25242587-25242588-C-T		1		Tsr3	T300I	1	SNYSGAEEI	9	1	594.42	9751.92	1.3	15	30.598	0	0	0	0.052	NoExpr	Pending	17	25242587	25242588	25242588	C	T	missense_variant
4-146466498-146466499-G-C		1	1	Zfp992	D226H	1	IHLSIHQGI	2	2	594.42	2994.06	1.9	4.8	0.548	0	0	0	0.5	NoExpr	Pending	4	146466498	146466499	146466499	G	C	missense_variant
3-30659084-30659085-G-A		2		Lrriq4	R445Q	1	LYIENNQLEQL	7	2	595.81	548.125	0.23	0.3	0	0	0	0	0.278	NoExpr	Pending	3	30659084	30659085	30659085	G	A	missense_variant
18-36962252-36962253-C-T		1	2	Pcdha5	S605F	1	YNAWLFYEL	6	3	597.04	1042.32	2.199	3.2	0	0	0	0	0.029	NoExpr	Pending	18	36962252	36962253	36962253	C	T	missense_variant
6-32163415-32163416-C-T		2		Plxna4	R1679Q	1	SEIYLTQLL	7	2	597.16	1371.17	1.7	3.8	0.039	0	0	0	0.85	NoExpr	Pending	6	32163415	32163416	32163416	C	T	missense_variant
12-113306433-113306434-T-G		1	1	Ighg2b	K322N	1	HEGLNNYYL	5	2	598.18	643.917	2.4	2.5	0	0	0	0	0.012	NoExpr	Pending	12	113306433	113306434	113306434	T	G	missense_variant
9-5321499-5321500-C-T		1		Casp4	H84Y	1	SYHGEANL	2	1	598.54	9338.1	0.38	4.1	39.813	0	0	0	0.232	NoExpr	Pending	9	5321499	5321500	5321500	C	T	missense_variant
12-115208513-115208514-C-T		2		Ighv1-55	C5Y	1	SYIILILVAAA	2	2	598.54	11604	2	20	0	0	0	0	0.01	NoExpr	Pending	12	115208513	115208514	115208514	C	T	missense_variant
17-23291793-23291794-A-G		2		Vmn2r114	C571R	1	QYANTEQNHR	10	2	603.34	788.642	0.8	1.2	0	0	0	0	0.043	NoExpr	Pending	17	23291793	23291794	23291794	A	G	missense_variant
12-115593233-115593235-TT-CT		1		Ighv1-66	N76S	1	IYPGSGSTK	7	1	604.31	1589.5	1.9	3.6	0	0	0	0	0.221	NoExpr	Pending	12	115593233	115593235	115593234	TT	CT,CC	missense_variant
12-113879112-113879113-C-T		1		Ighv2-9	M111I	1	SLQTDDTAI	9	1	605.05	2192.64	1.8	5.6	0	0	0	0	0.034	NoExpr	Pending	12	113879112	113879113	113879113	C	T	missense_variant
5-138988374-138988375-A-G		1		Pdgfa	V80A	1	APIRRKRSI	1	1	605.05	1210.85	3	3.4	20.706	0	0	0	0.097	NoExpr	Pending	5	138988374	138988375	138988375	A	G	missense_variant

7-7312836-7312837-C-A		1	1	Vmn2r30	V666F	1	IVFTVAFST	7	2	606.74	15922.1	2.801	22	0	0	0	0	0.3	NoExpr	Pending	7	7312836	7312837	7312837	C	A	missense_variant
7-86164094-86164095-C-T		1		Vmn2r75	E500K	1	KWATDMDQI	1	1	607.1	5834.44	2	13	0	0	0	0	0.586	NoExpr	Pending	7	86164094	86164095	86164095	C	T	missense_variant
16-32754576-32754577-T-A		2		Muc4	S1484T	1	HMLTSTSSTQI	4	2	607.97	1161.4	0.91	1.5	0.018	0	0	0	0.007	NoExpr	Pending	16	32754576	32754577	32754577	T	A	missense_variant
2-90052006-90052007-C-T		1		Olfr140	G106R	1	AHLLRGTEI	5	1	608.96	267.01	2.5	1.2	0	0	0	0	0.514	NoExpr	Pending	2	90052006	90052007	90052007	C	T	missense_variant
6-57404710-57404711-A-G		1		Vmn1r19	N83S	1	FYISRVMRGL	4	1	609.68	799.89	0.72	0.93	0	0	0	0	0.221	NoExpr	Pending	6	57404710	57404711	57404711	A	G	missense_variant
4-28947572-28947573-A-G		2		Epha7	E615G	1	TYIDPETYGDP	9	2	611.63	767.658	1.2	1.4	0	0	0	0	0.261	NoExpr	Pending	4	28947572	28947573	28947573	A	G	missense_variant
16-45442598-45442599-G-A		1		Gm609	A172V	1	EYFVQNANATV	4	1	613.59	1804.21	1	0.91	0	0	0	0	0.074	NoExpr	Pending	16	45442598	45442599	45442599	G	A	missense_variant
13-67671481-67671482-C-A		1	1	Zfp738	S130I	1	EECAKSFHI	9	2	613.76	2570.4	2.699	9.5	2.953	0	0	0	0.02	NoExpr	Pending	13	67671481	67671482	67671482	C	A	missense_variant
11-73453653-73453654-T-A		1		Olfr380	K186M	1	LLMLACSDI	3	1	614.13	4179.17	2	8	0	0	0	0	0.3	NoExpr	Pending	11	73453653	73453654	73453654	T	A	missense_variant
7-141639453-141639454-C-T		2		Muc6	A1769T	1	PVIYTTSTI	8	2	616.72	650.264	1.8	2.1	0.024	0	0	0	0.059	NoExpr	Pending	7	141639453	141639454	141639454	C	T	missense_variant
1-6810623-6810624-A-G		1		St18	D447G	1	AMTQGKSQL	5	1	616.72	2168.15	2	5.8	0.045	0	0	0	0.198	NoExpr	Pending	1	6810623	6810624	6810624	A	G	missense_variant
12-118931855-118931856-C-T		3		Abcb5	A402T	1	SYPSRPSTKV	8	3	617.55	1223.6	0.5	1.4	0	0	0	0	0.418	NoExpr	Pending	12	118931855	118931856	118931856	C	T	missense_variant
1-166269457-166269458-G-A		1		Ildr2	G82E	1	SYCQDRMEESI	8	1	617.74	254.608	0.5	0.6	0.298	0	0	0	0.684	NoExpr	Pending	1	166269457	166269458	166269458	G	A	missense_variant
X-74303922-74303923-C-G		1		Atp6ap1	L437V	1	IFTYGVHMI	6	1	618.14	616.723	1.2	1.2	115.79	0	0	0	0.054	NoExpr	Pending	X	74303922	74303923	74303923	C	G	missense_variant
3-107213457-107213458-G-A		1		Cym	T277I	1	CPAVLDTGI	9	1	618.28	9185.23	3.1	29	0	0	0	0	0.277	NoExpr	Pending	3	107213457	107213458	107213458	G	A	missense_variant
7-7476901-7476902-G-C		1		Vmn2r32	L91V	1	RYPDVLPNMSL	5	1	618.28	883.597	0.7	0.4	0	0	0	0	0.066	NoExpr	Pending	7	7476901	7476902	7476902	G	C	missense_variant
2-66508624-66508625-T-A		1		Scn9a	K1172N	1	GNGKVWWTI	2	1	622.43	1202.98	2	3.4	0	0	0	0	0.203	NoExpr	Pending	2	66508624	66508625	66508625	T	A	missense_variant
18-80130096-80130097-G-C		1		Adnp2	P366A	1	LNQAVNTAV	4	1	623.86	822.413	1.6	1.7	6.82	0	0	0	0.04	NoExpr	Pending	18	80130096	80130097	80130097	G	C	missense_variant
3-90349472-90349473-A-T		1		Gatad2b	N196I	1	KEIVVQKTPV	3	1	625.01	1989.19	1	3.6	12.087	0	0	0	0.127	NoExpr	Pending	3	90349472	90349473	90349473	A	T	missense_variant
8-71689356-71689357-G-A		1		Insl3	V34M	1	GHHLMRTLV	5	1	625.01	1217.78	3.1	4.6	0.001	0	0	0	0.545	NoExpr	Pending	8	71689356	71689357	71689357	G	A	missense_variant
12-103895049-103895050-G-T		1	1	Serpina1c	L402I	1	EEHTQSPIF	8	2	625.01	598.537	3	2.9	0.386	0	0	0	0.073	NoExpr	Pending	12	103895049	103895050	103895050	G	T	missense_variant
6-40571909-40571910-G-A		1	1	Olfr460	D175N	1	NYCKSNVVN	9	2	625.3	837.703	2.801	3.2	0	0	0	0	0.048	NoExpr	Pending	6	40571909	40571910	40571910	G	A	missense_variant
15-66541676-66541677-C-G		3		Tmem71	G182A	1	AYILPQSLR	1	3	626.74	1167.51	2	3.3	1.769	0	0	0	0.065	NoExpr	Pending	15	66541676	66541677	66541677	C	G	missense_variant
12-114682827-114682828-T-G		1		Ighv1-18	N52T	1	GYTFTDYTM	8	1	626.99	1120.11	0.87	2.6	0	0	0	0	0.032	NoExpr	Pending	12	114682827	114682828	114682828	T	G	missense_variant
2-3462551-3462552-C-A		3		Suv39h2	D376Y	1	YYESDEFTV	1	3	626.99	7468.96	2	13	4.408	0	0	0	0.472	NoExpr	Pending	2	3462551	3462552	3462552	C	A	missense_variant
2-98667242-98667243-T-A		1		Gm10800	D20V	1	CHISRPTVV	9	1	629.72	5165.23	2.9	12	0.566	0	0	0	0.012	NoExpr	Pending	2	98667242	98667243	98667243	T	A	missense_variant
15-4912795-4912796-C-T		2		Mroh2b	H347Y	1	NYTTSIEKTVK	2	2	631.81	6591.36	1.4	18	0	0	0	0	0.037	NoExpr	Pending	15	4912795	4912796	4912796	C	T	missense_variant
16-32754966-32754967-C-G		1		Muc4	Q1614E	1	LSTASSTEI	8	1	632.54	400.95	2	1.3	0.018	0	0	0	0.021	NoExpr	Pending	16	32754966	32754967	32754967	C	G	missense_variant
12-103691891-103691892-C-G		1		Serpina1f	E251D	1	HYLFRVDDL	7	1	632.79	743.464	2.1	2	0	0	0	0	0.164	NoExpr	Pending	12	103691891	103691892	103691892	C	G	missense_variant
1-85610748-85610749-A-G		2		Sp140	E69G	1	EYQGTCKNL	4	2	632.79	748.618	1	1.2	54.864	0	0	0	0.059	NoExpr	Pending	1	85610748	85610749	85610749	A	G	missense_variant
13-100161675-100161677-TG-GA		4	1	Naip2	S617F	1	YLFRTCLLI	3	5	634.25	1125.28	2.1	3.2	8.368	0	0	0	0.053	NoExpr	Pending	13	100161675	100161677	100161676	TG	GA	missense_variant
11-46336437-46336438-A-C		1		Itk	F459V	1	DYLRSQRGLV	10	1	634.81	9402.78	0.9	8.5	0.171	0	0	0	0.04	NoExpr	Pending	11	46336437	46336438	46336438	A	C	missense_variant
8-123208024-123208025-C-T		1		Chmp1a	R64Q	1	NWLQMASRV	4	1	635.71	661.03	1.3	0.9	50.203	0	0	0	0.036	NoExpr	Pending	8	123208024	123208025	123208025	C	T	missense_variant
7-108613041-108613042-C-A		1		Olfr506	T245N	1	AFSTCNSHL	6	1	637.18	881.231	2.1	2.6	0	0	0	0	0.026	NoExpr	Pending	7	108613041	108613042	108613042	C	A	missense_variant
16-44419262-44419263-G-A		2	1	Cfap44	V558I	1	AELHLKYIF	8	3	638.01	1806.47	2.4	3	0	0	0	0	0.561	NoExpr	Pending	16	44419262	44419263	44419263	G	A	missense_variant
10-83504132-83504133-G-C		1		Aldh1l2	P584A	1	KEALGACAI	3	1	638.4	5903.23	2.801	14	20.285	0	0	0	0.04	NoExpr	Pending	10	83504132	83504133	83504133	G	C	missense_variant
6-42538470-42538471-C-T		2		Olfr455	D184N	1	HFFCNITAL	5	2	638.4	1309.84	1.1	2.2	0	0	0	0	0.044	NoExpr	Pending	6	42538470	42538471	42538471	C	T	missense_variant
10-22371586-22371587-T-A		1		Raet1d	S187R	1	CRQKMDEFL	2	1	638.4	1227.69	2.1	3.4	0.357	0	0	0	0.332	NoExpr	Pending	10	22371586	22371587	22371587	T	A	missense_variant
7-5125843-5125844-C-T		1		Rasl2-9	R29H	1	KTTFMKHHL	7	1	638.4	937.756	2.699	4.1	0.236	0	0	0	0.046	NoExpr	Pending	7	5125843	5125844	5125844	C	T	missense_variant
3-87938679-87938680-G-A		1		Isg20l2	R300Q	1	SLTRDTSQI	8	1	638.68	984.564	2	3	21.131	0	0	0	0.039	NoExpr	Pending	3	87938679	87938680	87938680	G	A	missense_variant
6-73044834-73044835-C-A		1		Dnah6	E3501D	1	FVIDNLGKQFI	4	1	639.56	833.451	2.6	3	0.032	0	0	0	0.25	NoExpr	Pending	6	73044834	73044835	73044835	C	A	missense_variant
17-23387403-23387404-G-C		1		Vmn2r116	S430T	1	NYTCKKLYSYL	3	1	639.73	854.25	0.71	1.2	0	0	0	0	0.135	NoExpr	Pending	17	23387403	23387404	23387404	G	C	missense_variant
16-88962819-88962820-T-C		1		Krtap16-3	S2G	1	GYYSGYSGG	1	1	641.34	205.159	2.1	0.6	0	0	0	0	0.058	NoExpr	Pending	16	88962819	88962820	88962820	T	C	missense_variant
1-105678183-105678184-C-T		1		Relch	T183I	1	NRAGSISIL	8	1	642.82	298.717	2.1	0.8	8.032	0	0	0	0.183	NoExpr	Pending	1	105678183	105678184	105678184	C	T	missense_variant
12-115242907-115242908-T-A		2		Ighv1-56	K82M	1	YYNEMFKGK	5	2	643.07	14330.5	2.1	17	0	0	0	0	0.021	NoExpr	Pending	12	115242907	115242908	115242908	T	A	missense_variant
X-152612667-152612668-T-G		1		Shroom2	N1442H	1	TYLSEEHL	7	1	645.41	763.133	0.5	0.7	0.37	0	0	0	0.741	NoExpr	Pending	X	152612667	152612668	152612668	T	G	missense_variant
6-131678761-131678762-G-A		1	1	Tas2r106	T42I	1	KFSIAGFIL	4	2	645.63	688.934	2.4	2.4	0	0	0	0	0.231	NoExpr	Pending	6	131678761	131678762	131678762	G	A	missense_variant
14-20262885-20262886-A-G		1		Kcnk16	V250A	1	AWLAVALSL	6	1	646.04	845.785	2.1	2.5	0	0	0	0	0.051	NoExpr	Pending	14	20262885	20262886	20262886	A	G	missense_variant
4-146893819-146893820-C-T		1		Gm21411	C29Y	1	EYLSFAQRTL	2	1	646.34	8297.38	0.7	15	0	0	0	0	0.018	NoExpr	Pending	4	146893819	146893820	146893820	C	T	missense_variant
7-12362283-12362284-C-T		1		Vmn1r84	D161N	1	TAINVIGPNTI	4	1	646.96	760.116	2.6	3.4	0	0	0	0	0.246	NoExpr	Pending	7	12362283	12362284	12362284	C	T	missense_variant
2-111983151-111983152-A-T		1		Olfr1309	H315Q	1	GYLRQYRKM	5	1	650.04	1527.88	0.85	4.2	0	0	0	0	0.09	NoExpr	Pending	2	111983151	111983152	111983152	A	T	missense_variant
7-102742199-102742200-C-T		1		Olfr78	D268N	1	RFGNSLNPI	7	1	650.05	1734.67	0.85	2	0	0	0	0	0.235	NoExpr	Pending	7	102742199	102742200	102742200	C	T	missense_variant
17-23477454-23477456-TG-CA		1		Vmn2r117	H326C	1	AFGQCHSEI	5	1	651	760.782	1.7	2.8	0	0	0	0	0.077	NoExpr	Pending	17	23477454	23477456	23477455	TG	CA	missense_variant
9-38524216-38524217-T-A		1		Olfr911-ps1	C162S	1	AMIHTGSIL	7	1	652.02	3713.06	1.5	5.9	0	0	0	0	0.025	NoExpr	Pending	9	38524216	38524217	38524217	T	A	missense_variant
4-147678235-147678237-GT-TA		1		Zfp534	Y46L	1	LYMDVMLENL	10	1	652.08	3440.65	0.7	4.6	0.043	0	0	0	0.091	NoExpr	Pending	4	147678235	147678237	147678236	GT	TA	missense_variant
4-145534921-145534923-AC-TA		1		Zfp990	Y46L	1	LYMDVMLENL	10	1	652.08	3440.65	0.7	4.6	0.034	0	0	0	0.071	NoExpr	Pending	4	145534921	145534923	145534922	AC	TA	missense_variant
4-84276138-84276139-C-G		1		Bnc2	G916A	1	SDAASDEGI	4	1	652.65	1009.86	3.301	3.5	0.177	0	0	0	0.186	NoExpr	Pending	4	84276138	84276139	84276139	C	G	missense_variant
4-96630745-96630746-A-T		1		Cyp2j5	F431Y	1	QYKKRESFL	2	1	654.77	5957.86	0.8	7.8	0	0	0	0	0.061	NoExpr	Pending	4	96630745	96630746	96630746	A	T	missense_variant
4-115532666-115532667-G-T		1		Cyp4a10	G500W	1	LVLKSKNWI	8	1	654.77	703.218	2.1	3.1	0	0	0	0	0.025	NoExpr	Pending	4	115532666	115532667	115532667	G	T	missense_variant
6-70518726-70518727-A-C		2		Igkv3-12	E79D	1	IYLASNLDS	8	2	656.28	641.342	2.1	1.7	0	0	0	0	0.192	NoExpr	Pending	6	70518726	70518727	70518727	A	C	missense_variant
16-29629654-29629655-G-C		1		Opa1	W899C	1	CRIQRMLAI	1	1	657.79	861.172	2.1	2.5	14.081	0	0	0	0.1	NoExpr	Pending	16	29629654	29629655	29629655	G	C	missense_variant
12-112735255-112735256-C-T		1	1	Cep170b	P190S	1	SYSERPKDL	1	2	659.31	6824.8	1.9	11	5.729	0	0	0	0.345	NoExpr	Pending	12	112735255	112735256	112735256	C	T	missense_variant
7-40993461-40993462-C-G		1		4930433I11Rik	T185S	1	QYQDSTVSESK	8	1	659.75	659.754	1.2	1.2	0	0	0	0	0.057	NoExpr	Pending	7	40993461	40993462	40993462	C	G	missense_variant
11-69649177-69649178-G-A		1		Fxr2	S287N	1	NYLEFSEDSV	1	1	659.75	315.108	0.28	0.2	28.581	0	0	0	0.577	NoExpr	Pending	11	69649177	69649178	69649178	G	A	missense_variant
10-30647991-30647992-T-G		1		Ncoa7	S891R	1	SYFINGDIRSL	9	1	660.25	292.51	1.4	0.45	6.872	0	0	0	0.283	NoExpr	Pending	10	30647991	30647992	30647992	T	G	missense_variant

6-56957122-56957123-G-A		1		Vmn1r4	R204K	1	KHQRQCKHL	1	1	661.09	1566.27	1.8	4.8	0.002	0	0	0	0.02	NoExpr	Pending	6	56957122	56957123	56957123	G	A	missense_variant
3-62419694-62419695-A-G		1	1	Arhgef26	K543R	1	RLLATNPSF	1	2	663.48	1131.96	2.5	3.6	0.278	0	0	0	0.125	NoExpr	Pending	3	62419694	62419695	62419695	A	G	missense_variant
17-36030685-36030686-G-A		1		H2-T23	P298S	1	SSTVSNMVI	1	1	663.88	6872.11	2.801	16	36.919	0	0	0	0.049	NoExpr	Pending	17	36030685	36030686	36030686	G	A	missense_variant
10-115385762-115385763-G-C		1		Zfc3h1	G189A	1	SGFASSQSW	4	1	663.88	1291.99	2.801	3.6	7.019	0	0	0	0.079	NoExpr	Pending	10	115385762	115385763	115385763	G	C	missense_variant
11-59328678-59328679-A-C		1		Wnt9a	K177T	1	KYSSTFVKEFL	5	1	664.95	1672.99	0.71	0.79	0.338	0	0	0	0.334	NoExpr	Pending	11	59328678	59328679	59328679	A	C	missense_variant
4-129083798-129083799-G-A		2		Rnf19b	S602N	1	HYQLVSGNS	8	2	665.41	353.256	1.6	0.78	22.297	0	0	0	0.499	NoExpr	Pending	4	129083798	129083799	129083799	G	A	missense_variant
9-109274569-109274570-G-A		1		Fbxw14	H348Y	1	DYFGVSDKDV	2	1	665.98	8950.22	0.9	16	0.02	0	0	0	0.069	NoExpr	Pending	9	109274569	109274570	109274570	G	A	missense_variant
7-5480927-5480928-C-A		1		Vmn2r28	A758S	1	GYLACLSL	7	1	665.98	1048.24	0.44	0.68	0	0	0	0	0.027	NoExpr	Pending	7	5480927	5480928	5480928	C	A	missense_variant
7-10158938-10158939-C-A		1		Vmn2r52	A758S	1	GYLACLSL	7	1	665.98	1048.24	0.44	0.68	0	0	0	0	0.032	NoExpr	Pending	7	10158938	10158939	10158939	C	A	missense_variant
3-62488922-62488923-C-T		1		Dhx36	A438T	1	IYKERWPTYI	8	1	666.15	1723.69	0.86	2.6	23.512	0	0	0	0.331	NoExpr	Pending	3	62488922	62488923	62488923	C	T	missense_variant
10-26990809-26990810-C-T		1	1	Lama2	G2896D	1	NYTTRRIDPV	8	2	666.93	854.358	0.98	1.1	0.191	0	0	0	0.52	NoExpr	Pending	10	26990809	26990810	26990810	C	T	missense_variant
6-132980313-132980314-T-G		1		Tas2r109	K218Q	1	LHQRMQQHV	3	1	666.95	2225.89	1.4	5.1	0	0	0	0	0.042	NoExpr	Pending	6	132980313	132980314	132980314	T	G	missense_variant
8-71472239-71472240-G-A		1		Dda1	E44K	1	REYPSKQII	6	1	668.48	822.413	2.1	2.5	37.56	0	0	0	0.232	NoExpr	Pending	8	71472239	71472240	71472240	G	A	missense_variant
11-58552173-58552174-G-A		1	1	Olfr328	H22Y	1	FSQSKYPAL	6	2	669.89	841.395	2.5	3	0	0	0	0	0.058	NoExpr	Pending	11	58552173	58552174	58552174	G	A	missense_variant
10-105413515-105413516-C-G		1	1	Tmtc2	A119P	1	YWTFMPGLM	6	2	670.29	503.605	1.8	1.6	0.102	0	0	0	0.136	NoExpr	Pending	10	105413515	105413516	105413516	C	G	missense_variant
19-5892550-5892551-C-T		1		Tigd3	A184T	1	LYRTVPGRV	4	1	673.38	487.63	1.3	1.3	0.613	0	0	0	0.25	NoExpr	Pending	19	5892550	5892551	5892551	C	T	missense_variant
10-76420486-76420487-A-G		1		Pcnt	F640L	1	KFAKEQDAL	9	1	674.19	5239.14	2.1	12	3.574	0	0	0	0.551	NoExpr	Pending	10	76420486	76420487	76420487	A	G	missense_variant
7-26611631-26611632-A-T		1	3	Vmn1r185	F149L	1	LIIFVYPFI	1	4	674.19	575.44	3.199	2.3	0	0	0	0	0.074	NoExpr	Pending	7	26611631	26611632	26611632	A	T	missense_variant
5-137380871-137380872-C-G		1	1	Zan	G5322A	1	EEQAATFIC	4	2	674.19	767.658	3.199	3.5	0.001	0	0	0	0.206	NoExpr	Pending	5	137380871	137380872	137380872	C	G	missense_variant
12-40073324-40073325-C-T		1		Scin	A520T	1	RNLTSITRI	4	1	674.67	430.616	0.8	0.89	0.429	0	0	0	0.423	NoExpr	Pending	12	40073324	40073325	40073325	C	T	missense_variant
2-181794824-181794825-G-A		1		Myt1	R91Q	1	KYSRHQSLQ	6	1	674.93	464.793	2.199	1.7	0	0	0	0	0.05	NoExpr	Pending	2	181794824	181794825	181794825	G	A	missense_variant
7-101836738-101836739-C-T		1		Inppl1	C5Y	1	VYGTPSPGGAL	2	1	677.19	10414	1	18	11.938	0	0	0	0.192	NoExpr	Pending	7	101836738	101836739	101836739	C	T	missense_variant
11-59099940-59099941-T-C		2		Obscn	E1602G	1	KGQQAHSEV	2	2	677.78	885.299	1.9	2.3	0.384	0	0	0	0.023	NoExpr	Pending	11	59099940	59099941	59099941	T	C	missense_variant
4-40190786-40190787-C-T		2		Aco1	A717V	1	SYGSRRGNDV	10	2	678.73	385.19	0.88	0.39	16.186	0	0	0	0.055	NoExpr	Pending	4	40190786	40190787	40190787	C	T	missense_variant
9-70125150-70125151-G-C		2		Fam81a	P20A	1	AYSSVSLVE	1	2	679.34	3972.74	2.199	7.9	0.008	0	0	0	0.234	NoExpr	Pending	9	70125150	70125151	70125151	G	C	missense_variant
1-85028735-85028736-T-C		2		AC167036.2	I37V	1	SLTVVIIAI	4	2	681.52	948.615	3.301	4.3	0	0	0	0	0.036	NoExpr	Pending	1	85028735	85028736	85028736	T	C	missense_variant
2-41112689-41112690-G-T		1		Lrp1b	S2099Y	1	VYDRAHANGSY	2	1	681.52	19893.4	1.2	22	0.022	0	0	0	0.03	NoExpr	Pending	2	41112689	41112690	41112690	G	T	missense_variant
4-156338745-156338746-G-A		1	1	Vmn2r-ps159	V747I	1	SVIAFHIVL	7	2	682.07	3264.6	2.6	8	0	0	0	0	0.02	NoExpr	Pending	4	156338745	156338746	156338746	G	A	missense_variant
9-73075511-73075512-A-C		1		Rab27a	K33T	1	LYQYTDGTF	8	1	682.48	2239.19	1.3	3.7	1.046	0	0	0	0.191	NoExpr	Pending	9	73075511	73075512	73075512	A	C	missense_variant
7-7291660-7291661-G-C		1		Clcn4	T367S	1	IAVTAVSAI	7	1	682.75	1064.78	2.1	2.7	10.637	0	0	0	0.166	NoExpr	Pending	7	7291660	7291661	7291661	G	C	missense_variant
2-98662367-98662368-T-G		1	1	Gm10801	F44L	1	IFHVLHWTL	9	1	685.22	482.87	1.9	1.6	0.12	0	0	0	0.022	NoExpr	Pending	2	98662367	98662368	98662368	T	G	missense_variant
9-20588518-20588519-C-T		2		Zfp846	T15I	1	CYQVSVIFDDV	7	2	685.3	344.913	1.7	0.54	3.269	0	0	0	0.289	NoExpr	Pending	9	20588518	20588519	20588519	C	T	missense_variant
17-23640153-23640154-C-T		1		Mmp25	R173H	1	KKHTLTWSI	3	1	688.93	1376.93	1.4	3.2	0.045	0	0	0	0.094	NoExpr	Pending	17	23640153	23640154	23640154	C	T	missense_variant
7-35547979-35547980-G-A		1	1	Nudt19	L335F	1	SPYVYEIYMTF	11	2	688.93	819.146	0.75	0.78	33.06	0	0	0	0.61	NoExpr	Pending	7	35547979	35547980	35547980	G	A	missense_variant
7-3717411-3717412-A-T		1		Pirb	Y321N	1	EYNEPRLSVL	3	1	690.97	548.858	0.6	0.38	5.132	0	0	0	0.029	NoExpr	Pending	7	3717411	3717412	3717412	A	T	missense_variant
4-118869385-118869386-G-A		1		Olfr1331	V202I	1	HIIEMVDLI	9	1	691.97	4637.25	2	9.8	0	0	0	0	0.524	NoExpr	Pending	4	118869385	118869386	118869386	G	A	missense_variant
19-20640086-20640087-C-T		1		Aldh1a1	S461L	1	CYMMLLAQC	6	1	693.57	632.543	1.7	1.2	0.098	0	0	0	0.359	NoExpr	Pending	19	20640086	20640087	20640087	C	T	missense_variant
4-56946850-56946851-G-C		1		Tmem245	S187R	1	DYFSRLWIWTL	5	1	694.56	449.656	1.5	0.92	3.541	0	0	0	0.19	NoExpr	Pending	4	56946850	56946851	56946851	G	C	missense_variant
6-42912284-42912285-C-T		1		Olfr447	A254V	1	YGTVIVMYI	4	1	695.44	392.88	2.199	1.5	0	0	0	0	0.843	NoExpr	Pending	6	42912284	42912285	42912285	C	T	missense_variant
18-88869528-88869529-G-A		1		Socs6	R421C	1	RYLLSLSFC	9	1	695.44	1045.59	0.96	2.9	8.376	0	0	0	0.027	NoExpr	Pending	18	88869528	88869529	88869529	G	A	missense_variant
2-130248713-130248714-G-A		1		Tmc2	S660N	1	CWAVMSNNV	7	1	698.65	265.62	1.3	0.6	0	0	0	0	0.43	NoExpr	Pending	2	130248713	130248714	130248714	G	A	missense_variant
5-111225997-111225998-C-G		1		Ttc28	A1069G	1	GKTVSYSSL	1	1	698.65	369.905	2.199	1.7	0.995	0	0	0	0.212	NoExpr	Pending	5	111225997	111225998	111225998	C	G	missense_variant
3-83033096-83033097-G-A		1		Fga	G686E	1	SYRGTAEDAL	7	1	701.66	389.85	0.91	0.56	0	0	0	0	0.163	NoExpr	Pending	3	83033096	83033097	83033097	G	A	missense_variant
17-18597855-18597856-G-A		1		Vmn2r96	G565E	1	CFMALESYA	6	1	701.88	575.559	1.6	1.5	0	0	0	0	0.04	NoExpr	Pending	17	18597855	18597856	18597856	G	A	missense_variant
4-147178798-147178799-A-G		1		Zfp991	H211R	1	RYRSSKSDK	1	1	703.22	496.695	1.8	1.2	5.091	0	0	0	0.667	NoExpr	Pending	4	147178798	147178799	147178799	A	G	missense_variant
5-15030266-15030267-G-C		1		Gm17019	A177G	1	KEVQGDWAI	5	1	704.01	497.976	3	2.5	0.103	0	0	0	0.056	NoExpr	Pending	5	15030266	15030267	15030267	G	C	missense_variant
12-113685590-113685591-C-G		2		Ighv2-5	A81P	1	APFMSRLSI	2	2	708.09	933.448	2.199	2.7	0	0	0	0	0.03	NoExpr	Pending	12	113685590	113685591	113685591	C	G	missense_variant
10-79479207-79479208-A-C		1	1	Vmn2r83	D430A	1	AYKARTML	4	2	710.56	806.492	0.42	0.91	0	0	0	0	0.048	NoExpr	Pending	10	79479207	79479208	79479208	A	C	missense_variant
2-71118177-71118178-G-C		1	1	Cybrd1	G20A	1	AFLSVIFVL	1	1	710.94	504.464	2.699	2	0.122	0	0	0	0.06	NoExpr	Pending	2	71118177	71118178	71118178	G	C	missense_variant
1-186632484-186632485-T-C		1		Tgfb2	S271G	1	NYIIPNKGEEL	8	1	711.02	711.017	0.7	0.7	0.162	0	0	0	0.026	NoExpr	Pending	1	186632484	186632485	186632485	T	C	missense_variant
1-132046445-132046446-A-G		1		Mfsd4a	V382A	1	VFINVVGAV	8	1	711.64	1303.95	2.1	3.1	2.612	0	0	0	0.224	NoExpr	Pending	1	132046445	132046446	132046446	A	G	missense_variant
X-127063259-127063260-G-A		2		Gm382	V1023I	1	KRIKSLQME	3	2	711.66	2781.92	3.5	13	0	0	0	0	0.75	NoExpr	Pending	X	127063259	127063260	127063260	G	A	missense_variant
18-35213088-35213089-A-G		1		Lrrtm2	S387P	1	EYTKISSPS	8	1	711.85	605.466	2.199	1.4	0.726	0	0	0	0.203	NoExpr	Pending	18	35213088	35213089	35213089	A	G	missense_variant
17-46134990-46134991-C-T		1		Rsph9	E218K	1	KYDIPRGSWSI	1	1	711.98	1737.86	0.48	2	0.087	0	0	0	0.395	NoExpr	Pending	17	46134990	46134991	46134991	C	T	missense_variant
4-9537694-9537695-C-T		1		Asph	R361K	1	KYPQSPRAK	9	1	713	1312.47	3.4	5	28.8	0	0	0	0.182	NoExpr	Pending	4	9537694	9537695	9537695	C	T	missense_variant
12-113769970-113769971-T-C		1		Ighv2-9-1	N77D	1	TDYNSALKSRL	2	1	714.3	1351.7	3	3.1	0	0	0	0	0.033	NoExpr	Pending	12	113769970	113769971	113769971	T	C	missense_variant
4-111980312-111980313-T-A		1		Skint7	F96Y	1	EYANRTEYV	8	1	717.11	1697.62	1.7	2.6	0	0	0	0	0.02	NoExpr	Pending	4	111980312	111980313	111980313	T	A	missense_variant
6-97296288-97296289-G-A		1		Frmd4b	T714I	1	FFILSKSQR	3	1	717.94	1866.77	2.199	5.1	2.64	0	0	0	0.062	NoExpr	Pending	6	97296288	97296289	97296289	G	A	missense_variant
17-45568408-45568409-T-A		2		Hsp90ab1	N629Y	1	KHLEIYPDHPI	6	2	719.41	1003.51	1.3	1.3	1571.3	0	0	0	0.04	NoExpr	Pending	17	45568408	45568409	45568409	T	A	missense_variant
10-107494294-107494295-G-C		1		Myf6	A137G	1	KVEILRSGI	8	1	719.41	263.081	3.5	1.3	0.236	0	0	0	0.226	NoExpr	Pending	10	107494294	107494295	107494295	G	C	missense_variant
7-29103579-29103580-A-G		1		Ryr1	L676P	1	FPTAQATHL	2	1	719.41	611.069	2.199	1.8	0.153	0	0	0	0.262	NoExpr	Pending	7	29103579	29103580	29103580	A	G	missense_variant
2-86122769-86122770-C-T		1	1	Olfr1038-ps	P282L	1	SVFYTVILM	8	2	719.45	1629.3	2.6	3.2	0	0	0	0	0.376	NoExpr	Pending	2	86122769	86122770	86122770	C	T	missense_variant
10-102544813-102544814-C-T		1		Rassf9	T19I	1	TRHKNRSPI	9	1	719.6	11805.7	1.2	19	0.056	0	0	0	0.664	NoExpr	Pending	10	102544813	102544814	102544814	C	T	missense_variant
2-164289936-164289937-G-A		1		Svs3a	V143M	1	GQMKSQTML	3	1	721.26	2361.89	2.199	6.2	0	0	0	0	0.067	NoExpr	Pending	2	164289936	164289937	164289937	G	A	missense_variant
5-36065346-36065347-A-C		1	1	Sorcs2	F355V	1	TVQDEYIVL	8	2	722.49	337.323	3.1	1.8	1.236	0	0	0	0.105	NoExpr	Pending	5	36065346	36065347	36065347	A	C	missense_variant

2-88446227-88446228-T-C		1		Olfr1182	T237A	1	ALSACGSHI	4	1	724.59	1135.25	2.199	3.2	0	0	0	0	0.008	NoExpr	Pending	2	88446227	88446228	88446228	T	C	missense_variant
7-3716727-3716728-G-A		1		Pirb	T473I	1	IMQHSSQQTDI	11	1	725.91	6978.43	3	13	5.132	0	0	0	0.023	NoExpr	Pending	7	3716727	3716728	3716728	G	A	missense_variant
6-43116448-43116449-G-A		1		Olfr441	A236T	1	TFSTCSSHL	1	1	727.23	653.522	3.5	2.1	0	0	0	0	0.838	NoExpr	Pending	6	43116448	43116449	43116449	G	A	missense_variant
7-8471616-8471617-C-G		1		Vmn2r45	G804A	1	VYHSTKAKHMV	7	1	727.58	419.643	0.7	0.6	0	0	0	0	0.089	NoExpr	Pending	7	8471616	8471617	8471617	C	G	missense_variant
17-20042712-20042713-T-G		1	1	Vmn2r104	Q162P	1	APIGTLLQL	2	2	727.78	18551.1	2.699	17	0	0	0	0	0.225	NoExpr	Pending	17	20042712	20042713	20042713	T	G	missense_variant
11-104289964-104289965-C-T		1		Mapt	P82L	1	AAAQLHTEI	5	1	727.93	1686.9	2	3.4	0.122	0	0	0	0.549	NoExpr	Pending	11	104289964	104289965	104289965	C	T	missense_variant
12-72157485-72157486-T-G		1		Ccdc175	K260N	1	TYYKKNELTRL	6	1	728	611.631	1.2	0.81	0	0	0	0	0.24	NoExpr	Pending	12	72157485	72157486	72157486	T	G	missense_variant
2-86217361-86217362-A-C		1		Olfr1046	F116C	1	IFIITELCI	8	1	728.22	656.281	2.1	1.6	0	0	0	0	0.046	NoExpr	Pending	2	86217361	86217362	86217362	A	C	missense_variant
16-56260477-56260478-C-G		2		Impg2	P882A	1	SVHYTEMAI	8	2	729.61	1452.41	3.1	3.3	0.025	0	0	0	0.129	NoExpr	Pending	16	56260477	56260478	56260478	C	G	missense_variant
15-50660861-50660862-C-G		1	1	Trps1	C1224S	1	CVHSGIVFL	4	2	730.85	732.825	2.9	2.7	5.236	0	0	0	0.048	NoExpr	Pending	15	50660861	50660862	50660862	C	G	missense_variant
9-21230766-21230767-C-T		1		Keap1	V604M	1	SGMGVAVTM	3	1	731.58	2276.46	2.199	6.1	17.732	0	0	0	0.239	NoExpr	Pending	9	21230766	21230767	21230767	C	T	missense_variant
16-32751954-32751956-AC-GA		1		Muc4	N611R	1	TETSSQRTI	7	1	732.98	467.832	2.199	1.4	0.018	0	0	0	0.015	NoExpr	Pending	16	32751954	32751956	32751955	AC	GA	missense_variant
11-60779684-60779685-T-G		2		Smcr8	I553S	1	AYADNEGAS	9	2	732.98	44.884	2.199	0.2	4.952	0	0	0	0.175	NoExpr	Pending	11	60779684	60779685	60779685	T	G	missense_variant
15-60919764-60919765-G-A		1		A1bg	A274V	1	GFSPTRDVI	8	1	733.42	490.072	1.5	0.8	0	0	0	0	0.882	NoExpr	Pending	15	60919764	60919765	60919765	G	A	missense_variant
11-45983392-45983393-G-A		1		Sox30	A377T	1	NAFMVWTRI	7	1	734.67	2938.26	2.301	7.5	0	0	0	0	0.162	NoExpr	Pending	11	45983392	45983393	45983393	G	A	missense_variant
4-139779672-139779673-C-T		1		Pax7	G351E	1	AYEARHSFS	3	1	736.36	853.277	2.199	2.5	0	0	0	0	0.286	NoExpr	Pending	4	139779672	139779673	139779673	C	T	missense_variant
11-85838473-85838474-C-G		1		Tbx2	A561G	1	QHMLGSQGI	5	1	736.65	638.396	1.7	1.3	0.247	0	0	0	0.241	NoExpr	Pending	11	85838473	85838474	85838474	C	G	missense_variant
5-94383664-94383665-T-G		1		AA792892	L136W	1	LYAWDLMPL	4	1	740.05	1143.56	1.7	1.5	0	0	0	0	0.041	NoExpr	Pending	5	94383664	94383665	94383665	T	G	missense_variant
8-14942625-14942626-C-T		1		Arhgef10	T288I	1	KKQLSHDLI	9	1	741.46	12366.9	2.199	24	5.423	0	0	0	0.586	NoExpr	Pending	8	14942625	14942626	14942626	C	T	missense_variant
6-86732975-86732976-G-C		2		Gmcl1	D63E	1	CYCHPESETDD	6	2	743.14	735.143	1.9	1.3	15.174	0	0	0	0.218	NoExpr	Pending	6	86732975	86732976	86732976	G	C	missense_variant
17-34715849-34715850-G-C		2		Tnxb	E2478Q	1	QYQDTENQTQA	8	2	743.14	735.143	1.3	1.3	1.654	0	0	0	0.393	NoExpr	Pending	17	34715849	34715850	34715850	G	C	missense_variant
18-45685368-45685369-C-T		2		Kcnn2	T511I	1	ILIGSIHAL	1	2	743.46	957.767	1.5	2.2	0	0	0	0	0.387	NoExpr	Pending	18	45685368	45685369	45685369	C	T	missense_variant
5-109047050-109047051-G-C		1		Vmn2r11	T803S	1	SFLPVYHST	1	1	743.46	2787.79	2.301	4.8	0	0	0	0	0.042	NoExpr	Pending	5	109047050	109047051	109047051	G	C	missense_variant
X-165252383-165252384-A-T		3	1	Glra2	Y256N	1	NYLIQMYI	1	4	743.53	323.11	0.96	0.46	0	0	0	0	0.456	NoExpr	Pending	X	165252383	165252384	165252384	A	T	missense_variant
13-89705412-89705413-C-G		1		Vcan	G476A	1	ISQHATASQAV	7	1	743.75	1174.84	2.5	1.7	1.004	0	0	0	0.186	NoExpr	Pending	13	89705412	89705413	89705413	C	G	missense_variant
2-98662299-98662300-A-G		1		Gm10801	S22G	1	GRCFSFSMI	1	1	750.05	239.933	2.301	0.8	0.12	0	0	0	0.024	NoExpr	Pending	2	98662299	98662300	98662300	A	G	missense_variant
16-32751941-32751942-A-T		1		Muc4	T607S	1	TESSSQNTI	3	1	750.34	467.832	2.301	1.4	0.018	0	0	0	0.015	NoExpr	Pending	16	32751941	32751942	32751942	A	T	missense_variant
1-33777641-33777642-G-A		1		Zfp451	P409L	1	CHISEGSRL	9	1	750.34	5309.95	1.9	13	11.241	0	0	0	0.691	NoExpr	Pending	1	33777641	33777642	33777642	G	A	missense_variant
12-115847942-115847943-T-A		1		Ighv1-76	T97S	1	SAYMQLSSL	1	1	751.22	2296.63	2.199	6	0	0	0	0	0.019	NoExpr	Pending	12	115847942	115847943	115847943	T	A	missense_variant
7-141639591-141639592-C-T		1		Muc6	E1723K	1	SVTPTSKVI	7	1	751.22	540.866	3.6	2.3	0.024	0	0	0	0.027	NoExpr	Pending	7	141639591	141639592	141639592	C	T	missense_variant
6-123315808-123315809-T-C		1		Vmn2r19	L270S	1	RFMERFSSTRV	8	1	751.22	828.057	1.3	1.5	0	0	0	0	0.488	NoExpr	Pending	6	123315808	123315809	123315809	T	C	missense_variant
11-116677607-116677608-G-A		1		St6galnac2	P343L	1	HYFEREKKLLI	9	1	753.15	633.695	0.5	0.6	0.127	0	0	0	0.284	NoExpr	Pending	11	116677607	116677608	116677608	G	A	missense_variant
7-23984778-23984779-T-C		1		Vmn1r181	L223P	1	QYIFTPNQD	6	1	753.51	771.063	2.301	2.3	0	0	0	0	0.029	NoExpr	Pending	7	23984778	23984779	23984779	T	C	missense_variant
X-164533533-164533534-C-A		3		Asb9	L167M	1	DYNISHLGTPM	11	3	754.59	370.52	0.98	0.55	0	0	0	0	0.114	NoExpr	Pending	X	164533533	164533534	164533534	C	A	missense_variant
7-85410224-85410225-G-A		1		Vmn2r69	S492F	1	LYMFKEMI	4	1	754.9	314.72	0.97	0.2	0	0	0	0	0.046	NoExpr	Pending	7	85410224	85410225	85410225	G	A	missense_variant
2-122290606-122290607-G-A		1	1	Duox2	L775F	1	QFFAQVLDI	2	2	755.25	2642.96	2.301	6.9	0	0	0	0	0.345	NoExpr	Pending	2	122290606	122290607	122290607	G	A	missense_variant
19-11472453-11472454-C-T		1	1	Ms4a6c	P70L	1	LGIILASVL	9	2	756.99	6840.53	2.301	16	26.147	0	0	0	0.04	NoExpr	Pending	19	11472453	11472454	11472454	C	T	missense_variant
4-73403464-73403465-A-C		2		Gm11487	L111W	1	AYRWYSTYDW	4	2	762.11	808.81	0.98	1.2	0	0	0	0	0.025	NoExpr	Pending	4	73403464	73403465	73403465	A	C	missense_variant
10-129625329-129625330-T-C		2		Olfr798	I244V	1	SHMVVVSIS	4	2	762.24	1219.24	2.6	3.4	0	0	0	0	0.129	NoExpr	Pending	10	129625329	129625330	129625330	T	C	missense_variant
7-23835106-23835107-A-T		1		Vmn1r176	V207E	1	IWSSVSMEI	8	1	762.43	839.963	1.4	1	0	0	0	0	0.142	NoExpr	Pending	7	23835106	23835107	23835107	A	T	missense_variant
12-114746390-114746392-CT-TC		1		Ighv1-22	S78D	1	GYINPNNGGTD	11	1	763.63	562.186	3.4	2.2	0	0	0	0	0.04	NoExpr	Pending	12	114746390	114746392	114746391	CT	TC	missense_variant
1-85485033-85485034-T-A		3		AC147806.1	I37F	1	SITTSLTFV	8	3	764.29	1229.28	2.301	3.8	0	0	0	0	0.062	NoExpr	Pending	1	85485033	85485034	85485034	T	A,C	missense_variant
15-35709611-35709612-T-G		1		Vps13b	S1902A	1	TRSAARQAL	4	1	764.29	1143.56	2.301	3.2	4.081	0	0	0	0.137	NoExpr	Pending	15	35709611	35709612	35709612	T	G	missense_variant
15-77389712-77389713-T-A		1	1	Apol7a	K183M	1	EIIRMLHAL	5	2	765.76	12850.9	2.301	18	13.286	0	0	0	0.029	NoExpr	Pending	15	77389712	77389713	77389713	T	A	missense_variant
12-101509249-101509250-A-G		1		Catsperb	E351G	1	STIKNGKSI	6	1	767.66	792.984	1.5	1.5	0	0	0	0	0.075	NoExpr	Pending	12	101509249	101509250	101509250	A	G	missense_variant
14-101913324-101913325-C-A		1		Lmo7	F1150L	1	SSLSVTTDL	9	1	767.66	6267.44	1.8	9.9	31.723	0	0	0	0.146	NoExpr	Pending	14	101913324	101913325	101913325	C	A	missense_variant
16-32752014-32752015-C-A		1		Muc4	P631Q	1	SSAQSTTHM	4	1	767.66	585.724	1.7	1.7	0.018	0	0	0	0.022	NoExpr	Pending	16	32752014	32752015	32752015	C	A	missense_variant
17-35321065-35321066-A-C		1		H2-Q1	S104R	1	RYYNQSKGGI	1	1	767.82	293.74	0.9	0.45	6.324	0	0	0	0.023	NoExpr	Pending	17	35321065	35321066	35321066	A	C	missense_variant
18-37005864-37005865-A-C		1		Pcdha11	E182D	1	EYFSLDVPTTD	6	1	770.69	1354.82	2.801	2.9	0	0	0	0	0.157	NoExpr	Pending	18	37005864	37005865	37005865	A	C	missense_variant
17-25746951-25746952-C-G		1		Mslnl	T600S	1	SAYPPSSLI	1	1	771.06	2506.63	2.301	4.3	0.048	0	0	0	0.051	NoExpr	Pending	17	25746951	25746952	25746952	C	G	missense_variant
17-12719893-12719894-A-C		1		Igf2r	F508V	1	VFINVCHRV	1	1	773.8	390.176	1.2	0.8	4.042	0	0	0	0.224	NoExpr	Pending	17	12719893	12719894	12719894	A	C	missense_variant
6-122713538-122713539-C-T		1		Nanog	P301L	1	NYSVTLPGEI	6	1	775.3	1682.67	1	2	0	0	0	0	0.04	NoExpr	Pending	6	122713538	122713539	122713539	C	T	missense_variant
3-96263012-96263013-G-C		1	1	H4c14	L98V	1	RQGRTVYGF	6	2	775.94	992.727	2.199	2.7	0.078	0	0	0	0.053	NoExpr	Pending	3	96263012	96263013	96263013	G	C	missense_variant
17-34958106-34958107-G-A		2		Hspa1b	R301W	1	FYTSITRAW	9	2	776.48	1325.14	1	2.3	4.701	0	0	0	0.091	NoExpr	Pending	17	34958106	34958107	34958107	G	A	missense_variant
3-28807281-28807282-G-A		2		Rpl22l1	R107H	1	HYFQISQDE	1	2	778.5	1057.45	2.4	2.9	1156.4	0	0	0	0.063	NoExpr	Pending	3	28807281	28807282	28807282	G	A	missense_variant
X-94638018-94638019-C-G		1		Gspt2	A588G	1	GRLRTAGTI	1	1	782.1	361.626	1.3	0.95	6.342	0	0	0	0.344	NoExpr	Pending	X	94638018	94638019	94638019	C	G	missense_variant
3-151517481-151517482-A-G		2		Adgrl4	Y565C	1	IHLCLIVVGVI	4	2	782.91	1046.43	2.9	2.2	3.979	0	0	0	0.381	NoExpr	Pending	3	151517481	151517482	151517482	A	G	missense_variant
15-82391001-82391002-T-G		1		Cyp2d11	K248T	1	QGQTSFMAI	4	1	783.59	543.363	2	1.8	0	0	0	0	0.143	NoExpr	Pending	15	82391001	82391002	82391002	T	G	missense_variant
12-114708823-114708824-T-A		1		Ighv1-19	S59C	1	CHGKSLEWI	1	1	783.9	534.884	2.199	1.4	0	0	0	0	0.062	NoExpr	Pending	12	114708823	114708824	114708824	T	A	missense_variant
12-114746448-114746449-T-A		1		Ighv1-22	S59C	1	CHGKSLEWI	1	1	783.9	534.884	2.199	1.4	0	0	0	0	0.042	NoExpr	Pending	12	114746448	114746449	114746449	T	A	missense_variant
17-7804856-7804857-C-T		1	1	Fndc1	E40K	1	KVNAKTHSF	5	2	784.45	759.397	2.1	2.2	0.16	0	0	0	0.424	NoExpr	Pending	17	7804856	7804857	7804857	C	T	missense_variant
18-20575041-20575042-G-A		1		Dsg2	E67K	1	KDLSRKNPI	1	1	787.86	8265.33	2.6	18	0.048	0	0	0	0.051	NoExpr	Pending	18	20575041	20575042	20575042	G	A	missense_variant
13-12294368-12294369-G-T		1		Actn2	L273I	1	KVIAVNQEN	3	1	789.02	2084.92	3.6	9.1	0.322	0	0	0	0.184	NoExpr	Pending	13	12294368	12294369	12294369	G	T	missense_variant
4-88683345-88683346-T-A		2		Ifna2	K145I	1	IYFHRITVYL	1	2	790.47	266.59	1.1	0.41	0	0	0	0	0.054	NoExpr	Pending	4	88683345	88683346	88683346	T	A	missense_variant
2-58137903-58137904-C-T		1		Cytip	V190M	1	KWMELRSLH	3	1	790.84	2673.56	2.4	6.9	3.51	0	0	0	0.468	NoExpr	Pending	2	58137903	58137904	58137904	C	T	missense_variant

14-53635152-53635153-G-T		1		Trav13-2	A29S	1	QVQQSPSSL	7	1	790.84	4614.13	2.801	12	0	0	0	0	0.019	NoExpr	Pending	14	53635152	53635153	53635153	G	T	missense_variant
17-20113527-20113528-C-A		1		Fpr-rs7	L233F	1	GFVNSSRPL	2	1	791.35	6564.03	2.301	7.8	0	0	0	0	0.026	NoExpr	Pending	17	20113527	20113528	20113528	C	A	missense_variant
11-78751079-78751080-C-G		1		Ccnq	V195L	1	RFQAQHLAL	9	1	791.86	1467.74	1.7	3.1	18.278	0	0	0	0.045	NoExpr	Pending	11	78751079	78751080	78751080	C	G	missense_variant
17-33997115-33997116-C-A		1		H2-K1	V310F	1	SNMATVAFL	8	1	792.67	792.984	2.4	1.9	663.43	0	0	0	0.021	NoExpr	Pending	17	33997115	33997116	33997116	C	A	missense_variant
4-88571581-88571582-T-C		1		Ifna14	K73E	1	QQIKEAQAI	5	1	792.67	1321.57	2.4	3.5	0	0	0	0	0.035	NoExpr	Pending	4	88571581	88571582	88571582	T	C	missense_variant
11-58529814-58529815-G-A		1		Olfr330	T57I	1	LILLVLSDI	9	1	792.67	13004.4	2.4	24	0	0	0	0	0.015	NoExpr	Pending	11	58529814	58529815	58529815	G	A	missense_variant
7-40993293-40993294-A-C		1		4930433I11Rik	D129A	1	SDNASLGGI	4	1	792.98	1145.75	3.9	4.9	0	0	0	0	0.085	NoExpr	Pending	7	40993293	40993294	40993294	A	C	missense_variant
11-109517419-109517420-G-A		1		Arsg	A133T	1	LRQEGYVTTM	9	1	792.98	2192.64	2.801	4.3	0.639	0	0	0	0.268	NoExpr	Pending	11	109517419	109517420	109517420	G	A	missense_variant
3-53516991-53516992-G-A		1		Frem2	S3008F	1	WYIHTIYTVKF	11	1	792.98	1607.56	1.4	1.9	0	0	0	0	0.273	NoExpr	Pending	3	53516991	53516992	53516992	G	A	missense_variant
2-98667255-98667256-G-T		1		Gm10800	R16S	1	CHISSPTVDI	5	1	793.07	2198.63	0.6	2.4	0.566	0	0	0	0.018	NoExpr	Pending	2	98667255	98667256	98667256	G	T	missense_variant
7-49464780-49464781-T-A		1		Nav2	S838T	1	RQLATRGSSI	5	1	793.34	777.07	0.6	0.6	4.528	0	0	0	0.656	NoExpr	Pending	7	49464780	49464781	49464781	T	A	missense_variant
6-67896529-67896530-C-A		1		Igkv14-126	P81T	1	YYATSLADGVT	11	1	794.29	874.742	1.6	1.9	0	0	0	0	0.065	NoExpr	Pending	6	67896529	67896530	67896530	C	A	missense_variant
17-45568404-45568405-G-A		2		Hsp90ab1	P630L	1	LEINLDHPI	5	2	796.32	1845.4	1.9	5	1571.3	0	0	0	0.04	NoExpr	Pending	17	45568404	45568405	45568405	G	A	missense_variant
17-34958181-34958182-A-C		1		Hspa1b	S276A	1	KRTLSASTQ	6	1	800	822.413	3.301	3.3	4.701	0	0	0	0.188	NoExpr	Pending	17	34958181	34958182	34958182	A	C	missense_variant
6-57524931-57524932-T-G		1		Ppm1k	K82T	1	TYGKPIPKI	1	1	800	253.081	2.4	0.7	1.524	0	0	0	0.203	NoExpr	Pending	6	57524931	57524932	57524932	T	G	missense_variant
12-103587518-103587519-T-C		1		Ppp4r4	V412A	1	PEAPVRHTI	3	1	800	1585.22	1.6	3.6	0	0	0	0	0.231	NoExpr	Pending	12	103587518	103587519	103587519	T	C	missense_variant
14-53538269-53538270-G-A		1		Trav12-1	R4H	1	MNMHPVTSSV	4	1	802.53	727.232	1.3	1.2	0	0	0	0	0.029	NoExpr	Pending	14	53538269	53538270	53538270	G	A	missense_variant
6-41188956-41188957-A-T		1		Trbv20	L105F	1	AYLEDRGFYL	8	1	802.86	999.171	0.8	1	0	0	0	0	0.853	NoExpr	Pending	6	41188956	41188957	41188957	A	T	missense_variant
12-114222989-114222990-C-T		1		Ighv7-4	S54N	1	YYMNWVRQP	4	1	803.69	696.429	2.5	1.8	0	0	0	0	0.033	NoExpr	Pending	12	114222989	114222990	114222990	C	T	missense_variant
5-124779161-124779162-G-C		1		Dnah10	E2063Q	1	GYAGRTQL	7	1	806.31	695.51	0.3	0.3	0.004	0	0	0	0.076	NoExpr	Pending	5	124779161	124779162	124779162	G	C	missense_variant
2-128676211-128676212-C-T		1	1	Anapc1	D241N	1	NPAVKIVFL	1	2	806.85	2054.82	2.4	4.6	19.667	0	0	0	0.442	NoExpr	Pending	2	128676211	128676212	128676212	C	T	missense_variant
5-3344457-3344458-G-C		1		Cdk6	R31P	1	AYGKVFKAP	9	1	807.72	908.364	3.301	3.6	17.121	0	0	0	0.064	NoExpr	Pending	5	3344457	3344458	3344458	G	C	missense_variant
3-39009783-39009784-G-A		1		Fat4	A4630T	1	SITPSDADI	3	1	810.33	598.537	4	3	0.049	0	0	0	0.177	NoExpr	Pending	3	39009783	39009784	39009784	G	A	missense_variant
2-109894548-109894549-T-C		1		Lin7c	V41A	1	KLQALQRAL	8	1	810.33	1039.3	1.6	2.2	45.493	0	0	0	0.189	NoExpr	Pending	2	109894548	109894549	109894549	T	C	missense_variant
11-71123708-71123710-GG-CA		1		Nlrp1a	T238M	1	SQMQPVHTL	3	1	810.33	1858.19	2.1	4.7	0	0	0	0	0.089	NoExpr	Pending	11	71123708	71123710	71123709	GG	CA	missense_variant
11-3221174-3221175-C-T		1		Eif4enif1	S222L	1	SYTEEEPEWFL	11	1	812.29	2416.9	3.301	8.5	18.018	0	0	0	0.353	NoExpr	Pending	11	3221174	3221175	3221175	C	T	missense_variant
6-84575234-84575235-G-A		1		Cyp26b1	S307F	1	AYATTASASTF	11	1	812.62	783.213	0.34	2.3	2.829	0	0	0	0.882	NoExpr	Pending	6	84575234	84575235	84575235	G	A	missense_variant
7-3897221-3897222-G-A		1		Gm14548	S127L	1	SLLAQASPV	3	1	813	1079.17	2.301	2.9	0.65	0	0	0	0.033	NoExpr	Pending	7	3897221	3897222	3897222	G	A	missense_variant
17-26923017-26923018-G-C		2		Kifc5b	E157Q	1	RYREKTQTL	7	2	813.85	1175.03	1.2	2.6	9.958	0	0	0	0.039	NoExpr	Pending	17	26923017	26923018	26923018	G	C	missense_variant
8-43521737-43521738-T-G		1		Adam26b	I76L	1	KNLMSRNLL	9	1	816.75	319.956	2.5	0.9	0	0	0	0	0.079	NoExpr	Pending	8	43521737	43521738	43521738	T	G	missense_variant
11-101411979-101411980-C-A		1	1	Aarsd1	A117S	1	SGQHLITSV	8	2	820.52	851.314	2.301	2.5	34.595	0	0	0	0.014	NoExpr	Pending	11	101411979	101411980	101411980	C	A	missense_variant
7-31375891-31375893-GA-CT		1		Scgb1b3	E56L	1	EYVKQYKDDPL	11	1	821.69	3454.02	2.5	9.2	0	0	0	0	0.055	NoExpr	Pending	7	31375891	31375893	31375892	GA	CT	missense_variant
5-98737674-98737675-G-A		1	1	Cfap299	G147E	1	EDFEVYFNE	9	2	821.92	1761.29	3	5.3	0	0	0	0	0.395	NoExpr	Pending	5	98737674	98737675	98737675	G	A	missense_variant
14-121912246-121912247-G-C		1		Gpr18	A122G	1	RYMGIVQPK	4	1	826.51	302.91	1.2	0.75	0.476	0	0	0	0.107	NoExpr	Pending	14	121912246	121912247	121912247	G	C	missense_variant
5-15580673-15580674-A-C		1		Gm21083	N159T	1	SRLLMEETL	8	1	826.53	1476.55	1.9	3.8	0	0	0	0	0.168	NoExpr	Pending	5	15580673	15580674	15580674	A	C	missense_variant
15-58182880-58182881-C-A		1		Fbxo32	G311V	1	CYPRREQYV	9	1	827.47	4635.43	1.5	8.4	0.461	0	0	0	0.378	NoExpr	Pending	15	58182880	58182881	58182881	C	A	missense_variant
1-85089561-85089562-C-T		1		A530032D15Rik	G188R	1	VWAPGRTFL	6	1	828.11	1004.82	2.1	2.9	13.981	0	0	0	0.056	NoExpr	Pending	1	85089561	85089562	85089562	C	T	missense_variant
7-105368574-105368575-C-T		1		Olfr692	T83I	1	IDLGLATSI	1	1	828.44	805.65	1.5	2.2	0	0	0	0	0.543	NoExpr	Pending	7	105368574	105368575	105368575	C	T	missense_variant
17-15544857-15544858-G-C		1		Prdm9	N553K	1	RGFTQKSHL	6	1	828.44	927.385	1.8	2.3	0.937	0	0	0	0.013	NoExpr	Pending	17	15544857	15544858	15544858	G	C	missense_variant
13-4145267-4145268-C-G		1		Akr1c18	A64P	1	IGQPILSKI	4	1	830.35	539.622	2.301	1.8	0.608	0	0	0	0.071	NoExpr	Pending	13	4145267	4145268	4145268	C	G	missense_variant
15-101460176-101460178-CC-TG		2		Krt81	G398A	1	EYQEVMNSKLA	11	2	835.11	845.8	3.301	1.4	0	0	0	0	0.027	NoExpr	Pending	15	101460176	101460178	101460177	CC	TG	missense_variant
2-119070100-119070101-C-G		1		Knl1	S761C	1	CLAGTSKTI	1	1	836.1	570.506	2.1	1.4	4.801	0	0	0	0.273	NoExpr	Pending	2	119070100	119070101	119070101	C	G	missense_variant
12-115208227-115208229-CC-TT		2		Ighv1-55	G73N	1	IYPNSGSTNYN	4	2	837.07	2522.78	2.9	8.6	0	0	0	0	0.024	NoExpr	Pending	12	115208227	115208229	115208228	CC	TT	missense_variant
16-48565644-48565645-G-T		1		Morc1	D544Y	1	KYERERQLQ	2	1	838.03	35288.6	2.9	50	0	0	0	0	0.167	NoExpr	Pending	16	48565644	48565645	48565645	G	T	missense_variant
4-101909336-101909337-G-T		1		Gm12800	A73S	1	SGVEMLQAV	1	1	839.63	1607.9	2.5	4	0	0	0	0	0.026	NoExpr	Pending	4	101909336	101909337	101909337	G	T	missense_variant
9-40782560-40782561-C-T		1		Clmp	T358I	1	VHHTTLTKAEI	11	1	842.24	14403.3	1	15	0.912	0	0	0	0.304	NoExpr	Pending	9	40782560	40782561	40782561	C	T	missense_variant
8-40999693-40999694-C-T		1		Mtus1	M1019I	1	SEIKKSHEI	9	1	845.45	3206.93	2.5	8.1	3.232	0	0	0	0.663	NoExpr	Pending	8	40999693	40999694	40999694	C	T	missense_variant
1-85484519-85484520-C-T		1		AC147806.1	R208Q	1	IYREGKQAGHF	7	1	846.99	1249.17	3.9	2.5	0	0	0	0	0.084	NoExpr	Pending	1	85484519	85484520	85484520	C	T	missense_variant
1-84964290-84964291-G-A		1		AC167036.1	R208Q	1	IYREGKQAGHF	7	1	846.99	1249.17	3.9	2.5	0	0	0	0	0.023	NoExpr	Pending	1	84964290	84964291	84964291	G	A	missense_variant
10-129238885-129238886-A-C		1		Olfr774	I246L	1	SHMIVLSI	6	1	847.12	1143.76	0.91	1.3	0	0	0	0	0.041	NoExpr	Pending	10	129238885	129238886	129238886	A	C	missense_variant
17-34907588-34907589-A-G		1		Ehmt2	N782S	1	SHLEVARYM	1	1	848.69	1995.68	2	3.2	42.236	0	0	0	0.14	NoExpr	Pending	17	34907588	34907589	34907589	A	G	missense_variant
11-65989859-65989860-T-G		1	1	Dnah9	N2637T	1	KFGTFPTTL	4	2	849.36	1132.64	2.5	3.2	0	0	0	0	0.156	NoExpr	Pending	11	65989859	65989860	65989860	T	G	missense_variant
4-116337488-116337489-G-A		1		Mast2	P202S	1	HGHTGNSSL	8	1	849.36	1393.99	2.5	2.1	19.608	0	0	0	0.258	NoExpr	Pending	4	116337488	116337489	116337489	G	A	missense_variant
3-138286841-138286842-G-C		1		Adh1	E240Q	1	KELGATQCI	7	1	849.69	2905.76	2.5	5.2	0.296	0	0	0	0.048	NoExpr	Pending	3	138286841	138286842	138286842	G	C	missense_variant
7-12041929-12041930-A-T		1		Vmn1r77	K211I	1	LYIHKRQVRNI	3	1	849.8	1806.68	0.31	3.3	0	0	0	0	0.057	NoExpr	Pending	7	12041929	12041930	12041930	A	T	missense_variant
12-114708836-114708837-G-C		1		Ighv1-19	N54K	1	YYMKWVKQS	4	1	855.38	877.183	1.4	2.6	0	0	0	0	0.03	NoExpr	Pending	12	114708836	114708837	114708837	G	C	missense_variant
1-66473470-66473471-A-C		1		Unc80	N180T	1	TFQTSMATV	4	1	855.38	784.45	4.199	3.9	0	0	0	0	0.208	NoExpr	Pending	1	66473470	66473471	66473471	A	C	missense_variant
1-88355257-88355258-C-G		1		Trpm8	L756V	1	AYVVLMDFHSV	4	1	855.85	1287.89	1.2	1.8	0	0	0	0	0.199	NoExpr	Pending	1	88355257	88355258	88355258	C	G	missense_variant
14-50414141-50414142-T-G		1		Olfr738	I199M	1	SLMEMTSST	3	1	857.55	549.87	2.4	1.8	0	0	0	0	0.043	NoExpr	Pending	14	50414141	50414142	50414142	T	G	missense_variant
1-116184441-116184442-T-G		1	1	Cntnap5a	S487R	1	IYSGKRYYF	6	2	858.68	1101.11	3.301	4.2	0	0	0	0	0.215	NoExpr	Pending	1	116184441	116184442	116184442	T	G	missense_variant
6-136400878-136400879-A-G		1		E330021D16Rik	S318P	1	SIEPVILQI	4	1	864.68	953.119	4.301	4.8	0	0	0	0	0.887	NoExpr	Pending	6	136400878	136400879	136400879	A	G	missense_variant
3-9264685-9264686-T-G		1		Zbtb10	F368C	1	KICKAHKNI	3	1	864.68	476.884	3.301	1.8	0.123	0	0	0	0.302	NoExpr	Pending	3	9264685	9264686	9264686	T	G	missense_variant
7-10088027-10088028-C-T		1	1	Vmn2r51	V577I	1	CIQKGITFL	6	1	865.15	875.165	2.199	2.3	0	0	0	0	0.043	NoExpr	Pending	7	10088027	10088028	10088028	C	T	missense_variant
17-37151436-37151437-T-G		1		Olfr93	E178D	1	FLCDVPSLI	4	1	865.49	813.317	2.5	2	0	0	0	0	0.19	NoExpr	Pending	17	37151436	37151437	37151437	T	G	missense_variant
6-120952378-120952379-G-C		1		Mical3	A1842G	1	KRLHRGQII	6	1	867.14	814.873	2.699	2.6	1.482	0	0	0	0.394	NoExpr	Pending	6	120952378	120952379	120952379	G	C	missense_variant

11-71156168-71156169-G-A		1		Nlrp1b	A1122V	1	SYEVVRAEN	4	1	867.14	468.911	2.5	1.4	2.122	0	0	0	0.034	NoExpr	Pending	11	71156168	71156169	71156169	G	A	missense_variant
7-8472031-8472032-G-C		1		Vmn2r45	L666V	1	VFTVAVSTV	6	1	869.7	845.454	1.3	0.9	0	0	0	0	0.025	NoExpr	Pending	7	8472031	8472032	8472032	G	C,A	missense_variant
14-69800781-69800782-G-C		1		Rhobtb2	P10A	1	DYERANVETI	5	1	869.88	1163.16	0.7	0.7	2.258	0	0	0	0.077	NoExpr	Pending	14	69800781	69800782	69800782	G	C	missense_variant
17-35383075-35383076-A-T		2		H2-Q4	N305Y	1	YYTCHVHHEGL	1	2	870.38	1686.38	2.6	4.8	130.46	0	0	0	0.059	NoExpr	Pending	17	35383075	35383076	35383076	A	T	missense_variant
2-88487514-88487515-C-T		1		Olfr1184	T261I	1	IFAYLRPPI	9	1	871.49	14726.2	2.301	19	0	0	0	0	0.404	NoExpr	Pending	2	88487514	88487515	88487515	C	T	missense_variant
2-86255419-86255420-T-G		1		Olfr1049	N91T	1	NFVVDKNTI	8	1	873.15	1791.68	2.6	4.6	0	0	0	0	0.032	NoExpr	Pending	2	86255419	86255420	86255420	T	G	missense_variant
7-12321454-12321455-A-C		1		Vmn1r83	L225R	1	KHIHSAQQR	9	1	873.49	119.47	3.301	0.36	0	0	0	0	0.203	NoExpr	Pending	7	12321454	12321455	12321455	A	C	missense_variant
14-79096447-79096448-C-T		1		Vwa8	S1346L	1	LTLAVGQKI	3	1	873.49	1101.77	2.6	3.1	7.582	0	0	0	0.067	NoExpr	Pending	14	79096447	79096448	79096448	C	T	missense_variant
10-130034453-130034454-G-A		1		Olfr821	S276N	1	KGIALLNTS	7	1	874.09	375.868	4.301	1.9	0	0	0	0	0.664	NoExpr	Pending	10	130034453	130034454	130034454	G	A	missense_variant
4-132833090-132833091-G-A		1		Ppp1r8	R100W	1	IWLEPHKPQQI	2	1	874.09	1785.2	1.6	3.5	35.562	0	0	0	0.079	NoExpr	Pending	4	132833090	132833091	132833091	G	A	missense_variant
13-8836797-8836798-C-T		1		Wdr37	V318I	1	LYDVETSELI	10	1	875.91	2192.64	0.8	0.81	4.943	0	0	0	0.606	NoExpr	Pending	13	8836797	8836798	8836798	C	T	missense_variant
16-13697428-13697429-T-C		1		Bfar	C294R	1	RFLPFIHTI	1	1	876.7	376.56	1.2	1.1	10.388	0	0	0	0.047	NoExpr	Pending	16	13697428	13697429	13697429	T	C	missense_variant
1-126025950-126025951-C-T		1		Nckap5	A1023T	1	HHALGTTHM	7	1	876.96	3937.4	3.1	14	0.009	0	0	0	0.499	NoExpr	Pending	1	126025950	126025951	126025951	C	T	missense_variant
12-114896432-114896434-AA-GT		2		Ighv1-37	F52Y	1	YYMNWVKQS	2	2	877.18	8032.38	2.6	18	0	0	0	0	0.015	NoExpr	Pending	12	114896432	114896434	114896433	AA	GT	missense_variant
13-63511819-63511820-A-C		1		Ptch1	S1340A	1	TATAMGSSV	2	1	877.18	743.173	2.301	2.2	4.296	0	0	0	0.305	NoExpr	Pending	13	63511819	63511820	63511820	A	C	missense_variant
X-74303841-74303842-G-A		1		Atp6ap1	D410N	1	SYASNCAGF	5	1	877.53	1535.54	2.6	4.2	115.79	0	0	0	0.042	NoExpr	Pending	X	74303841	74303842	74303842	G	A	missense_variant
7-141638885-141638886-G-A		1		Muc6	P1958L	1	LHLSQPSTV	1	1	880.15	3296.78	1.6	4.6	0.024	0	0	0	0.133	NoExpr	Pending	7	141638885	141638886	141638886	G	A	missense_variant
15-30647189-30647190-A-G		1		Ctnnd2	Q344R	1	PIHRLSSTI	4	1	881.63	1208.06	2.1	2.3	0.104	0	0	0	0.111	NoExpr	Pending	15	30647189	30647190	30647190	A	G	missense_variant
1-85635574-85635575-A-C		1		Sp140	N330T	1	NFLPTPPRI	5	1	881.91	1663.76	3.4	7.3	54.864	0	0	0	0.027	NoExpr	Pending	1	85635574	85635575	85635575	A	C	missense_variant
12-53947199-53947200-A-G		1		Npas3	H204R	1	DYVRPGDHV	4	1	882.45	1906.3	3.6	3.9	0.257	0	0	0	0.572	NoExpr	Pending	12	53947199	53947200	53947200	A	G	missense_variant
16-32754904-32754905-C-T		1		Muc4	A1593V	1	SHVPSMSSS	3	1	883.6	437.342	2.4	1.2	0.018	0	0	0	0.05	NoExpr	Pending	16	32754904	32754905	32754905	C	T	missense_variant
18-36940296-36940297-C-T		2		Pcdha2	T327I	1	KGIPAMSGH	3	2	883.6	2314.53	4.398	12	0	0	0	0	0.14	NoExpr	Pending	18	36940296	36940297	36940297	C	T	missense_variant
6-71963681-71963682-T-G		1		Polr1a	I1000S	1	GYLQRCISKHL	8	1	884.87	884.871	1.2	1.4	15.468	0	0	0	0.238	NoExpr	Pending	6	71963681	71963682	71963682	T	G	missense_variant
2-112092469-112092470-G-A		1	1	Olfr1314	T77I	1	ICPKMIYDL	1	2	885.12	1569.75	2.6	4.6	0	0	0	0	0.263	NoExpr	Pending	2	112092469	112092470	112092470	G	A	missense_variant
11-59122858-59122859-C-T		1		Obscn	R1054H	1	KEQQAHSEV	6	1	885.3	807.402	2.301	2	0.384	0	0	0	0.046	NoExpr	Pending	11	59122858	59122859	59122859	C	T	missense_variant
12-110665717-110665718-T-C		1		Dync1h1	I4474T	1	HYTVPAGMTVT	11	1	887.05	539.18	2.199	0.21	19.21	0	0	0	0.104	NoExpr	Pending	12	110665717	110665718	110665718	T	C	missense_variant
10-40647594-40647595-C-T		1		Ddo	P194S	1	RRLVGDSMI	7	1	887.34	7400.48	2.4	14	0	0	0	0	0.034	NoExpr	Pending	10	40647594	40647595	40647595	C	T	missense_variant
18-42228741-42228742-G-C		1	1	Lars	T600R	1	IESLSDSRI	8	2	889.39	439.805	2.6	1.3	111.96	0	0	0	0.035	NoExpr	Pending	18	42228741	42228742	42228742	G	C	missense_variant
2-129630101-129630102-T-G		1		Sirpa	V487G	1	TYADLDMGHLS	8	1	890.66	884.525	2.699	2.8	19.421	0	0	0	0.237	NoExpr	Pending	2	129630101	129630102	129630102	T	G	missense_variant
12-115803066-115803067-C-T		2		Ighv1-74	C5Y	1	SYIILFLVATA	2	2	891.01	9759.45	2.6	18	0	0	0	0	0.013	NoExpr	Pending	12	115803066	115803067	115803067	C	T	missense_variant
8-123208079-123208080-G-C		1		Chmp1a	R46G	1	AGVYAENAI	2	1	891.79	855.579	2.6	2.5	50.203	0	0	0	0.036	NoExpr	Pending	8	123208079	123208080	123208080	G	C	missense_variant
X-164533551-164533552-A-G		1		Asb9	N173D	1	LYVACKDQQV	7	1	892.2	869.881	0.86	0.7	0	0	0	0	0.125	NoExpr	Pending	X	164533551	164533552	164533552	A	G	missense_variant
9-57136708-57136709-C-A		1		Man2c1	F268L	1	CHIDTAWLWPL	11	1	893.21	2390.89	0.86	2.9	11.168	0	0	0	0.061	NoExpr	Pending	9	57136708	57136709	57136709	C	A	missense_variant
15-85559099-85559100-G-C		1		Wnt7b	A38G	1	SVVALGGNI	7	1	893.21	2069.59	1.8	3.3	0.078	0	0	0	0.086	NoExpr	Pending	15	85559099	85559100	85559100	G	C	missense_variant
16-87722516-87722517-G-A		1		Bach1	R565H	1	IHRRSKNRI	2	1	893.49	1381.21	2.6	3.8	7.293	0	0	0	0.272	NoExpr	Pending	16	87722516	87722517	87722517	G	A	missense_variant
12-75966729-75966730-T-G		1		Syne2	F2898L	1	AYKDANVLERL	8	1	897.18	897.181	1.2	1.2	13.111	0	0	0	0.076	NoExpr	Pending	12	75966729	75966730	75966730	T	G	missense_variant
11-57769625-57769626-A-G		1		Galnt10	I297V	1	MYYKRVPI	6	1	898.67	1022.35	0.7	0.8	4.522	0	0	0	0.587	NoExpr	Pending	11	57769625	57769626	57769626	A	G	missense_variant
10-90049876-90049877-C-T		1		Anks1b	A145V	1	QYGHSEVVVVL	9	1	899.25	546.651	3.699	1.4	2.768	0	0	0	0.641	NoExpr	Pending	10	90049876	90049877	90049877	C	T	missense_variant
17-23345177-23345178-G-C		1		Vmn2r115	W108S	1	SESKLMSFM	1	1	899.68	1710.37	2.6	4.6	0	0	0	0	0.391	NoExpr	Pending	17	23345177	23345178	23345178	G	C	missense_variant
2-113525520-113525521-A-C		1		Fmn1	K867T	1	TSSSSQHTI	8	1	901.76	2958.63	2.1	6.4	1.999	0	0	0	0.125	NoExpr	Pending	2	113525520	113525521	113525521	A	C	missense_variant
9-106463368-106463369-G-A		1		Pcbp4	A388T	1	YTAKMATAN	7	1	902.93	4106.84	4.5	18	42.635	0	0	0	0.733	NoExpr	Pending	9	106463368	106463369	106463369	G	A	missense_variant
7-7312845-7312846-T-A		1		Vmn2r30	T663S	1	FSVAVSTVL	2	1	905.92	1271.34	2.199	2.7	0	0	0	0	0.31	NoExpr	Pending	7	7312845	7312846	7312846	T	A	missense_variant
7-132759533-132759534-G-A		1		Fam53b	P255L	1	ANSTPASTL	9	1	908.01	8205.22	2.6	18	3.813	0	0	0	0.466	NoExpr	Pending	7	132759533	132759534	132759534	G	A	missense_variant
1-118662019-118662020-C-T		1		Tfcp2l1	T270I	1	NNIPSPSYN	3	1	912.2	2371.87	2.699	6.2	0.041	0	0	0	0.176	NoExpr	Pending	1	118662019	118662020	118662020	C	T	missense_variant
11-4672397-4672398-G-A		1		Ascc2	C488Y	1	AYLEHYSYDSE	2	1	912.75	17695.7	2.801	27	18.094	0	0	0	0.055	NoExpr	Pending	11	4672397	4672398	4672398	G	A	missense_variant
15-47847206-47847207-G-T		1		Csmd3	S1661R	1	SFQDRKLPERI	5	1	912.75	555.751	1.6	0.8	0.005	0	0	0	0.027	NoExpr	Pending	15	47847206	47847207	47847207	G	T	missense_variant
14-50414145-50414146-A-C		1		Olfr738	M201L	1	IELTSSTLSSL	3	1	912.75	1076.17	2.1	2.3	0	0	0	0	0.035	NoExpr	Pending	14	50414145	50414146	50414146	A	C	missense_variant
1-63592636-63592637-C-T		1		Adam23	T824I	1	RRFDPIQQGPI	6	1	913.86	2659.99	3	3	0.076	0	0	0	0.607	NoExpr	Pending	1	63592636	63592637	63592637	C	T	missense_variant
8-105375702-105375703-A-G		1		Plekhg4	N108S	1	AFSELESLL	7	1	914.66	2405.8	2.5	4.7	0.081	0	0	0	0.192	NoExpr	Pending	8	105375702	105375703	105375703	A	G	missense_variant
1-51755732-51755733-C-T		1		Myo1b	S1066N	1	FLFNSDHLI	4	1	918.88	684.321	2.1	1.4	2.526	0	0	0	0.29	NoExpr	Pending	1	51755732	51755733	51755733	C	T	missense_variant
7-26611682-26611683-G-C		1		Vmn1r185	H132Q	1	AIMQNGCSI	4	1	918.88	1507.27	2.699	3.9	0	0	0	0	0.043	NoExpr	Pending	7	26611682	26611683	26611683	G	C	missense_variant
7-85620587-85620588-A-T		1	1	Vmn2r71	I436F	1	FDSWKMFSF	9	2	920.09	2772.24	3.1	6	0	0	0	0	0.06	NoExpr	Pending	7	85620587	85620588	85620588	A	T	missense_variant
17-35264047-35264048-G-A		2		H2-D1	E185K	1	AYLKGECVE	4	2	921	2513.39	2.699	6.5	1154.2	0	0	0	0.03	NoExpr	Pending	17	35264047	35264048	35264048	G	A	missense_variant
8-3876306-3876307-C-T		1		Cd209d	A133T	1	WHNSTTTCQEL	7	1	922.68	1378.08	1.8	2.8	0.18	0	0	0	0.221	NoExpr	Pending	8	3876306	3876307	3876307	C	T	missense_variant
2-160957180-160957181-G-A		1		Chd6	P2328L	1	AGQTTLSTTHL	11	1	922.68	7208.66	1.7	14	3.328	0	0	0	0.047	NoExpr	Pending	2	160957180	160957181	160957181	G	A	missense_variant
17-35264039-35264040-C-A		1		H2-D1	A182D	1	DYLEGECVEWL	1	1	922.68	344.702	1.7	0.6	1154.2	0	0	0	0.037	NoExpr	Pending	17	35264039	35264040	35264040	C	A	missense_variant
14-53073258-53073259-T-C		1		Trav13d-4	F106L	1	SSQTTDSGTYL	11	1	922.68	2469.77	1.7	4.8	0	0	0	0	0.035	NoExpr	Pending	14	53073258	53073259	53073259	T	C	missense_variant
7-7312964-7312965-C-T		1		Vmn2r30	S623N	1	ANNRNLSYL	5	1	927.39	316.293	2.699	1	0	0	0	0	0.375	NoExpr	Pending	7	7312964	7312965	7312965	C	T	missense_variant
X-74303875-74303876-T-C		1	1	Atp6ap1	M421T	1	SPGIWTGLL	6	2	928.97	1035.33	3.199	2.8	115.79	0	0	0	0.044	NoExpr	Pending	X	74303875	74303876	74303876	T	C	missense_variant
17-37624371-37624373-CC-GT		1		Olfr116	AG87-88AR	1	SSLARSGYI	5	1	929.16	388.269	2.699	1.2	0	0	0	0	0.028	NoExpr	Pending	17	37624371	37624373	37624372	CC	GT	missense_variant
18-80972970-80972971-C-T		1		Sall3	E581K	1	GLNNTKSGI	6	1	929.16	1143.12	2.699	3.2	0	0	0	0	0.365	NoExpr	Pending	18	80972970	80972971	80972971	C	T	missense_variant
3-146001982-146001983-C-G		1		Syde2	A826G	1	CGIQVDSVN	2	1	929.16	1919.07	2.801	5.8	0.011	0	0	0	0.029	NoExpr	Pending	3	146001982	146001983	146001983	C	G	missense_variant
1-90214837-90214838-C-T		1		Ackr3	L340F	1	KYSAKTGFTKL	8	1	930.12	1139.58	0.7	0.7	31.594	0	0	0	0.689	NoExpr	Pending	1	90214837	90214838	90214838	C	T	missense_variant
6-88884689-88884690-T-G		1		Mcm2	M814L	1	KFSVMRSLR	8	1	931.3	736.36	2.699	2.9	54.411	0	0	0	0.051	NoExpr	Pending	6	88884689	88884690	88884690	T	G	missense_variant
7-22871382-22871383-A-C		1		Vmn1r160	I54L	1	SKQRPRQVL	9	1	931.67	319.345	3.699	1.4	0	0	0	0	0.013	NoExpr	Pending	7	22871382	22871383	22871383	A	C	missense_variant

2-20766469-20766470-G-C		1		Etl4	K671N	1	AHVSAGNVL	7	1	933.75	1864.16	3.5	7.7	6.769	0	0	0	0.062	NoExpr	Pending	2	20766469	20766470	20766470	G	C	missense_variant
1-85610765-85610766-G-A		1		Sp140	V75I	1	EYQETCKNLIP	10	1	937.57	2427.98	1.6	2.6	54.864	0	0	0	0.01	NoExpr	Pending	1	85610765	85610766	85610766	G	A,C	missense_variant
11-51593953-51593954-A-C		1		Phykpl	E247A	1	GGLFVADAI	8	1	939.92	2046.87	2.699	4.6	6.792	0	0	0	0.182	NoExpr	Pending	11	51593953	51593954	51593954	A	C	missense_variant
7-103328662-103328663-A-G		1		Olfr598	H59R	1	IIIPAERSL	7	1	940.29	553.681	2.699	1.8	0	0	0	0	0.087	NoExpr	Pending	7	103328662	103328663	103328663	A	G	missense_variant
10-58224914-58224915-A-C		1		AW822073	W6G	1	WQAGQEQAL	4	1	942.45	1285.55	2.699	3.1	0	0	0	0	0.029	NoExpr	Pending	10	58224914	58224915	58224915	A	C	missense_variant
5-136059811-136059812-C-T		1		Upk3bl	P55L	1	STFTLEQLL	8	1	942.45	740.048	2.699	2.2	11.824	0	0	0	0.516	NoExpr	Pending	5	136059811	136059812	136059812	C	T	missense_variant
7-7312838-7312839-G-C		1		Vmn2r30	A665G	1	VFTVGVSTV	5	1	942.45	869.7	1.8	1.3	0	0	0	0	0.3	NoExpr	Pending	7	7312838	7312839	7312839	G	C	missense_variant
17-35439698-35439699-C-A		1		H2-Q7	S104R	1	RYYNQSKGGSH	1	1	942.86	1006.1	1.7	1.8	3.606	0	0	0	0.157	NoExpr	Pending	17	35439698	35439699	35439699	C	A	missense_variant
1-85610751-85610752-C-T		1		Sp140	T70I	1	EYQEICKNLV	5	1	942.86	704.109	1.7	1	54.864	0	0	0	0.011	NoExpr	Pending	1	85610751	85610752	85610752	C	T	missense_variant
3-94343347-94343348-T-G		1		Them5	F73V	1	LYQEVLEKTKS	5	1	942.86	3454.02	1.7	6.7	0.016	0	0	0	0.147	NoExpr	Pending	3	94343347	94343348	94343348	T	G	missense_variant
9-73932847-73932848-C-T		1		Unc13c	M240I	1	CISQTHDVI	9	1	948.62	3598.24	3	9.2	3.369	0	0	0	0.303	NoExpr	Pending	9	73932847	73932848	73932848	C	T	missense_variant
2-76753052-76753053-C-T		1		Ttn	E20753K	1	KHQKVGDDAW	1	1	950.34	3033.45	2.9	5.8	0.056	0	0	0	0.435	NoExpr	Pending	2	76753052	76753053	76753053	C	T	missense_variant
6-5168386-5168387-T-A		1		Pon1	K340I	1	KGILLIGTV	3	1	951.17	6224.29	2.199	15	0	0	0	0	0.08	NoExpr	Pending	6	5168386	5168387	5168387	T	A	missense_variant
2-88486836-88486837-C-T		1		Olfr1184	A35V	1	CYLVILCGNLL	4	1	952.24	1987.97	1.5	0.7	0	0	0	0	0.426	NoExpr	Pending	2	88486836	88486837	88486837	C	T	missense_variant
17-13883818-13883819-G-C		1		Afdn	G1139A	1	LYNNSAQNA	9	1	952.99	809.264	1.5	2.5	17.952	0	0	0	0.086	NoExpr	Pending	17	13883818	13883819	13883819	G	C	missense_variant
8-110519286-110519287-A-G		1		Hydin	T2211A	1	APQVQISSSPL	1	1	953.12	1183.39	4.102	3.6	0	0	0	0	0.272	NoExpr	Pending	8	110519286	110519287	110519287	A	G	missense_variant
11-58529827-58529828-C-T		1		Olfr330	V53I	1	LLILSDTHL	3	1	953.37	4626.58	2.699	6.5	0	0	0	0	0.028	NoExpr	Pending	11	58529827	58529828	58529828	C	T	missense_variant
11-115205126-115205127-G-C		2		Tmem104	A165P	1	IYAPAVPV	4	2	955.21	823	1.3	0.79	2.643	0	0	0	0.072	NoExpr	Pending	11	115205126	115205127	115205127	G	C	missense_variant
17-35266194-35266195-C-T		1		H2-D1	P302S	1	SSTDSYMVI	1	1	957.39	9910.37	2.801	20	1154.2	0	0	0	0.028	NoExpr	Pending	17	35266194	35266195	35266195	C	T	missense_variant
11-76076501-76076502-C-T		1		Vps53	S600N	1	FSTVISNSI	7	1	957.39	451.95	2.6	1.3	13.886	0	0	0	0.279	NoExpr	Pending	11	76076501	76076502	76076502	C	T	missense_variant
2-148700412-148700413-G-A		1		Napb	A209V	1	FKVALCHFI	3	1	959.6	484.273	2.1	1	1.417	0	0	0	0.443	NoExpr	Pending	2	148700412	148700413	148700413	G	A	missense_variant
X-73259107-73259108-A-C		1		Xlr3c	H138Q	1	KQAETLSNM	2	1	961.81	500.27	2.801	1.2	0.003	0	0	0	0.03	NoExpr	Pending	X	73259107	73259108	73259108	A	C	missense_variant
7-56328748-56328749-G-A		1		Oca2	R555H	1	IHVWRLTAQHI	10	1	963.49	1318.61	1	1	0	0	0	0	0.2	NoExpr	Pending	7	56328748	56328749	56328749	G	A	missense_variant
7-46079510-46079511-G-C		1		Nomo1	E1073D	1	LYKSDSLDNPI	5	1	969.97	963.47	1.1	0.77	32.813	0	0	0	0.037	NoExpr	Pending	7	46079510	46079511	46079511	G	C	missense_variant
2-110745913-110745914-T-G		1		Ano3	Q431H	1	NESQVSHEI	7	1	970.71	620.998	2.801	2	0.004	0	0	0	0.384	NoExpr	Pending	2	110745913	110745914	110745914	T	G	missense_variant
10-130210347-130210348-T-G		1		Olfr827	S261R	1	SHLGVVRLL	7	1	970.71	305.14	2.801	0.8	0	0	0	0	0.225	NoExpr	Pending	10	130210347	130210348	130210348	T	G	missense_variant
5-75163746-75163747-G-A		1		Pdgfra	V88I	1	LFVTVLEVI	9	1	971.09	3900.23	1.4	5.2	7.715	0	0	0	0.602	NoExpr	Pending	5	75163746	75163747	75163747	G	A	missense_variant
12-114896418-114896419-T-A		1		Ighv1-37	K57M	1	GYFMNWVM	8	1	971.54	994.489	0.8	1.4	0	0	0	0	0.085	NoExpr	Pending	12	114896418	114896419	114896419	T	A	missense_variant
2-98662301-98662303-TA-AA		1		Gm10801	SR22-23RR	1	RRCFSFSMI	1	1	972.95	239.933	2.199	0.8	0.12	0	0	0	0.055	NoExpr	Pending	2	98662301	98662303	98662302	TA	AA,GG	missense_variant
18-61245580-61245581-G-C		1		Pde6a	G325A	1	DYILHAKEDI	6	1	974.18	1477.88	0.9	1.2	0.02	0	0	0	0.052	NoExpr	Pending	18	61245580	61245581	61245581	G	C	missense_variant
6-57926211-57926212-T-A		1		Vmn1r23	T194S	1	VGVMLTSSI	7	1	975.57	1521.46	2.5	3.5	0	0	0	0	0.252	NoExpr	Pending	6	57926211	57926212	57926212	T	A	missense_variant
4-147510858-147510860-AC-TA		1		Zfp982	Y46L	1	LYIDVMLENL	10	1	983.2	4174.84	0.9	7.3	0.99	0	0	0	0.074	NoExpr	Pending	4	147510858	147510860	147510859	AC	TA	missense_variant
9-31161300-31161301-G-A		1		Aplp2	R504C	1	RYVRAENKDCL	10	1	983.74	948.156	1.3	0.93	75.728	0	0	0	0.043	NoExpr	Pending	9	31161300	31161301	31161301	G	A	missense_variant
4-68792849-68792850-G-A		1		Brinp1	P374S	1	CRHNSNHQL	5	1	984.22	3244.07	2.801	7.5	0.017	0	0	0	0.514	NoExpr	Pending	4	68792849	68792850	68792850	G	A	missense_variant
3-122549201-122549202-G-T		1		Fnbp1l	R410S	1	RRKKLQQSI	8	1	984.22	2153.23	2.801	5.8	2.47	0	0	0	0.071	NoExpr	Pending	3	122549201	122549202	122549202	G	T	missense_variant
X-60293649-60293650-C-T		1		Atp11c	G223R	1	YRFVRRISI	5	1	984.56	466.675	2.199	0.8	8.298	0	0	0	0.475	NoExpr	Pending	X	60293649	60293650	60293650	C	T	missense_variant
2-132879658-132879659-G-C		1		Lrrn4	C79W	1	SLESLPSWL	8	1	984.56	1290.38	2.801	3.5	0	0	0	0	0.115	NoExpr	Pending	2	132879658	132879659	132879659	G	C	missense_variant
7-25331086-25331087-C-G		1		Megf8	C488W	1	FYHLGWHQWV	6	1	989.6	613.42	1	0.57	9.057	0	0	0	0.077	NoExpr	Pending	7	25331086	25331087	25331087	C	G	missense_variant
9-112136557-112136558-C-T		1		Arpp21	G415E	1	AYSENEMGGQY	6	1	990.56	1015.57	1.8	2.1	0	0	0	0	0.512	NoExpr	Pending	9	112136557	112136558	112136558	C	T	missense_variant
2-86217338-86217339-C-T		1		Olfr1046	D124N	1	NRYVAICKPLL	1	1	992.45	978.836	3.1	2.9	0	0	0	0	0.047	NoExpr	Pending	2	86217338	86217339	86217339	C	T	missense_variant
15-36234652-36234653-A-T		1		Spag1	K853I	1	LYLSIAERFK	5	1	995.27	2646.31	1.8	5.8	0.711	0	0	0	0.221	NoExpr	Pending	15	36234652	36234653	36234653	A	T	missense_variant
X-136734410-136734411-T-G		1		Morf4l2	K23Q	1	FKQPTRSNM	3	1	996	3786.69	2.1	7	259.32	0	0	0	0.055	NoExpr	Pending	X	136734410	136734411	136734411	T	G	missense_variant

Supplementary Table 5. The shared neoantigens between CT26 and CT26-iPSC vaccined tumors.

ID	H-2-Kd	Gene	biotype	AA Change	IC50 WT	IC50 MT
7-4834243-4834244-T-G	4	Shisa7	missense_variant	K274T	336.38	199.77
5-61809303-61809304-G-T	2	G6pd2	missense_variant	A141S	101.499	109.485
9-70125150-70125151-G-C	2	Fam81a	missense_variant	P20A	3972.739	679.344
12-115242852-115242853-C-A	2	Ighv1-56	missense_variant	M100I	123.905	102.351
9-21230766-21230767-C-T	1	Keap1	missense_variant	V604M	2276.46	731.577
7-40993293-40993294-A-C	1	4930433I11Rik	missense_variant	D129A	1145.75	792.984
9-40100652-40100653-G-A	1	Olfr984	missense_variant	T279I	235.45	49.49
15-89190248-89190249-C-G	2	Dennd6b	missense_variant	A122P	772.64	576.23
8-48276524-48276525-G-C	1	Tenm3	missense_variant	A1482G	308.503	433.601
11-71156153-71156154-T-G	1	Nlrp1b	missense_variant	N1127T	468.911	417.917
19-5892550-5892551-C-T	1	Tigd3	missense_variant	A184T	487.63	673.38
1-85610751-85610752-C-T	1	Sp140	missense_variant	T70I	704.109	942.862
X-79355059-79355060-A-C	1	Cfap47	missense_variant	L2332R	104.014	302.754
2-63980415-63980416-C-A	1	Fign	missense_variant	S170I	688.934	42.252
4-147678235-147678237-GT-TA	1	Zfp534	missense_variant	Y46L	3440.646	652.08
7-43815408-43815409-A-C	1	Klk7	missense_variant	K236T	765.755	121.98
17-13073603-13073604-G-A	1	Tcp10b	missense_variant	D305N	579.202	414
1-171699598-171699599-C-T	2	Cd48	missense_variant	P198S	371.612	263.081
10-90049876-90049877-C-T	1	Anks1b	missense_variant	A145V	546.651	899.249
2-98667255-98667256-G-T	1	Gm10800	missense_variant	R16S	2198.63	793.07
19-53635751-53635752-A-C	2	Smc3	missense_variant	D733A	156.84	250.7
6-40464143-40464144-T-G	1	Wee2	missense_variant	M461R	79.632	82.241
17-36165685-36165686-A-C	1	Gm8909	missense_variant	S271A	591.916	395.603
12-32205628-32205629-C-T	2	Pik3cg	missense_variant	D120N	461.61	571.097
16-43969362-43969363-C-T	1	Zdhhc23	missense_variant	G354D	209.329	188.725
9-39184494-39184495-T-C	1	Olfr943	missense_variant	F106L	44.857	125.265
10-130210776-130210777-G-A	1	Olfr827	missense_variant	L118F	3000.805	123.322
6-73044834-73044835-C-A	1	Dnah6	missense_variant	E3501D	833.451	639.558
4-139779672-139779673-C-T	1	Pax7	missense_variant	G351E	853.277	736.36
7-9986483-9986484-G-T	2	Vmn2r49	missense_variant	S360Y	5418.512	493.08
5-65825383-65825384-C-G	3	N4bp2	missense_variant	S1660R	88.973	313.71
19-11472453-11472454-C-T	1	Ms4a6c	missense_variant	P70L	6840.534	756.99
11-74206597-74206598-A-G	1	Olfr43	missense_variant	F206S	251.868	108.009
4-88835957-88835958-A-T	2	Ifna5	missense_variant	K145I	1637.176	482.072
5-15529187-15529188-A-T	1	Gm21190	missense_variant	F11I	9960.015	514.15
2-86347205-86347206-T-G	1	Olfr1055	missense_variant	M187L	636.62	396.762
13-92131447-92131448-G-A	1	Rasgrf2	missense_variant	A16V	2289.621	560.916
1-105678183-105678184-C-T	1	Relch	missense_variant	T183I	298.717	642.821
9-73932847-73932848-C-T	1	Unc13c	missense_variant	M240I	3598.239	948.615
19-13410933-13410934-C-T	1	Olfr1469	missense_variant	R122C	38.53	24.071
9-89603089-89603090-C-G	1	Minar1	missense_variant	A85P	206.933	216.465
2-87693491-87693492-A-T	1	Olfr1136	missense_variant	L130H	265.461	131.826
1-85591681-85591682-G-T	1	Sp110	missense_variant	P142T	1746.184	548.391
10-83504132-83504133-G-C	1	Aldh1l2	missense_variant	P584A	5903.234	638.396
6-132803745-132803747-GA-TT	2	Tas2r117	missense_variant	PT282-283PS	169.044	118.577
7-26611631-26611632-A-T	1	Vmn1r185	missense_variant	F149L	575.44	674.186
16-32751954-32751956-AC-GA	1	Muc4	missense_variant	N611R	467.832	732.976
2-132879658-132879659-G-C	1	Lrrn4	missense_variant	C79W	1290.382	984.564
1-139733518-139733519-A-C	1	Gm4788	missense_variant	L531V	232.745	221.758
17-37299758-37299759-G-A	3	Olfr101	missense_variant	T221I	38.823	264.399
17-37923941-37923943-CA-TG	1	Olfr128	missense_variant	AI125-126AV	102.603	61.12
4-147178798-147178799-A-G	1	Zfp991	missense_variant	H211R	496.695	703.218

4-86583171-86583172-C-T	2	Haus6	missense_variant	A821T	44.082	78.21
4-145534921-145534923-AC-TA	1	Zfp990	missense_variant	Y46L	3440.646	652.08
10-100064109-100064110-G-C	1	Kitl	missense_variant	M52I	96.79	69.796
5-7978488-7978489-T-G	1	Steap4	missense_variant	F356V	144.82	174.916
14-37092018-37092019-G-A	1	Cdhr1	missense_variant	A150V	274.322	139.988
6-148842863-148842864-G-A	3	Caprin2	missense_variant	S1021F	115.15	65.842
2-98667242-98667243-T-A	1	Gm10800	missense_variant	D20V	5165.234	629.72
1-159324957-159324958-C-G	1	Cop1	missense_variant	P593A	340.97	562.34
10-102544813-102544814-C-T	1	Rassf9	missense_variant	T19I	11805.653	719.598
19-33749463-33749464-G-A	1	Lipo2	missense_variant	P58S	99.83	31.496
3-90349472-90349473-A-T	1	Gatad2b	missense_variant	N196I	1989.193	625.01
1-132046445-132046446-A-G	1	Mfsd4a	missense_variant	V382A	1303.947	711.639
4-156334442-156334443-C-T	1	Vmn2r-ps159	missense_variant	A340V	762.237	133.025
6-86732975-86732976-G-C	2	Gmcl1	missense_variant	D63E	735.143	743.14
12-115294364-115294365-A-G	1	Ighv8-8	missense_variant	S19P	154.202	508.265
16-56260477-56260478-C-G	2	Impg2	missense_variant	P882A	1452.413	729.609
5-137380871-137380872-C-G	1	Zan	missense_variant	G5322A	767.658	674.186
6-84575234-84575235-G-A	1	Cyp26b1	missense_variant	S307F	783.213	812.62
1-88355257-88355258-C-G	1	Trpm8	missense_variant	L756V	1287.894	855.85
9-37711664-37711666-TG-CA	2	Olfr160	missense_variant	FN204-205FD	223.607	271.686
X-73259107-73259108-A-C	1	Xlr3c	missense_variant	H138Q	500.27	961.812
1-51755732-51755733-C-T	1	Myo1b	missense_variant	S1066N	684.321	918.883
19-13103021-13103022-G-A	1	Olfr1458	missense_variant	A94V	39.72	72.626
18-73931082-73931083-C-T	1	Mapk4	missense_variant	S356N	487.316	420.1
8-71472239-71472240-G-A	1	Dda1	missense_variant	E44K	822.413	668.482
2-5891296-5891297-C-T	1	Sec61a2	missense_variant	C45Y	325.837	158.125
9-106463368-106463369-G-A	1	Pcbp4	missense_variant	A388T	4106.841	902.926
17-38209033-38209034-C-T	4	Olfr135	missense_variant	P263L	36.96	31.48
3-127562336-127562337-A-G	1	Zgrf1	missense_variant	N404S	242.01	94.095
3-64086727-64086728-C-T	1	Vmn2r1	missense_variant	A165V	333.496	376.782
7-21350126-21350127-T-C	2	Vmn1r128	missense_variant	F252S	363.153	146.631
12-115847938-115847939-G-A	2	Ighv1-76	missense_variant	A98V	100.95	152.31
6-141943448-141943449-G-C	4	Slco1a1	missense_variant	Q46E	20.78	20.57
4-146466247-146466248-C-T	2	Zfp992	missense_variant	T142I	254.608	154.782
17-51742704-51742705-C-G	1	Satb1	missense_variant	V574L	714.644	323.043
2-87034020-87034021-C-G	2	Olfr1105	missense_variant	A67P	791.152	484.273
17-36168002-36168003-T-C	1	Gm8909	missense_variant	Q90R	298.717	477.815
16-32751941-32751942-A-T	1	Muc4	missense_variant	T607S	467.832	750.343
2-86958857-86958858-A-C	2	Olfr1099	missense_variant	F200C	153.2	317.147
7-5481284-5481285-T-A	1	Vmn2r28	missense_variant	T639S	40.087	39.264
11-76076501-76076502-C-T	1	Vps53	missense_variant	S600N	451.95	957.392
X-74303872-74303874-GG-TT	1	Atp6ap1	missense_variant	W420F	239.04	148.624
1-85099520-85099521-A-G	1	A530032D15Rik	missense_variant	V108A	1112.397	565.98
14-69800781-69800782-G-C	1	Rhobtb2	missense_variant	P10A	1163.161	869.881
4-47049323-47049324-G-A	1	Anks6	missense_variant	H194Y	3571.87	201.958
12-103895049-103895050-G-T	1	Serpina1c	missense_variant	L402I	598.537	625.01
9-38473292-38473293-T-A	1	Olfr905	missense_variant	L182H	491.4	173.63
X-155053804-155053805-A-T	2	Magea5	missense_variant	F197Y	2689.057	180.44
10-128886413-128886414-C-T	1	Gdf11	missense_variant	V191I	884.75	592.096
7-141638885-141638886-G-A	1	Muc6	missense_variant	P1958L	3296.78	880.15
2-3462551-3462552-C-A	3	Suv39h2	missense_variant	D376Y	7468.958	626.989
7-15555545-15555546-G-A	3	Obox1	missense_variant	G87S	1410.05	471.42
8-105270613-105270614-A-C	2	Hsf4	missense_variant	K64T	374.72	352.242
17-20114053-20114054-G-A	2	Fpr-rs7	missense_variant	T58I	1179.533	97.6
5-48219744-48219745-A-C	1	Slit2	missense_variant	D409A	2166.44	206.17

8-92996027-92996029-CT-TG	1	Slc6a2	missense_variant	P551L	2740.81	371.823
8-13319799-13319800-C-T	2	Tmco3	missense_variant	P604L	1670.75	444.898
2-111983151-111983152-A-T	1	Olfr1309	missense_variant	H315Q	1527.883	650.04
12-115765432-115765433-C-T	3	Ighv8-13	missense_variant	R68Q	460.45	269.14
10-79247983-79247984-C-T	2	Vmn2r81	missense_variant	P64L	32.156	61.981
11-59099940-59099941-T-C	2	Obscn	missense_variant	E1602G	885.299	677.782
1-174612523-174612524-T-G	1	Fmn2	missense_variant	L1169R	489.88	427.98
4-88835970-88835971-G-C	5	Ifna5	missense_variant	R149S	266.59	80.72
16-58824247-58824248-G-T	4	Olfr175	missense_variant	L154M	61.3	52.16
7-85624374-85624375-G-A	1	Vmn2r71	missense_variant	S799N	77.137	103.576
15-101460176-101460178-CC-TG	2	Krt81	missense_variant	G398A	845.8	835.11
10-129767874-129767875-A-C	1	Olfr808	missense_variant	K126N	96.154	38.331
7-105024326-105024327-G-A	1	Olfr675	missense_variant	L214F	10491.317	417.667
13-13393294-13393295-G-C	2	Gpr137b	missense_variant	L67V	37.919	38.91
2-55437488-55437489-T-G	2	Kcnj3	missense_variant	F97V	623.864	462.658
17-37299650-37299651-G-A	5	Olfr101	missense_variant	T257I	39.46	60.33
12-114896406-114896407-C-A	1	Ighv1-37	missense_variant	G61V	534.884	355.705
7-107097554-107097555-T-A	1	Olfr17	missense_variant	L30H	58.614	49.162
2-88412285-88412286-G-A	2	Olfr1180	missense_variant	P124L	59.09	148
X-73448840-73448841-C-T	1	Haus7	missense_variant	S138N	200.489	377.65
17-37299636-37299638-CA-TG	2	Olfr101	missense_variant	PA261-262PT	39.46	38.55
7-26611642-26611643-G-C	1	Vmn1r185	missense_variant	L146V	138.357	117.22
7-43497859-43497860-A-G	2	4931406B18Rik	missense_variant	V316A	57.84	37.71
2-122290606-122290607-G-A	1	Duox2	missense_variant	L775F	2642.956	755.249
7-104411593-104411594-C-T	3	Trim30a	missense_variant	S325N	42.92	192.8
4-28947572-28947573-A-G	2	Epha7	missense_variant	E615G	767.658	611.631
2-85976087-85976088-A-C	2	Olfr1029	missense_variant	S282R	158.68	145.52
2-91492595-91492596-G-A	1	Lrp4	missense_variant	D1142N	347.68	218.04
10-82284746-82284747-C-G	1	4932415D10Rik	missense_variant	R4143T	1923.95	539.834
7-45651030-45651031-C-T	1	Fut2	missense_variant	A106T	323.035	401.079
2-33565196-33565197-C-T	2	Lmx1b	missense_variant	M282I	263.26	422.756
17-35380445-35380446-C-A	1	H2-Q4	missense_variant	D168E	1112.95	509.96
7-7312964-7312965-C-T	1	Vmn2r30	missense_variant	S623N	316.293	927.385
2-86217338-86217339-C-T	1	Olfr1046	missense_variant	D124N	978.836	992.453
7-137459263-137459264-G-A	1	Glrx3	missense_variant	G209E	599.55	457.83
12-113769970-113769971-T-C	1	Ighv2-9-1	missense_variant	N77D	1351.699	714.299
1-188415854-188415855-C-T	1	Ush2a	missense_variant	T993I	2178.161	140.42
14-53105270-53105271-G-A	1	Trav6n-5	missense_variant	A89T	537.157	579.421
1-174191043-174191044-G-A	1	Spta1	missense_variant	E527K	453.169	358.171
7-5480797-5480798-C-G	2	Vmn2r28	missense_variant	S801T	378.02	411.84
8-43521737-43521738-T-G	1	Adam26b	missense_variant	I76L	319.956	816.752
1-161060750-161060751-G-A	1	Dars2	missense_variant	L200F	774.462	552.077
14-53538269-53538270-G-A	1	Trav12-1	missense_variant	R4H	727.232	802.528
7-5125817-5125818-C-T	2	Rasl2-9	missense_variant	E38K	2618.726	218.822
9-109722218-109722219-A-C	3	Fbxw26	missense_variant	F337V	113.097	90.11
11-115205094-115205095-C-T	3	Tmem104	missense_variant	T154I	86.38	75.381
9-119932693-119932694-C-T	1	Gorasp1	missense_variant	G91D	567.017	573.186
2-25906707-25906709-AA-TT	2	Kcnt1	missense_variant	K780L	1738.842	282.21
10-94579064-94579065-G-A	2	Tmcc3	missense_variant	G241R	197.738	101.63
7-8368345-8368346-G-C	1	Vmn2r44	missense_variant	T567R	711.017	571.321
13-8836797-8836798-C-T	1	Wdr37	missense_variant	V318I	2192.642	875.911
5-146170326-146170327-T-C	3	Gm6309	missense_variant	I70V	138.385	169.86
12-115242836-115242837-T-A	1	Ighv1-56	missense_variant	T106S	123.905	123.336
14-52349728-52349729-G-A	5	Olfr1513	missense_variant	H106Y	22.71	36.83
13-12294368-12294369-G-T	1	Actn2	missense_variant	L273I	2084.923	789.024

1-44156942-44156943-G-A	2	Ercc5	missense_variant	S32N	755.249	385.558
10-22371417-22371418-C-T	1	Raet1d	missense_variant	T131I	2767.515	168.69
4-140827908-140827910-CA-TG	4	Padi1	missense_variant	YV306-307YM	36.37	16.01
10-83233649-83233650-C-T	1	Slc41a2	missense_variant	G546D	141.87	359.68
1-93024632-93024633-G-A	2	Kif1a	missense_variant	A1308V	1133.079	536.118
5-103529659-103529660-T-C	1	Ptpn13	missense_variant	S715P	18.96	151.039
7-47589881-47589882-C-T	1	Mrgpra3	missense_variant	D99N	1061.696	181.063
4-131799653-131799654-A-G	2	Ptpru	missense_variant	L669P	855.376	579.421
2-76789486-76789487-C-T	1	Ttn	missense_variant	V14200I	515.537	106.233
7-49464889-49464890-T-C	1	Nav2	missense_variant	V874A	214.93	93.083
5-109047017-109047018-G-C	2	Vmn2r11	missense_variant	T814R	59.09	333.95
18-34845348-34845349-C-T	1	Reep2	missense_variant	P94S	2951.821	447.806
2-128676211-128676212-C-T	1	Anapc1	missense_variant	D241N	2054.82	806.85
2-110745913-110745914-T-G	1	Ano3	missense_variant	Q431H	620.998	970.711
8-14942625-14942626-C-T	1	Arhgef10	missense_variant	T288I	12366.876	741.464
X-74303850-74303851-G-A	1	Atp6ap1	missense_variant	G413S	1535.536	575.34
11-3153459-3153460-C-T	1	Sfi1	missense_variant	R438Q	182.339	359.948
17-36167948-36167949-T-C	1	Gm8909	missense_variant	H108R	666.931	548.908
17-37624371-37624373-CC-GT	1	Olfr116	missense_variant	AG87-88AR	388.269	929.159
2-131561347-131561348-A-G	2	Adra1d	missense_variant	V274A	313.394	169.469
8-79690418-79690419-T-G	1	Abce1	missense_variant	N321T	1319.98	549.69
11-104289964-104289965-C-T	1	Mapt	missense_variant	P82L	1686.903	727.931
7-80513276-80513277-G-A	1	Blm	missense_variant	P109S	1391.909	172.468
11-58552173-58552174-G-A	1	Olfr328	missense_variant	H22Y	841.395	669.885
17-23291036-23291037-C-T	1	Vmn2r114	missense_variant	G823E	411.235	591.684
12-114479302-114479303-C-T	2	Ighv10-1	missense_variant	V21M	133.77	116.169
3-130620788-130620789-C-T	3	Etnppl	missense_variant	S111F	291.246	441.835
18-23569662-23569663-G-C	1	Dtna	missense_variant	V84L	317.147	533.654
17-37590035-37590036-A-C	1	Olfr114	missense_variant	W106G	350.825	576.886
7-100878819-100878820-C-G	2	Arhgef17	missense_variant	G1738A	751.779	577.112
11-71114434-71114435-G-T	5	Nlrp1a	missense_variant	H651Q	92.081	35.76
7-45881541-45881542-T-A	1	Kdelr1	missense_variant	L132M	338.135	502.447
8-110866086-110866087-G-A	1	Cog4	missense_variant	V432I	478.035	385.15
1-164263402-164263403-C-G	1	Slc19a2	missense_variant	A461G	424.268	433.79
11-103455479-103455480-T-C	1	Lrrc37a	missense_variant	I3187V	1455.761	535.908
18-46850859-46850860-T-A	1	Lvrn	missense_variant	H223Q	659.754	388.269
11-99350948-99350949-G-A	1	Krt27	missense_variant	A42V	275.48	545.871
15-66541676-66541677-C-G	3	Tmem71	missense_variant	G182A	1167.508	626.744
4-146893819-146893820-C-T	1	Gm21411	missense_variant	C29Y	8297.38	646.338
18-36940296-36940297-C-T	2	Pcdha2	missense_variant	T327I	2314.528	883.597
17-37590083-37590084-C-T	3	Olfr114	missense_variant	G90S	597.06	176.64
4-116337488-116337489-G-A	1	Mast2	missense_variant	P202S	1393.991	849.356
10-34403686-34403687-C-T	1	Nt5dc1	missense_variant	D120N	1032.975	566.357
9-3001256-3001257-T-A	1	Gm10722	missense_variant	F111Y	160.262	228.997
6-121784364-121784366-AG-CA	1	Gm7298	missense_variant	QV1293-1294HM	4731.513	198.153
10-127064639-127064640-G-A	1	Cdk4	missense_variant	V92M	590.323	419.846
2-58137903-58137904-C-T	1	Cytip	missense_variant	V190M	2673.56	790.842
5-146526216-146526217-G-A	3	Gm3404	missense_variant	V70M	427.54	154
12-115847897-115847899-CA-TC	1	Ighv1-76	missense_variant	AV111-112AI	2270.335	405.442
3-40721631-40721632-T-C	1	Slc25a31	missense_variant	S203P	29.75	503.85
1-116184441-116184442-T-G	1	Cntnap5a	missense_variant	S487R	1101.108	858.677
11-9577190-9577191-T-G	2	Abca13	missense_variant	F4535V	460.352	300.67
6-41188956-41188957-A-T	1	Trbv20	missense_variant	L105F	999.171	802.86
12-118931855-118931856-C-T	3	Abcb5	missense_variant	A402T	1223.601	617.55
2-79333634-79333635-G-A	1	Cerkl	missense_variant	T447I	32.584	21.429

2-66317951-66317952-T-G	1	Scn1a	missense_variant	K1094T	1574.925	414.085
9-50913501-50913502-G-C	1	Sik2	missense_variant	A328G	108.306	94.095
11-69649177-69649178-G-A	1	Fxr2	missense_variant	S287N	315.108	659.754
4-3172365-3172366-G-A	2	Vmn1r2	missense_variant	G95E	454.423	483.571
17-34958181-34958182-A-C	1	Hspa1b	missense_variant	S276A	822.413	800
12-115861900-115861901-G-T	1	Ighv1-77	missense_variant	T106K	123.905	98.648
17-20776350-20776351-C-T	1	Vmn1r228	missense_variant	A302T	526.126	498.988
12-113716789-113716791-GT-TA	3	Ighv2-6	missense_variant	T77Y	976.967	104.35
7-28814083-28814084-T-G	1	Hnrnpl	missense_variant	I203R	367.18	169.83
9-109274569-109274570-G-A	1	Fbxw14	missense_variant	H348Y	8950.221	665.978
4-147678312-147678313-C-A	2	Zfp534	missense_variant	D21Y	381.795	281.728
6-39522875-39522876-G-A	2	Dennd2a	missense_variant	P252S	3484.095	528.555
17-29627179-29627180-G-C	2	Rnf8	missense_variant	E305D	323.661	334.264
2-98662377-98662379-CC-TC	1	Gm10801	missense_variant	P48S	1109.003	344.702
1-167226427-167226428-G-A	2	Uck2	missense_variant	P247L	1661.881	352.51
7-85150570-85150571-A-C	1	Vmn2r67	missense_variant	H486Q	794.017	553.134
2-88487514-88487515-C-T	1	Olfr1184	missense_variant	T261I	14726.176	871.485
5-136059811-136059812-C-T	1	Upk3bl	missense_variant	P55L	740.048	942.454
17-23387403-23387404-G-C	1	Vmn2r116	missense_variant	S430T	854.25	639.73
3-39009783-39009784-G-A	1	Fat4	missense_variant	A4630T	598.537	810.331
6-5168386-5168387-T-A	1	Pon1	missense_variant	K340I	6224.293	951.174
13-67683431-67683432-C-A	1	Zfp738	missense_variant	V3F	1746.184	483.159
19-11472464-11472465-T-C	1	Ms4a6c	missense_variant	Y74H	292.71	241.7
7-10158943-10158944-C-A	1	Vmn2r52	missense_variant	C756F	828.057	548.908
11-3221174-3221175-C-T	1	Eif4enif1	missense_variant	S222L	2416.898	812.288
9-20314065-20314066-C-T	3	Olfr18	missense_variant	A285T	65.133	72.576
12-115359183-115359184-T-A	2	Ighv1-61	missense_variant	S103C	123.905	119.149
6-57524931-57524932-T-G	1	Ppm1k	missense_variant	K82T	253.081	800
2-112092469-112092470-G-A	1	Olfr1314	missense_variant	T77I	1569.748	885.116
13-89607947-89607948-A-C	1	Hapln1	missense_variant	K290N	1358.595	487.316
3-9264685-9264686-T-G	1	Zbtb10	missense_variant	F368C	476.884	864.682
X-165252383-165252384-A-T	3	Glra2	missense_variant	Y256N	323.11	743.53
6-52729333-52729334-C-T	1	Tax1bp1	missense_variant	H107Y	104.847	174.345
15-78254866-78254867-G-C	2	Ncf4	missense_variant	E141D	266.846	351.633
14-101913324-101913325-C-A	1	Lmo7	missense_variant	F1150L	6267.437	767.658
12-114708823-114708824-T-A	1	Ighv1-19	missense_variant	S59C	534.884	783.899
17-25242587-25242588-C-T	1	Tsr3	missense_variant	T300I	9751.917	594.415
4-118869385-118869386-G-A	1	Olfr1331	missense_variant	V202I	4637.245	691.974
16-48565644-48565645-G-T	1	Morc1	missense_variant	D544Y	35288.64	838.031
12-37108954-37108955-C-T	3	Meox2	missense_variant	S42F	719.598	157.431
6-42912284-42912285-C-T	1	Olfr447	missense_variant	A254V	392.88	695.441
12-114538690-114538692-CA-GT	2	Ighv1-7	missense_variant	W52T	1007.14	337.51
7-103506429-103506430-G-A	1	Olfr610	missense_variant	P172L	8912.509	552.077
7-7479794-7479795-T-C	1	Vmn2r32	missense_variant	N60S	257.686	325.282
12-113736297-113736298-C-T	2	Ighv5-9-1	missense_variant	V65I	384.671	539.622
17-33999988-33999989-T-A	3	H2-K1	missense_variant	R27W	56.364	58.456
10-84591235-84591236-A-C	3	Tcp11l2	missense_variant	K188T	445.8	79.95
12-86058952-86058953-A-C	2	Tgfb3	missense_variant	F343V	556.019	80.555
10-22181157-22181158-C-T	1	Raet1e	missense_variant	P127L	7421.79	259.48
1-6810623-6810624-A-G	1	St18	missense_variant	D447G	2168.153	616.723
2-160957180-160957181-G-A	1	Chd6	missense_variant	P2328L	7208.657	922.678
X-74303922-74303923-C-G	1	Atp6ap1	missense_variant	L437V	616.723	618.144
9-106435842-106435843-C-T	1	Acy1	missense_variant	R126K	457.03	433.32
11-58757723-58757724-G-T	2	Olfr316	missense_variant	D20Y	155.955	57.412
2-87489670-87489671-A-C	1	Pramel7	missense_variant	I426R	242.153	103.774

16-32754346-32754347-C-T	2	Muc4	missense_variant	T1407I	500.035	328.852
14-39472102-39472103-G-A	2	Nrg3	missense_variant	S233F	84.09	274.3
7-8368096-8368097-G-A	4	Vmn2r44	missense_variant	T650I	434.34	303.969
7-49464780-49464781-T-A	1	Nav2	missense_variant	S838T	777.07	793.341
5-86906598-86906599-A-C	2	Ugt2b34	missense_variant	F108V	77.16	124.63
4-129083798-129083799-G-A	2	Rnf19b	missense_variant	S602N	353.256	665.411
2-164289936-164289937-G-A	1	Svs3a	missense_variant	V143M	2361.892	721.257
12-114896432-114896434-AA-GT	2	Ighv1-37	missense_variant	F52Y	8032.377	877.183
17-35471053-35471054-G-C	1	H2-Q10	missense_variant	W191S	271.686	265.387
2-62764576-62764577-C-T	1	Kcnh7	missense_variant	M716I	552.981	550.44
7-12362283-12362284-C-T	1	Vmn1r84	missense_variant	D161N	760.116	646.964
6-40571579-40571580-C-T	2	Olfr460	missense_variant	L65F	114.62	115.571
7-41288151-41288152-T-C	1	Gm5592	missense_variant	V286A	647.277	461.653
11-98250138-98250139-C-T	1	Cdk12	missense_variant	T1402I	1239.3	587.96
9-27010422-27010423-G-A	1	Vps26b	missense_variant	R269C	670.023	239.933
3-107213457-107213458-G-A	1	Cym	missense_variant	T277I	9185.229	618.284
3-62488922-62488923-C-T	1	Dhx36	missense_variant	A438T	1723.693	666.15
6-69775920-69775921-T-G	2	Igkv5-45	missense_variant	K59T	1294.971	229.61
1-154448915-154448916-G-A	1	Cacna1e	missense_variant	T1256I	492.617	299.473
17-36167930-36167932-GC-TT	1	Gm8909	missense_variant	A114K	340.993	289.908
13-63511819-63511820-A-C	1	Ptch1	missense_variant	S1340A	743.173	877.183
2-84940987-84940988-T-A	2	Slc43a3	missense_variant	Y108N	334.264	248.25
5-98737674-98737675-G-A	1	Cfap299	missense_variant	G147E	1761.286	821.921
7-98161940-98161941-G-A	3	Capn5	missense_variant	P47L	3616.263	375.868
1-66473470-66473471-A-C	1	Unc80	missense_variant	N180T	784.45	855.376
4-147510858-147510860-AC-TA	1	Zfp982	missense_variant	Y46L	4174.842	983.196
2-87252095-87252096-T-C	3	Olfr1115	missense_variant	I53T	597.159	112.76
3-82039854-82039855-G-A	1	Gucy1b1	missense_variant	T349I	2939.409	170.71
12-51365553-51365554-C-T	1	G2e3	missense_variant	S459F	1709.588	356.074
3-122549201-122549202-G-T	1	Fnbp1l	missense_variant	R410S	2153.228	984.215
17-20029552-20029553-T-C	2	Vmn2r104	missense_variant	S819G	149.31	204.4
15-30647189-30647190-A-G	1	Ctnnd2	missense_variant	Q344R	1208.064	881.63
1-166269457-166269458-G-A	1	Ildr2	missense_variant	G82E	254.608	617.74
15-82391001-82391002-T-G	1	Cyp2d11	missense_variant	K248T	543.363	783.592
9-39950300-39950301-G-T	2	Olfr975	missense_variant	L157I	469.85	432.3
9-39217761-39217762-C-T	4	Olfr944	missense_variant	T135I	222.89	397.274
15-58182880-58182881-C-A	1	Fbxo32	missense_variant	G311V	4635.43	827.47
6-71963681-71963682-T-G	1	Polr1a	missense_variant	I1000S	884.871	884.871
10-52101838-52101839-A-T	4	Ros1	missense_variant	L1458H	194.09	93.6
2-90052006-90052007-C-T	1	Olfr140	missense_variant	G106R	267.01	608.96
6-58955280-58955281-T-A	1	Fam13a	missense_variant	K369M	143.579	41.696
17-36168009-36168010-C-T	1	Gm8909	missense_variant	E88K	298.717	226.6
2-86922884-86922885-C-A	4	Olfr1098	missense_variant	A216S	480.72	331.18
7-48871006-48871007-A-G	3	E2f8	missense_variant	I522T	315.566	440.646
4-84276138-84276139-C-G	1	Bnc2	missense_variant	G916A	1009.857	652.654
7-143536025-143536026-C-T	2	Nap1l4	missense_variant	V63I	164.32	65.68
7-103328821-103328822-C-T	1	Olfr598	missense_variant	T112I	338.135	242.153
14-4558360-4558361-C-G	1	Gm3047	missense_variant	I194M	392.726	548.605
17-33999978-33999980-AC-GT	3	H2-K1	missense_variant	V30T	56.364	44.361
10-116353855-116353856-G-A	2	Ptprb	missense_variant	A1520T	207.434	73.572
7-25331086-25331087-C-G	1	Megf8	missense_variant	C488W	613.42	989.601
8-25596378-25596379-T-A	3	Letm2	missense_variant	L10F	276.45	313.23
17-57224868-57224869-C-T	1	C3	missense_variant	V254I	254.608	69.16
9-38378165-38378166-A-G	1	Olfr251	missense_variant	Q95R	363.864	286.788
2-87213695-87213696-G-A	3	Olfr1113	missense_variant	G268E	86.52	72.89

17-37590033-37590034-C-A	1	Olfr114	missense_variant	W106C	350.825	267.972
15-80918576-80918577-A-C	1	Tnrc6b	missense_variant	N1527T	964.029	559.86
1-85089561-85089562-C-T	1	A530032D15Rik	missense_variant	G188R	1004.824	828.114
3-55477848-55477849-A-C	1	Dclk1	missense_variant	K360N	340.993	397.274
X-138220466-138220467-C-G	2	Il1rapl2	missense_variant	R147G	125.49	120.45
X-94638018-94638019-C-G	1	Gspt2	missense_variant	A588G	361.626	782.096
3-73049203-73049204-G-A	1	Slitrk3	missense_variant	P745L	2781.922	223.607
10-129625329-129625330-T-C	2	Olfr798	missense_variant	I244V	1219.242	762.237
3-94343347-94343348-T-G	1	Them5	missense_variant	F73V	3454.017	942.862
7-86171680-86171681-T-C	1	Vmn2r75	missense_variant	K15R	1633.37	578.442
6-136400878-136400879-A-G	1	E330021D16Rik	missense_variant	S318P	953.119	864.682
7-85410257-85410258-T-C	1	Vmn2r69	missense_variant	E481G	1714.313	556.6
2-111420896-111420897-C-T	1	Olfr1286	missense_variant	C18Y	9080.087	392.11
16-32754904-32754905-C-T	1	Muc4	missense_variant	A1593V	437.342	883.597
X-136734410-136734411-T-G	1	Morf4l2	missense_variant	K23Q	3786.692	996.002
17-36168007-36168008-C-G	1	Gm8909	missense_variant	E88D	298.717	314.963
8-110415777-110415778-A-C	3	Hydin	missense_variant	E763A	216.686	120.734
7-132759533-132759534-G-A	1	Fam53b	missense_variant	P255L	8205.216	908.009
2-87833826-87833827-G-C	3	Olfr1148	missense_variant	V263L	113.49	109.47
3-79480804-79480805-G-A	1	Fnip2	missense_variant	P843L	525.658	384.09
7-101504601-101504602-G-C	1	Gm45837	missense_variant	G485A	295.978	123.669
1-85485033-85485034-T-A	3	AC147806.1	missense_variant	I37F	1229.28	764.293
7-5480932-5480933-C-A	1	Vmn2r28	missense_variant	C756F	828.057	548.908
13-89705412-89705413-C-G	1	Vcan	missense_variant	G476A	1174.84	743.75
4-147755956-147755957-C-T	1	Zfp984	missense_variant	G146R	254.608	194.267
16-58824442-58824443-T-C	1	Olfr175	missense_variant	N89D	166.376	205.712
17-37624365-37624366-C-T	3	Olfr116	missense_variant	G90S	388.269	145.912
14-53133063-53133064-G-A	1	Trav6n-6	missense_variant	A91T	537.157	579.421
3-5412115-5412116-T-G	2	Zfhx4	missense_variant	F3239V	150.77	260.178
13-4145267-4145268-C-G	1	Akr1c18	missense_variant	A64P	539.622	830.348
12-114896418-114896419-T-A	1	Ighv1-37	missense_variant	K57M	994.489	971.538
17-22572068-22572070-TT-AC	1	Vmn2r111	missense_variant	IN82-83MY	348.201	103.951
2-86978564-86978565-A-C	2	Olfr1100	missense_variant	I77S	96.18	59.09
1-84963776-84963777-G-A	2	AC167036.1	missense_variant	V37I	2234.91	354.13
6-136328830-136328831-C-T	1	Eif4a3l1	missense_variant	L97F	524.916	522.504
3-53516991-53516992-G-A	1	Frem2	missense_variant	S3008F	1607.56	792.984
8-21734548-21734549-A-G	1	Defa24	missense_variant	I5V	685.899	482.072
15-35709611-35709612-T-G	1	Vps13b	missense_variant	S1902A	1143.563	764.293
12-114937284-114937285-G-A	5	Ighv1-42	missense_variant	P60L	2326.4	54.25
12-113685542-113685543-C-T	1	Ighv2-5	missense_variant	V97I	701.601	412.344
17-43087957-43087958-C-T	1	Tnfrsf21	missense_variant	P652S	615.304	574.235
17-23640153-23640154-C-T	1	Mmp25	missense_variant	R173H	1376.93	688.934
5-110893009-110893010-T-C	1	Ttc28	missense_variant	L88S	2555.26	507.92
3-40910580-40910581-C-G	1	Abhd18	missense_variant	P86A	49.31	57.1
10-127658676-127658677-G-A	1	Stat6	missense_variant	M703I	442.1	86.714
14-50414145-50414146-A-C	1	Olfr738	missense_variant	M201L	1076.168	912.748
1-66414347-66414348-A-T	4	Map2	missense_variant	K799I	2339.707	302.608
11-87889593-87889594-C-T	1	Olfr462	missense_variant	V101I	24.333	20.027
7-102984444-102984445-G-A	1	Olfr578	missense_variant	L240F	1042.533	221.2
2-88728951-88728952-A-G	1	Olfr1197	missense_variant	Y216H	420.562	316.104
10-76420486-76420487-A-G	1	Pcnt	missense_variant	F640L	5239.14	674.186
7-26611615-26611616-G-T	2	Vmn1r185	missense_variant	P155T	1059.473	234.471
17-37151436-37151437-T-G	1	Olfr93	missense_variant	E178D	813.317	865.486
7-56328748-56328749-G-A	1	Oca2	missense_variant	R555H	1318.609	963.487
17-37952445-37952446-C-A	2	Olfr761	missense_variant	D193Y	5371.431	401.079

10-107494294-107494295-G-C	1	Myf6	missense_variant	A137G	263.081	719.406
4-147613774-147613775-G-A	1	Zfp979	missense_variant	T159I	62.964	153.14
17-23900506-23900509-TAA-CTC	3	Dcpp2	missense_variant	YN98-99YS	65.44	93.38
2-76892550-76892551-T-C	1	Ttn	missense_variant	K5680R	507.294	273.691
13-22179086-22179087-A-T	4	Vmn1r191	missense_variant	C166S	56.364	60.814
2-86217361-86217362-A-C	1	Olfr1046	missense_variant	F116C	656.281	728.216
4-88835981-88835982-A-T	2	Ifna5	missense_variant	Y153F	1528.481	434.6
2-129630101-129630102-T-G	1	Sirpa	missense_variant	V487G	884.525	890.656
5-14676235-14676236-C-T	1	Pclo	missense_variant	P1703S	659.754	98.876
1-107271843-107271844-T-C	1	Serpinb3c	missense_variant	S316G	359.948	544.829
2-86988394-86988395-A-C	2	Olfr1101	missense_variant	S260R	93.15	98.43
12-113611289-113611290-G-A	3	Ighv2-3	missense_variant	L82F	284.66	346.39
6-42838383-42838384-T-G	2	Olfr449	missense_variant	F168V	839.963	86.43
2-86181567-86181568-T-C	2	Olfr52	missense_variant	D181G	1145.591	531.377
2-86255419-86255420-T-G	1	Olfr1049	missense_variant	N91T	1791.678	873.152
8-108947744-108947745-G-A	2	Zfhx3	missense_variant	S1809N	371.823	204.3
10-22181175-22181176-C-T	1	Raet1e	missense_variant	T133I	2767.515	168.69
2-40697359-40697360-C-T	1	Lrp1b	missense_variant	S3961N	437.342	427.98
11-58551585-58551586-A-T	4	Olfr328	missense_variant	S218T	166.96	135.935
9-73075511-73075512-A-C	1	Rab27a	missense_variant	K33T	2239.185	682.48
3-98619430-98619431-T-C	2	Hsd3b5	missense_variant	H233R	243.824	375.56
12-59160181-59160182-G-A	3	Mia2	missense_variant	A317T	869.49	111.91
1-14887649-14887650-T-G	1	Trpa1	missense_variant	N695H	389.789	363.773
11-58529802-58529803-G-A	1	Olfr330	missense_variant	T61I	72.083	65.137
11-71109048-71109049-G-A	1	Nlrp1a	missense_variant	P762S	27.102	102.603
6-43116448-43116449-G-A	1	Olfr441	missense_variant	A236T	653.522	727.232
2-87357847-87357848-C-A	1	Olfr1120	missense_variant	P135T	38.331	34.03
12-114222980-114222981-C-T	2	Ighv7-4	missense_variant	R57H	696.429	446.909
1-173860338-173860339-C-A	1	Mndal	missense_variant	S443I	438.359	301.877
3-55255881-55255882-G-A	1	Dclk1	missense_variant	G132S	948.615	358.996
1-84964290-84964291-G-A	1	AC167036.1	missense_variant	R208Q	1249.169	846.993
6-97296288-97296289-G-A	1	Frmd4b	missense_variant	T714I	1866.767	717.943
12-115145530-115145531-T-A	2	Ighv1-52	missense_variant	S103C	123.905	119.149
14-14931283-14931284-G-C	1	Nek10	missense_variant	G752A	173.884	72.654
7-38099525-38099526-T-G	2	Ccne1	missense_variant	D259A	1365.99	160.36
2-87628700-87628701-G-T	2	Olfr1131	missense_variant	K79N	461.94	291.42
8-26110759-26110760-A-G	2	Hook3	missense_variant	V40A	835.776	451.95
2-87544979-87544980-G-A	1	Olfr1128	missense_variant	S188F	369.199	208.094
5-75163746-75163747-G-A	1	Pdgfra	missense_variant	V88I	3900.228	971.091
2-89193310-89193311-G-T	2	Olfr1226	missense_variant	S241Y	23699.603	537.157
3-5402307-5402308-G-T	1	Zfhx4	missense_variant	D2509Y	409.84	392.45
7-23984770-23984772-AT-CC	1	Vmn1r181	missense_variant	IF220-221IL	92.683	130.017
17-46134990-46134991-C-T	1	Rsph9	missense_variant	E218K	1737.86	711.98
10-36828795-36828796-G-A	1	Hs3st5	missense_variant	G32R	720.211	454.423
5-36065346-36065347-A-C	1	Sorcs2	missense_variant	F355V	337.323	722.487
17-7804856-7804857-C-T	1	Fndc1	missense_variant	E40K	759.397	784.45
17-37382615-37382616-A-C	1	Olfr105-ps	missense_variant	Q16H	759.819	400.599
10-40920378-40920379-C-T	1	Wasf1	missense_variant	A35V	382.023	431.609
13-59474612-59474613-C-G	1	Agtpbp1	missense_variant	S933T	323.035	263.008
12-58212757-58212758-G-A	2	Sstr1	missense_variant	G56S	1718.264	549.655
18-44168135-44168136-T-G	2	Spinkl	missense_variant	K45T	1377.63	258.54
10-105413515-105413516-C-G	1	Tmtc2	missense_variant	A119P	503.605	670.286
11-71181702-71181703-C-T	4	Nlrp1b	missense_variant	C438Y	222.377	113.78
14-50414141-50414142-T-G	1	Olfr738	missense_variant	I199M	549.87	857.551
16-59216159-59216160-A-C	2	Olfr199	missense_variant	L151R	170.612	105.704

9-3450127-3450128-C-T	1	Cwf19l2	missense_variant	L610F	2558.586	95.94
5-15473119-15473120-G-T	1	Gm21149	missense_variant	L160I	1476.59	506.128
10-27459393-27459394-A-C	1	Lama2	missense_variant	I95S	2052.391	581.112
14-119041595-119041596-T-A	1	Uggt2	missense_variant	K833I	23.05	53.62
12-101509249-101509250-A-G	1	Catsperb	missense_variant	E351G	792.984	767.658
6-122082717-122082718-G-A	2	Mug2	missense_variant	A1352T	251.868	238.281
6-132957684-132957685-T-C	1	Tas2r131	missense_variant	M54V	652.654	451.771
6-57404710-57404711-A-G	1	Vmn1r19	missense_variant	N83S	799.89	609.68
18-78109935-78109936-T-G	1	Slc14a1	missense_variant	I313L	1023.505	577.112
4-156338745-156338746-G-A	1	Vmn2r-ps159	missense_variant	V747I	3264.6	682.072
11-60762537-60762538-C-T	3	Top3a	missense_variant	V82I	113.308	107.711
7-7384459-7384460-A-T	3	Vmn2r31	missense_variant	I704K	185.038	139.399
2-130248713-130248714-G-A	1	Tmc2	missense_variant	S660N	265.62	698.651
17-19394458-19394459-T-G	1	Vmn2r99	missense_variant	S814A	149.31	208.574
15-60919764-60919765-G-A	1	A1bg	missense_variant	A274V	490.072	733.42
14-53073258-53073259-T-C	1	Trav13d-4	missense_variant	F106L	2469.769	922.678
14-53635152-53635153-G-T	1	Trav13-2	missense_variant	A29S	4614.132	790.842
11-71123708-71123710-GG-CA	1	Nlrp1a	missense_variant	T238M	1858.189	810.331
11-67205509-67205510-G-A	1	Myh1	missense_variant	E272K	291.44	330.01
7-65663890-65663891-G-A	1	Tarsl2	missense_variant	E353K	1059.65	585.18
4-88683345-88683346-T-A	2	Ifna2	missense_variant	K145I	266.59	790.47
9-19249033-19249034-G-A	1	Olfr843	missense_variant	R122C	36.58	64.54
18-36004746-36004747-C-T	1	Psd2	missense_variant	A464V	437.342	560.916
11-60779684-60779685-T-G	2	Smcr8	missense_variant	I553S	44.884	732.976
19-40723131-40723132-C-T	1	Entpd1	missense_variant	A132V	254.84	388.83
19-11472482-11472483-T-A	4	Ms4a6c	missense_variant	S80T	95.556	149.025
3-103812188-103812189-G-C	3	Ap4b1	missense_variant	G73A	115.45	51.81
4-147755804-147755805-T-A	3	Zfp984	missense_variant	K196N	80.869	52.459
1-173926891-173926892-C-T	1	Ifi203	missense_variant	E758K	93.56	76.048
2-148700412-148700413-G-A	1	Napb	missense_variant	A209V	484.273	959.599
7-11880408-11880409-C-T	1	Vmn1r75	missense_variant	L23F	201.878	202.81
14-50736547-50736548-C-A	1	Olfr749	missense_variant	V205F	870.623	23.442
4-156331348-156331350-AG-TA	3	Vmn2r-ps159	missense_variant	PV56-57PI	81.403	30.19
4-88602850-88602851-T-A	2	Ifna12	missense_variant	Y153F	1528.481	434.6
13-100161675-100161677-TG-GA	4	Naip2	missense_variant	S617F	1125.278	634.249
3-138286841-138286842-G-C	1	Adh1	missense_variant	E240Q	2905.762	849.689
11-57769625-57769626-A-G	1	Galnt10	missense_variant	I297V	1022.351	898.669
2-87071285-87071286-G-A	2	Olfr1107	missense_variant	P283S	280.711	35.817
11-46577224-46577225-C-T	1	BC053393	missense_variant	S42F	433.81	278.3
9-38402967-38402968-T-G	1	Olfr147	missense_variant	F31L	27.595	40.163
19-20640086-20640087-C-T	1	Aldh1a1	missense_variant	S461L	632.543	693.57
9-40782560-40782561-C-T	1	Clmp	missense_variant	T358I	14403.28	842.24
14-34343681-34343682-G-A	1	Glud1	missense_variant	V546I	398.24	141.15
12-113879112-113879113-C-T	1	Ighv2-9	missense_variant	M111I	2192.642	605.049
12-105222561-105222562-G-A	1	Tcl1	missense_variant	R31C	265.87	306.024
9-114629057-114629058-T-C	2	Cnot10	missense_variant	T127A	531.377	392.493
11-59328678-59328679-A-C	1	Wnt9a	missense_variant	K177T	1672.99	664.95
2-119070100-119070101-C-G	1	Knl1	missense_variant	S761C	570.506	836.103
2-86122769-86122770-C-T	1	Olfr1038-ps	missense_variant	P282L	1629.296	719.449
3-30659084-30659085-G-A	2	Lrriq4	missense_variant	R445Q	548.125	595.81
7-7476901-7476902-G-C	1	Vmn2r32	missense_variant	L91V	883.597	618.284
9-38047584-38047585-G-A	2	Olfr884	missense_variant	R121K	47.592	45.576
18-80972970-80972971-C-T	1	Sall3	missense_variant	E581K	1143.115	929.159
7-8472031-8472032-G-C	1	Vmn2r45	missense_variant	L666V	845.454	869.7
X-104565824-104565825-G-C	2	Zdhhc15	missense_variant	P205A	767.658	588.356

12-115208227-115208229-CC-TT	2	Ighv1-55	missense_variant	G73N	2522.784	837.065
16-58916367-58916368-C-T	1	Olfr180	missense_variant	R91K	205.712	339.829
2-132879719-132879720-G-T	1	Lrrn4	missense_variant	P59H	944.257	307.86
17-20030004-20030005-G-C	1	Vmn2r104	missense_variant	T668S	638.646	409.506
6-71625787-71625788-G-A	1	Kdm3a	missense_variant	P105S	1534.935	465.683
17-23360024-23360025-G-A	1	Vmn2r115	missense_variant	G824E	411.235	591.684
16-17574522-17574523-G-T	1	Slc7a4	missense_variant	A349D	315.689	411.396
7-102753071-102753072-G-A	2	Olfr560	missense_variant	P286S	801.611	296.56
5-8397260-8397261-T-C	1	Dbf4	missense_variant	T650A	687.211	374.188
9-123828645-123828646-C-G	2	Fyco1	missense_variant	A822P	1828.1	172.468
5-106892702-106892703-T-A	1	Hfm1	missense_variant	H732L	249.158	238.605
1-171530501-171530502-G-C	3	Itln1	missense_variant	S226C	136.855	560.093
17-35265980-35265981-G-A	1	H2-D1	missense_variant	V273M	591.916	468.015
16-44419262-44419263-G-A	2	Cfap44	missense_variant	V558I	1806.467	638.014
2-146109987-146109988-G-A	2	Cfap61	missense_variant	G797S	304.93	260.3
18-49723694-49723695-A-C	1	Dtwd2	missense_variant	I175S	676.488	98.46
7-7291660-7291661-G-C	1	Clcn4	missense_variant	T367S	1064.78	682.747
3-102898878-102898879-T-G	1	Sycp1	missense_variant	M499L	279.12	157.78
11-58566975-58566976-G-A	1	Olfr224	missense_variant	A123V	37.919	51.54
5-15030266-15030267-G-C	1	Gm17019	missense_variant	A177G	497.976	704.005
1-20520287-20520288-C-T	1	Pkhd1	missense_variant	G1745D	296.545	298.6
17-34715849-34715850-G-C	2	Tnxb	missense_variant	E2478Q	735.143	743.14
9-66462954-66462955-G-A	2	Herc1	missense_variant	G3008R	115.571	98.59
2-109894548-109894549-T-C	1	Lin7c	missense_variant	V41A	1039.295	810.331
1-85028735-85028736-T-C	2	AC167036.2	missense_variant	I37V	948.615	681.52
17-18597846-18597847-T-C	1	Vmn2r96	missense_variant	M562T	2063.33	490.298
7-104564378-104564379-C-T	1	Olfr652	missense_variant	L53F	211.393	60.407
2-86508576-86508578-GG-AA	1	Olfr1076	missense_variant	LV39-40LM	585.724	520.002
4-89691819-89691820-G-C	1	Dmrta1	missense_variant	C339S	574.46	391.976
6-146396964-146396965-G-A	1	Itpr2	missense_variant	L366F	138.302	89.501
X-74303855-74303856-C-A	1	Atp6ap1	missense_variant	F414L	1535.536	249.88
12-115193709-115193710-T-A	1	Ighv1-54	missense_variant	T106S	123.905	123.336
5-96769650-96769651-G-A	1	Fras1	missense_variant	R3510K	323.504	323.504
1-85484519-85484520-C-T	1	AC147806.1	missense_variant	R208Q	1249.169	846.993
10-58224914-58224915-A-C	1	AW822073	missense_variant	W6G	1285.553	942.454
12-110665717-110665718-T-C	1	Dync1h1	missense_variant	I4474T	539.18	887.05
9-3359583-3359584-C-T	1	Alkbh8	missense_variant	T291I	340.993	503.394
5-146491780-146491781-G-A	3	Gm6370	missense_variant	V70M	427.54	154
10-128408556-128408557-C-T	1	Nabp2	missense_variant	G90S	572.915	216.815
7-102659059-102659060-C-T	3	Olfr555	missense_variant	P80S	111.88	65.438
1-85610748-85610749-A-G	2	Sp140	missense_variant	E69G	748.618	632.791
8-40999693-40999694-C-T	1	Mtus1	missense_variant	M1019I	3206.934	845.454
19-39643354-39643355-T-A	3	Cyp2c67	missense_variant	N133Y	2880.448	57.94
7-63758015-63758016-C-T	1	Otud7a	missense_variant	T689I	3289.198	207.297
14-14087496-14087497-T-G	1	Atxn7	missense_variant	H225Q	346.011	309.353
1-84964326-84964327-G-A	1	AC167036.1	missense_variant	R220Q	380.417	552.408
17-34958106-34958107-G-A	2	Hspa1b	missense_variant	R301W	1325.135	776.48
12-113702557-113702558-C-G	1	Ighv5-12	missense_variant	L7F	2031.562	80.878
12-91825362-91825363-C-T	2	Sel1l	missense_variant	A299T	187.107	124.87
14-60027953-60027954-T-G	1	Atp8a2	missense_variant	K311T	1120.108	127.427
7-85624395-85624396-C-T	1	Vmn2r71	missense_variant	A806V	677.376	371.39
5-109047050-109047051-G-C	1	Vmn2r11	missense_variant	T803S	2787.79	743.464
16-87437318-87437319-C-T	1	Rwdd2b	missense_variant	S61N	155.51	280.02
X-60293649-60293650-C-T	1	Atp11c	missense_variant	G223R	466.675	984.564
2-88682892-88682893-G-T	1	Olfr1195	missense_variant	L280I	220.206	145.881

15-76537777-76537778-T-C	1	Fbxl6	missense_variant	Q187R	274.641	226.511
11-59122858-59122859-C-T	1	Obscn	missense_variant	R1054H	807.402	885.299
10-77548638-77548639-G-A	1	Itgb2	missense_variant	C198Y	207.297	288.29
7-31375891-31375893-GA-CT	1	Scgb1b3	missense_variant	E56L	3454.017	821.694
1-63592636-63592637-C-T	1	Adam23	missense_variant	T824I	2659.99	913.861
11-110301544-110301545-T-G	1	Abca5	missense_variant	I714L	388.29	433.112
16-32755090-32755091-T-C	2	Muc4	missense_variant	I1655T	478.729	485.389
3-14770865-14770866-C-T	1	Car1	missense_variant	S131N	16.915	22.712
9-19360965-19360966-G-C	1	Olfr846	missense_variant	L130V	18.96	32.589
17-36032407-36032408-G-A	3	H2-T23	missense_variant	R26W	30.549	30.761
11-120956420-120956421-G-A	1	Slc16a3	missense_variant	R145H	82.08	35.11
16-58916374-58916376-CT-TC	1	Olfr180	missense_variant	VD88-89VN	205.712	166.376
6-4516913-4516914-G-A	1	Col1a2	missense_variant	M191I	1145.591	316.12
7-39408114-39408115-G-C	1	Gm5114	missense_variant	H693Q	228.498	548.908
12-113306429-113306430-A-G	1	Ighg2b	missense_variant	Y324H	337.357	459.293
17-35473270-35473271-T-G	1	H2-Q10	missense_variant	S270A	591.916	395.603
5-129697820-129697821-G-A	1	Septin14	missense_variant	L97F	256.012	222.89
4-56946850-56946851-G-C	1	Tmem245	missense_variant	S187R	449.656	694.56
8-43569341-43569342-T-G	2	Adam26a	missense_variant	E370D	38.394	43.46
3-130631732-130631733-G-T	2	Etnppl	missense_variant	G396W	437.342	537.157
17-35383075-35383076-A-T	2	H2-Q4	missense_variant	N305Y	1686.38	870.382
1-32414963-32414964-G-C	1	Khdrbs2	missense_variant	A144P	18.124	79.298
4-148472044-148472045-G-A	3	Mtor	missense_variant	V971M	85.364	45.576
19-4762288-4762289-A-G	4	Rbm4b	missense_variant	N242S	212.94	66.07
9-20286211-20286212-G-A	2	Olfr39	missense_variant	G179D	390.023	392.726
18-68408021-68408022-C-G	2	Mc2r	missense_variant	A67P	337.91	231.3
1-90214837-90214838-C-T	1	Ackr3	missense_variant	L340F	1139.58	930.12
7-14491915-14491917-CC-GT	3	Sult2a7	missense_variant	W33Y	276.91	153.116
8-110519286-110519287-A-G	1	Hydin	missense_variant	T2211A	1183.386	953.119
4-88683321-88683322-T-A	2	Ifna2	missense_variant	Y153F	1528.481	434.6
6-29204633-29204634-T-C	1	Impdh1	missense_variant	Y282C	34.442	65.779
6-40900068-40900069-G-A	1	Prss58	missense_variant	L9F	329.678	343.2
17-23345958-23345959-T-G	2	Vmn2r115	missense_variant	I273M	268.762	261.144
13-65296554-65296555-C-G	1	Zfp369	missense_variant	T504S	315.99	353.256
6-70518726-70518727-A-C	2	Igkv3-12	missense_variant	E79D	641.342	656.281
2-87846960-87846961-C-T	2	Olfr1150-ps1	missense_variant	T230I	37.919	78.933
4-49380300-49380301-G-A	2	Acnat2	missense_variant	P359L	298.6	41.313
2-66508624-66508625-T-A	1	Scn9a	missense_variant	K1172N	1202.984	622.429
5-111225997-111225998-C-G	1	Ttc28	missense_variant	A1069G	369.905	698.651
16-25871091-25871092-G-C	1	Trp63	missense_variant	R436S	970.711	420.01
1-126025950-126025951-C-T	1	Nckap5	missense_variant	A1023T	3937.4	876.96
7-7241578-7241579-C-T	2	Vmn2r29	missense_variant	C432Y	519.111	24.271
7-86164094-86164095-C-T	1	Vmn2r75	missense_variant	E500K	5834.44	607.1
6-132803729-132803730-T-C	1	Tas2r117	missense_variant	V277A	899.684	401.874
15-44526819-44526820-C-T	2	Pkhd1l1	missense_variant	P1444L	644.303	71.3
10-116314083-116314084-G-C	3	Ptprb	missense_variant	G200A	149.93	117.49
5-139393032-139393033-G-A	1	Gpr146	missense_variant	V197M	296.82	223.07
4-32707627-32707628-G-C	3	Mdn1	missense_variant	G1639A	306.024	243.824
7-23984786-23984787-G-A	1	Vmn1r181	missense_variant	D226N	771.063	575.559
7-105368574-105368575-C-T	1	Olfr692	missense_variant	T83I	805.65	828.438
17-35380616-35380617-G-T	2	H2-Q4	missense_variant	K225N	143.492	111.099
7-20811666-20811668-AC-TG	1	Vmn1r114	missense_variant	KF173-174NI	10497.6	278.029
1-152843263-152843264-C-T	1	Smg7	missense_variant	G912D	325.282	511.988
17-35266194-35266195-C-T	1	H2-D1	missense_variant	P302S	9910.373	957.392
7-5125843-5125844-C-T	1	Rasl2-9	missense_variant	R29H	937.756	638.396

15-78297059-78297060-G-C	1	Csf2rb2	missense_variant	T44S	501.291	358.59
10-22371586-22371587-T-A	1	Raet1d	missense_variant	S187R	1227.694	638.396
17-23345177-23345178-G-C	1	Vmn2r115	missense_variant	W108S	1710.37	899.684
6-128559068-128559069-T-C	1	A2ml1	missense_variant	H720R	794.804	520.002
1-85259729-85259731-TC-CT	1	C130026I21Rik	missense_variant	D111S	565.98	197.39
10-8207552-8207553-C-T	2	Ust	missense_variant	R353K	411.15	396.278
1-85610789-85610790-G-T	1	Sp140	missense_variant	D83Y	5435.757	124.24
17-13883818-13883819-G-C	1	Afdn	missense_variant	G1139A	809.264	952.994
18-6992834-6992835-C-T	2	Mkx	missense_variant	V150I	686.32	384.83
7-10158938-10158939-C-A	1	Vmn2r52	missense_variant	A758S	1048.238	665.978
7-12321454-12321455-A-C	1	Vmn1r83	missense_variant	L225R	119.47	873.494
10-121615051-121615052-C-G	1	Xpot	missense_variant	A148P	58.088	88
10-36993649-36993650-C-T	4	Hdac2	missense_variant	P228S	44.12	39.48
2-86639294-86639296-GA-AT	2	Olfr1084	missense_variant	SH137-138SY	429.625	415.997
11-73453653-73453654-T-A	1	Olfr380	missense_variant	K186M	4179.17	614.13
4-115532666-115532667-G-T	1	Cyp4a10	missense_variant	G500W	703.218	654.772
X-74303875-74303876-T-C	1	Atp6ap1	missense_variant	M421T	1035.33	928.966
10-130210347-130210348-T-G	1	Olfr827	missense_variant	S261R	305.14	970.711
17-34157316-34157317-G-T	1	H2-DMb1	missense_variant	V137F	6439.172	457.963
18-37005864-37005865-A-C	1	Pcdha11	missense_variant	E182D	1354.815	770.69
2-85991848-85991849-T-G	1	Olfr1031	missense_variant	F11V	378.521	301.481
18-60550219-60550220-A-C	1	Dctn4	missense_variant	N290T	299.473	373.104
12-114538612-114538613-T-C	1	Ighv1-7	missense_variant	K78R	326.498	537.157
7-40993461-40993462-C-G	1	4930433I11Rik	missense_variant	T185S	659.754	659.754
16-58872883-58872885-CT-TC	1	Olfr177	missense_variant	VD88-89VN	205.712	166.376
7-12041929-12041930-A-T	1	Vmn1r77	missense_variant	K211I	1806.675	849.8
11-103631333-103631334-G-C	1	Lyzl6	missense_variant	C145W	115.571	79.999
7-85620587-85620588-A-T	1	Vmn2r71	missense_variant	I436F	2772.235	920.089
11-36047471-36047472-A-T	2	Tenm2	missense_variant	L1458H	246.752	105.502
17-17893094-17893095-C-G	1	Fpr2	missense_variant	L118V	462.658	471.752
6-57137805-57137806-A-C	2	Vmn1r11	missense_variant	S152R	319.22	272.327
17-35473276-35473277-G-A	1	H2-Q10	missense_variant	V272M	591.916	450.049
12-113685590-113685591-C-G	2	Ighv2-5	missense_variant	A81P	933.448	708.093
8-93076192-93076193-C-T	2	Ces1b	missense_variant	D90N	196.38	154.782
17-37299642-37299644-GG-TA	2	Olfr101	missense_variant	IR259-260IS	39.46	36.67
15-55212194-55212195-G-A	1	Deptor	missense_variant	S265N	460.66	473.54
7-5125778-5125780-CT-TG	1	Rasl2-9	missense_variant	LV50-51FI	445.656	362.243
4-147755719-147755720-G-A	1	Zfp984	missense_variant	L225F	4786.301	179.473
7-35547979-35547980-G-A	1	Nudt19	missense_variant	L335F	819.146	688.934
11-77472850-77472851-C-T	1	Ankrd13b	missense_variant	A429T	1763.031	510.49
12-115193725-115193726-C-A	2	Ighv1-54	missense_variant	M100I	123.905	102.351
4-88827848-88827849-C-A	3	Ifna6	missense_variant	T145K	577.28	266.59
11-65989859-65989860-T-G	1	Dnah9	missense_variant	N2637T	1132.635	849.356
17-33999943-33999944-G-A	2	H2-K1	missense_variant	R42W	6044.62	466.675
1-84964237-84964238-T-G	1	AC167036.1	missense_variant	F190L	272.327	271.075
9-109326626-109326627-C-T	2	Fbxw28	missense_variant	C359Y	194.267	158.168
17-20540591-20540592-C-A	2	Vmn2r109	missense_variant	L834F	359.749	256.448
12-113625613-113625614-C-G	1	Ighv5-6	missense_variant	S82T	778.45	454.62
10-89506605-89506606-T-C	1	Nr1h4	missense_variant	Q4R	579.549	487.85
12-115604157-115604158-C-T	1	Ighv1-67	missense_variant	G45S	567.884	162.963
16-32752014-32752015-C-A	1	Muc4	missense_variant	P631Q	585.724	767.658
8-63927832-63927833-A-C	1	Sgo2b	missense_variant	V655G	228.171	416.16
12-55676671-55676672-T-C	2	Ralgapa1	missense_variant	T1690A	472.835	297.43
8-15998852-15998853-T-A	1	Csmd1	missense_variant	K2283M	157.06	241.68
7-12670590-12670591-T-G	1	Vmn2r55	missense_variant	N295T	883.597	433.221

17-35266221-35266222-G-A	4	H2-D1	missense_variant	V311I	82.431	81.487
4-147390493-147390494-C-T	1	Zfp978	missense_variant	T166I	2169.046	154.782
7-9986358-9986359-C-G	2	Vmn2r49	missense_variant	V402L	298.47	249.98
1-12872308-12872309-A-C	1	Slco5a1	missense_variant	I704M	450.049	282.657
17-23477454-23477456-TG-CA	1	Vmn2r117	missense_variant	H326C	760.782	651
15-101528756-101528757-T-C	1	Krt84	missense_variant	T324A	186.78	120.022
9-38816146-38816147-G-T	2	Olfr922	missense_variant	V215F	470.793	362.936
4-68792849-68792850-G-A	1	Brinp1	missense_variant	P374S	3244.068	984.215
X-93593841-93593842-C-T	1	Pola1	missense_variant	C501Y	5114.816	431.35
4-88571581-88571582-T-C	1	Ifna14	missense_variant	K73E	1321.569	792.666
4-147390321-147390322-A-G	1	Zfp978	missense_variant	I109V	347.608	284.616
16-58823835-58823836-C-T	1	Olfr175	missense_variant	S291N	95.29	31.74
13-62172900-62172901-G-A	1	Zfp808	missense_variant	R648Q	598.537	243.824
2-165304933-165304934-G-A	3	Elmo2	missense_variant	A241V	356.074	456.685
5-18267182-18267183-C-T	4	Gnai1	missense_variant	A301T	74.97	46.73
2-129609071-129609072-G-A	1	Sirpa	missense_variant	G84E	338.8	581.92
5-94383664-94383665-T-G	1	AA792892	missense_variant	L136W	1143.563	740.048
12-65055164-65055165-C-T	2	Prpf39	missense_variant	P339S	896.314	353.39
9-19477584-19477585-C-T	5	Olfr850	missense_variant	V222I	13.034	5.085
12-114682674-114682675-C-T	3	Ighv1-18	missense_variant	R103H	521.86	171.281
17-18597855-18597856-G-A	1	Vmn2r96	missense_variant	G565E	575.559	701.875
6-132980313-132980314-T-G	1	Tas2r109	missense_variant	K218Q	2225.89	666.945
11-109517419-109517420-G-A	1	Arsg	missense_variant	A133T	2192.642	792.984
15-4912795-4912796-C-T	2	Mroh2b	missense_variant	H347Y	6591.36	631.809
2-87358133-87358134-C-T	2	Olfr1120	missense_variant	T230I	37.919	78.933
X-23958723-23958724-T-C	1	Gm4985	missense_variant	K71E	113.097	74.111
11-119436165-119436166-T-G	1	Rnf213	missense_variant	F1661V	174.345	283.702
1-174391788-174391789-C-G	1	Olfr248	missense_variant	A240G	408.69	428.805
7-103538837-103538838-A-C	1	Olfr612	missense_variant	L132R	433.221	500.853
14-50736550-50736551-A-G	4	Olfr749	missense_variant	F204L	59.602	65.654
16-96593076-96593077-T-G	2	Dscam	missense_variant	K2008N	51.642	41.384
7-10274335-10274336-A-T	2	Vmn1r66	missense_variant	L257I	291.66	115.902
12-114416642-114416643-T-C	2	Ighv6-5	missense_variant	M85V	387.338	544.616
5-71623936-71623937-G-A	1	Gabra4	missense_variant	A351V	1133.262	277.629
2-76753052-76753053-C-T	1	Ttn	missense_variant	E20753K	3033.45	950.342
7-18890252-18890253-G-A	1	Pglyrp1	missense_variant	V154I	1534.27	515.24
11-73187746-73187747-G-A	1	Ctns	missense_variant	P184L	864.682	289.908
6-32163415-32163416-C-T	2	Plxna4	missense_variant	R1679Q	1371.166	597.159
4-146466246-146466247-A-T	2	Zfp992	missense_variant	T142S	254.608	236.037
15-41096161-41096162-G-A	2	Zfpm2	missense_variant	M212I	396.36	104.494
13-116303260-116303261-C-T	1	Isl1	missense_variant	V184M	388.269	263.008
2-167104677-167104678-C-T	1	Kcnb1	missense_variant	G750E	189.71	262.476
17-35471031-35471032-C-A	1	H2-Q10	missense_variant	L184M	271.686	439.24
7-105600065-105600066-T-G	1	Hpx	missense_variant	N9H	573.186	471.752
17-45568404-45568405-G-A	2	Hsp90ab1	missense_variant	P630L	1845.398	796.324
7-10088027-10088028-C-T	1	Vmn2r51	missense_variant	V577I	875.165	865.147
12-40073324-40073325-C-T	1	Scin	missense_variant	A520T	430.616	674.668
11-58529814-58529815-G-A	1	Olfr330	missense_variant	T57I	13004.39	792.666
5-71869471-71869472-A-C	3	Gabrb1	missense_variant	N105T	230.192	120.251
17-37299756-37299757-T-A	6	Olfr101	missense_variant	N222Y	38.823	29.18
11-71114444-71114445-T-G	2	Nlrp1a	missense_variant	Y648S	64.282	91.22
6-57926211-57926212-T-A	1	Vmn1r23	missense_variant	T194S	1521.458	975.574
12-115242907-115242908-T-A	2	Ighv1-56	missense_variant	K82M	14330.456	643.073
11-45983392-45983393-G-A	1	Sox30	missense_variant	A377T	2938.258	734.666
7-29103579-29103580-A-G	1	Ryr1	missense_variant	L676P	611.069	719.406

13-68689306-68689307-A-T	2	Adcy2	missense_variant	L641H	270.29	108.893
7-101836738-101836739-C-T	1	Inppl1	missense_variant	C5Y	10414.043	677.189
6-42838692-42838693-G-A	1	Olfr449	missense_variant	V271I	274.05	399.788
17-36167979-36167980-C-T	1	Gm8909	missense_variant	G98S	1528.481	361.626
1-105741328-105741329-G-A	2	Relch	missense_variant	G1028D	287.799	468.911
17-20113527-20113528-C-A	1	Fpr-rs7	missense_variant	L233F	6564.03	791.35
5-71641090-71641091-T-C	1	Gabra4	missense_variant	R156G	558.586	231.787
2-158851763-158851764-C-T	4	Dhx35	missense_variant	T646I	9.289	50.033
12-114708691-114708692-T-G	3	Ighv1-19	missense_variant	N103H	954.06	171.281
10-26990809-26990810-C-T	1	Lama2	missense_variant	G2896D	854.358	666.931
11-96107512-96107513-G-A	3	Calcoco2	missense_variant	A28V	211.831	326.656
14-4558365-4558366-C-T	1	Gm3047	missense_variant	A196V	392.726	249.511
9-39819802-39819803-C-T	1	Olfr970	missense_variant	L55F	94.805	75.48
15-57259135-57259136-T-A	5	Slc22a22	missense_variant	Q148H	8.89	21.941
1-85610757-85610759-AG-GC	3	Sp140	missense_variant	K72S	748.618	101.92
2-103566901-103566902-G-C	1	Abtb2	missense_variant	G59A	274.322	196.38
5-124130480-124130481-A-T	1	Pitpnm2	missense_variant	F548Y	2150.63	196.377
8-70891658-70891659-A-C	1	Slc5a5	missense_variant	V139G	406.874	401.292
9-112136557-112136558-C-T	1	Arpp21	missense_variant	G415E	1015.57	990.558
2-145604547-145604548-G-A	3	Slc24a3	missense_variant	R292H	468.911	313.394
16-31127336-31127337-A-G	1	Acap2	missense_variant	F281L	3704.333	519.9
3-151517481-151517482-A-G	2	Adgrl4	missense_variant	Y565C	1046.43	782.907
6-123315808-123315809-T-C	1	Vmn2r19	missense_variant	L270S	828.057	751.224
12-75966729-75966730-T-G	1	Syne2	missense_variant	F2898L	897.181	897.181
2-111983699-111983700-C-G	1	Olfr1309	missense_variant	A133P	96.154	94.095
X-152612667-152612668-T-G	1	Shroom2	missense_variant	N1442H	763.133	645.41
1-33777641-33777642-G-A	1	Zfp451	missense_variant	P409L	5309.945	750.343
14-46384000-46384001-T-G	1	Bmp4	missense_variant	N362T	225.99	191.465
2-49947546-49947547-C-T	1	Lypd6b	missense_variant	P177L	1274.266	523.914
14-53363953-53363954-G-A	2	Trav13n-4	missense_variant	G60E	123.42	133.85
14-50298648-50298649-A-C	1	Olfr733	missense_variant	V220G	158.168	140.42
7-5480927-5480928-C-A	1	Vmn2r28	missense_variant	A758S	1048.238	665.978
12-72157485-72157486-T-G	1	Ccdc175	missense_variant	K260N	611.631	728
11-3140172-3140173-C-T	4	Sfi1	missense_variant	A716T	626.14	325.07
7-3717393-3717394-C-A	4	Pirb	missense_variant	V327L	51.179	79.816
3-90056567-90056568-C-G	1	4933434E20Rik	missense_variant	A109G	117.56	281.358
18-22920876-22920877-G-C	1	Nol4	missense_variant	P193A	828.057	497.976
6-131678761-131678762-G-A	1	Tas2r106	missense_variant	T42I	688.934	645.631
17-58967344-58967345-A-T	2	Pdzph1	missense_variant	S835T	257.093	375.051
6-80021913-80021914-A-G	1	Lrrtm4	missense_variant	E103G	358.996	364.83
13-21132993-21132994-T-A	3	Olfr263	missense_variant	F73Y	1562.104	204.5
17-69287624-69287625-C-A	1	Epb41l3	missense_variant	A898E	264.296	575.559
17-45568408-45568409-T-A	2	Hsp90ab1	missense_variant	N629Y	1003.51	719.406
12-113306433-113306434-T-G	1	Ighg2b	missense_variant	K322N	643.917	598.177
3-108402733-108402734-T-C	2	Celsr2	missense_variant	D1560G	356.93	331.329
11-70316308-70316309-C-G	1	Alox12e	missense_variant	G557A	1073.27	405.442
2-89816453-89816454-A-G	4	Olfr1255	missense_variant	I43V	295.57	264.53
18-84013927-84013928-T-C	2	Tshz1	missense_variant	N785S	125.05	111.496
1-186632484-186632485-T-C	1	Tgfb2	missense_variant	S271G	711.017	711.017
10-129387749-129387750-G-T	1	Olfr784	missense_variant	G39V	682.747	522.709
15-47838487-47838488-A-G	1	Csmd3	missense_variant	L1733P	140.78	172.43
9-39217775-39217776-A-G	5	Olfr944	missense_variant	I140V	53.03	41.347
6-89747328-89747329-C-T	3	Vmn1r41	missense_variant	T284I	79.138	176.97
4-115532645-115532646-C-T	1	Cyp4a10	missense_variant	L493F	471.752	51.895
7-3897221-3897222-G-A	1	Gm14548	missense_variant	S127L	1079.17	812.999

10-130034453-130034454-G-A	1	Olfr821	missense_variant	S276N	375.868	874.088
6-57956170-57956171-T-G	2	Vmn1r24	missense_variant	K121Q	613.762	131.594
7-3717411-3717412-A-T	1	Pirb	missense_variant	Y321N	548.858	690.971
7-141639453-141639454-C-T	2	Muc6	missense_variant	A1769T	650.264	616.723
2-88486836-88486837-C-T	1	Olfr1184	missense_variant	A35V	1987.97	952.24
1-59199148-59199149-G-T	4	Als2	missense_variant	L675I	137.49	111.89
7-141639591-141639592-C-T	1	Muc6	missense_variant	E1723K	540.866	751.224
9-39461642-39461643-T-G	6	Olfr954	missense_variant	F71V	40.559	37.09
14-50515313-50515314-A-G	1	Olfr742	missense_variant	I37V	200.676	235.955
6-120952378-120952379-G-C	1	Mical3	missense_variant	A1842G	814.873	867.142
3-83033096-83033097-G-A	1	Fga	missense_variant	G686E	389.85	701.66
11-58529905-58529906-G-A	2	Olfr330	missense_variant	H27Y	2496.636	156.466
7-7394422-7394424-TG-CT	2	Vmn2r31	missense_variant	IN278-279ID	119.43	200.028
8-15028516-15028517-G-C	1	Kbtbd11	missense_variant	G372A	436.606	154.618
2-178403761-178403762-C-A	1	Sycp2	missense_variant	D22Y	5167.256	121.995
11-58684276-58684277-C-T	1	Olfr320	missense_variant	L135F	190.108	86.099
18-35213088-35213089-A-G	1	Lrrtm2	missense_variant	S387P	605.466	711.85
9-45450582-45450583-G-A	1	Dscaml1	missense_variant	V214I	1172.438	286.477
12-103691891-103691892-C-G	1	Serpina1f	missense_variant	E251D	743.464	632.791
17-33997115-33997116-C-A	1	H2-K1	missense_variant	V310F	792.984	792.666
2-113525520-113525521-A-C	1	Fmn1	missense_variant	K867T	2958.626	901.758
2-180713220-180713221-C-T	1	Gid8	missense_variant	P7S	134.86	67.01
6-70961016-70961017-G-A	4	Foxi3	missense_variant	S385N	574.93	120
17-38208357-38208358-G-A	2	Olfr135	missense_variant	A38T	218.82	260.178
17-30656922-30656923-C-G	1	Dnah8	missense_variant	A508G	666.945	403.887
7-42011872-42011873-C-A	2	Vmn2r59	missense_variant	R839S	437.613	131.853
13-23217383-23217384-G-T	2	Vmn1r221	missense_variant	K4N	306.024	543.001
1-58752442-58752443-G-C	1	Cflar	missense_variant	A302P	1104.079	89.743
12-113597644-113597645-C-T	1	Ighv5-4	missense_variant	A52T	384.671	484.273
9-5321499-5321500-C-T	1	Casp4	missense_variant	H84Y	9338.1	598.537
9-89090620-89090621-T-A	1	Trim43b	missense_variant	K160I	241.32	85.483
4-123917596-123917597-T-G	1	Rragc	missense_variant	F22V	254.736	170.644
6-122713538-122713539-C-T	1	Nanog	missense_variant	P301L	1682.67	775.3
7-103565110-103565111-C-A	1	Olfr616	missense_variant	S56I	138.909	148.226
17-35471049-35471050-G-C	1	H2-Q10	missense_variant	E190Q	271.686	145.053
12-113597884-113597885-C-G	1	Ighv5-4	missense_variant	L7F	1314.71	52.34
11-57289373-57289374-C-T	5	Gria1	missense_variant	L717F	2795.74	280.93
17-47869256-47869257-C-T	1	Foxp4	missense_variant	S586N	376.782	424.708
1-107271842-107271843-C-T	1	Serpinb3c	missense_variant	S316N	359.948	425.853
14-32659999-32660000-T-G	1	3425401B19Rik	missense_variant	Q1336P	1179.859	68.522
4-100441382-100441383-A-T	5	Ror1	missense_variant	K651M	89.14	83.537
14-53337566-53337567-G-A	1	Trav13n-3	missense_variant	E89K	690.383	414.311
X-127063259-127063260-G-A	2	Gm382	missense_variant	V1023I	2781.922	711.664
8-105375702-105375703-A-G	1	Plekhg4	missense_variant	N108S	2405.803	914.661
4-88683332-88683333-C-G	5	Ifna2	missense_variant	R149S	266.59	80.72
12-113306426-113306427-A-G	2	Ighg2b	missense_variant	Y325H	337.357	474.526
5-3344457-3344458-G-C	1	Cdk6	missense_variant	R31P	908.364	807.718
17-37589975-37589976-C-T	1	Olfr114	missense_variant	V126I	37.005	45.61
11-78287788-78287789-T-A	1	2610507B11Rik	missense_variant	F2006I	5471.293	248.365
5-52848759-52848760-A-T	1	Anapc4	missense_variant	L330F	1066.817	304.853
3-101439441-101439442-G-C	1	Igsf3	missense_variant	L564F	435.341	306.782
X-64178266-64178267-T-C	2	3830417A13Rik	missense_variant	F276S	1130.472	503.605
X-74303841-74303842-G-A	1	Atp6ap1	missense_variant	D410N	1535.536	877.526
5-16302320-16302321-A-C	1	Cacna2d1	missense_variant	K356T	431.519	498.884
9-18856953-18856954-G-A	1	Olfr829	missense_variant	V110I	423.79	123.63

7-140336052-140336053-T-G	2	Olfr527	missense_variant	L64V	105.017	105.017
1-174449438-174449439-C-T	4	Olfr220	missense_variant	A272V	1824.253	455.08
2-88025360-88025361-T-G	1	Olfr1161	missense_variant	L213R	566.018	478.443
7-107129167-107129168-G-A	2	Olfr715	missense_variant	T75I	759.397	480.939
2-180733805-180733806-T-A	1	Slc17a9	missense_variant	S174T	337.33	325.46
9-20588518-20588519-C-T	2	Zfp846	missense_variant	T15I	344.913	685.299
12-103587518-103587519-T-C	1	Ppp4r4	missense_variant	V412A	1585.222	800
16-32751992-32751993-C-A	2	Muc4	missense_variant	P624T	62.374	69.677
17-37590017-37590018-T-C	1	Olfr114	missense_variant	M112V	350.825	497.84
10-51725419-51725420-C-T	1	Rfx6	missense_variant	P646S	3724.689	543.001
17-23291793-23291794-A-G	2	Vmn2r114	missense_variant	C571R	788.642	603.34
17-45568433-45568435-CA-TG	1	Hsp90ab1	missense_variant	M620T	80.11	261.873
16-29260943-29260944-C-G	2	Atp13a5	missense_variant	A939P	157.493	297.228
1-118662019-118662020-C-T	1	Tfcp2l1	missense_variant	T270I	2371.865	912.2
1-155789391-155789392-C-T	2	Qsox1	missense_variant	G268R	260.178	373.474
4-24596142-24596143-C-T	1	Mms22l	missense_variant	S1074F	611.631	579.421
9-104123116-104123117-C-T	2	Acad11	missense_variant	A690V	762.535	410.289
1-85610765-85610766-G-A	1	Sp140	missense_variant	V75I	2427.98	937.57
X-100594004-100594005-G-A	2	P2ry4	missense_variant	H96Y	77.999	75.525
8-82864146-82864147-C-G	1	Rnf150	missense_variant	I46M	267.87	387.87
8-125447857-125447858-C-T	1	Sipa1l2	missense_variant	S1227N	587.611	580.885
7-23984778-23984779-T-C	1	Vmn1r181	missense_variant	L223P	771.063	753.512
2-172551490-172551491-C-T	1	Tfap2c	missense_variant	P84S	4594.73	534.675
9-44818767-44818768-C-T	2	Kmt2a	missense_variant	M3414I	127.391	100.482
9-103479866-103479867-C-A	1	Bfsp2	missense_variant	M120I	240.93	431.59
17-33269092-33269093-G-A	1	Olfr63	missense_variant	R123H	59.09	71.795
2-130041822-130041823-G-C	1	Tgm3	missense_variant	G467A	1485.268	585.724
12-53947199-53947200-A-G	1	Npas3	missense_variant	H204R	1906.3	882.45
15-50660861-50660862-C-G	1	Trps1	missense_variant	C1224S	732.825	730.853
6-28526128-28526129-G-A	1	Snd1	missense_variant	G169S	736.36	428.637
13-21674751-21674752-G-T	2	Olfr1360	missense_variant	T64N	180.097	196.38
2-86639305-86639306-A-G	2	Olfr1084	missense_variant	V134A	429.625	232.322
12-114746448-114746449-T-A	1	Ighv1-22	missense_variant	S59C	534.884	783.899
7-103155588-103155589-T-G	1	Olfr589	missense_variant	T53P	128.777	300.67
7-8471616-8471617-C-G	1	Vmn2r45	missense_variant	G804A	419.643	727.579
10-102385080-102385081-G-A	1	Mgat4c	missense_variant	D75N	668.64	344.81
X-102071364-102071365-C-T	2	Nhsl2	missense_variant	H108Y	8876.873	421.949
2-86131198-86131199-C-T	3	Olfr1039	missense_variant	V155M	4120.975	339.625
17-34907588-34907589-A-G	1	Ehmt2	missense_variant	N782S	1995.676	848.69
6-57002713-57002714-A-C	2	Vmn1r6	missense_variant	K120N	202.768	94.189
4-62499759-62499760-A-T	1	Hdhd3	missense_variant	F60I	1130.03	51.297
4-9537694-9537695-C-T	1	Asph	missense_variant	R361K	1312.472	713.001
4-40190786-40190787-C-T	2	Aco1	missense_variant	A717V	385.19	678.73
8-3876306-3876307-C-T	1	Cd209d	missense_variant	A133T	1378.08	922.678
12-115193691-115193692-C-T	1	Ighv1-54	missense_variant	V112I	2270.335	405.442
14-67724570-67724571-A-G	1	Kctd9	missense_variant	Y21C	579.421	579.421
8-105949816-105949817-C-G	2	Slc12a4	missense_variant	G491A	1179.995	386.16
16-30308613-30308614-G-A	1	Gp5	missense_variant	T414I	149.624	111.643
17-20042712-20042713-T-G	1	Vmn2r104	missense_variant	Q162P	18551.13	727.78
11-116677607-116677608-G-A	1	St6galnac2	missense_variant	P343L	633.695	753.147
3-62419694-62419695-A-G	1	Arhgef26	missense_variant	K543R	1131.957	663.483
4-132833090-132833091-G-A	1	Ppp1r8	missense_variant	R100W	1785.201	874.088
X-102520774-102520775-T-G	3	Phka1	missense_variant	K1092N	61.389	99.86
X-74303847-74303848-G-T	1	Atp6ap1	missense_variant	A412S	1535.536	337.19
7-21133364-21133365-A-C	2	Vmn1r122	missense_variant	F255C	562.92	363.153

12-115461046-115461047-C-G	3	Ighv1-62-3	missense_variant	Q101H	100.95	171.32
10-75638159-75638160-G-A	1	Susd2	missense_variant	H654Y	1815.73	109.39
17-80216999-80217000-C-G	3	Ttc39d	missense_variant	L363V	454.988	265.461
11-114751822-114751823-C-T	1	Dnaic2	missense_variant	P417S	209.552	243.824
8-20944652-20944653-C-T	2	AY761185	missense_variant	D20N	107.421	101.88
10-22371399-22371400-C-T	1	Raet1d	missense_variant	P125L	7421.79	259.48
19-11472471-11472472-A-C	4	Ms4a6c	missense_variant	N76T	95.556	70.349
1-85635574-85635575-A-C	1	Sp140	missense_variant	N330T	1663.757	881.91
2-87269648-87269649-C-A	2	Olfr1116	missense_variant	F268L	246.752	368.205
5-128797656-128797657-C-T	1	Rimbp2	missense_variant	G297E	274.641	186.247
2-30113327-30113328-C-A	1	Zer1	missense_variant	G26C	275.86	196.2
9-27019335-27019336-C-T	1	Vps26b	missense_variant	G121E	166.441	223.404
7-103517987-103517988-A-C	1	Olfr611	missense_variant	L132R	433.221	500.853
17-12719893-12719894-A-C	1	Igf2r	missense_variant	F508V	390.176	773.8
10-81362244-81362245-G-C	1	Mfsd12	missense_variant	G320A	163.305	143.88
17-35380588-35380590-CC-GG	1	H2-Q4	missense_variant	S216W	128.777	170.612
X-143101994-143101995-G-T	2	Rtl9	missense_variant	R801I	92.276	118.099
3-36076046-36076047-G-A	3	Acad9	missense_variant	G213D	138.909	205.712
12-115208513-115208514-C-T	2	Ighv1-55	missense_variant	C5Y	11604.036	598.537
14-121912246-121912247-G-C	1	Gpr18	missense_variant	A122G	302.91	826.51
11-58529827-58529828-C-T	1	Olfr330	missense_variant	V53I	4626.579	953.367
16-57133127-57133128-A-C	1	Tomm70a	missense_variant	K129Q	283.962	369.199
17-34148621-34148622-G-T	1	H2-DMb2	missense_variant	V137F	6439.172	457.963
11-58529560-58529561-A-G	1	Olfr330	missense_variant	Y142H	767.88	503.78
2-129208196-129208197-C-T	3	Slc20a1	missense_variant	T425I	97.1	110.676
5-15580673-15580674-A-C	1	Gm21083	missense_variant	N159T	1476.59	826.533
15-36234652-36234653-A-T	1	Spag1	missense_variant	K853I	2646.306	995.274
9-32259654-32259655-C-T	2	Arhgap32	missense_variant	P1593S	594.415	179.995
2-87435373-87435374-T-A	1	Olfr1124	missense_variant	Y296N	146.43	326.54
17-35471086-35471087-C-T	3	H2-Q10	missense_variant	T202M	143.492	268.762
12-114222989-114222990-C-T	1	Ighv7-4	missense_variant	S54N	696.429	803.693
7-42136836-42136837-T-C	1	Vmn2r60	missense_variant	F355L	237.684	239.883
8-85171760-85171761-T-C	1	Cks1brt	missense_variant	L81P	495.119	479.34
18-45685368-45685369-C-T	2	Kcnn2	missense_variant	T511I	957.767	743.464
9-38606772-38606773-T-G	2	Olfr914	missense_variant	F103V	51.895	73.365
12-112735255-112735256-C-T	1	Cep170b	missense_variant	P190S	6824.801	659.311
17-19811942-19811943-A-G	1	Vmn2r103	missense_variant	T660A	238.605	265.87
2-89283062-89283063-T-G	1	Olfr1229	missense_variant	K23N	74.97	84.446
2-85400458-85400459-G-A	2	Olfr992	missense_variant	H25Y	1383.025	288.955
1-182748902-182748903-G-C	2	Ccdc185	missense_variant	P74A	604.074	502.643
2-36859574-36859575-A-C	3	Olfr351	missense_variant	L258V	126.29	207.65
7-23835106-23835107-A-T	1	Vmn1r176	missense_variant	V207E	839.963	762.43
16-16868672-16868673-C-T	1	Vpreb1	missense_variant	G118R	432.636	352.242
11-51593953-51593954-A-C	1	Phykpl	missense_variant	E247A	2046.869	939.918
7-103328662-103328663-A-G	1	Olfr598	missense_variant	H59R	553.681	940.286
6-126101834-126101835-G-A	1	Ntf3	missense_variant	T236I	357.347	255.912
7-102742199-102742200-C-T	1	Olfr78	missense_variant	D268N	1734.67	650.05
2-88794939-88794940-C-T	1	Olfr1201	missense_variant	A186V	1209.273	564.716
10-30647991-30647992-T-G	1	Ncoa7	missense_variant	S891R	292.51	660.252
12-98815984-98815985-T-G	2	Eml5	missense_variant	D1396A	437.17	131.72
5-138988374-138988375-A-G	1	Pdgfa	missense_variant	V80A	1210.849	605.049
7-111079319-111079320-T-G	1	Eif4g2	missense_variant	K108N	863.495	504.766
9-15352941-15352942-C-T	1	Cep295	missense_variant	S136N	94.643	275.48
16-87722516-87722517-G-A	1	Bach1	missense_variant	R565H	1381.211	893.491
