# Supplementary material for: Neoantigen-augmented iPSC cancer vaccine combined with radiotherapy promotes antitumor immunity in poorly immunogenic cancers
Source: NPJ Vaccines. 2024 May 31;9:95. doi: 10.1038/s41541-024-00881-5 (PMC11143272; doi:10.1038/s41541-024-00881-5)
Supplement: Supplementary file 2 — REPORTING SUMMARY [file 41541_2024_881_MOESM2_ESM.pdf]

nature portfolio

Corresponding author(s): Professor KS Clifford Chao

Last updated by author(s): Apr 3, 2024

Reporting Summary

Nature Portfolio wishes to improve the reproducibility of the work that we publish. This form provides structure for consistency and transparency in reporting. For further information on Nature Portfolio policies, see our Editorial Policies and the Editorial Policy Checklist.

Statistics

For all statistical analyses, confirm that the following items are present in the figure legend, table legend, main text, or Methods section.

n/a	Confirmed	
☐	☒	The exact sample size (*n*) for each experimental group/condition, given as a discrete number and unit of measurement
☐	☒	A statement on whether measurements were taken from distinct samples or whether the same sample was measured repeatedly
☐	☒	The statistical test(s) used AND whether they are one- or two-sided<br>*Only common tests should be described solely by name; describe more complex techniques in the Methods section.*
☐	☒	A description of all covariates tested
☐	☒	A description of any assumptions or corrections, such as tests of normality and adjustment for multiple comparisons
☐	☒	A full description of the statistical parameters including central tendency (e.g. means) or other basic estimates (e.g. regression coefficient) AND variation (e.g. standard deviation) or associated estimates of uncertainty (e.g. confidence intervals)
☐	☒	For null hypothesis testing, the test statistic (e.g. *F*, *t*, *r*) with confidence intervals, effect sizes, degrees of freedom and *P* value noted<br>*Give P values as exact values whenever suitable.*
☒	☐	For Bayesian analysis, information on the choice of priors and Markov chain Monte Carlo settings
☒	☐	For hierarchical and complex designs, identification of the appropriate level for tests and full reporting of outcomes
☒	☐	Estimates of effect sizes (e.g. Cohen's *d*, Pearson's *r*), indicating how they were calculated

Our web collection on statistics for biologists contains articles on many of the points above.

Software and code

Policy information about availability of computer code

Data collection	No software was used.
Data analysis	PRISM 9 GraphPad software.

For manuscripts utilizing custom algorithms or software that are central to the research but not yet described in published literature, software must be made available to editors and reviewers. We strongly encourage code deposition in a community repository (e.g. GitHub). See the Nature Portfolio guidelines for submitting code & software for further information.

Data

Policy information about availability of data

All manuscripts must include a data availability statement. This statement should provide the following information, where applicable:

- Accession codes, unique identifiers, or web links for publicly available datasets
- A description of any restrictions on data availability
- For clinical datasets or third party data, please ensure that the statement adheres to our policy

All RNA-seq are deposited in a publicly accessible NCBI database GSE262852 (https://www.ncbi.nlm.nih.gov/geo/query/acc.cgi?acc=GSE262852). The other datasets used and/or analyzed during the current study are available from the corresponding author on reasonable request.

Research involving human participants, their data, or biological material

Policy information about studies with human participants or human data. See also policy information about sex, gender (identity/presentation), and sexual orientation and race, ethnicity and racism.

Reporting on sex and gender	NA
Reporting on race, ethnicity, or other socially relevant groupings	NA
Population characteristics	NA
Recruitment	NA
Ethics oversight	NA

Note that full information on the approval of the study protocol must also be provided in the manuscript.

Field-specific reporting

Please select the one below that is the best fit for your research. If you are not sure, read the appropriate sections before making your selection.

☒ Life sciences ☐ Behavioural & social sciences ☐ Ecological, evolutionary & environmental sciences

For a reference copy of the document with all sections, see nature.com/documents/nr-reporting-summary-flat.pdf

Life sciences study design

All studies must disclose on these points even when the disclosure is negative.

Sample size	For cell-based experiments, each experiment included at least three samples. For animal experiments, each group includes at least 5-8 mice. All of the data are expressed as the mean ± SEM. Intergroup differences were appropriately assessed by either unpaired two-tailed Student's t test or one-way analysis of variance (ANOVA) with multiple comparison tests using PRISM 9 GraphPad software.
Data exclusions	*Describe any data exclusions. If no data were excluded from the analyses, state so OR if data were excluded, describe the exclusions and the rationale behind them, indicating whether exclusion criteria were pre-established.*
Replication	The cell-based experiments were repeated at least three times, independently. The animal experiments were repeated twice, independently.
Randomization	After inoculation with CT26 cancer cells for five days, mice were randomly divided into different subgroups.
Blinding	All experiments did not blinded during data collection and allocation.

Reporting for specific materials, systems and methods

We require information from authors about some types of materials, experimental systems and methods used in many studies. Here, indicate whether each material, system or method listed is relevant to your study. If you are not sure if a list item applies to your research, read the appropriate section before selecting a response.

Materials & experimental systems

n/a	Involved in the study	
☐	☒	Antibodies
☐	☒	Eukaryotic cell lines
☒	☐	Palaeontology and archaeology
☐	☒	Animals and other organisms
☒	☐	Clinical data
☒	☐	Dual use research of concern
☒	☐	Plants

Methods

n/a	Involved in the study	
☒	☐	ChIP-seq
☐	☒	Flow cytometry
☒	☐	MRI-based neuroimaging

Antibodies

Antibodies used	For western blot: Glud1 (1:1000, A7631, ABclonal, MA, USA), Mtch1 (1:1000, A8063, ABclonal), E2F8 (1:1000, A1135, ABclonal), and cleaved caspase-3 (1:1000, #9661, Cell Signaling and IR96-401, iReal Biotech., Taipei, Taiwan), p-AktS473 (1:1000, #9271, Cell

Signaling, CA, USA) and GAPDH (1:3000, IR3-8, iReal Biotech., Taipei, Taiwan). For IHC analysis: anti-mouse CD11c (1:300, ab219799, Abcam, Cambridge, UK), anti-mouse CD8a (1:300, ab217344, Abcam), anti-mouse GzmB (1:300, ab255598, Abcam), anti-NKG2D antibody (1:300, ab203353) and Ki67 (1:300, ab15580, Abcam). For flow cytometric analysis: (1) CD8/MDSC panel: PerCP/Cy5 anti-mouse CD8a (1:100, clone 53-6.7, Cat#100732, BioLegend, CA, USA), FITC anti-human/mouse CD11b (1:100, clone M1/70, Cat#101206, BioLegend), PE/Cy7 anti-mouse CD45 (1:100, clone 30-F11, Cat#103114, BioLegend), APC anti-mouse Gr-1 (1:100, clone RB6-8C5, Cat#108412, BioLegend) and PE anti-mouse CD44 (1:100, clone QA19A43, Cat#163610, BioLegend); (2) Foxp+ Treg panel: APC anti-mouse CD25 (1:100, clone PC61, Cat#102012, BioLegend), APC/Cy7 anti-mouse CD4 (1:100, clone RM4-5, Cat#100526, BioLegend), PerCP/Cy5 anti-mouse CD8a (1:100, clone 53-6.7, Cat#100732, BioLegend) and PE anti-mouse FOXP3 (1:100, clone QA20A67, Cat#118904, BioLegend); (3) IFN[illegible]+CD8+ T cell panel: FITC anti-mouse IFNγ (1:100, clone XMG1.2, Cat#505806, BioLegend), PerCP/Cy5 anti-mouse CD8a (1:100, clone 53-6.7, Cat#100732, BioLegend) and PE/Cy7 anti-mouse CD45 (1:100, clone 30-F11, Cat#103114, BioLegend), and their isotypes (BioLegend).

Validation: Each antibodies were validated and showed in the manufactures' website.

Eukaryotic cell lines

Policy information about cell lines and Sex and Gender in Research

Cell line source(s): Mouse CT26 colorectal cancer cell line and 4T1 breast cancer cell line were purchased from American Type Culture Collection (ATCC). Murine iPSCs from female BALB/c mice were generated by Sendai reprogramming kit.

Authentication: They were not further authenticated but were cultured for a limited number of passages (<10 passages).

Mycoplasma contamination: CT26, 4T1 and mouse iPSC cell line were routinely tested for mycoplasma contamination by PCR.

Commonly misidentified lines (See ICLAC register): NA

Animals and other research organisms

Policy information about studies involving animals; ARRIVE guidelines recommended for reporting animal research, and Sex and Gender in Research

Laboratory animals: Female Balb/c mice

Wild animals: NA

Reporting on sex: Female Balb/c mice

Field-collected samples: *For laboratory work with field-collected samples, describe all relevant parameters such as housing, maintenance, temperature, photoperiod and end-of-experiment protocol OR state that the study did not involve samples collected from the field.*

Ethics oversight: *Identify the organization(s) that approved or provided guidance on the study protocol, OR state that no ethical approval or guidance was required and explain why not.*

Note that full information on the approval of the study protocol must also be provided in the manuscript.

Plants

Seed stocks: NA

Novel plant genotypes: NA

Authentication: NA

Flow Cytometry

Plots

Confirm that:

☒ The axis labels state the marker and fluorochrome used (e.g. CD4-FITC).

☒ The axis scales are clearly visible. Include numbers along axes only for bottom left plot of group (a 'group' is an analysis of identical markers).

☒ All plots are contour plots with outliers or pseudocolor plots.

☒ A numerical value for number of cells or percentage (with statistics) is provided.

Methodology

Sample preparation	Tumors and spleens from the mice were isolated and weighed and then placed in petri dishes containing basal RPMI media at room temperature to prevent dehydration, as previously described 60, 61. The tumor and spleen were minced into small pieces (1-2 mm) using a beaver blade, filtered through a 70 µm strainer, spun down, and resuspended in basal RPMI media. Thereafter, the cell suspensions were layered over Ficoll-Paque medium and centrifuged at 1,025 × g for 20 min. The mononuclear cell layer was transferred into a conical tube, and 20 ml of complete RPMI medium was added and then gently mixed and centrifuged at 650 × g for 10 min. Finally, the supernatant was removed, and splenic and tumor-infiltrating lymphocytes (TILs) were resuspended in complete RPMI medium.<br>TILs were then resuspended in 500 uL of staining buffer (2% BSA and 0.1% NaN3 in PBS). The cells were stained with a surface marker panel and intracellular cytokines by different panel antibodies. The intracellular marker Foxp3 was fixed and permeabilized by a FOXP3 Fix/Perm buffer set before staining (Cat#421403, Biolegend). The intracellular marker INFg was activated by the protein transport inhibitor brefeldin A for 3 h before staining.
Instrument	Guava® easyCyte™ Flow Cytometer (Luminex, CA, USA)
Software	FlowJo v10.0.7 software (Ashland, USA).
Cell population abundance	The cell population abundance was shown and presented as %.
Gating strategy	All the gating strategies were shown in the text and supplementary information

☒ Tick this box to confirm that a figure exemplifying the gating strategy is provided in the Supplementary Information.
